# Using systems biology and drug repositioning approaches to discover FDA-approved drugs candidates for endometriosis treatment

**DOI:** 10.1371/journal.pone.0330841

**Published:** 2025-09-12

**Authors:** Samira Sanami, Shahrzad Aghaamoo, Sajjad Ahmad, Ali Fazli, Bahareh Mansouri, Jonas Ivan Nobre Oliveira, Mojgan Rahmanian

**Affiliations:** 1 Abnormal Uterine Bleeding Research Center, Semnan University of Medical Sciences, Semnan, Iran; 2 Department of Health and Biological Sciences, Abasyn University, Peshawar, Pakistan; 3 Department of Biophysics and Pharmacology, Federal University of Rio Grande do Norte, Natal, Rio Grande do Norte, Brazil; AMET University, INDIA

## Abstract

Endometriosis is characterized by the presence of endometrial tissue outside the uterine cavity. The administration of drugs designated for this condition has significant adverse effects, such as signs of estrogen insufficiency and suppression of ovulation. Considering this issue, this study aims to employ drugs repurposing approaches for the treatment of endometriosis. The GSE120103 dataset was selected to assess the expression of genes involved in endometriosis, then differentially expressed genes (DEGs) were identified using the “Limma” package in R Studio. Functional and pathway enrichment analysis of 708 up-regulated and 414 down-regulated DEGs was performed using ShinyGO 0.81 and DAVID tools. the Search Tool for the Retrieval of Interacting Genes/Proteins (STRING) was then used to build a protein-protein interaction (PPI) network of up-regulated DEGs, followed by Cytoscape software to identify hub genes. Vascular endothelial growth factor receptor 2 (VEGFR2) and interleukin-6 (IL-6) were identified as hub genes, assessments suggested that VEGFR2 may be a more promising possibility for druggability than IL-6. 16 FDA-approved drugs targeting VEGFR2 were identified, and molecular docking analysis indicated that ponatinib (−9.6 kcal/mol) had a more favorable binding energy than the co-crystal ligand (−9.2 kcal/mol). Moreover, molecular dynamics (MD) simulation analysis demonstrated considerable stability of the VEGFR2-ponatinib complex over a 100 nanoseconds (ns) timescale. The findings of this study indicate that ponatinib may provide considerable therapeutic promise for the treatment of endometriosis. Nevertheless, additional experimental investigations are required to evaluate its therapeutic efficacy.

## Introduction

Endometriosis impacts around 10% (190 million) of women and girls of reproductive age worldwide [1]. Endometriosis is a multifaceted and systemic clinical disease that can adversely affect women’s reproductive health and quality of life; it may commence with an individual’s first menstrual period and persist until menopause [1]. This condition’s pathological hallmark is abnormal endometrial tissue growth in the uterine outer regions [2]. The ovaries and pelvic peritoneum are the predominant locations for the development of endometriotic lesions. Endometriotic lesions may also occur in other locations, including the fallopian tubes, abdominal wall, intestines, cervix, bladder, and vagina [3,4]. A comprehensive understanding of the pathogenesis of endometriosis may have significant clinical and therapeutic ramifications. Endometriosis has a complex pathophysiology that involves several pathways, including genetics, abnormal endocrine signaling, dysregulated cell growth and death, ectopic endometrial tissue, and altered immunity [5].

Endometriosis is a chronic condition that induces significant pain during menstruation, sexual intercourse, urination, or defecation. Endometriosis symptoms encompass chronic pelvic pain, bloating, nausea, fatigue, and occasionally depression, anxiety, and infertility. Symptoms of endometriosis encompass abdominal distension accompanied by infection, irregular uterine hemorrhage, vaginal discharge, pelvic pain, and discomfort in the lower abdomen. Additional symptoms of endometriosis encompass constipation accompanied by stomach or intestinal pain [1,6,7]. The sole definitive method for diagnosing endometriosis requires surgical intervention or laparoscopy, leading to a latency period of 7–11 years from the onset of symptoms to a conclusive diagnosis [8], and entails an increased risk of disease progression during that time [9]. In recent years, noninvasive imaging techniques, particularly transvaginal ultrasonography and magnetic resonance imaging (MRI), have improved diagnostic accuracy for endometriosis, enabling the staging and classification of different types of the endometriosis without the need for surgical procedures [10–12].

Drug repurposing denotes the identification of novel therapeutic applications for established medications to improve their efficacy and optimize their use [13]. The advantages of Food and Drug Administration (FDA)-approved drug repositioning are numerous, and since many preclinical and clinical trials have already been carried out on these drugs, their use saves time and cost as compared to developing de novo drugs [14]. The primary hurdles involve identifying drug target proteins (receptors) associated with diseases and discovering pharmacological agents (small molecules) that can mitigate these disorders through contact with the target proteins. The identification of hub genes via bioinformatics has become an effective method for discovering prospective biomarkers and therapeutic targets [15]. Transcriptomics analysis is a prominent strategy for discovering genetic biomarkers. Proteins produced by genomic biomarkers are regarded as key receptors [16–18].

Endometriosis treatment options currently include drugs, surgical procedures, surgery combined with drugs, and assisted reproductive technology (ART). While surgery remains an appropriate method for endometriosis-related pelvic pain, its efficacy is undermined by limitations, including a 40−50% recurrence rate after surgery [19]. Administering drugs to reduce related symptoms is preferable for patients without surgical indications. First-line pharmacological treatments comprise non-steroidal anti-inflammatory drugs (NSAIDs), progestins, and oral contraceptives (OCs). Second-line drugs comprise gonadotropin-releasing hormone agonists (GnRH-a) and the levonorgestrel intrauterine delivery method. Nonetheless, the administration of the mentioned drugs, including GnRH-a, is associated with significant adverse effects, such as symptoms of estrogen insufficiency and inhibition of ovulation. Consequently, it is inappropriate for prolonged usage, particularly for individuals with reproductive requirements [20]. Given these limitations, there is an urgent need for identification of new biological targets and drugs. Zhang *et al*.‘s study indicated that the primary biological targets of tanshinone IIA, the active component of Chinese medicine Danshen (*Salvia miltiorrhiza* Bge.), for endometriosis treatment are vascular endothelial growth factor A (VEGFA), matrix metalloproteinase 9 (MMP-9), estrogen receptor-1 (ESR1), intercellular adhesion molecule (ICAM)‑1, and interleukin-2 (IL‑2), which exert pathological effects on the adhesion, invasion, and angiogenesis of ectopic endometrial tissue within the pelvic and abdominal cavities in endometriosis [21].

The current study also aimed to find hub genes in endometriosis, identify and evaluate FDA-approved drugs that target the proteins encoded by these genes. In the present study, we first downloaded GSE120103 dataset from the Gene Expression Omnibus (GEO). After pre-processing and normalizing the data by the “Limma” package in R Studio, we identified DEGs. Next, Gene ontology (GO) and Kyoto Encyclopedia of Genes and Genomes (KEGG) enrichment analyses were performed by the ShinyGO 0.81 and DAVID tools, respectively. PPI network was constructed using the Search Tool for the Retrieval of Interacting Genes/Proteins (STRING), and Cytoscape software was used to identify cluster modules and hub genes. Then, target transcription factors (TFs) and microRNAs (miRNAs) of selected hub genes were predicted by NetworkAnalyst tool. Subsequently, the druggability of hub genes and their associated TFs was assessed using the Drug-Gene Interaction Database (DGIdb 5.0). The DrugBank database was searched to find FDA-approved drugs that were ligands for the target protein, and then their virtual screening was performed using PyRx 0.8 software. Finally, molecular dynamics (MD) simulation of the target protein and docked complexes were performed by the Assisted Model Building with Energy Refinement (AMBER) 18 program.

## Materials and methods

### Data collection

The GSE120103 dataset was obtained from the GEO (https://www.ncbi.nlm.nih.gov/geo/(database (accessed on 3 February 2025) [22]. This gene expression profile was generated using the GPL6480 (Agilent-014850 Whole Human Genome Microarray 4x44K G4112F) platform and derived from a study conducted by Bhat *et al* [23]. The GSE120103 dataset contains 9 human endometrial tissue samples from each of the four groups: fertile women with endometriosis (FE), fertile women without endometriosis (FC), infertile women with endometriosis (IE), and infertile women without endometriosis (IC).

### Identification of differentially expressed genes (DEGs)

Using “Limma” package [24] in R Studio [25], we identified differentially expressed genes (DEGs) between the FE and FC groups, as well as between the IE and IC groups, in the GSE120103 dataset. DEGs were selected based on |log2 fold change (FC)| > 1 and adjusted P-value < 0.05. The “ggplot2” package in RStudio was used to create the volcano plot [26]. The “Venn” package in R Studio was used to screen for common up- and down-regulated DEGs in the FE and IE groups [27].

### Functional and pathway enrichment analysis of DEGs

GO functional analysis and KEGG pathway assessment of common up- and down-regulated DEGs between the FE and IE groups were conducted using ShinyGO 0.81 (https://bioinformatics.sdstate.edu/go/) (accessed on 5 February 2025) [28] and DAVID (https://davidbioinformatics.nih.gov/) (accessed on 5 February 2025) [29], respectively. The GO terms are divided into three categories: biological processe (BP), molecular function (MF), and cellular component (CC). In the analysis conducted in the ShinyGO 0.81 tool, False Discovery Rate (FDR) value of 0.05 was used as the significance cutoff, as well as, the significance level in the analysis conducted in the DAVID tool was set to P- value < 0.05. The ShinyGO 0.81 tool used an FDR value of 0.05 as the significance cutoff, while the DAVID tool used a P-value < 0.05 as the significance level.

### PPI network analysis and hub gene identification

We used the STRING (version: 12.0) (https://string-db.org/) (accessed on 6 February 2025) to develop a protein-protein interaction (PPI) network of shared up-regulated DEGs in the FE and IE groups. A minimum required interaction score of 0.700 (high confidence) was set for the generation of this interaction network. The database currently comprises 12535 organisms, 59.3 million proteins, and more than 20 billion documented interactions [30]. We then used the Cytoscape software (version 3.10.3) to further analyze and visualize the PPI network. Cytoscape is an open-source software platform designed for visualizing intricate networks and merging them with many types of attribute data [31]. We next used the CytoHubba plugin [32] to identify hub genes based on five topological analysis algorithms: Betweenness [33], BottleNeck [34], Closeness [35], Degree [36], and Stress [37]. The top 10 genes were chosen based on each method, and the shared genes among these five algorithms were considered as the final hub genes.

### Regulatory network analysis of hub genes

The NetworkAnalyst tool (https://www.networkanalyst.ca/) (accessed on 7 February 2025) was used to map interactions among important transcriptional regulatory transcription factors (TFs) and post-transcriptional regulatory microRNAs (miRNAs) with hub genes [38–40]. The JASPAR [41] and miRTarBase v9.0 [42] databases were used to generate the TFs-hub genes network and the miRNAs-hub genes network, respectively.

### Identification of druggable genes

The DGIdb 5.0 (https://dgidb.org/) (accessed on 8 February 2025) was used to analyze the druggability of hub genes and their related TFs identified in our study. The DGIdb provides information regarding drug-gene interactions and druggability sourced from publications, databases, and various online resources [43]. This database categorizes genes as potentially druggable based on their presence in selected pathways, molecular functions, and gene families from the Gene Ontology, the Human Protein Atlas, IDG, “druggable genome” lists from Hopkins and Groom (2002) [44] and Russ and Lampel (2005) [45].

### Retrieval and preparation of protein target structure and FDA-approved drugs

The target protein’s X-ray crystal structure was obtained from the RCSB Protein Data Bank (PDB) (https://www.rcsb.org/) (accessed on 9 February 2025) in PDB format (Supplementary S1 File) [46]. We removed all co-crystallized ligands, heteroatoms, and associated crystal water molecules from the parent structure using the UCSF Chimera software (version 1.10.2) [47] to ensure that pre-bound molecules did not influence the binding affinity of the predicted drugs. To fill in the missing residues in the target protein’s crystal structure, homology modeling was performed using SWISS-MODEL software (https://swissmodel.expasy.org/) (accessed on 9 February 2025) and the parent sequence as a template [48]. The pKa values of the ionizable groups in the protein were ascertained using PROPKA version 3.1, facilitating the optimization of the hydrogen bond network at pH 7.4 [49]. Energy minimization was done using UCSF Chimera software (version 1.10.2) with default parameters such as steepest descent steps of 1000, steepest descent step size of 0.02 Å, conjugate step gradient of 10, conjugate steps of 0.02 Å, and update interval of 100. The DrugBank database (https://go.drugbank.com/) (accessed on 10 February 2025) [50] was searched to identify the FDA-approved drugs that formed ligands for the target protein during the docking analysis. Among the identified drugs, those with inhibitory activity and available structures were retrieved from the PubChem database (https://pubchem.ncbi.nlm.nih.gov/) (accessed on 10 February 2025) in structure-data file (SDF) format [51].

### Molecular docking-based virtual screening

Molecular docking-based virtual screening was conducted to identify drugs with high binding affinity for the target protein. The AutoDock Vina Wizard [52] in PyRx 0.8 software was utilized for screening [53]. The appropriate file formats for the receptor and ligand for molecular docking were generated in PDBQT format using PyRx 0.8 software. Active sites were defined as regions within the target protein structure that were less than 5 Å distant from the co-cocrystal ligands. The molecular docking analysis was conducted using dimensions of X = 29.3683 Å, Y = 23.2777 Å, and Z = 17.8267 Å, respectively, and the center coordinates for X, Y, and Z were set as −1.5459, 35.5985, and 14.8893, respectively. The grid box contained all of the residues required for binding, including Leu35, Gly36, Arg37, Gly38, Gly41, Val43, Ala61, Lys63, Glu80, Ile83, Leu84, Ile87, Val93, Val94, Val111, Glu112, Phe113, Cys114, Leu164, His171, Leu180, Ile189, Cys190, and Asp191. To validate the docking method, we also performed redocking of the co-crystal ligand with the target protein. Following docking, hit drugs candidates were chosen based on binding affinity scores.

### Interaction analysis in docked complexes

The analysis of protein-ligand interactions is a crucial component of the drug discovery process, since it elucidates the molecular mechanisms that may account for the efficacy or ineffectiveness of specific drugs [54]. In this study, the ProLIF package was used to visualize docking results and analyze interactions between receptor protein and ligand residues in the docked complexes. ProLIF (Protein-Ligand Interaction Fingerprints) is a Python utility for generating interaction fingerprints for molecular complexes. These fingerprints are vector representations of molecular interactions in three dimensions. This occurs frequently between proteins and ligands [55].

### Molecular dynamics simulation

The dynamic behavior of unbound receptor and docked complexes was investigated using MD simulation. The simulation protocol was executed with the AMBER 18 program [56]. The primary coordinates were derived from the unbound receptors, docked complexes, and topology files were prepared utilizing the tLEAP interface of AMBER 18. System solvation was performed with three-point transferable intermolecular potential (TIP3P) water, and the force fields used for computations were General Amber Force Field (GAFF) [57] and ff99SB. To neutralize the system’s overall charge, sodium counter-ions were introduced. To eliminate steric clashes, the docked protein complex underwent minimization through 1500 steepest descent and 1000 conjugate gradient steps. Langevin dynamics were utilized for system heating over 10 picoseconds (ps) [58], followed by 100 ps of equilibration in the canonical (NVT) ensemble. During the production run, hydrogen bonds were constrained using the SHAKE algorithm [59]. The temperature of the system was incrementally raised from 0 to 300 K over 200 ps at constant volume, after which the system was equilibrated at constant pressure. The production run lasted for a total of 100 nanoseconds (ns). The final MD trajectories for unbound receptor and docked complexes was examined. The analysis includes generation root mean square deviation (RMSD), root mean square fuctuation (RMSF), radius of gyration (Rg), and solvent accessible surface area (SASA) values for free receptor and docked complexes.

### MM-PBSA/GBSA analysis

The molecular mechanics Poisson–Boltzmann surface area (MM-PBSA) and molecular mechanics generalized Born surface area (MM-GBSA) methods were employed to compute the binding free energies of the docked complexes [60]. A total of 450 frames each after 0.2 ns were extracted from the complete MD trajectory and analyzed using the MM-PBSA computation via the MMPBSA.py module [61] of AMBER18. To determine the binding free energies (△G_bind_) values, the following equation was used:


$${\Delta G}_{bind}\;=\;{\Delta G}_{complex}-\;[{\Delta G}_{receptor}\;+\;{\Delta G}_{ligand}\;$$ (1)

ΔG_complex_ represents the total free energy of the protein-ligand complex, while ΔG_receptor_ and ΔG_ligand_ indicate the total free energies of the separated protein and ligand in solvent, respectively.


$$\Delta G\;=\;E_{gas}\;+\;{\Delta G}_{solv}-{TS}_{solute}\;$$ (2)

T denotes the temperature, while S signifies the entropy contribution to ligand binding, determined by established approximations. The gas phase energy (E_gas_) is commonly derived from the force field’s MM. It encompasses contributions from internal energy, electrostatic interactions, and van der Waals interaction energies as follows:


$$E_{gas}\;=\;E_{int}\;+{\;E}_{ele}\;+\;{E\;}_{vdw}\;$$ (3)

The ΔG_solv_ term is computed via an implicit solvent model and divided into electrostatic and non-polar components.


$${\Delta G}_{solv}\;=\;{\Delta G}_{ele}\;+\;{\Delta G\;}_{np}\;$$ (4)

### Ethics statement

The ethical committee of Semnan University of Medical Sciences approved this study with the number: IR.SEMUMS.REC.1403.248.

## Results

### Identification of differentially expressed genes (DEGs)

**Fig 1:**
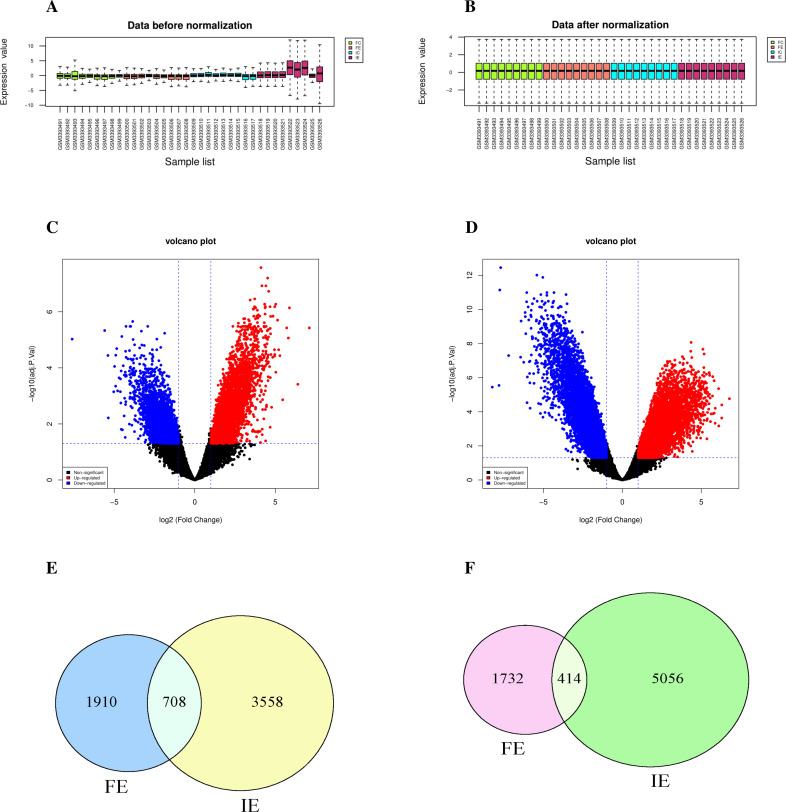
DEGs analysis for the GSE120103 dataset. **(A)** Box plot of the expression data before and (B after normalization. **(C)** Volcano plot of DEG between FE and FC groups. **(D)** Volcano plot of DEG between IE and IC groups. **(E)** The Venn diagram of the overlapping up-regulated DEGs in FE and IE. **(F)** The Venn diagram of the overlapping down-regulated DEGs in FE and IE.

All four groups of the GSE120103 dataset underwent normalization, and a box plot was generated to illustrate the data distribution before and after to normalization (Fig 1A, B). A total of 4764 DEGs were identified between the FE and FC groups, with 2618 up-regulated and 2146 down-regulated. On the other hand, 9736 DEGs were found between the IE and IC groups, comprising 4266 up-regulated and 5470 down-regulated DEGs (Supplementary S1 Table). The DEGs between the FE and FC groups, as well as between the IE and IC groups, were depicted using a volcano plot (Fig 1C, D). According to the Venn diagram, 708 up-regulated and 414 down-regulated DEGs were found to be common in both FE and IE (Fig 1E, F).

### Functional and pathway enrichment analysis of DEGs

**Fig 2:**
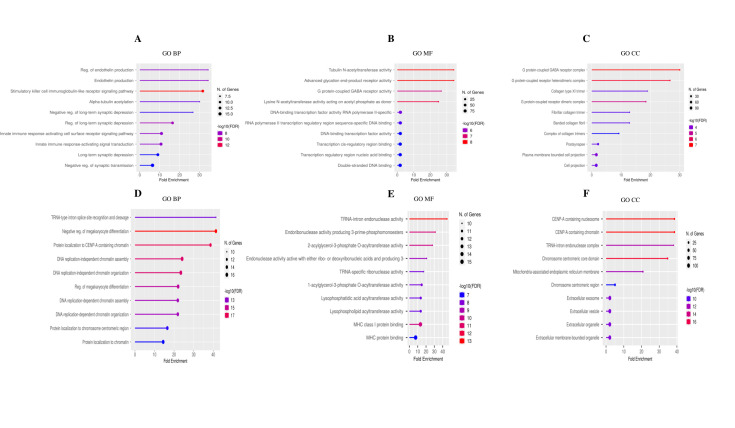
The lollipop charts of the 10 most significant functional enrichment analyses of GO for shared DEGs between the FE and IE groups. **(A)** BP corresponding to common up-regulated DEGs, **(B)** MF corresponding to common up-regulated DEGs, **(C)** CC corresponding to common up-regulated DEGs, **(D)** BP corresponding to common down-regulated DEGs, **(E)** MF corresponding to common down-regulated DEGs, **(F)** CC corresponding to common down-regulated DEGs.

The ShinyGO 0.81 tool provided 355 GO items for common up-regulated DEGs between the FE and IE groups, including 269 BP items (Supplementary S2 Table), 42 MF items (Supplementary S3 Table), and 44 CC items (Supplementary S4 Table). Similarly, 405 GO items for common down-regulated DEGs between the FE and IE groups were obtained, comprising 294 BP (Supplementary S5 Table), 44 MF (Supplementary S6 Table), and 67 CC (Supplementary S7 Table) items. Fig 2 illustrates the 10 most significant functional enrichment analyses of BP, CC, and MF catalogs for up- and down-regulated DEGs. The DAVID tool identified up-regulated DEGs in 18 KEGG enrichment pathways, with 10 being significant (P-value < 0.05) (Supplementary S8 Table). Down-regulated DEGs were associated in 31 KEGG enrichment pathways, 21 of which were significant (Supplementary S9 Table).

### PPI network analysis and hub gene identification

**Fig 3:**
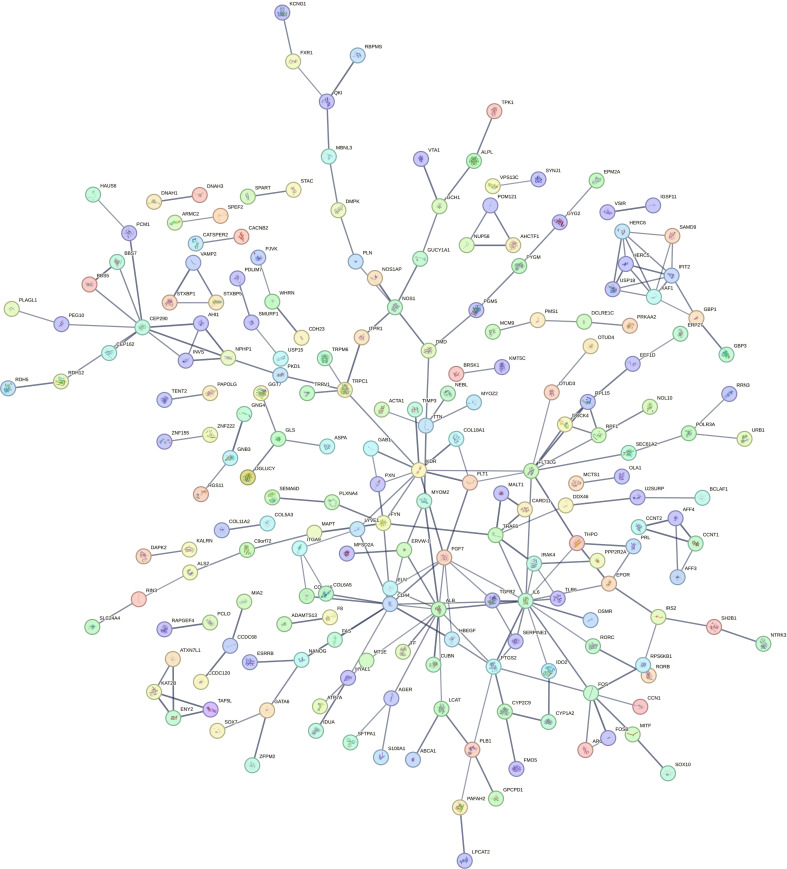
The PPI network comprising shared up-regulated DEGs in the FE and IE groups. The network comprises 614 nodes and 236 edges. Each node signifies a protein, while each edge denotes a protein-protein association.

**Fig 4:**
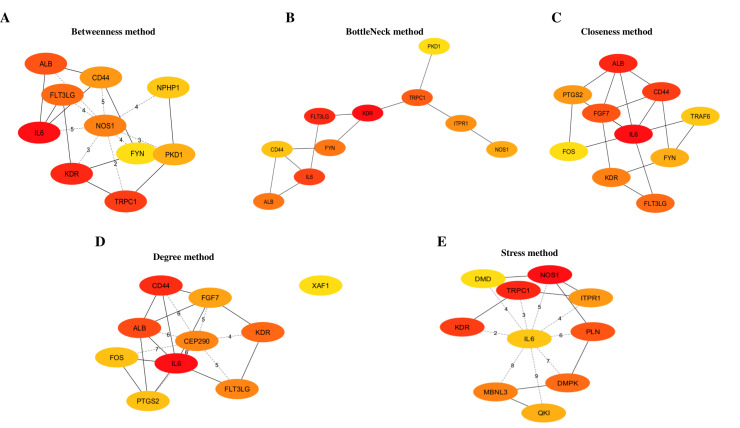
The top 10 genes identified using the five approaches given by the Cytoscape’s CytoHubba plugin(A) The top 10 genes identified using Betweenness method. **(B)** The top 10 genes identified using BottleNeck method. **(C)** The top 10 genes identified using Closeness method. **(D)** The top 10 genes identified using Degree method. **(E)** The top 10 genes identified using Stress method. The colors represent high (red) to low (yellow) scores.

**Fig 5:**
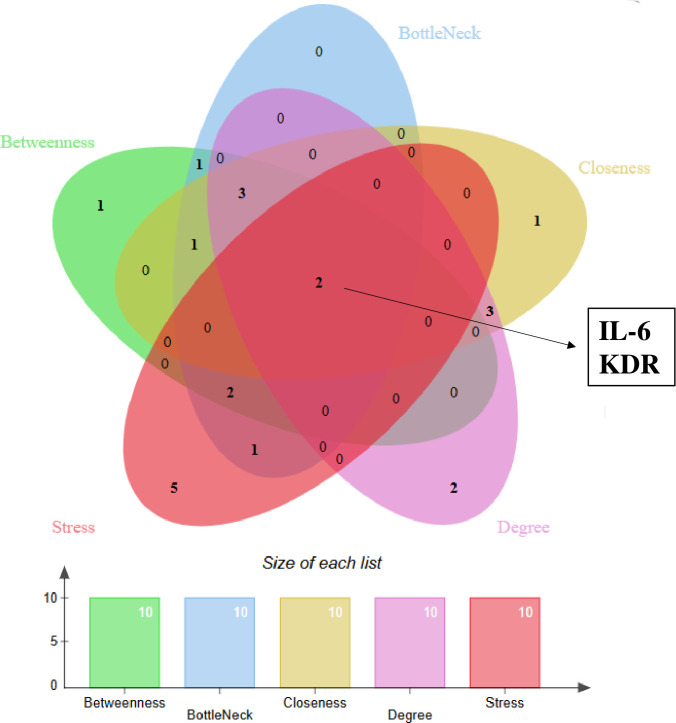
The Venn diagram reveals the presence of two common hub genes, IL-6 and KDR, resulting from the intersection of the top 10 genes identified using the five approaches given by the Cytoscape’s CytoHubba plugin.

The PPI network of shared up-regulated DEGs in the FE and IE groups, consisting of 614 nodes and 236 edges, was generated using STRING (Fig 3). To enhance clarity, we removed isolated genes that lacked interactions with other genes. Supplementary S10 Table and Fig 4 indicate the top 10 genes identified through the five methods provided by Cytoscape’s CytoHubba plugin. The Venn diagram indicated a presence of two common genes, namely IL-6 and Kinase Insert Domain Receptor (KDR), among the genes identified by all five approaches (Fig 5). Consequently, we selected these genes as potential hub gene candidates.

### Regulatory network analysis of hub genes

**Fig 6:**
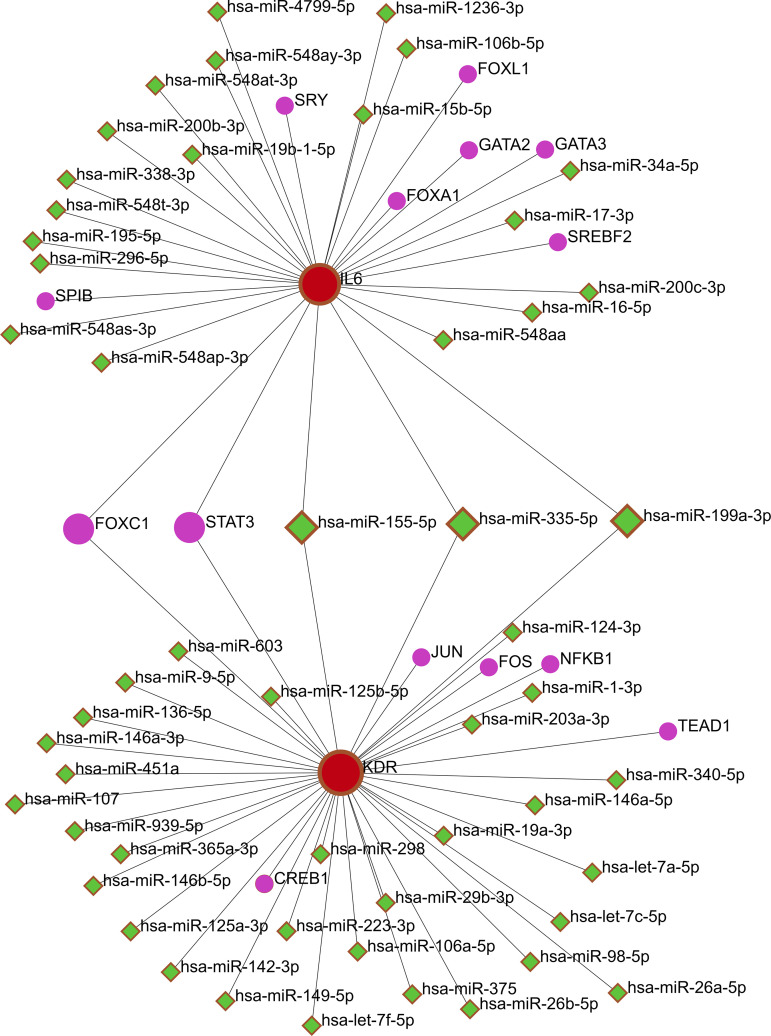
The miRNAs/TFs-hub genes network. The red circle, green diamond, and purple circle indicate hub genes, miRNAs, and TFs, respectively.

The miRNAs/TFs-hub genes network was constructed using the NetworkAnalyst tool. The miRNAs/TFs-IL6 network comprises 7 TFs and 33 miRNAs (Supplementary S11 Table), while the miRNAs/TFs-KDR network consists of 9 TFs and 22 miRNAs (Supplementary S12 Table). In the network, hsa-miR-155-5p, hsa-miR-335-5p, hsa-miR-199a-3p, FOXC1, and STAT3 interacted with both hub genes (Fig 6).

### Identification of druggable genes

The DGIdb 5.0 revealed that among the hub genes and their corresponding TFs, IL-6, KDR, and STAT3 had druggability potential (Table 1). We chose the KDR (kinase insert domain receptor) gene for further investigation because it belongs to the kinase family, is located on the outside of the plasma membrane, and exists in both druggable genome lists from Hopkins and Groom (2002) and Russ and Lampel (2005). Synonyms for the KDR gene include vascular endothelial growth factor receptor 2 (VEGFR2), VEGFR, fetal liver kinase 1 (FLK1), and CD309, with VEGFR2 utilized in this article henceforth.

**Table 1:** Results of the evaluation of the druggability potential of hub genes and their related TFs.

gene	category	sources
**IL6**	DRUG RESISTANCE	CIViC
	DRUGGABLE GENOME	HingoraniCasas
	GROWTH FACTOR	GO
**FOXC1**	TRANSCRIPTION FACTOR	Pharos
**STAT3**	CLINICALLY ACTIONABLE	CarisMolecularIntelligence, FoundationOneGenes, Oncomine, Tempus
	DRUGGABLE GENOME	HingoraniCasas
	KINASE	Pharos
	NUCLEAR HORMONE RECEPTOR	GO
	TRANSCRIPTION FACTOR COMPLEX	GO
	TUMOR SUPPRESSOR	GO
**KDR**	CLINICALLY ACTIONABLE	CarisMolecularIntelligence, FoundationOneGenes, MskImpact, Oncomine, Tempus
	DRUGGABLE GENOME	HingoraniCasas, HopkinsGroom, RussLampel
	EXTERNAL SIDE OF PLASMA MEMBRANE	GO
	KINASE	HopkinsGroom, Pharos, dGene
	TYROSINE KINASE	GO, dGene

### Retrieval and preparation of protein target structure and FDA-approved drugs

The PDB format structure of the human VEGFR2 protein (ID: 1YWN, resolution: 1.71 Å) in complex with 4-amino-furo[2,3-d] pyrimidine (also known as leukaemia inhibitory factor (LIF)) was downloaded from RCSB PDB, and the preparation process was conducted on it (Supplementary S13 File). A collection of 16 FDA-approved drugs in SDF format was obtained from the DrugBank database (Supplementary S14 File). This library included a list of drugs that possessed both an available structure and inhibitory characteristics (Table 2).

**Table 2:** A list of FDA-approved drugs targeting human VEGFR2, with their binding affinity values (kcal/mol) against human VEGFR2.

NO.	Drugbank ID	Chemical Formula	Drug	Binding Affinity (kcal/mol)
1	DB09079	C_31_H_33_N_5_O_4_	Nintedanib	−7.6
**2**	DB06589	C_21_H_23_N_7_O_2_S	Pazopanib	−8.9
**3**	DB08896	C_21_H_15_ClF_4_N_4_O_3_	Regorafenib	−9.1
**4**	DB14840	C_24_H_21_BrFN_5_O_2_	Ripretinib	−8.9
**5**	DB05294	C_22_H_24_BrFN_4_O_2_	Vandetanib	−7.8
**6**	DB06626	C_22_H_18_N_4_OS	Axitinib	−8.7
**7**	DB12010	C_23_H_26_FN_6_O_9_P	Fostamatinib	−8.8
**8**	DB11679	C_21_H_19_N_3_O_5_	Fruquintinib	−8.9
**9**	DB09078	C_21_H_19_ClN_4_O_4_	Lenvatinib	−8.1
**10**	DB06595	C_35_H_30_N_4_O_4_	Midostaurin	−8.4
**11**	DB11828	C_30_H_29_ClN_6_O_3_	Neratinib	−8.5
**12**	DB08901	C_29_H_27_F_3_N_6_O	Ponatinib	−9.6
**13**	DB15822	C_27_H_32_FN_9_O_2_	Pralsetinib	−9.1
**14**	DB00398	C_21_H_16_ClF_3_N_4_O_3_	Sorafenib	−8.9
**15**	DB01268	C_22_H_27_FN_4_O_2_	Sunitinib	−7.8
**16**	DB11800	C_22_H_19_ClN_4_O_5_	Tivozanib	−8.7
**17**	DB04727	C_27_H_19_F_4_N_5_O_3_	LIF	−9.2

### Molecular docking-based virtual screening

The molecular docking analysis, which used FDA-approved drugs as ligands targeting human VEGFR2, revealed valuable insights on molecular interactions. Molecular docking was carried out using a grid box including all of the binding residues, including Leu35, Gly36, Arg37, Gly38, Gly41, Val43, Ala61, Lys63, Glu80, Ile83, Leu84, Ile87, Val93, Val94, Val111, Glu112, Phe113, Cys114, Leu164, His171, Leu180, Ile189, Cys190, and Asp191. The results of molecular binding analysis revealed that 16 FDA-approved drugs had binding affinities ranging from −7.6 to −9.6 kcal/mol, with only one drug (ponatinib) having a greater binding affinity than the co-crystal ligand (LIF) and being chosen for further study. Table 2 presents the binding affinities of the FDA-approved drugs targeting human VEGFR2.

### Interaction analysis in docked complexes

**Fig 7:**
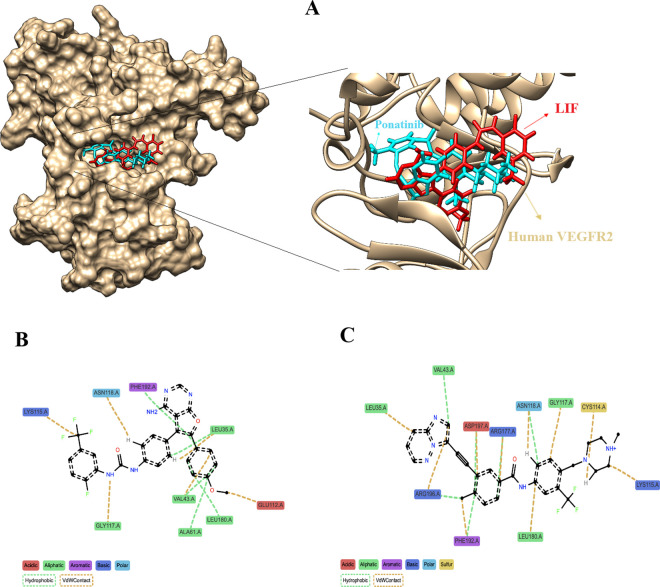
The binding conformation of LIF and ponatinib within the binding pocket of human VEGFR2. **(A)** 3D interactions of LIF (red) and ponatinib (blue) with human VEGFR2 (tan). **(B)** Interactions map of LIF with human VEGFR2. **(C)** Interactions map of ponatinib with human VEGFR2.

The binding orientation and interaction of ponatinib and LIF within the binding pocket of human VEGFR2 were predicted using the UCSF Chimera software and ProLIF package, respectively. Fig 7A illustrates that ponatinib overlaps with the LIF and occupies the active site. LIF established different interactions with human VEGFR2, including 6 van der Waals (vdW) contacts (leu35, Val43, Glu112, Lys115, Gly117, Asn118) and 5 hydrophobic interactions (Leu35, Val43, Ala61, Gly117, Leu180) (Fig 7B). Likewise, ponatinib formed many interactions with human VEGFR2 including 10 vdW contacts (Leu35, Cys114, Lys115, Gly117, Asn118, Arg177, Leu180, Phe192, Arg196, Asp197) and 6 hydrophobic interactions (Val43, Asn118, Arg177, Phe192, Arg196, Asp197) (Fig 7C).

### Molecular dynamics simulation

**Fig 8:**
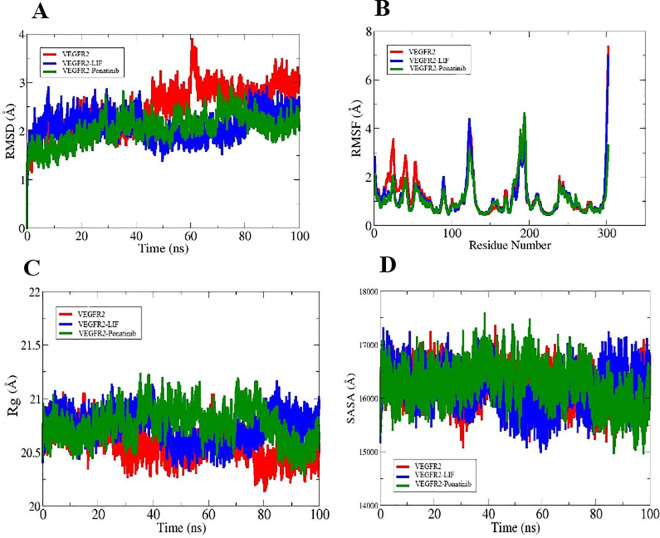
A graphical representation of the MD simulation results. **(A)** RMSD plot. **(B)** RMSF plot. **(C)** Rg plot. **(D)** SASA plot.

The RMSD is an fundamental parameter employed to evaluate structural deviations and the overall stability of proteins [62]. The RMSD plot of free VEGFR2 increased from 1.1 Å to 3.9 Å during the first 60 ns of the simulation, then fluctuated between 2.5 Å and 3.5 Å until the end. The RMSD plot of the VEGFR2-LIF complex indicates a minimum value of 0.9 Å and a maximum value of 2.9 Å, whereas the RMSD plot for the VEGFR2-ponatinib complex reveals a minimum value of 0 Å and a maximum value of 2.9 Å (Fig 8A). The fluctuations of residues during the simulation were evaluated using an RMSF analysis. The RMSF plot for the initial 75 residues of the free VEGFR2 exhibited greater fluctuations compared to the VEGFR2 complexed with LIF and ponatinib. Beyond this segment, with the exception of minor fluctuations, the RMSF plots s for VEGFR2, VEGFR2-LIF, and VEGFR2-ponatinib complexes overlapped throughout the simulation (Fig 8B). Rg denotes the folding rate and quantifies the protein’s compactness during simulation [63]; typically, a protein with a greater Rg indicates a less densely packed structure [64]. The maximal Rg values for VEGFR2, VEGFR2-LIF, and VEGFR2-ponatinib complexes were 21.05 Å, 21.2 Å, and 21.25 Å, respectively (Fig 8C). SASA is a measurement of a protein structure’s surface area that solvent molecules can access [65]. The SASA plots for VEGFR2, VEGFR2-LIF, and VEGFR2-ponatinib were nearly overlapping during the initial 23 ns. Within the range of 23–50 ns, the least SASA value was seen for VEGFR2, whereas the maximum SASA value was recorded for VEGFR2-ponatinib. During the final 20 ns of the simulation, the VEGFR2 plot fluctuated, whereas the VEGFR2-LIF and VEGFR2- ponatinib plots reached almost steady state (Fig 8D).

### MM-PBSA/GBSA analysis

The MM-GBSA and MM-PBSA methods were used to calculate the binding free energies of VEGFR2-LIF and VEGFR2-ponatinib complexes. The total MM-GBSA binding free energy was −82.51 ± 5.20 kcal/mol and −85.52 ± 5.06 for VEGFR2-LIF and VEGFR2-ponatinib complexes, respectively. The total MM-PBSA binding free energy was −82.38 ± 5.34 kcal/mol and −84.89 ± 5.65 kcal/mol for VEGFR2-LIF and VEGFR2-ponatinib complexes, respectively. The computed values indicate that the contributions of vdW energy are substantial in both methods. The contributions of each energy component are presented in Table 3.

**Table 3:** Binding free energy value for VEGFR2-LIF and VEGFR2-ponatinib complexes.

Method	Energy section	VEGFR2-LIF complex (kcal/mol) ± Standard Deviation	VEGFR2-ponatinib complex (kcal/mol) ± Standard Deviation
**MM-GBSA**	van der Waals energy	−75.69 ± 5.36	−78.52 ± 5.88
	Electrostatic energy	−24.36 ± 3.57	−25.87 ± 2.75
	Solvation energy (SE)	17.54 ± 2.56	18.87 ± 2.11
	Gas phase energy	−100.05 ± 4.85	−104.39 ± 6.47
	Total binding energy	−82.51 ± 5.20	−85.52 ± 5.06
**MM-PBSA**	van der Waals energy	−75.69 ± 5.36	−78.52 ± 5.88
	Electrostatic energy	−24.36 ± 3.57	−25.87 ± 2.75
	Salvation energy (SE)	17.67 ± 2.59	19.50 ± 2.88
	Gas phase energy	−100.05 ± 4.85	−104.39 ± 6.47
	Total binding energy	−82.38 ± 5.34	−84.89 ± 5.65

## Discussion

Endometriosis, a chronic and often painful systemic disorder, can negatively impact physical and mental health, quality of life, and productivity. This condition imposes substantial economic and social burdens on patients, their families, and society at large. Despite its prevalence and considerable costs, endometriosis receives minimal funding and research attention, significantly limiting our understanding of the disorder; therefore, urgent investigation into its diagnosis and treatment is necessary [66].

Bioinformatics analysis can find diagnostic markers, providing potential biomarkers for human disease diagnosis and improving our understanding of disease pathophysiology [67]. In this study, to identify effective diagnostic biomarkers for endometriosis patients, the GSE120103 dataset, which contains gene expression data of fertile and infertile women with endometriosis and their control groups, was downloaded from the GEO database and integrated analysis was performed. In this work, 708 common up-regulated DEGs and 414 common down-regulated DEGs were found between FE and IE.

GO enrichment analysis indicated that the most of the common up-regulated DEGs between FE and IE were mainly associated with four GO BP terms (i.e., regulation of response to stimulus, system development, cellular developmental process, and cell differentiation), six GO MF terms (i.e., cation binding, metal ion binding, nucleic acid binding, DNA binding, transcription regulator activity, and double-stranded DNA binding), and three GO CC terms (i.e., cell projection, plasma membrane bounded cell projection, and neuron projection).

The highest number of the common up-regulated DEGs between FE and IE were associated with four KEGG pathways including herpes simplex virus (HSV) 1 infection, mitogen-activated protein kinase (MAPK) signaling pathway, phosphatidylinositol 3-kinase-protein kinase B (PI3K/AKT) signaling pathway, and cytoskeleton in muscle cells. Cong *et al*. reported in their study that the herpes simplex virus (HSV) 1 infection pathway is implicated in endometriosis [68]. HSV is the primary agent responsible for infections in the oro-facial regions and genital tracts. Farsimadan *et al*. claimed that HSV is associated with infertility [69]. Consequently, HSV may cause female infertility by damaging the endometrium. Honda *et al*. demonstrated that both MAPK and PI3K/AKT pathways are constitutively active in ovarian endometriosis [70]. The MAPK pathway promotes the development and maintenance of ectopic endometrial tissues by influencing the actions of several cytokines, whereas the PI3K/AKT pathway enhances cell survival, proliferation, and migration [71]. To confirm the correlation of these pathways with endometriosis, the expression of each protein within these pathways can be evaluated in vitro in endometriosis by immunohistochemistry (IHC) and Western blot analysis. The expression levels of the genes that encode these proteins can be evaluated using real-time polymerase chain reaction (PCR). Cao et *al*. evaluated the dose-dependent inhibitory effect of ginsenoside Rg3 on ectopic endometrial development in treated mice vs the control group. Immunohistochemistry and Western blotting validated that the expression levels of VEGF, p-Akt, and p-mTOR were down-regulated in lesions subjected to ginsenoside Rg3 treatment. Real-time PCR results indicated that the mRNA expression levels of VEGF, Akt, and mTOR were decreased in ectopic endometrium [72].

Based on the PPI network of shared up-regulated DEGs in the FE and IE groups, we identified two potential hub genes: VEGFR2 and IL-6. VEGFs and their receptors (VEGFRs) are the most critical and particular factors that promote the proliferation of endothelial cells, regulate the development of blood vessels from precursor cells during early embryogenesis and the formation of blood vessels from preexisting vessels later on, and improve vascular endothelial cells’ chemotaxis and vascular permeability [73,74]. VEGFR2 is activated upon VEGF binding, initiating a phosphorylation process that enhances endothelial cell proliferation and migration [75,76]. The study by Cao et al. suggests that VEGF binding to VEGFR2 in vascular endothelial cells increases vascular permeability, enhances ectopic endometrial cell invasion and angiogenesis, promotes cell proliferation, and reduces cell apoptosis, ultimately leading to endometriosis [72]. VEGFA signaling via VEGFR2 is well known to promote angiogenesis and facilitate the growth of endometrial lesions [77–79]. Studies have indicated that VEGFR2 is significantly elevated in endometriotic tissues [77,80]. Bourlev *et al*. reported elevated expression of VEGFR2 in the blood vessels of eutopic endometrium from women with endometriosis compared to those without the condition [81].

IL-6 is a pro-inflammatory cytokine that is mostly produced by monocytes and macrophages, although it can also be secreted by T lymphocytes, B lymphocytes, hepatocytes, fibroblasts, keratinocytes, endothelial cells, mesangial cells, and other tumor cells [82]. Incognito *et al*. conducted a systematic review that included all studies published up to December 2022 that assessed IL-6 in serum, peritoneal fluid, follicular fluid, or endometrial biopsy samples, correlating their findings with endometriosis-related infertility. This study’s results indicated a correlation between elevated blood and peritoneal fluid IL-6 levels and the incidence of endometriosis-related infertility [83]. Increased concentrations of IL-6 in the peritoneal fluid of patients with endometriosis underscore its role in disease progression. According to studies, IL-6 activates macrophages, which can enhance endometrial cell proliferation [84]. Research demonstrates that IL-6, in conjunction with other cytokines, cultivates a peritoneal milieu favorable for the implantation and proliferation of endometriotic cells, underscoring its importance in endometriosis and various inflammatory and autoimmune disorders [85].

miRNAs are diminutive non-coding RNAs, averaging 22 nucleotides in length. The majority of miRNAs are transcribed from DNA sequences into primary miRNAs (pri-miRNAs), which are further processed into precursor miRNAs (pre-miRNAs) and mature miRNAs. In the majority of instances, miRNAs engage with the 3′ UTR of target mRNAs to inhibit expression [86]. miRNAs have been identified as possible biomarkers for the diagnosis of endometriosis. Several miRNAs associated with this condition have been published, including miR-451, the miR-200 family, miR-199, and miR-125 [87]. In this study, we predicted that three miRNA targets, hsa-miR-155-5p, hsa-miR-335-5p, and hsa-miR-199a-3p, regulate both hub genes. Daikoku *et al*. reported down-regulated expression of miR-199a-3p in the endometrium, which was associated with up-regulated expression of the cyclooxygenase-2 (Cox-2) gene, implying that low expression levels of miR199a-3p may correlate with the development of endometrial cancer [88].

Furthermore, both hub genes were associated with the TF targets FOXC1 and STAT3. FOXC1 is a major member of the FOX protein family, exhibiting abnormal expression in endometrial cancer and potentially influencing the migration and invasion of this malignancy; however, its mechanism of action is unknown [89]. In endometrial cancer, the downregulation of FOXC1 by miRNA, specifically miRNA 204 and miRNA 495, inhibits cancer cell proliferation and migration [90,91], suggesting that FOXC1 may possibly act as a potential oncogene in endometrial carcinoma [92]. FOXC1 stimulates VEGF expression via Notch signaling pathways [93]; the association of increased VEGF expression with endometrial cancer angiogenesis shows that FOXC1 is an important angiogenic and prognostic factor [94]. Chen *et al*. revealed that STAT3 is active in human endometrial and cervical cancers, and that inhibiting constitutive STAT3 signaling may serve as an effective target for intervention in these malignancies [95]. Kim *et al*. indicated that phosphorylated STAT3 is significantly expressed and activated in the endometrium of individuals with endometriosis [96].

We employed DGIdb 5.0 to identify potentially druggable genes among hub genes and TFs predicted to interact with drugs. Evaluation indicated that VEGFR2 may be a superior candidate for druggability compared to other hub gene and TFs. 16 FDA-approved drugs, along with the co-crystal ligand (LIF), were docked against VEGFR2. The docked complexes were assessed according to binding energy. Among 16 FDA-approved drugs, ponatinib had a greater negative energy than LIF. ProLIF analysis revealed that LIF is well packed into the VEGFR2 binding site. Binding affinity may be moderate and specificity may be lower due to the non-specific nature of vdW interactions. It was also shown that ponatinib not only fits tightly into the binding site, but also forms several strong interactions and binding specificity, resulting in higher affinity and better binding specificity than LIF. This analysis confirms that ponatinib binds more tightly to VEGFR2 than LIF based on the richness of interactions, diversity, and interaction of both non-polar and polar residues. The insights derived from this thorough interaction study illuminate potential mechanisms by which ponatinib may influence VEGFR2 function. This enhances its potential as a promising candidate for the treatment of endometriosis. Ponatinib, a powerful tyrosine kinase inhibitor (TKI), was initially developed to target the BCR-ABL1 oncoprotein. Several studies have shown that ponatinib blocks a wide range of additional oncogenic tyrosine kinases upstream of MAPK and PI3K [97,98]. MAPK signaling kinases are classified into three families: extracellular signal-regulated kinase (ERK), p38, and c-Jun N-terminal kinase (JNK) [99]. The study conducted by Ngô *et al*. demonstrates that endometriotic cells, in conjunction with endometrial cells from patients with endometriosis, activated the ERK pathway more significantly than endometrial cells from healthy individuals. This occurrence was associated with a heightened proliferation of endometriotic cells compared to endometrial cells. [100]. The activation of the PI3K/AKT/mTOR signaling pathway is significant throughout the transformation from normal endometrium to endometriosis, as revealed by the changed expression of essential pathway components in both eutopic and ectopic endometrium [101].

An RMSD value of <3Å is acceptable. The higher the RMSD value, the more deviation from the protein’s original conformation occurs [102]. The RMSD plot demonstrates that in the last 50 ns of the simulation, the RMSD of the VEGFR2 is larger than 3 Å, while the RMSD of the VEGFR2-LIF and VEGFR2-ponatinib complexes is less than 3 Å. The fluctuations in the VEGFR2-ponatinib complex are lower than in the VEGFR2-LIF complex. This observation could be ascribed to ponatinib’s numerous interactions with the active binding region of VEGFR2, which resulted in fewer conformational changes and a relatively stable pose. The RMSF plots of the VEGFR2-LIF and VEGFR2-ponatinib complexes exhibit lower residue fluctuations and lower RMSF values compared to the VEGFR2 residues. The results indicate that LIF and ponatinib can establish appropriate and stable interactions with the active site of VEGFR2 during the simulation. These results align with the findings from the RMSD results. The Rg plot revealed a lower value for free VEGFR2 compared to both VEGFR2-LIF and VEGFR2-ponatinib complexes, indicating a more compact VEGFR2 structure in the free state. The SASA plot indicated that the SASA values for both VEGFR2-LIF and VEGFR2-ponatinib complexes were close to those of free VEGFR2 for the majority of the simulation duration. Ponatinib exhibits a stronger binding affinity to VEGFR2 compared to LIF, as indicated by lower total binding energies in both MM-GBSA and MM-PBSA analyses. The energy contributions, especially from vdW and electrostatic interactions, support a more favorable and stable interaction profile for ponatinib.

This study enhances our comprehension of the molecular pathophysiology of endometriosis and subsequently offers new avenues for detection and targeting to improve therapy strategies. This computational work has identified a potential biomarker and an associated candidate drug through the analysis of extensive biological data; nonetheless, it is important to recognize the limits of this study. The sample size in our study is constrained, and we did not account for individual variations. A more reliable outcome could be attained by integrating additional data sets and samples in a subsequent experiment. This computational analysis cannot directly prescribe treatment for the condition. To authenticate our computational findings and further evaluate the therapeutic potential of the identified biomarker and corresponding candidate drug, we recommend for experimental validation via cellular assays and the establishment of animal models to assess their efficacy and safety in a more biologically pertinent context.

## Conclusion

The computational findings of this study suggest that VEGFR2 is a promising target for endometriosis treatment. This study assessed FDA-approved drugs for their ability to target the VEGFR2 protein in diseased situations. The virtual screening approach commenced with molecular docking of 16 FDA-approved pharmaceuticals, followed by an evaluation of binding affinity. Ponatinib exhibited superior binding affinity compared to the ligand within the structure. Moreover, interaction analysis demonstrated that both occupy the identical binding pocket and interact with essential amino acids. MD simulations lasting 100 ns were used to analyze the docked complexes. MD simulations validated the structural stability of the ponatinib-VEGFR2 complex. Based on these findings, ponatinib has been discovered as a viable endometriosis treatment. However, more experimental and clinical trials studies are needed to corroborate these findings and investigate the therapeutic potential of ponatinib for endometriosis.

## Supporting information

S1 Table The up- and down-regulated DEGs between groups in the GSE120103 dataset. (DOCX)

S2 Table The list of functional analysis of enriched GO BP terms of common up-regulated DEGs between the FE and IE groups. (DOCX)

S3 Table The list of functional analysis of enriched GO MF terms of common up-regulated DEGs between the FE and IE groups. (DOCX)

S4 Table The list of functional analysis of enriched GO CC terms of common up-regulated DEGs between the FE and IE groups. (DOCX)

S5 Table The list of functional analysis of enriched GO BP terms of common down-regulated DEGs between the FE and IE groups. (DOCX)

S6 Table The list of functional analysis of enriched GO MF terms of common down-regulated DEGs between the FE and IE groups. (DOCX)

S7 Table The list of functional analysis of enriched GO CC terms of common down-regulated DEGs between the FE and IE groups. (DOCX)

S8 Table The list of KEGG pathways involving common up-regulated DEGs between the FE and IE groups. (DOCX)

S9 Table The list of KEGG pathways involving common down-regulated DEGs between the FE and IE groups. (DOCX)

S10 Table The top 10 ranked hub genes by the Betweenness, BottleNeck, Closeness, Degree, and Stress algorithms in the CytoHubba plugin of Cytoscape. (DOCX)

S11 Table The list of nodes inside the miRNAs/TFs-IL-6 network. (DOCX)

S12 Table The list of nodes inside the miRNAs/TFs-KDR network. (DOCX)

S1 File This file is PDB format structure of the human VEGFR2 protein (ID: 1YWN) in complex with leukaemia inhibitory factor (LIF). (PDB).
(PDB)

S2 File This file is prepared structure of the human VEGFR2 protein. (PDB).
(PDB)

S3 File This file contains of 16 FDA-approved drugs in SDF format was obtained from the DrugBank database. (ZIP)
