# Supplementary material for: Using systems biology and drug repositioning approaches to discover FDA-approved drugs candidates for endometriosis treatment
Source: PLoS One. 2025 Sep 12;20(9):e0330841. doi: 10.1371/journal.pone.0330841 (PMC12431326; doi:10.1371/journal.pone.0330841)
Supplement: S1 Table — (DOCX) [file pone.0330841.s001.docx]

**Table S2**

The up- and down-regulated DEGs between groups in the GSE120103 dataset.

| **Up-regulated DEGs between FE and FC groups** | **Down-regulated DEGs between FE and FC groups** | **Up-regulated DEGs between IE and IC groups** | **Down-regulated DEGs between IE and IC groups** | **Common up-regulated DEGs between FE and IE groups** | **Common down-regulated DEGs between FE and IE groups** |
| --- | --- | --- | --- | --- | --- |
| BCLAF1 | CCSAP | ACTC1 | MLEC | BCLAF1 | CCSAP |
| PDXDC2P | KCNF1 | CSE1L-AS1 | HSP90AA1 | LOC100129397 | DOK7 |
| LOC100129397 | LRRN1 | MT1E | HMGB1 | FYN | SLC19A1 |
| FYN | DOK7 | IL19 | TES | KLF8 | KCNN4 |
| KLF8 | SLC19A1 | TMEM105 | ALCAM | LINC00312 | CCND1 |
| PRKAG2 | SORCS3 | ARSK | CACNA1I | ATP13A4 | ITIH5 |
| LINC00312 | BPIFB1 | ANKRD20A5P | C6orf106 | ANGPTL1 | MMP7 |
| ATP13A4 | CHRNA4 | TM6SF1 | LARS | ABI3BP | PNMAL1 |
| ANGPTL1 | TUBB2B | SAR1B | UBE2Z | COL4A6 | ELMSAN1 |
| SLC26A10 | KCNN4 | MARCH1 | G3BP1 | LOC202181 | HNF1A |
| KLHL9 | GH2 | ARHGEF10 | TIA1 | MCTS1 | CTBP1 |
| ABI3BP | LOC100996291 | CYP2D6 | UBXN4 | TNAP | MMP11 |
| RBM5 | CNGB1 | GHRL | TCF12 | GPR155 | FBN2 |
| LOC285097 | CCND1 | LOC100128288 | KDELR1 | MDH1B | MREG |
| COL4A6 | KIRREL2 | ZSWIM8 | PPME1 | KIF13A | FGFR3 |
| NRBP2 | ITIH5 | ZAP70 | STX6 | PRUNE2 | B4GALT2 |
| ZNF207 | TRABD2B | CCL3L3 | PTTG1IP | GABBR1 | SRRM3 |
| AFG3L1P | GPR3 | NADSYN1 | HIST1H4H | LCAT | WNT11 |
| CAPN3 | DRD5 | TUBB4A | NRP1 | KLF6 | TICRR |
| SLC23A3 | SPRR3 | LOC101927502 | BCLAF1 | SLC26A6 | SP100 |
| LOC202181 | FLJ38576 | HOPX | HSP90AB1 | ZNF25 | RAB8A |
| MCTS1 | MMP7 | FOXF1 | FAM98A | SND1-IT1 | MARCKSL1 |
| TNAP | PNMAL1 | NLRP1 | CDC40 | ERV3-2 | LAMC2 |
| GNRH1 | SBK2 | ITGAL | SMARCC1 | MYOZ2 | SNAI1 |
| PABPC1L | CYP4F2 | ADAMTSL4 | ZC3H11A | CCDC144B | BCL2A1 |
| BDP1 | ELMSAN1 | DENND1C | CLIC4 | MYO1B | ATP8B3 |
| LRRC75B | NPBWR2 | PIK3R5 | HIST1H4E | CCDC144A | SLC47A1 |
| GPR155 | HNF1A | CSAG1 | LAMA4 | SEC61A2 | RUNX1 |
| CRYGS | KRT24 | SPACA3 | NAPG | COL5A3 | HIP1 |
| OMD | PRSS56 | SLC22A11 | KDM5B | MEGF10 | MEX3D |
| RAPH1 | IL24 | SPDYE2 | RHOQ | OLA1 | CD276 |
| METTL3 | FUT8-AS1 | PITX1 | KCTD9 | GPCPD1 | LMNB2 |
| MDH1B | PRR3 | LOC101928370 | HIST1H3B | NTRK3 | LAPTM4B |
| DMTF1 | SPACA3 | HMX2 | GIGYF2 | PLXNA4 | PDIA4 |
| ACAD11 | TCTE3 | IRS2 | TOB2 | SHISA3 | ENTPD6 |
| FAM13A-AS1 | GFAP | CYP11A1 | LIG3 | TBC1D3B | DCAKD |
| LHFPL3 | C16orf78 | PPP2R2B | NBPF3 | ARHGEF10 | BIRC5 |
| GOLGA8A | GLIS2 | GPR135 | EFCAB14 | ZDHHC21 | ANKRD11 |
| CCNL2 | CTBP1 | CT45A5 | RCAN3 | SRD5A3-AS1 | TUBB3 |
| KIF13A | SIX3-AS1 | GABRG2 | FNBP1L | TRPC1 | BRF1 |
| MYO15A | SYNGR4 | GDPD5 | PTMA | SLC38A2 | KCTD1 |
| ATXN7 | GDF5 | CNTN2 | FLRT2 | ARL17B | LRTOMT |
| LINC01133 | SLC22A12 | GK | CRCP | ELN | NRP1 |
| NR4A2 | ASIC2 | FAM65C | MTRNR2L6 | LOC400756 | SAYSD1 |
| PHC3 | LOC643406 | CACNB1 | B3GALNT2 | AKNA | ANKRD65 |
| SMAD1 | C2CD4A | ICOS | ADAMTS7 | IDO2 | CTSC |
| PRUNE2 | KCNV2 | MARCH3 | TRIO | HESX1 | TMEM132A |
| ZMYM2 | DUX3 | ASGR2 | CCDC50 | TMEM198B | CACNG6 |
| LOC155060 | CDKN2A | SLC4A1 | AP3M2 | HELQ | SDK2 |
| CCDC18-AS1 | PAQR7 | ADGRG2 | ASPH | KAT2B | ZNF618 |
| SNORA70 | CCDC71L | LOC105376790 | ARL17B | CROCCP3 | TWIST1 |
| FUBP1 | FAM201A | IGHA2 | HIST1H3D | CDH23 | ZNF775 |
| CDC42-IT1 | DICER1-AS1 | SLC11A1 | DLGAP4 | MALAT1 | TRABD |
| GABBR1 | MMP11 | KIAA1107 | LSM12 | CEP44 | FANCI |
| TSPYL2 | KANK4 | SCRT1 | DNAJC8 | RUSC1-AS1 | TEF |
| PILRB | CYP4F30P | ZNF296 | COL1A2 | LOC643201 | PRSS12 |
| LOC101928524 | SFTPC | RGS11 | MYOF | ZSCAN30 | ADAMTS7 |
| LCAT | FBN2 | CARD11 | SERBP1 | ZNF439 | CARD10 |
| SLC6A16 | SLA | SYDE2 | ZBED3-AS1 | C1orf21 | MRAP2 |
| KLF6 | NAT8L | HSPG2 | DICER1 | AFF4 | MANEAL |
| ZFAS1 | MREG | PLCB2 | MZT2B | LOC401320 | CIB2 |
| VPS53 | FOXRED2 | PSORS1C2 | EID1 | RORC | AK1 |
| AHSA2 | MOP-1 | PAQR7 | PITPNB | MCM9 | RRP7A |
| ZNF765 | FGFR3 | VCY | TUBB | BCO2 | NUDT16L1 |
| GON4L | B4GALT2 | NOVA2 | TPM4 | SLC4A7 | STMN1 |
| LINC00894 | ACBD4 | FCGR2B | UBE2S | ASPA | SEMA3E |
| SLC26A6 | FAM124A | MYOZ3 | HMGN1 | C7 | ITPK1 |
| ZNF700 | C10orf82 | CLSTN3 | TTC3 | ZNF518A | RPS6KA1 |
| GDPD3 | GHRHR | PLXDC1 | HS2ST1 | C9orf131 | S100A5 |
| ADHFE1 | C10orf76 | TMEM86A | WIPI2 | SPEF2 | MEX3A |
| ZNF25 | EDN2 | LINC00671 | NBPF10 | GUSBP4 | MLXIP |
| SND1-IT1 | HPX-2 | FUNDC2 | ITPRIPL2 | CYP2C9 | SFRP1 |
| BUB3 | MAMSTR | RASL12 | MEX3D | KLHDC1 | DENND2A |
| RUFY3 | LOC100132147 | OR7E5P | SP3P | ARHGAP33 | MYCNOS |
| CARF | TGIF2 | GLYAT | RFX7 | DIO3OS | ACVR2B |
| ZNF37BP | NEUROD2 | TTC22 | KDM4A | ZNF155 | C19orf48 |
| PNPLA7 | STMND1 | PLAGL1 | SAFB | HBEGF | NLN |
| PROK1 | ERVV-2 | TRGV5 | WNT6 | PAPOLG | SYT6 |
| PLEKHH2 | JMJD1C-AS1 | ATXN1L | HIST1H3F | GK5 | PPP2R2C |
| APBB3 | ANKRD53 | ERVH-4 | LOC645513 | ASAP1-IT2 | CDT1 |
| RHOT1 | GFER | MAP4K1 | PSMD2 | MIR17HG | CCND2 |
| ERRFI1 | SRRM3 | MEF2D | GLG1 | ZFPM2 | LRRTM1 |
| DPY19L2 | MCEMP1 | CCDC57 | MED13 | RRN3 | SEC14L2 |
| ERV3-2 | LOC107161159 | LTBP3 | LBX1 | ABCA1 | CFAP206 |
| CIRBP | VPREB1 | LOC101927181 | HDGF | FAM153A | SOX4 |
| MYOZ2 | WNT11 | CDX2 | CCDC8 | LOC286382 | MARK2 |
| TACC1 | IL1B | BEST1 | PIP4K2A | MON2 | PPP6R1 |
| BTNL9 | RASGRF1 | LOC105370792 | GOLGA2P10 | LOC100505915 | TSPAN9 |
| DKFZP586B0319 | APBA2 | GRIN2C | ENC1 | ARHGEF28 | MTA1 |
| HERC2P2 | KCNQ2 | BCAT1 | UBE2V1 | H2AFJ | NEO1 |
| NDUFS4 | LCN15 | GFI1 | HIST1H4I | TDRD10 | CADM1 |
| CCDC144B | LOC101929122 | CD7 | ZMYND11 | ASAH1 | NACA |
| ACCS | TICRR | TMEM74B | CCSAP | L3MBTL1 | KCNQ1 |
| ARPC4-TTLL3 | ARHGAP39 | TRIP10 | RC3H1 | RGS11 | PIN1P1 |
| ZNF266 | ITGB2 | TMC8 | NOTCH2NL | RGS5 | SYT11 |
| MYO1B | NKAIN1 | SLC7A5 | HIST1H4C | HNF1B | AGR2 |
| EXOC3 | RIMBP2 | TRPV5 | CCND2 | EPOR | CHAT |
| CCDC144A | CASP14 | LOC389332 | RHOJ | LENG8 | B4GALT1 |
| LINC00461 | ADAMTS14 | IGHM | RALBP1 | HCG27 | FJX1 |
| DPY19L2P2 | TMPRSS6 | LINC00324 | FAM60A | TRAF3IP2-AS1 | BAX |
| LINC00667 | SLC32A1 | PRND | PTPN11 | ATAT1 | MIF-AS1 |
| RYR3 | AJAP1 | TMEM89 | SF3B2 | ACSS1 | TMSB15A |
| SCAF11 | SOX8 | RHOB | TMEM158 | ZSWIM8 | RITA1 |
| TPM1 | SP100 | SPACA1 | ZBTB38 | CAPRIN2 | GPSM1 |
| CMAHP | IL3RA | RUNX2 | KIF3B | XAF1 | CDKN2C |
| SEC61A2 | TMEM191B | ACAN | PUM2 | ZNF577 | LTBP3 |
| C1orf145 | IL17F | CHRD | ITGB1 | KIAA0040 | H2AFX |
| COL5A3 | ICAM5 | FSCN1 | COL3A1 | ANKRD20A2 | AIF1L |
| PARP6 | FGD2 | PRL | NFIA | MGA | KRT17 |
| PLEKHM3 | RAB8A | SHB | NBPF19 | KBTBD3 | STMN3 |
| CDC14A | NAGS | LAYN | ARCN1 | PEAR1 | PTK7 |
| GSDMB | ZDHHC8 | LOC105379239 | EIF2AK2 | AHI1 | KMT2A |
| LOC100132356 | CYP2W1 | CRTAM | PRKAB2 | GRIP2 | FDXR |
| KMT2C | SHISA7 | FGD5 | MAN1A2 | ABCC9 | FBXL17 |
| MEGF10 | OR8J1 | LTB4R | REST | MIR22HG | OPA3 |
| OLA1 | NOC2LP2 | PLXNA4 | BBC3 | ZNF23 | RNPS1 |
| LOC100506124 | CYP2A13 | KALRN | WHAMM | UBE2Q2L | ESPL1 |
| HEMK1 | MYLK2 | MZT2B | PFDN1 | USP47 | CBY1 |
| GPCPD1 | MYADML | POM121L1P | TTC3P1 | ACOT11 | TBL1XR1 |
| NTRK3 | IGHD | EMP1 | UBE2O | LOC100233156 | MRPS6 |
| RPL23AP32 | MARCKSL1 | KIR2DL4 | ZNF492 | CATSPER2 | FNDC3B |
| PLXNA4 | LAMC2 | PFKFB3 | DDX6 | GGT7 | MCM2 |
| SLC25A27 | B3GALT2 | NFKB2 | HERC2 | DYNC2H1 | FOXD2 |
| IGFBP5 | PFKFB3 | PTX3 | XIAP | LOC100505984 | NREP |
| SHISA3 | TMEM114 | LINC01013 | ZNF253 | KCNIP2 | IFITM5 |
| TBC1D3B | TFF3 | C16orf78 | EXOC3 | DFNB59 | TRIM16L |
| LINC00672 | TFF1 | PTF1A | ZCCHC17 | RUFY2 | C10orf2 |
| ARHGEF10 | CRHR1 | MASP2 | HIST2H4B | FOSB | OTUD7A |
| MUM1L1 | CD1D | PLXNA3 | MTPN | SPATA9 | C14orf169 |
| ZDHHC21 | ZBP1 | JPH2 | LOC283788 | HECTD4 | KRT14 |
| SRD5A3-AS1 | CRCT1 | CLCN1 | IGFBP5 | TAPT1-AS1 | INF2 |
| ATF3 | MTUS2 | RBM38 | RPN1 | CCNT2 | HRK |
| PIWIL4 | NLGN3 | LIMD2 | SAP25 | SREK1IP1 | CTBP1-AS2 |
| TRPC1 | FGF4 | CES3 | PRR5 | ECM2 | LMNB1 |
| SLC38A2 | IGLL5 | C17orf99 | CASC4 | DNAJC27 | LRRC45 |
| ARL17B | SNAI1 | CELA2B | SERPINH1 | TAF5L | MAGED4B |
| ELN | WFDC3 | LINC00893 | SEPT7 | SEPT8 | SRSF9 |
| LOC400756 | AQP9 | SSC5D | SEL1L | LOC440934 | BBC3 |
| AKNA | BCL2A1 | SLC37A2 | KPNB1 | ELMOD3 | PAFAH1B3 |
| C19orf18 | CCDC120 | SPN | VPS18 | OXGR1 | SQSTM1 |
| IDO2 | ATP8B3 | C11orf72 | MDM4 | DOCK4 | TNFRSF21 |
| LOC105377450 | XIRP2 | ZIM2 | PHF6 | CCDC171 | ZBTB47 |
| SLC25A35 | OLFML2B | EBI3 | TXLNA | VCX2 | B3GNT9 |
| HESX1 | OLIG1 | KCNE5 | HIST2H2AC | HIPK1-AS1 | FOLH1 |
| LOC102723694 | CST6 | KCNV2 | ADD1 | KIDINS220 | RAD54L |
| MED13L | KCNG4 | MLLT1 | SFXN1 | CCDC57 | ST14 |
| CMYA5 | SSX5 | SCD | SNRK | PNISR | EXOSC5 |
| CALD1 | GRPR | RUVBL1 | ARL6IP1 | BICC1 | FADD |
| TMEM198B | SPOCK2 | ZNF548 | ALDH3A2 | ZNF404 | SDC1 |
| HELQ | NKD2 | MAMSTR | LPGAT1 | INHA | RIBC2 |
| MEG3 | GABRG2 | PTGDR | HNRNPCL1 | MDM4 | FUT6 |
| PGGHG | SLC47A1 | GGT5 | CHRAC1 | GOLGA8F | MFSD9 |
| RORA | KCNK16 | CEACAM3 | STAU2 | MAPK10 | CLDN10 |
| FAM98B | CDH15 | LOC101927948 | MYH10 | ARHGAP30 | TSPAN10 |
| PDGFRA | OGDHL | CEBPB | OXR1 | SERPINE1 | DPEP3 |
| LOC100190986 | MGC16275 | USP31 | PABPC1 | MBNL3 | SHISA5 |
| KCNQ1OT1 | LOC100286906 | CYP21A2 | IGF1 | STAC | VAMP2 |
| KAT2B | RUNX1 | GS1-124K5.11 | ZMAT2 | LOC100509780 | MYH10 |
| SLF2 | LOC149373 | LOC102723809 | RPS2 | RDH5 | ACSS1 |
| CROCCP3 | SLC5A5 | GPX2 | STX2 | ADAMTS9 | PRR7 |
| MEDAG | MYOZ3 | ANKRD24 | YWHAZ | ZNF273 | GFRA2 |
| SCAPER | PLAUR | PLBD2 | RPS9 | ADCY10P1 | TMEM106C |
| CDH23 | MUC8 | SLC6A18 | FBXO9 | DUSP5P1 | WNT6 |
| MALAT1 | MBD3 | MYBPC3 | AXIN2 | UNC80 | RAX2 |
| ENO3 | TMEM63B | MYADML | SCLT1 | TRPV1 | GNB1 |
| RAB3GAP1 | PIP5K1C | DLX3 | RABL6 | CACNB2 | LEF1 |
| HSD17B3 | HIP1 | MLLT6 | ANKIB1 | DENND4A | HEXIM2 |
| ULK2 | KCNH3 | POM121 | DNAJC3 | MT1E | EN2 |
| CEP44 | CXCL8 | UPK3A | PSD3 | STRN | C1orf109 |
| SCARA5 | STEAP3 | SEC24A | CNN2 | ANKMY1 | E2F2 |
| RUSC1-AS1 | KISS1R | LOC101060524 | ZBED1 | PAPPA | RAB3B |
| LOC643201 | C1orf158 | PIP5K1A | FAM35BP | NXPH1 | PRKAR1A |
| ZNF83 | ATP1A2 | LOC149373 | ANP32E | SPIN3 | AUNIP |
| ZSCAN30 | ALAS2 | KIR2DS4 | RRBP1 | VPS13C | NANOS3 |
| PDK4 | AMIGO3 | NLRC4 | PARN | EEF1D | BCL2L13 |
| ZEB2 | IRS2 | C10orf91 | CCDC125 | FSIP2 | FARP1 |
| ZNF439 | GJB3 | GPR182 | PSMA7 | SH2B1 | CPXM1 |
| C1orf21 | SERPINA6 | SEPT12 | TLN2 | SEPT7P2 | TRIB2 |
| AFF4 | ST8SIA2 | TMEM255B | FBXO11 | SNRK | RCC1 |
| LOC401320 | LOC100129115 | RGPD1 | LARP4 | ZNF248 | DPYSL4 |
| RORC | GCM1 | CD300A | GPATCH11 | DMPK | CCDC86 |
| SNX29 | HOPX | RGS1 | FAM169A | LNX2 | CDC25A |
| MCM9 | LINC01146 | THEMIS2 | MIA3 | LOC728613 | SLC1A4 |
| BCO2 | KIAA0101 | ERVH-3 | CENPT | ZNF177 | TK1 |
| INTU | LOC101929680 | CCSAP | SF3B1 | PON3 | POLD3 |
| PIWIL2 | ANGPT4 | POLR3A | NBPF9 | GOLGA2P7 | SMAP1 |
| SLC4A7 | PPP1R1C | NFE4 | ATF7IP | PHYKPL | ALYREF |
| CLHC1 | VWA5B2 | APOA4 | TMTC3 | ADAM33 | HIST1H4G |
| HNRNPDL | ABHD16B | FLT1 | HK2 | ZNF507 | ACOT11 |
| ZEB1 | MMP17 | GPR37L1 | MBNL3 | APC | PRKCZ |
| ASPA | MEX3D | CAMK2G | NOSIP | CEP85L | ZMIZ2 |
| CCT6P1 | C21orf58 | XCR1 | NCOR1 | MMRN1 | ANKRD36B |
| CCL8 | GPR156 | CCDC71L | ANKRD13A | NAV2 | RREB1 |
| C7 | MYBL2 | OCM2 | SUPT16H | ZNF222 | CRYL1 |
| RBM19 | FAM214B | H2AFB2 | MTDH | CCDC30 | THOC6 |
| ZNF518A | LAT2 | CD3E | ZWILCH | ICA1L | HGS |
| C9orf131 | DPP10-AS1 | LINC00216 | USP33 | FOS | FOXL2 |
| SPEF2 | GABRA3 | PKD1 | PAPOLA | LMF1 | C2orf70 |
| ZMYND8 | CD276 | UBFD1 | SMAD5 | ANKRD20A5P | TOR4A |
| LOC100505771 | PIAS4 | FOXK1 | MDM1 | D2HGDH | SLC38A10 |
| SLC40A1 | CHST8 | GGT2 | MRAS | LOC100129406 | PDXK |
| AFDN | ADGRA1 | TANC2 | RCC2 | U2SURP | PFDN2 |
| LINC01140 | LMNB2 | MYOG | HOOK3 | KALRN | CCDC28B |
| GUSBP4 | NRSN2 | OR7E62P | PCNX4 | BCL2L11 | C20orf85 |
| DIP2B | PCDHB2 | PMFBP1 | RPL18 | DZIP3 | PACSIN1 |
| CYP2C9 | HAGHL | WRNIP1 | CDC42EP5 | ADAMTSL4 | H6PD |
| KLHDC1 | FAM84A | SLIT3 | RBM22 | ZNF675 | HIST2H3A |
| HBS1L | VWA5A | IL1F10 | SKI | UBR4 | CLN3 |
| ABCA6 | LAPTM4B | P2RX3 | MYL6 | STAG3L3 | MAN2B1 |
| ARHGAP33 | MOBP | PPP1R1C | TCEA1 | LOC202025 | SLC34A2 |
| ZNF514 | PDIA4 | MUC8 | AP3D1 | MYO15B | RCAN3 |
| KLF4 | DGAT2 | LYPD6B | ZNF124 | GPATCH2L | CFI |
| DIO3OS | XRCC3 | CCDC113 | ZBTB8A | GLIDR | MBP |
| ZNF155 | ENTPD6 | LOC105373876 | SYNCRIP | SNORD22 | LBH |
| HERC2P7 | DCAKD | TINAGL1 | ENAH | IRS2 | DBN1 |
| LOC283788 | NKX1-2 | MBOAT7 | DDX24 | SLC4A3 | SLC2A10 |
| HBEGF | CROCC | ARHGAP33 | SUZ12 | VTA1 | AGPS |
| B2M | ERN1 | PAX5 | RAD23A | COLQ | NEK2 |
| INTS6-AS1 | PRAP1 | SPATA25 | TMED4 | GUSBP3 | FGD1 |
| KCNJ8 | C16orf89 | SENCR | POP1 | DAAM1 | CKAP2 |
| SLC37A3 | LOC100131170 | CDA | PDZD8 | DMXL1 | SLC39A9 |
| BRINP1 | TPRX1 | FAM153A | KCNE4 | GNB3 | SH3D21 |
| LOC100507387 | GRIN1 | TBX19 | HIPK1 | LINC01410 | SIPA1L1 |
| PAPOLG | CPNE7 | PAX4 | DDX17 | PAFAH2 | PKMYT1 |
| OGG1 | LINC00176 | MAGEB1 | REEP3 | HSD17B6 | TGFBI |
| GK5 | GFRA4 | BDH2 | ZNF625 | PRKAA2 | CDCA8 |
| GOLGA6L9 | SPATA31E1 | ZNF595 | APPL1 | ZNF585A | SMIM6 |
| ASAP1-IT2 | CARD11 | OLFM2 | GPR135 | CXorf36 | LOC105370792 |
| DET1 | P2RX2 | APLP1 | CEP97 | FAS | KRBA1 |
| PER1 | LINC00167 | TRIOBP | YWHAG | FGF7 | RRM2 |
| MIR17HG | TBX22 | FARP2 | RB1 | CCDC85A | SIN3A |
| ZFPM2 | DPP10 | IL3RA | SOX4 | CENPT | FBRS |
| RRN3 | KRT16P3 | LINC01554 | ARIH1 | C5orf56 | WDR13 |
| RICTOR | MAST1 | THAP7-AS1 | MARCKSL1 | TRDMT1 | CANX |
| MARCH7 | FAM187A | CBX4 | GOLGA2P7 | FAM78B | IER5 |
| ABCA1 | AATK | SIRPB2 | DOCK7 | TAS2R43 | EMID1 |
| FLJ40712 | LCN1 | TTYH2 | MYO5A | HERC5 | MBOAT7 |
| C6orf141 | CHST13 | KRTAP1-1 | NCL | UBQLNL | FEN1 |
| DDX60L | IL37 | CYP3A43 | VIM | GEM | ATRX |
| ZNF540 | MSRA | PPM1N | TUBA1C | SPAG9 | LRRC20 |
| POU5F1 | BHLHA15 | MGC16025 | TLE1 | CDKL3 | ZDHHC13 |
| FAM153A | DNAJC5B | LNPK | FAM172A | TYW5 | CCDC65 |
| MYEF2 | BIRC5 | NPAP1 | HIST1H2AG | DCLRE1C | C19orf25 |
| ENGASE | VSTM2B | IL21R | COX15 | FMNL1 | MCM3 |
| LINC00342 | ANKRD11 | NXNL1 | TYW3 | FAM153B | FADS2 |
| CCNL1 | SERPINB2 | CXCL2 | G3BP2 | TTC26 | CHID1 |
| GOLGA2P10 | CT47A11 | SOX7 | FANCI | DUSP8 | FAM155A |
| AP5M1 | SRCIN1 | CCER2 | ANKRD11 | AGER | MON1B |
| GARNL3 | KLK13 | FAM214B | YBX1 | LOC440434 | TES |
| UBE2D3 | TUBB3 | LETM2 | MCRIP1 | SLC22A3 | GPI |
| TAS2R45 | FAM47A | LINC00469 | OAZ2 | ROR1 | HIST1H3F |
| AAK1 | OR10H2 | CD27 | PPM1K | LINC01061 | CDCA5 |
| LOC286382 | CDH4 | ZNF703 | ZDHHC21 | VCX3A | PLA1A |
| LOC650226 | ADAMTSL5 | MMP25 | EEF1D | RCSD1 | EYA2 |
| LINC01089 | KIFC3 | IGLJ3 | DDX42 | CSGALNACT2 | SLC31A1 |
| CFAP69 | C20orf144 | TNFAIP8L3 | GTF2A1 | KATNBL1 | CHADL |
| ANKRD20A11P | MAP2K7 | BBC3 | TPM3 | SPACA6 | HBA2 |
| MON2 | BRF1 | FOXI1 | NCAPH2 | TRPM6 | NUP93 |
| RBM20 | PLEKHF1 | RHPN1 | ZDHHC17 | CYP4Z1 | PFN1P2 |
| LOC100505915 | LIN28B-AS1 | ADGRG3 | PI4K2A | ZNF440 | SERPINH1 |
| MBD6 | WFDC5 | H6PD | EIF4A1 | CRISPLD2 | SDF2L1 |
| KIAA0754 | PLPPR2 | ETS1 | RCC1 | CYR61 | FBLIM1 |
| MTHFSD | ABCC6 | BACH2 | OXSR1 | FLJ30901 | MYC |
| ARHGEF28 | CLEC4M | WDFY3-AS2 | PAWR | RPF1 | FBXO17 |
| H2AFJ | SHD | DNAJC25 | HOXA10 | KIF27 | CDC45 |
| SUZ12 | IGHM | MAP6D1 | DST | WDFY3-AS2 | CCDC113 |
| TDRD10 | CNTN2 | NPIPA1 | CLIC5 | LYVE1 | QPRT |
| TTC21A | SSX2 | SMAD6 | PDIA3 | SYCP2 | SOX3 |
| LUC7L3 | LOC439933 | NUAK2 | GJC1 | DICER1-AS1 | CSNK1G2 |
| PARP12 | SMKR1 | CSHL1 | UTP14A | PDLIM7 | TSPAN14 |
| ASAH1 | MUC5B | OSGIN2 | DNAJC22 | ZNF169 | TNXB |
| L3MBTL1 | GPR31 | GPR45 | PRRC1 | NUDT13 | HJURP |
| MICAL3 | KCTD1 | C9orf47 | M6PR | SERPINA4 | RAB38 |
| ARSD | DMBT1 | IL10 | ZXDC | ZNF680 | FBRSL1 |
| CRIPAK | CRYM | PPP2R2D | NDUFA11 | AFF3 | RBBP4 |
| MT1H | COL20A1 | FCMR | PRKCI | THPO | MEST |
| LINC00260 | BPIFB6 | LINC01126 | ERC1 | RIN3 | EVA1B |
| VMP1 | CTRB2 | ANGPTL6 | CREBBP | TTC37 | KHSRP |
| GATA2-AS1 | CHGB | PRODH | MRE11A | CD55 | C9orf24 |
| LINS1 | JPH3 | IL37 | DIAPH3 | COL11A2 | CDH24 |
| LOC100289230 | LRTOMT | FAS | ISOC2 | KIAA1107 | EBPL |
| TBC1D3P2 | NRP1 | BCL2L11 | ATRX | EGFL8 | B9D1 |
| WDFY2 | ADAT3 | IGLL5 | PLCB1 | GALNT15 | WNT4 |
| ZFP36 | CACNG7 | CHRNA4 | ERP29 | TIMP3 | ARPIN |
| ERAP2 | SAYSD1 | NXF5 | MRPL30 | STAC2 | SERPINB8 |
| RGS11 | CCDC115 | PKLR | DNAH11 | STXBP5 | PBLD |
| RGS5 | ZNF71 | GABRR3 | TMX4 | PCM1 | SLC27A5 |
| DMXL2 | LILRA1 | C20orf197 | CNOT6L | LINC00152 | LOC101928068 |
| HNF1B | ANKRD65 | PPP1R1A | RYBP | LGALS8 | DFFA |
| MCM3AP-AS1 | NTSR2 | HK3 | CDK11B | PXN | RPN1 |
| EPOR | TBXAS1 | EPN3 | WHSC1L1 | POLR3A | RPUSD2 |
| ZNF563 | ZNF843 | PARP10 | KIAA0319L | GAB1 | FGF13 |
| U2AF1 | GS1-24F4.2 | FIZ1 | PRMT7 | LSMEM1 | COX6A2 |
| NTN5 | RARA | IL12RB1 | AGO3 | CARD11 | ELFN1 |
| CMTM7 | SCGB2B2 | LRRC28 | ASCC3 | DKFZP434I0714 | ALKBH2 |
| LENG8 | CACNA1E | UCN3 | ST13 | PSORS1C1 | C19orf73 |
| CYP2E1 | SLC36A3 | ADARB2 | WDR82 | LINC01138 | SMO |
| TCTN1 | APOBEC2 | CCIN | GRIN2D | MTMR9LP | CDS2 |
| HCG27 | HPDL | SLC38A3 | LMAN2 | FAM210A | C6orf47 |
| SNHG8 | CTXN1 | OR11A1 | HIST1H4L | UBAP1L | SNF8 |
| RGPD6 | CTSC | ESAM | PRR36 | GRIK2 | TCF3 |
| MT1X | MUC4 | SPRR2C | ACBD5 | CNOT4 | PPP1CA |
| MAP3K8 | SLC22A1 | UPK3B | SET | PLIN5 | RAB3D |
| LINC00844 | LCE3E | GORAB | PRICKLE1 | RMDN2 | RRP7BP |
| CENPJ | TMEM132A | FRMD1 | CTNND1 | ADAM20P1 | B3GALT5-AS1 |
| TRAF3IP2-AS1 | PSORS1C2 | GPR62 | HNRNPA3 | IRAK4 | EIF4E |
| ATAT1 | B4GALT7 | MYOZ2 | RPL19P12 | LRRK1 | LRP12 |
| SLC46A2 | LOC100130741 | XRCC3 | PPFIBP1 | MALT1 | PCSK5 |
| KIAA0226L | AQP5 | ITPK1 | COX6A2 | KRBOX4 | PDIA6 |
| ZNF692 | MOB3C | CYP4A11 | NDUFAF2 | SCAND2P | LAGE3 |
| ACSS1 | LINC00482 | PANX3 | PARD6G | FMO5 | SMARCD3 |
| PIK3C2G | CLCA3P | MRGPRE | FAM120A | GLS | NDUFB2-AS1 |
| ZNF331 | SMPDL3B | MUC22 | FTHL17 | ZNF595 | MFAP2 |
| ZSWIM8 | SP3 | KRT32 | SRP72 | GABARAPL3 | CNN3 |
| CAPRIN2 | THEG | NPHP1 | HIST1H2AJ | DLEU2 | WHSC1 |
| MBL1P | GPC6 | PRDX3 | MRPL34 | CLASRP | FDX1L |
| XAF1 | PVT1 | SMR3A | RPL18A | BRWD3 | PTGER4P2-CDK2AP2P2 |
| EFHC1 | SGK223 | CD300LB | ZNF626 | LMBRD2 | HN1 |
| BTAF1 | SYNPO | ADAT3 | FRYL | FAM46B | TUBB |
| SNHG17 | FAM43B | GLTPD2 | KIAA1109 | CD44 | ACOT7 |
| NEAT1 | WFDC12 | MYOM1 | CNTLN | YAE1D1 | SNRNP25 |
| ZNF577 | ETV1 | TEF | TOMM34 | PBLD | OVGP1 |
| KIAA0895L | CACNG6 | LOC101929680 | PPIB | FLCN | HOXB4 |
| NEK3 | DOC2B | KIF21B | PLIN3 | CEP104 | ZSCAN10 |
| LRRFIP1 | HYOU1 | BTBD16 | EIF4B | ATPAF1 | RFX1 |
| TAF15 | NKX6-3 | CD48 | ZDHHC20 | PLPPR2 | MECP2 |
| EPHA4 | BPIFA1 | A1CF | VCP | TERF1 | PYM1 |
| KIAA0040 | SDK2 | FAIM2 | FTSJ3 | LOC100128988 | HIST1H4J |
| EPG5 | IQSEC3 | BCO1 | LUC7L | MBD5 | TPM4 |
| ZGRF1 | GLYATL1 | CTF1 | IFITM5 | PGC | FLYWCH2 |
| ANKRD20A2 | CSE1L-AS1 | CLEC4M | HAUS6 | FBXL13 | PSIP1 |
| CDC25B | FPR2 | SLC14A1 | BBX | HAUS6 | SSU72 |
| FLNB | ZNF618 | SBNO2 | HIST1H4F | MAMDC2 | CNTNAP2 |
| CBX3P2 | OTX1 | KCNN2 | SLC6A10P | TRMT13 | FAM134C |
| SERPINB9 | PANK1 | SLC4A5 | CCDC137 | C9orf72 | GMCL1 |
| MGA | SYNDIG1 | MUC6 | FRG1BP | ZNF44 | PAMR1 |
| PRRT2 | LMNTD2 | C3orf33 | PPP1R14B | MPP3 | CDKAL1 |
| LOC100506388 | BCL11A | INHBC | ALDOA | CD99P1 | SLC2A4RG |
| PHF24 | CBARP | LINC00847 | HIST2H3D | ZSCAN26 | SART3 |
| KBTBD3 | SIX1 | LOC100134822 | HRK | SGK494 | TUBA1C |
| VPS13D | FAM104A | LGALS12 | SCAF11 | F8 | R3HDM2 |
| PEAR1 | TWIST1 | INO80D | ASPM | ZMYM5 | NCL |
| LOC286272 | RIMBP3 | ADAMTS7 | PIK3R3 | RBMS3 | ZNF467 |
| ZNF10 | DLG4 | C2orf72 | HIP1 | SPG20 | AGPAT2 |
| AHI1 | RASGRP1 | ACR | GOLGA8A | ZNF596 | BMP8B |
| RNF19A | MYEOV | CRY2 | GLIS3 | ZNF17 | FAM64A |
| SNX29P2 | ZNF775 | FCN1 | VANGL1 | ADAMTSL3 | CTDP1 |
| PCF11 | TRABD | CSF3R | ETV5 | USP18 | ZNF124 |
| GRIP2 | FAM179A | OR52K2 | AEBP2 | BOLA2-SMG1P6 | CACNB3 |
| ABCC9 | HCG25 | TDRD10 | TUBB2A | LOC146880 | SUMO2 |
| MIR22HG | SLC29A2 | ECRP | MAPK1 | ACADSB | PLIN3 |
| LINC00116 | NKAIN4 | GFRA3 | RPL6 | PLB1 | GRK3 |
| ZNF23 | DAB1 | TFPI | SSB | ADAMTS6 | FUBP1 |
| LINC00174 | ITIH2 | DNHD1 | TUBA4A | TMEM147-AS1 | TCOF1 |
| ANKRD49 | RUVBL1 | FAM25A | UBE2R2 | C10orf54 | BHLHE23 |
| CA3 | DTX3 | TP53AIP1 | CABIN1 | MLLT6 | UGDH |
| RPL32P3 | RASIP1 | SIM1 | HDLBP | OTUD4 | TMEM98 |
| C3orf35 | MMP1 | ARID3A | SLC35B2 | DNAH3 | GTPBP6 |
| UBE2Q2L | APC2 | RASD2 | ZFAND5 | SP3 | GIPR |
| CYP4B1 | ETNPPL | SIRPG | DDOST | SELO | CMTM6 |
| ERVK13-1 | OR5F1 | AFDN-AS1 | EMC9 | RORB | SLC22A18 |
| WDR27 | FCAMR | ARSF | RFC1 | MAGI2-AS3 | BTN2A3P |
| FRMD4A | SCRT1 | TMEM43 | PCM1 | ESRRB | MANF |
| MAP4K5 | CXorf57 | BCL2L1 | ZNF704 | TLR6 | WDR70 |
| CCDC180 | ST8SIA3 | RAVER1 | UFM1 | POM121 | RNF24 |
| MBNL1 | KRT85 | S1PR3 | LOC255308 | PLN | ZNF629 |
| USP47 | CELA2A | ZIC5 | KNL1 | GCH1 | LOX |
| ACOT11 | CACNA1A | SPDYE3 | FAM189A2 | TRAPPC11 | KAZALD1 |
| SMG1 | LOC101060157 | EDC4 | FGD5-AS1 | PROSER2-AS1 | DCBLD2 |
| SMG1P1 | F11 | AOC1 | ING5 | KCNB1 | SLC52A3 |
| MT1L | WWOX | CBX8 | SUMF2 | CCDC149 | KDELR2 |
| LOC100233156 | SYT9 | MGC16275 | HIST1H4B | NOS1AP | CFAP57 |
| C1orf186 | UMOD | SPDYE7P | BRCC3 | LOC101927178 | PRMT1 |
| CATSPER2 | LOC105370612 | LYNX1 | GMCL1 | PGM5-AS1 | GRAP |
| GGT7 | C2CD4C | SPNS3 | BRI3BP | ABCA5 | PTMA |
| NAALAD2 | PRB4 | GRIK3 | RBM27 | TAL1 | ATP2C1 |
| RPGR | LCE4A | GGTLC2 | RAB8A | CCDC68 | ADGRG1 |
| GRIA2 | FANCI | NAGS | SETD2 | STEAP2 | RRS1 |
| MAOA | HTR6 | LINC00334 | OS9 | CEP290 | NUTF2 |
| PRDM5 | CX3CL1 | NAP1L5 | KDM5A | ZNF506 | GUSB |
| ZNF471 | ALOX5AP | RARA | PA2G4 | TF | PLK1 |
| DYNC2H1 | PF4 | CCR2 | SPATA13 | ZNF665 | HNRNPL |
| LOC100505984 | FOXC2 | DNAH2 | LZTS2 | GLCCI1 | ZDHHC24 |
| LOC100289058 | PTPRN | ITIH6 | DIO2 | PYGM | PHPT1 |
| MIR600HG | TNFRSF9 | OR5H1 | KIAA1143 | SLIT3 | ARID3A |
| DOCK11 | TEF | ASCL1 | GAPVD1 | DAPK2 | SND1 |
| LOC158863 | COL4A3 | DDX46 | R3HDM4 | MAP3K13 | PSMD8 |
| CHD9 | NLE1 | ANKRD11 | YAP1 | NOTCH2NL | INAFM1 |
| LINC00674 | PRSS12 | MADCAM1 | NPAS3 | PRL | CCDC47 |
| KCNIP2 | BID | PROSER3 | KIF1B | LOC100506472 | MRPS34 |
| COX4I1 | UHRF1 | PDLIM7 | HIST1H2AK | NAA16 | EVX1 |
| LOC101928673 | LOC105373876 | UNC5A | FAM111B | SERHL2 | NUSAP1 |
| DFNB59 | FERD3L | SH3PXD2B | KIF27 | CAMK2G | BRI3BP |
| SPTLC3 | RIMS4 | PAX7 | LEF1 | HTATIP2 | MPZL1 |
| ZNF451 | NCF2 | MAGEC1 | OSR2 | SEMA6D | TUBA1A |
| RUFY2 | ADAMTS7 | GVINP1 | SDAD1 | MORN4 | HLA-E |
| FOSB | SIT1 | COL6A6 | FKBP14 | EPM2A | ARHGEF39 |
| ADD1 | ITGB6 | HCK | ITGB8 | DMD | DNAJC22 |
| BMS1P5 | KRT9 | COX6B2 | NR2F2 | ITPR1 | AFF1 |
| TNKS | KRT3 | BPTF | HUWE1 | SMTN | PPP1R16B |
| MAN2A2 | TRIM35 | TREM2 | GON4L | ACVR1C | TRIOBP |
| UGGT2 | SLC11A1 | GPR17 | RPP14 | C8orf37 | SPC24 |
| CPO | CARD10 | LHFPL4 | EGFL6 | ZBED9 | SOX17 |
| LOC101928291 | ZSCAN1 | KRT31 | WASF2 | PMS1 | CENPO |
| SPATA9 | BMP15 | SOGA1 | CBX1 | PAPD4 | PFAS |
| DDX17 | MYO1G | HTR1B | SMAD9 | S100A1 | ANGPTL2 |
| LOC114224 | IGLV1-44 | CTRL | MAGT1 | CEP162 | TSEN34 |
| SYTL4 | SYT3 | TGM7 | FMR1 | LRP1B | COA4 |
| SEPT7P9 | MRAP2 | TM6SF2 | SMG1 | VCX | GAMT |
| HECTD4 | TLDC1 | GAPT | ANKRD17 | APOL6 | AASDH |
| LRRC37A3 | MANEAL | RENBP | DAD1 | USB1 | ARHGEF18 |
| SLC16A6 | CIB2 | ZNF26 | MCM7 | FAM13C | FAT1 |
| ZMAT1 | SPRR1A | DCAKD | TXLNG | UBE2B | SFRP4 |
| TAPT1-AS1 | PTF1A | SLC36A3 | DCAF5 | ZFX | TMEM125 |
| LOC285074 | PCNX3 | LOC440792 | EIF3D | SYDE2 |  |
| CCNT2 | CASC3 | TONSL | DEPDC1 | ARL17A |  |
| NGFR | AK1 | RAPSN | METTL2B | IKZF5 |  |
| SREK1IP1 | TTTY13 | STX11 | LMO4 | DNHD1 |  |
| TEKT4P2 | VWA7 | LOC650293 | RPS2P32 | LACC1 |  |
| ECM2 | PCDHGA7 | RASGRP3 | RAB11FIP1 | ACKR4 |  |
| EPM2AIP1 | GSDMA | SLC14A2 | HSP90B1 | OSGIN2 |  |
| FAM160B2 | NXF2 | CPA5 | JAK1 | EWSR1 |  |
| NKTR | APLN | ESRRB | ZBTB43 | LOC55338 |  |
| ZNF493 | RRP7A | SCML4 | OSBP | ITGB1BP2 |  |
| DNAJC27 | NUDT16L1 | LNX2 | EDF1 | KIF16B |  |
| WT1-AS | ZC3HAV1L | USP6 | SLC5A3 | CCDC50 |  |
| RN7SL737P | SBK1 | IGHD | EIF2B3 | ATXN7L1 |  |
| RFX3 | IGLV6-57 | GPHA2 | RASSF8-AS1 | GPBAR1 |  |
| ENPP3 | STMN1 | TBATA | SLX4IP | IGSF11 |  |
| RBM8A | ADAMTS16 | LOC100129115 | CLEC2D | STON1-GTF2A1L |  |
| FAM189A2 | WFDC10B | DKFZp779M0652 | C11orf58 | FAM86B3P |  |
| TAF5L | MYCN | LCE4A | C9orf62 | LINC00950 |  |
| PRKD3 | LIMD2 | SYNGR3 | H2AFY | TNRC18 |  |
| ARMCX4 | LGALS9C | MYO1G | NFYC-AS1 | PGBD1 |  |
| P4HTM | ANP32C | SOCS1 | HNRNPA0 | RBPMS |  |
| GALNT10 | BEX1 | TMEM234 | NAA35 | TPCN1 |  |
| SLC25A29 | SEMA3E | LOC102724760 | BCAP31 | KCNG1 |  |
| FILIP1L | ITPK1 | RAPGEF3 | BHLHE23 | IDUA |  |
| SEPT8 | TUSC5 | LMX1A | AHNAK | NPAT |  |
| IL6ST | GNAT1 | CARMIL1 | BCL10 | WHRN |  |
| NHLRC3 | RPS6KA1 | SYT9 | MALAT1 | SPOCK1 |  |
| LOC730102 | TMEM26 | ANKRD53 | ARL13B | NPHP1 |  |
| ANKRD36BP2 | S100A5 | LCE3B | FTH1 | ZNF492 |  |
| ARID2 | MAGEB2 | OTUD7A | NUBP2 | SPRYD7 |  |
| LOC440934 | OXER1 | ARL13B | PGR | SLC38A11 |  |
| HDAC6 | SERPINB5 | SAA3P | RHOBTB1 | URB1 |  |
| MLIP | CIRBP-AS1 | HGC6.3 | RPL35 | ARC |  |
| FNDC3A | CEP55 | LOC100506571 | CYB561 | TGFB2 |  |
| SFXN4 | ROBO3 | PGF | PPP2R5A | ENY2 |  |
| CD302 | MEX3A | LOC100131170 | SLC25A36 | ARRDC3 |  |
| MT1G | KLHL30 | FAM71F1 | HNRNPA1L2 | DDX46 |  |
| RNF34 | URB2 | SMG7-AS1 | SCARNA17 | ACTA1 |  |
| ELMOD3 | RNFT2 | OXGR1 | MAP4 | LOC100996345 |  |
| ANAPC1 | WBSCR28 | CCDC7 | ZNF292 | TECTA |  |
| THUMPD3-AS1 | AZU1 | SPACA6 | VEGFA | SOGA1 |  |
| ABCC8 | MLXIP | LOC100507646 | NUTF2 | SLC24A4 |  |
| FST | SFRP1 | LILRA1 | ZNF776 | SHROOM1 |  |
| CLCN2 | GH1 | LINC01158 | TSPAN14 | RPS6KB1 |  |
| OXGR1 | VCX2 | TYW5 | BAALC | RASGRP3 |  |
| RAPGEF2 | DNAH2 | TAL1 | PLOD2 | LOC286254 |  |
| NPFF | ADAMTS13 | PTPN22 | ZNF467 | DDI2 |  |
| KIAA1109 | DENND2A | NFATC1 | FOXN2 | SOX12 |  |
| DOCK4 | TSHR | PI16 | FOXQ1 | RGS1 |  |
| FAM13A | H1FOO | FLJ35700 | GNB1 | QKI |  |
| CCDC171 | MROH5 | FLJ30901 | BMPR2 | RASGRF2 |  |
| PKD2 | LAT | WDTC1 | NIPSNAP1 | SH3BGRL2 |  |
| GREB1L | MYCNOS | CXCL3 | PSAP | FAM135A |  |
| VCX2 | WNT3A | GRPR | TRAF3IP1 | DMGDH |  |
| NR2C2 | HTR3A | MATN1 | PRDX5 | FAM124A |  |
| ZNF138 | TMEM151A | NR1D1 | RCN2 | RERG |  |
| HIPK1-AS1 | DBIL5P | GJB3 | MAP2K2 | MFSD8 |  |
| ITPKB | EDC4 | LOC728175 | TRAPPC6A | MAPT |  |
| KIDINS220 | ADGRG6 | FAM131A | FCGRT | LOC100132363 |  |
| PPM1A | CHCHD6 | MAZ | SPCS1 | ZMYM1 |  |
| PLEKHA5 | AFF3 | TMEM52 | CCDC86 | OTUD3 |  |
| CCDC57 | F13A1 | PRLHR | TMOD3 | OR5P1P |  |
| PNISR | ZFYVE26 | LOC102723335 | ATP13A3 | CYP1A2 |  |
| BICC1 | LENG9 | GGT3P | RPS19 | TTN |  |
| NAIP | UNKL | CRYGA | FKBP2 | SMA4 |  |
| ZNF404 | ACVR2B | SLC35F5 | PRR7 | PRELP |  |
| ANKRD30BP2 | PPP1R3F | CTXN1 | SCAF4 | CPB1 |  |
| RALGAPA1 | NKX6-2 | PSPN | STUB1 | SIK1 |  |
| RSU1 | ASCL2 | DHDDS | HIST1H2BE | ERC2-IT1 |  |
| MATN2 | OR11A1 | HAP1 | ETS2 | ODAM |  |
| INHA | SLC2A5 | CLEC10A | SOX3 | ZNF678 |  |
| MDM4 | SOX18 | UBQLNL | HEXIM2 | TICRR |  |
| GOLGA8F | PCDHGA2 | CHPF | RALGPS2 | RAPGEF4 |  |
| COG2 | BHLHE22 | NOS3 | DIDO1 | LINC01554 |  |
| COQ9 | HHLA1 | PCDHGA7 | TBX2 | ALS2 |  |
| RSRP1 | TRIM46 | LCN15 | SCRN2 | ALPK1 |  |
| SF1 | SLC25A15 | CD244 | EP300 | LINC01590 |  |
| MAPK10 | RHOF | S1PR2 | ATP6V1A | ZNF107 |  |
| RIC8B | TREM1 | CACNA1B | LRRC37A4P | MAPKBP1 |  |
| TIMP4 | AMBN | MAPK15 | DIABLO | SLC25A37 |  |
| PKD1L2 | ASPHD2 | LILRB3 | DPP7 | MYOM2 |  |
| ARHGAP30 | HID1 | SYCP1 | GOLGA8EP | CEP126 |  |
| RANBP17 | LINC01405 | PLA2G5 | IDH2 | FGD5 |  |
| ADAMTS18 | HGF | POM121L4P | H6PD | EHBP1 |  |
| ELK4 | TTC39A | CTSG | RPL4 | TMEM133 |  |
| USPL1 | CCKBR | CLASRP | GDNF | MCF2L |  |
| SERPINE1 | C19orf48 | KRR1 | SEPT10 | LRRC70 |  |
| MBNL3 | SOX9 | SLC35G2 | KAT6A | SEPSECS |  |
| TESMIN | RAPSN | DHX34 | KHDRBS1 | COG3 |  |
| STAC | PLCB2 | ZBTB34 | ATP1A1 | LINC00597 |  |
| SYNPO2 | NLN | KIRREL3 | CCDC171 | APLF |  |
| LOC100509780 | SYT6 | LOC100130920 | ARPC5 | VAMP2 |  |
| MFI2-AS1 | LRRC3 | PRSS3 | ATP5I | FLT1 |  |
| NFAT5 | PPY | LIN7A | C4orf3 | ALB |  |
| RDH5 | IL21R | PTCRA | EDNRA | SAMD9 |  |
| DENND2C | APOBEC3A | ORAI2 | PCMTD1 | PTPRR |  |
| TOM1L2 | PPP2R2C | NR4A3 | HIST1H2AH | CCNT1 |  |
| ZNF280D | IL1RN | THAP2 | RAB7A | TMUB2 |  |
| SAPCD1 | CDT1 | GNG13 | ARL8B | PCDH20 |  |
| SF3B1 | SPEF1 | OR7E91P | ZCCHC7 | HERC6 |  |
| ADAMTS9 | CFAP61 | OPN1MW | RABGAP1L | STARD9 |  |
| ANKRA2 | HBM | RUNDC3A | PDCD7 | GRAMD1C |  |
| JADE1 | CCND2 | COMP | CAMKK2 | SERTAD2 |  |
| PILRA | TMEM164 | HPX-2 | KPNA3 | STOM |  |
| PHKA2 | SLC4A1 | FLT4 | TMEM98 | FAM41C |  |
| PDE4D | CDC42BPG | LY9 | SSFA2 | ERICH6 |  |
| ZNF273 | LOC653581 | OSMR | EIF2S3 | LARP4 |  |
| XPNPEP3 | LOC399851 | GSN-AS1 | SEZ6L2 | ING5 |  |
| ADCY10P1 | SLC18A3 | IRGC | GOLGA2 | NUP58 |  |
| DUSP5P1 | ERVH-3 | SCN5A | CHD4 | ZNF181 |  |
| TMTC3 | VGLL2 | PLG | UBB | IFIT2 |  |
| SYF2 | CAPN5 | SEMA3F | RGPD5 | SAP18 |  |
| EVA1C | PANX2 | C1orf27 | ZNF618 | NF1 |  |
| USP32P2 | SCGB1A1 | PCAT6 | C6orf89 | ZNF566 |  |
| STAMBPL1 | HK3 | ADAMTS13 | UTP20 | LINC00545 |  |
| ARV1 | CAPN9 | KIR3DL1 | RPS2P47 | LINC00944 |  |
| NXF1 | FAM27E5 | GAS2 | TCF4 | SOX7 |  |
| UNC80 | TRIM42 | APOL6 | RBAK | AIMP1 |  |
| JAZF1 | GPR45 | SEPT7P2 | FAT1 | KMT5C |  |
| CLEC3B | CENPB | HMGCR | LIMS1 | ZP3 |  |
| ZSCAN29 | ASB6 | LOC643733 | HEBP2 | WNK1 |  |
| TTC14 | LRRTM1 | PLD1 | CSNK2A1 | DTWD1 |  |
| TRPV1 | SEC14L2 | KDM8 | COPA | SCIMP |  |
| NIPAL2 | FGF1 | INSM1 | NFIX | CBWD5 |  |
| FLJ33360 | CFAP206 | ALDH3B2 | GEMIN5 | C14orf79 |  |
| ZNF337 | SOX4 | PBOV1 | NDUFV3 | ABHD14B |  |
| CACNB2 | PNMAL2 | CACNA1A | SPTBN1 | NANOG |  |
| TSPEAR-AS2 | CA9 | KCTD1 | ZFPM1 | SOX10 |  |
| AGPAT4-IT1 | CCDC57 | IL18BP | ZBTB45 | PGM5 |  |
| ZXDC | KRTAP2-4 | TREML1 | PCNT | C15orf52 |  |
| TRMT10A | SDS | HAGHL | NPEPPS | PVR |  |
| FBXO28 | SLC26A1 | CCL21 | C2CD5 | LCMT1-AS2 |  |
| PIDD1 | MADCAM1 | TRPM3 | RPS14 | HYAL1 |  |
| RAP2C-AS1 | EPHA7 | IGSF21 | THRAP3 | C14orf159 |  |
| C5orf42 | MARK2 | LOC100131232 | DHRS4 | KCNMA1 |  |
| DENND4A | PPP6R1 | HBZ | CUEDC1 | NUDT12 |  |
| MT1E | TSPAN9 | OR2C1 | EDARADD | ADAMTS13 |  |
| WDR19 | TCL1A | ZNF107 | COL14A1 | ZNF223 |  |
| RAB12 | SETD9 | LOC100130456 | RPLP0 | HIP1 |  |
| STRN | TMEM145 | PCDHB9 | CD3EAP | ZDHHC17 |  |
| FAR1 | LAMP5 | MYCL | APRT | IL6 |  |
| RBBP4 | AGAP2 | RIMBP2 | IGF2R | ZNF135 |  |
| FLJ42393 | PTHLH | ST8SIA2 | TULP3 | EIF4H |  |
| PLD5 | UBQLN3 | HIC2 | LINC00674 | ZNF625 |  |
| SSFA2 | CTF1 | CYP2A13 | IPO8 | ERP27 |  |
| GNPTAB | MTA1 | THEG | CD81 | ZNF681 |  |
| ANKMY1 | LOC728673 | LINC00950 | ZBTB10 | LOC100128288 |  |
| PAPPA | ACTC1 | CXorf36 | CCDC174 | ANKHD1 |  |
| TLR3 | HMOX1 | FFAR3 | ACTN1 | ATOH7 |  |
| PKD1P6-NPIPP1 | ZMAT4 | TEKT5 | TUBA1A | COL6A5 |  |
| SLC7A9 | MMP9 | LEPR | HIST1H4D | ZBTB7B |  |
| POU5F1P4 | FGF19 | TNFSF4 | RNASEH2C | ZNF154 |  |
| NXPH1 | PLA2G15 | LMOD2 | KPNA4 | MITF |  |
| SPIN3 | VGF | HTR7P1 | HNRNPU | LOC441666 |  |
| RYR2 | NEO1 | SPATA8-AS1 | TNPO1 | USP6 |  |
| VPS13C | CADM1 | C20orf203 | TGOLN2 | GPR135 |  |
| ZBTB16 | FAM162B | LOC149703 | RPL13A | SLC46A3 |  |
| PHBP19 | ZMYND15 | CD300LF | UBE2K | COMMD6 |  |
| SGCD | PAX4 | LOC647115 | ARF5 | VEGFA |  |
| AVIL | NACA | HGSNAT | GOLM1 | ZNF333 |  |
| FOXO1 | TTC30A | ADAM33 | ID2 | PTGS2 |  |
| EEF1D | IRX4 | CD177 | PTPRD | MLXIP |  |
| FKBP5 | MRGPRE | MUC3A | TMEM2 | CHIC1 |  |
| FSIP2 | C2orf50 | TSNARE1 | LZIC | ZNF432 |  |
| FAM200B | OXT | TLX2 | C5orf42 | ITGA9 |  |
| H1F0 | KCNJ15 | GCH1 | NISCH | WIF1 |  |
| SH2B1 | SEMA4F | IKZF1 | FBLN1 | FXR1 |  |
| UCHL5 | KCNQ1 | PACRGL | DCAF7 | AHCTF1 |  |
| COX19 | PIN1P1 | FZR1 | SRRM1 | ZFP41 |  |
| SEPT7P2 | CRP | CD44 | FGF13 | GBP3 |  |
| SEPT4 | SYT11 | SIGLEC6 | EIF5A | SAMD8 |  |
| SNRK | AGR2 | SLC17A9 | CASKIN1 | RDH12 |  |
| HSBP1L1 | PES1 | MELTF | CDK14 | ADAMTSL5 |  |
| AKAP13 | CHAT | SOWAHD | SNRNP70 | MARCH3 |  |
| ANKRD36B | KDM8 | TNIK | CNPY3 | GGT8P |  |
| ZNF248 | CSHL1 | CSF2RB | HYAL2 | SYNJ1 |  |
| DMPK | NPPC | NECAP1 | LOC100506990 | RBMXL1 |  |
| LNX2 | MET | MYC | GABPB1-AS1 | FLT3LG |  |
| LOC728613 | B4GALT1 | LINC01410 | TMEM97 | C16orf86 |  |
| ZNF177 | SPRN | ELMOD1 | ADGRA2 | KIR2DS2 |  |
| PON3 | PI3 | NRROS | PLEKHA1 | EMP1 |  |
| MAST2 | FJX1 | STMN1 | USP3 | CD33 |  |
| LIMS3-LOC440895 | IQCD | H2BFXP | ALKBH5 | USP36 |  |
| GOLGA2P7 | RHPN1 | OASL | SPOP | BCL6B |  |
| PHYKPL | C6orf25 | HRH3 | MXD1 | DBF4B |  |
| ADAM33 | KLRG2 | APC2 | OSBPL8 | IRX4 |  |
| ZFC3H1 | BAX | KRT85 | PTN | LOC100132790 |  |
| ZNF708 | ALPPL2 | CENPB | RPSA | BBS7 |  |
| CA5B | ODF3L2 | CISH | NKTR | DBIL5P2 |  |
| GOLGA1 | IL36A | LOC400794 | SERPINB6 | LOC100128164 |  |
| TRIM63 | HELZ2 | NRG1 | RNF115 | RPRM |  |
| POLG2 | MIF-AS1 | SYDE1 | ZNF322 | MZT2B |  |
| SEC24D | PKIB | LOC100505915 | BLOC1S6 | TTYH2 |  |
| ZNF507 | LOC284570 | ELFN2 | ZNF22 | DNAH1 |  |
| APC | GRAMD4 | TMEM174 | CD164 | BRSK1 |  |
| STAMBP | KRT16 | GPR173 | PIK3R1 | SLC25A25 |  |
| ECHDC2 | PTH2 | NOS1 | MIER1 | TPK1 |  |
| SUN1 | ANKRD33B | ZBTB22 | RASSF8 | XAGE3 |  |
| CEP85L | TMSB15A | KLHL14 | TMEM205 | DIP2A |  |
| USP49 | DACT2 | BTN2A1 | SCAND1 | RPL15 |  |
| ADAMTS10 | ASGR1 | NEFM | RNF213 | EME2 |  |
| CLCF1 | CASP8 | FYN | MAGED2 | LAMP2 |  |
| MMRN1 | CUX2 | C22orf31 | LARP1 | PLAGL1 |  |
| NAV2 | MYB | IFNL2 | RPL3 | RAD9A |  |
| LOC389906 | TTC22 | BEGAIN | ZC3H18 | CCDC82 |  |
| RBAK | RITA1 | PLPP4 | ZC3H7A | MFSD2A |  |
| IQCH-AS1 | MAPK15 | NKX3-1 | LRRC37A2 | ARMC2 |  |
| CERS4 | RASA3 | INA | ZBTB37 | BOD1L1 |  |
| ZNF222 | GPSM1 | MSI2 | CEP85L | ABCC13 |  |
| CCDC30 | INHBC | PTGES2-AS1 | SLC35B4 | ERVW-1 |  |
| MTR | MTSS1L | PEG10 | VAMP3 | TRABD2B |  |
| OR2A9P | CDKN2C | RTN4RL2 | RPS28 | ZFYVE16 |  |
| GOLGA8EP | PAX7 | AP2A1 | PPP4R2 | PPP2R2A |  |
| PATJ | CACNG8 | HTR3A | TTLL12 | CTAGE5 |  |
| C1orf101 | LELP1 | MPP6 | POM121 | SMCHD1 |  |
| LPIN1 | HBQ1 | PI3 | TAF2 | GNG4 |  |
| RABGAP1L | MATN4 | DOK5 | ZNF644 | INVS |  |
| ICA1L | LTBP3 | LOC101927178 | REXO4 | NOL10 |  |
| MPPED2 | H2AFX | PRR4 | SPC24 | NEBL |  |
| LOC100133091 | LHB | MGAT4A | NONO | ZNF780A |  |
| DKK1 | AIF1L | LILRP2 | BTN2A2 | FASTKD2 |  |
| HMBOX1 | HBD | ZNF585A | ARFGEF2 | CXorf57 |  |
| ATG4D | CECR6 | PDGFB | POU3F3 | ZNF738 |  |
| RAD50 | KRT17 | MUC20 | ZNF257 | RRN3P2 |  |
| KIAA0825 | FOXE1 | ROGDI | DUT | CCDC120 |  |
| PIKFYVE | SLC7A14 | STAG3L2 | DCAKD | AXDND1 |  |
| FOS | STMN3 | IGLON5 | CNN3 | SPATA6L |  |
| LMF1 | SPN | DYRK1B | BUB3 | LINC01001 |  |
| BMS1 | SIRPB1 | HIP1 | NPDC1 | NDUFA10 |  |
| BTRC | CDCA4 | PRRT1 | GINM1 | PEG10 |  |
| SUGP2 | IQCF4 | TNRC18 | GATAD1 | ZNF175 |  |
| ARHGEF9 | LILRB3 | AOX1 | IFT43 | BACH2 |  |
| APOLD1 | CXCR1 | TNFSF13B | PGD | STARD5 |  |
| MYL9 | PTK7 | VPREB3 | C7orf50 | C1orf27 |  |
| ANKRD20A5P | KMT2A | ADGRB1 | RACK1 | C17orf78 |  |
| DIS3 | KRTAP2-3 | VTN | RAC1 | GBP1 |  |
| SWAP70 | GAGE3 | HECTD2 | ZNF302 | DCLK1 |  |
| EZH1 | CRLF2 | CD69 | MLLT10 | MATN3 |  |
| KIAA0319L | GRID2 | TMEM154 | HNRNPC | TAS2R13 |  |
| ZNF589 | CLDN19 | ALOXE3 | JAM3 | TPT1-AS1 |  |
| U2AF1L4 | FDXR | ADAMTSL2 | UBTD2 | LPCAT2 |  |
| D2HGDH | PMEL | SHC1 | CCDC6 | USP15 |  |
| LOC100129406 | SHC3 | FMO1 | YWHAE | THSD7A |  |
| MIRLET7BHG | ODF3 | IQSEC1 | SEC63 | CDHR1 |  |
| U2SURP | TMEM255B | GPR3 | ARHGEF35 | STXBP1 |  |
| ARF1 | ONECUT2 | TNNI1 | CD320 | SMURF1 |  |
| VN1R1 | EFR3B | NAT8 | NOL8 | CHRD |  |
| FAM160A2 | NFE2 | IRAK3 | YLPM1 | ATP7A |  |
| LOC728743 | FZD10 | ST18 | ARF1 | ERVH-3 |  |
| CYB5R1 | FBXL17 | OLIG1 | TXN2 | RASA2 |  |
| RASEF | CHRNA2 | ABCA2 | HLA-B | TBX19 |  |
| ZDHHC11 | LZTS1 | RBPJL | PALLD | KLRF1 |  |
| HAND2-AS1 | AQP2 | NXF2 | PTGES3 | FAM106A |  |
| ANKRD26 | LOC100130078 | POU3F1 | EXTL3 | BMP3 |  |
| GOLGA8T | DMD | SMOC1 | KDM2A | TAS2R19 |  |
| NPIPB5 | KCNA2 | PRM1 | PTPN14 | ZNF664 |  |
| TARID | OPA3 | IGDCC4 | COL4A3BP | KDR |  |
| SLC16A1-AS1 | USP6NL | PTPRR | GLTSCR2 | ABL2 |  |
| GRAMD3 | LOC441204 | CTAGE5 | UBE2G2 | GATA6 |  |
| ZNF655 | RNPS1 | LCK | EVA1B | OSMR |  |
| APOL4 | KIF25-AS1 | P2RX1 | ZNF236 | SPESP1 |  |
| NLGN2 | FAM83H | BTN2A2 | OGDH | GUCY1A3 |  |
| SIGLEC11 | C5orf64 | LOC284570 | FMN1 | ZNF548 |  |
| KALRN | ESPL1 | CCDC144B | ANP32B | AKAP6 |  |
| SLC35E2 | CBY1 | PMS2P9 | TPCN1 | FILIP1 |  |
| MT1B | PIP | CDK5R1 | SF3B3 | PGS1 |  |
| USP12 | TRPV5 | MAGEB2 | BOD1L1 | SFTPA1 |  |
| BCL2L11 | DNALI1 | CASC2 | PSMD1 | WWC2 |  |
| GSTM2 | ALG1 | ELN | QRICH1 | FAM132A |  |
| DIAPH2 | IRS1 | NFKBIB | PPP1R3D | ATF6B |  |
| EDRF1 | OR1F2P | CPTP | DNPH1 | PCLO |  |
| TDRP | RGR | SORCS1 | ZFX | NOS1 |  |
| LYRM9 | SLC5A2 | TMEM151A | MCAM | ZNF99 |  |
| DZIP3 | DEUP1 | NRP2 | SH3KBP1 | EXD3 |  |
| NAP1L1 | TBL1XR1 | LINC01590 | SLFN5 | HOXB3 |  |
| BMS1P6 | ECEL1 | EPHX4 | BMS1P6 | ZNF614 |  |
| ADAMTSL4 | MRPS6 | AKAP6 | ARL6IP4 | CUBN |  |
| ZNF675 | SFT2D3 | SPTB | CCDC191 | GYG2 |  |
| ATG16L1 | FNDC3B | ST8SIA5 | IPO7 | PTGR2 |  |
| ANP32A-IT1 | NEU4 | FAM180A | GNA12 | ABCD1 |  |
| CCSER2 | HBB | CHST8 | ZNF493 | BBS5 |  |
| PTPN4 | FAM47C | DUOX2 | TNRC6B | SLC35F1 |  |
| UBR4 | MCM2 | CSAG4 | RASSF1 | CGA |  |
| LRRC37A4P | TRIM55 | SLC12A7 | LSM10 | PKD1 |  |
| STAG3L3 | PLPPR3 | DRD5 | TMSB10 | PTGES2-AS1 |  |
| ZBTB44 | FOXD2 | SLC26A1 | BCL9 | WDR48 |  |
| LOC202025 | NREP | LOC339803 | ATG9A | TRAF6 |  |
| SACM1L | DMRTC1B | ST8SIA3 | HIST1H3G | EFHD1 |  |
| PRPF3 | IFITM5 | FCAMR | PGLS | CCDC88A |  |
| MYO15B | PLAT | RUSC1-AS1 | RPLP0P2 | GOLGA8S |  |
| NHLRC2 | CGB3 | ESRRG | FGD4 | DNAJC24 |  |
| GPATCH2L | CCDC166 | ALPI | HIST1H2BJ | METTL22 |  |
| SGK2 | ASPDH | FGD6 | SEC61A1 | MDM1 |  |
| GLIDR | UTF1 | GFI1B | RBM25 | ELOVL7 |  |
| SECISBP2 | WFIKKN2 | LOC100130357 | VASP |  |  |
| MT1M | CXCR4 | CSH1 | COL1A1 |  |  |
| FAM45A | SLC6A18 | KCTD7 | MPP5 |  |  |
| CLK4 | TRIM16L | FAM65A | HNRNPH1 |  |  |
| SNORD22 | SIGLEC15 | SEPT6 | MTMR1 |  |  |
| AVPR1A | IL1RL2 | JOSD2 | MAP2K6 |  |  |
| ANKRD36 | LOC105376360 | ITGB2-AS1 | C12orf65 |  |  |
| FANCM | C10orf2 | ODF4 | CCT7 |  |  |
| PLCL1 | CEND1 | SPDYE1 | TUFM |  |  |
| AP2B1 | OTUD7A | FOXI3 | EIF3G |  |  |
| PRAC1 | NBEAL2 | WHRN | CEP89 |  |  |
| PRR16 | KRT6B | SHISA9 | OLA1 |  |  |
| ERMARD | FDCSP | LRMP | RASSF4 |  |  |
| CTNND1 | CYP4V2 | KLC2 | POLR2L |  |  |
| IRS2 | ADARB2-AS1 | ODAM | NOP56 |  |  |
| SLC4A3 | C14orf169 | LINC01128 | RUVBL2 |  |  |
| PCBP1-AS1 | TNFAIP8L3 | LLGL1 | KEAP1 |  |  |
| CSAD | TRMT44 | THPO | KLHL24 |  |  |
| VTA1 | COTL1 | KIFC2 | HIST1H2AM |  |  |
| SNED1 | ELOVL6 | ATAT1 | SRSF4 |  |  |
| MTRF1 | NFE4 | GPR35 | SRSF9 |  |  |
| CAMK2D | KRT14 | LOC100509780 | ZNF621 |  |  |
| STT3B | INF2 | RXFP3 | ERGIC1 |  |  |
| ZDHHC8P1 | KCNC1 | FAM120C | KIF22 |  |  |
| UTP23 | TCP10L | SGK494 | YKT6 |  |  |
| SSH1 | HCN1 | IL17F | UROD |  |  |
| TCP11L2 | GABRD | ZSCAN1 | COPG1 |  |  |
| LINC-PINT | AOC2 | FGFRL1 | C19orf57 |  |  |
| CLK1 | TREML1 | TLR6 | CD86 |  |  |
| COLQ | AOC1 | AKT2 | PPP1R9A |  |  |
| GUSBP3 | DUSP8 | DMRTB1 | RALY |  |  |
| MFSD4A | SIRPG | CPNE6 | TENM3 |  |  |
| DAAM1 | SERPINA11 | KRTAP2-3 | FGFR1OP2 |  |  |
| TLK2 | MISP3 | OSGIN1 | ETV6 |  |  |
| DMXL1 | FAM65A | MYOC | TTPAL |  |  |
| APOL3 | HRK | INVS | MECOM |  |  |
| LOC100130476 | C2CD4B | BEST2 | HNRNPL |  |  |
| CFD | CABLES1 | EPAS1 | EIF3A |  |  |
| HPSE2 | NOS3 | SLC34A1 | TEAD1 |  |  |
| SFMBT2 | FBXO2 | HSPB6 | GAPDH |  |  |
| ATXN2L | CTBP1-AS2 | PPAN-P2RY11 | GALNT4 |  |  |
| PDE7A | LMNB1 | LOC653160 | CRIP1 |  |  |
| GNB3 | ANKLE1 | LINC00483 | DGCR6 |  |  |
| GNAS | ESCO2 | CGB3 | TYW1 |  |  |
| MAP2K6 | ZNF697 | KCNK4 | CAMK2D |  |  |
| SLC12A6 | LRRC45 | LRP10 | SLX1A |  |  |
| MKNK1 | MAGED4B | CIRBP-AS1 | NAA16 |  |  |
| LINC01410 | NKX2-5 | VGF | LOC389765 |  |  |
| PAFAH2 | STXBP5L | CXCR5 | TCOF1 |  |  |
| RAMP2-AS1 | LOC101927827 | CORO6 | DCBLD2 |  |  |
| AP1G2 | LOC390705 | KCNG1 | C6orf118 |  |  |
| FLJ36840 | HILS1 | HHLA3 | ZNF511 |  |  |
| FAM46A | AMPH | PML | APLP2 |  |  |
| RPS6KC1 | SRSF9 | GIPC3 | RPL15 |  |  |
| ZNF91 | BBC3 | OR10J3 | SAFB2 |  |  |
| APOD | GDF1 | MORC1 | HPS1 |  |  |
| ANKRD18A | ADGRG5 | ODF3L2 | PIGY |  |  |
| SDPR | PAFAH1B3 | CLCNKA | CCDC88A |  |  |
| PYGO1 | TNFRSF10C | SPTBN5 | DDR2 |  |  |
| ENOSF1 | DRD3 | BORCS8-MEF2B | NAPRT |  |  |
| HSD17B6 | LCK | FUT8-AS1 | YES1 |  |  |
| UBE2W | FBXL14 | CBARP | EMP2 |  |  |
| IDS | SQSTM1 | C12orf49 | MAP3K1 |  |  |
| LIG4 | MGC45922 | SP3 | TESK1 |  |  |
| PRKAA2 | BCL11B | KRTAP6-3 | EEF1G |  |  |
| ZNF585A | KLHL33 | ACBD4 | MIF |  |  |
| CXorf36 | GRM5 | PTPN23 | SLC30A7 |  |  |
| TTC7B | KISS1 | ZFYVE26 | NUDT19 |  |  |
| LOC286161 | FCAR | MAPK8IP3 | TBL1XR1 |  |  |
| CEP295 | CDK5R1 | C1orf21 | AKT3 |  |  |
| FAS | SOCS1 | KISS1 | PDIA4 |  |  |
| FGD4 | TNFRSF21 | PTGER4P2-CDK2AP2P2 | CLCN3 |  |  |
| LINC00515 | KCNQ1DN | B3GNT6 | RBM5 |  |  |
| DENND1B | SHISA9 | STAG3L3 | GTSE1 |  |  |
| PHACTR1 | FHOD3 | CDHR2 | CDK2 |  |  |
| RALGAPA2 | STK11 | NAF1 | SERINC5 |  |  |
| PIGG | TLX2 | CAPN5 | CDCA2 |  |  |
| FGF7 | MTMR11 | PLPPR2 | OLFML3 |  |  |
| CCDC85A | MIR22HG | ZNF780A | RANGAP1 |  |  |
| CENPT | ZBTB47 | CRISPLD2 | SNRNP48 |  |  |
| PPARA | B3GNT9 | KRTAP4-12 | RPL29 |  |  |
| DPP8 | ZNF575 | KRTAP13-4 | HOXB8 |  |  |
| PRLR | INSM1 | VSIG1 | AP2B1 |  |  |
| ADAMTS1 | LMOD2 | ZNF843 | KLHDC10 |  |  |
| C5orf56 | TPGS2 | FOXN1 | CTAGE5 |  |  |
| MIR497HG | SRD5A1 | OPN4 | ZFP91 |  |  |
| TRDMT1 | CES3 | TLR7 | WEE1 |  |  |
| FBN2 | TMEM92 | ALS2 | PDE5A |  |  |
| ZNF586 | IRGC | NPTXR | CBX5 |  |  |
| LOC727820 | SGCG | C5AR1 | KLHL42 |  |  |
| FAM78B | DCAF12L1 | OXTR | NETO2 |  |  |
| ZNF783 | KIF26B | PROKR2 | FLJ37453 |  |  |
| TAS2R43 | MAGEC1 | ARMC2 | LOC100190986 |  |  |
| MAGI1 | C10orf91 | AKNA | CALB2 |  |  |
| HERC5 | THRSP | TRIM50 | HIST1H3H |  |  |
| UBQLNL | PRAMEF1 | SIK1 | H3F3A |  |  |
| GEM | RBPJL | P2RY8 | MVK |  |  |
| TRMT1 | FOLH1 | MCCD1 | MPHOSPH8 |  |  |
| LCLAT1 | PTGIR | HRH2 | HSPD1 |  |  |
| SPAG9 | TTC33 | CDC42EP1 | LPP |  |  |
| NT5E | FAM222A | SH2D5 | GNPTAB |  |  |
| DUOX1 | LIPE | FAM175A | PAXBP1 |  |  |
| LOC101928731 | ATCAY | HR | ACOT7 |  |  |
| CDKL3 | POM121L4P | MICU3 | RAB14 |  |  |
| TBC1D8B | ENKUR | TG | NUSAP1 |  |  |
| TYW5 | GPRIN2 | FAM131B | NF2 |  |  |
| DCLRE1C | TBX10 | JARID2-AS1 | GTF3C5 |  |  |
| ENPP1 | GLTPD2 | DYSF | BECN1 |  |  |
| SEC16B | BMP7 | CSNK1A1 | HIST1H3E |  |  |
| FMNL1 | RAD54L | VNN2 | YWHAB |  |  |
| PRG4 | WNT9B | SBK2 | MRPL12 |  |  |
| CXCL13 | DUOXA2 | TBX6 | CCS |  |  |
| LOC100507053 | C5orf52 | VCAM1 | GOLIM4 |  |  |
| TRIM52 | ST14 | TBX10 | PDP1 |  |  |
| YWHAG | EXOSC5 | FAM179A | SSH1 |  |  |
| ARHGAP21 | CFH | PPP1R3F | MOB2 |  |  |
| TMTC1 | PITX1 | KCNE1 | RANBP2 |  |  |
| BICDL2 | WWTR1-AS1 | KLF2 | NUDT1 |  |  |
| PPFIA1 | FADD | RHOH | KMT2E |  |  |
| PPP3CB | LYNX1 | SLC22A18 | PARP14 |  |  |
| FAM153B | SDC1 | RPS6KL1 | UBAP2L |  |  |
| ZNF823 | MUC6 | CX3CL1 | CCNY |  |  |
| TTC26 | TRIM15 | MGAM | DAXX |  |  |
| FBXL4 | CDH10 | IL36B | RNPS1 |  |  |
| MYZAP | TRGV5 | ELOVL3 | HACD3 |  |  |
| LUC7L | FLJ30901 | TMEM27 | SNX29 |  |  |
| TNRC6A | MS4A12 | CASKIN2 | UGGT2 |  |  |
| DUSP8 | GRIN2C | SAMD12 | TOMM40 |  |  |
| TMEM237 | LGR6 | RGR | RSL1D1 |  |  |
| CALHM2 | LOC100289094 | RTBDN | ALYREF |  |  |
| CFAP44 | LSR | KCNJ12 | GNAS |  |  |
| HAL | LOC146880 | TCOF1 | DDB1 |  |  |
| DCAF13 | C22orf34 | ADD2 | SEPHS2 |  |  |
| BROX | RIBC2 | MYADM | HIST1H1D |  |  |
| AGER | FUT6 | CCL13 | OAZ1 |  |  |
| AASS | MFSD9 | PANX2 | MTRNR2L2 |  |  |
| LOC440434 | CLDN10 | CPNE7 | HLTF |  |  |
| ZNF254 | CFAP65 | AFF3 | METTL21A |  |  |
| TMEM158 | OSM | SCNN1B | CXXC5 |  |  |
| SLC22A3 | HBG1 | GAGE7 | CTBP1-AS2 |  |  |
| TSPAN2 | ZNF703 | MYL7 | EIF3B |  |  |
| UCA1 | LINC01158 | MIR17HG | CHMP4B |  |  |
| PALMD | NEK8 | ABAT | EML4 |  |  |
| WHSC1 | BRINP3 | C3orf20 | ASH1L |  |  |
| ZNF587 | C15orf48 | WWTR1-AS1 | LGALS3BP |  |  |
| ROR1 | TSPAN10 | OR8B8 | RIF1 |  |  |
| SYNJ2BP | DPEP3 | G6PD | NELFA |  |  |
| DNM1L | HSFX1 | SLC51A | CHM |  |  |
| LINC01061 | SHISA5 | XPNPEP2 | TMEM14B |  |  |
| GTF2H2B | ITIH3 | FAXC | NDUFB9 |  |  |
| ZNF224 | FLJ90680 | TBC1D17 | H2AFX |  |  |
| FLJ34503 | FAM117B | SYT5 | DNLZ |  |  |
| PLAA | KCNC3 | CDH23 | ASRGL1 |  |  |
| FSTL3 | KIF1A | CCPG1 | CCDC25 |  |  |
| ORC4 | VAMP2 | WNT11 | DENND2A |  |  |
| PPP1R12B | NUDT4 | ATOH7 | MAP1B |  |  |
| ANGPTL5 | TMEM252 | GSX1 | RPS16 |  |  |
| FBXW11 | LINC01304 | LOC439938 | DDR1 |  |  |
| SETD6 | WDHD1 | C1orf61 | RTN3 |  |  |
| CDKAL1 | TMEM25 | FRMD8P1 | LCE1D |  |  |
| VCX3A | MYH10 | ATOH8 | AKAP13 |  |  |
| RCSD1 | ROBO1 | OTOP2 | GATAD2A |  |  |
| MAPK8 | ACSS1 | MYL4 | SYNJ2BP |  |  |
| CSGALNACT2 | PRR35 | FUT2 | TIMM13 |  |  |
| INO80E | RETN | KIR2DL2 | GPI |  |  |
| KRIT1 | RYR1 | TSPAN32 | TOMM6 |  |  |
| KATNBL1 | PRR7 | CD5 | HIST3H2A |  |  |
| SPACA6 | SHANK2-AS3 | LINC01405 | CTSA |  |  |
| KLF9 | TMEM132D | CASC3 | MTCH1 |  |  |
| TRPM6 | LOC100130456 | PHYKPL | TPR |  |  |
| CYP4Z1 | GFRA2 | DOK4 | CHD6 |  |  |
| MMP19 | TMEM106C | HDAC4 | ACTG1 |  |  |
| SOWAHC | HAS3 | ATP10A | CTNND2 |  |  |
| ZNF257 | MYL4 | NACC1 | ACIN1 |  |  |
| TRIM38 | WNT6 | MAGIX | AKR7A3 |  |  |
| MTO1 | CTHRC1 | ZC3H10 | GPAA1 |  |  |
| ZNF440 | OLFM1 | LINC00896 | DNAJC14 |  |  |
| SYNC | RAX2 | LYPD4 | NUMA1 |  |  |
| ZNF92 | FPR1 | NOVA1 | TOMM20 |  |  |
| CRISPLD2 | UBFD1 | SH3GL1 | PTK7 |  |  |
| SPPL3 | LINC00896 | UPB1 | MPG |  |  |
| CYR61 | SPI1 | ADAMTS14 | PSMA4 |  |  |
| GGTLC1 | FFAR4 | LIG3 | TRIM26 |  |  |
| FLJ30901 | MMP12 | IL1RL2 | NAB1 |  |  |
| EXOC4 | FCN1 | LOC200726 | KRT19 |  |  |
| DCAF5 | GNB1 | IL18RAP | BCOR |  |  |
| RPF1 | SEMA4B | IQCE | PPP1R35 |  |  |
| ADPGK | ANK1 | SLC30A3 | SYNGR2 |  |  |
| B3GALNT2 | NXNL1 | STAC2 | IRX3 |  |  |
| LOC100506282 | FUT8 | HSPB9 | BTC |  |  |
| KIF27 | LEF1 | INSL3 | RPS6KA1 |  |  |
| TIAL1 | HOXC12 | CYP2S1 | BZW1 |  |  |
| ZNF160 | POM121L1P | CAPN13 | KLC3 |  |  |
| PNPLA8 | LOC105369486 | IGF2 | BCR |  |  |
| THBS3 | UPK3A | KANK3 | NR1H3 |  |  |
| GTF2IRD2 | CXCL5 | MAB21L3 | RBP1 |  |  |
| ZBTB8A | GLTSCR1 | PGS1 | PABPN1 |  |  |
| DTX2P1-UPK3BP1-PMS2P11 | MAP3K14-AS1 | TMEM140 | TCEB2 |  |  |
| ALDH8A1 | ESRRB | ACADSB | CEP170B |  |  |
| WDFY3-AS2 | NLRP11 | CHST11 | PMS2P3 |  |  |
| DOCK5 | CPE | OR4S1 | PRCP |  |  |
| RELN | FRMPD3 | RGMA | ST6GALNAC6 |  |  |
| PAK3 | CASKIN2 | NATD1 | KANK2 |  |  |
| ZNF510 | APOBEC3B | CALY | EBP |  |  |
| SH3GLB2 | MSMB | FDCSP | PLCG1 |  |  |
| LYVE1 | KCNK7 | GHSR | EZR |  |  |
| TRIM22 | FZD1 | LMO1 | HCG18 |  |  |
| SYCP2 | CDH22 | OTX1 | FGFR1 |  |  |
| ADARB1 | LOC100996342 | ALDH3B1 | TCF7 |  |  |
| NUFIP2 | GABRQ | CCL17 | RNF19A |  |  |
| DICER1-AS1 | PROKR1 | MUC17 | NDUFV1 |  |  |
| PDLIM7 | HEXIM2 | MMP28 | BNIP3L |  |  |
| ZNF169 | NPY4R | FURIN | PTEN |  |  |
| DIRAS3 | TMEM239 | CREB3L3 | CDC42EP4 |  |  |
| USP53 | EN2 | LOC105370612 | PIGT |  |  |
| KLHL17 | NEUROG1 | HMX1 | TTC30B |  |  |
| NUDT13 | C1orf109 | RAP1GAP | RPL21 |  |  |
| SERPINA4 | NTRK2 | LENG8 | NELFB |  |  |
| ZNF680 | ARHGEF38 | LTK | FCRLB |  |  |
| AFF3 | LOC102723968 | CYP27B1 | TMEM121 |  |  |
| MIR1245A | HHIPL1 | COL20A1 | AHI1 |  |  |
| THPO | ATXN7L1 | NUDT13 | RGL1 |  |  |
| RIN3 | ZBED6CL | FAM122C | FAM13A |  |  |
| TTC37 | CSH2 | CYP2B6 | POLD1 |  |  |
| CACNB4 | LINC00094 | LAT2 | IER5L |  |  |
| PPHLN1 | CENPH | LINC00675 | TUBB8 |  |  |
| LOC105377134 | JCHAIN | NECTIN3 | PPP1R2 |  |  |
| CD55 | E2F2 | TPCN1 | PRR14L |  |  |
| MAN2C1 | IGHA2 | LOC105372179 | HEXA |  |  |
| KLHDC4 | BCL2L11 | IL27RA | CHID1 |  |  |
| TJAP1 | CLDN23 | GPR149 | FICD |  |  |
| COL11A2 | RAB3B | CBR3-AS1 | CTBP1 |  |  |
| PID1 | CSAG1 | LGR6 | STX8 |  |  |
| KIAA1107 | IL19 | LOC440434 | DPF2 |  |  |
| FAR2P1 | PEAR1 | CD274 | TAF13 |  |  |
| ASB14 | RTF1 | KATNAL2 | ZNF579 |  |  |
| EGFL8 | MUC5AC | ZNF25 | DPM3 |  |  |
| RHOXF1 | LOC100132014 | TCTE1 | ORAI2 |  |  |
| GALNT15 | RSPH6A | FLYWCH1 | PHF8 |  |  |
| TIMP3 | PRKAR1A | PYY | BAP1 |  |  |
| STAC2 | KLK3 | FAM92A1P2 | CDC14B |  |  |
| NPLOC4 | DBH | KIAA1614 | AP1G1 |  |  |
| STXBP5 | LINC00589 | BCAN | ANO1 |  |  |
| DICER1 | NDOR1 | LOC100132319 | NEK1 |  |  |
| LINC00937 | SHISA4 | STK38 | BOLA2-SMG1P6 |  |  |
| GUCY2F | AHSG | AZU1 | TMEM256 |  |  |
| PCM1 | MCF2L | GUSBP3 | MRPL41 |  |  |
| BTG2 | AUNIP | MAP7D2 | ATMIN |  |  |
| ZNF431 | NANOS3 | NCR1 | LCE5A |  |  |
| MYSM1 | BCL2L13 | LINC00597 | RGS3 |  |  |
| TMEM87A | FARP1 | TCAF2 | CLINT1 |  |  |
| BAZ2B | CPXM1 | LOC105376633 | NASP |  |  |
| ITGBL1 | TRIB2 | KCNH4 | SLC25A6 |  |  |
| DIS3L2 | IFNL2 | SLC29A2 | MYO9B |  |  |
| MCEE | CFAP47 | MS4A1 | XRCC6 |  |  |
| RAB7B | RCC1 | AGTR1 | CSNK1G1 |  |  |
| JMJD1C | DPYSL4 | LARP1 | TOR1AIP2 |  |  |
| SCML1 | CCDC86 | IL10RA | MVD |  |  |
| PAXBP1 | C22orf46 | PRM3 | AMOTL1 |  |  |
| LINC00152 | PRORY | HEATR4 | PYCRL |  |  |
| ZNF397 | CDC25A | PLVAP | SLF1 |  |  |
| C19orf43 | C2orf54 | DNM3 | ZNF3 |  |  |
| KIF3B | C22orf15 | RASGRP4 | PSMD13 |  |  |
| TTC21B | SLC1A4 | TOR2A | ZNHIT1 |  |  |
| MAP3K12 | RXFP3 | FASLG | IMPDH2 |  |  |
| LOC100506990 | PRRT3 | NAT8B | RPL7L1 |  |  |
| LGALS8 | CRYAA | CYP2F1 | TSC22D1 |  |  |
| AKT3 | SLC34A3 | TAS2R60 | NIPBL |  |  |
| ZNF75D | IL27RA | PROCA1 | NUCKS1 |  |  |
| CAB39L | LGALS12 | LAT | KIDINS220 |  |  |
| PXN | TAT | MAP4K2 | B3GALT5-AS1 |  |  |
| POLR3A | PRSS33 | ZZEF1 | ATG16L1 |  |  |
| ZNF644 | TK1 | TRABD2B | ITSN2 |  |  |
| ADCY4 | POLD3 | C17orf74 | RSRC1 |  |  |
| GAB1 | MYH15 | CTTNBP2NL | SPAG16 |  |  |
| PTK2 | JARID2-AS1 | EPHB2 | LEPROT |  |  |
| USP32P1 | SCLY | PCP4L1 | SMG6 |  |  |
| LSMEM1 | FRMD1 | BMP6 | YWHAH |  |  |
| SLC30A2 | SMAP1 | RPRM | GPR107 |  |  |
| SLC7A11 | RLN2 | ADAM22 | ZNF696 |  |  |
| CUL1 | ZNF548 | FGD2 | HGSNAT |  |  |
| SOX5 | ALYREF | TINCR | SURF2 |  |  |
| ZMYM6 | SLCO1A2 | DCANP1 | ACTR1A |  |  |
| LIMK1 | ATXN1L | PLEKHF1 | CPTP |  |  |
| HTR2A | NELL1 | BCL2L14 | GTPBP8 |  |  |
| ATP8B2 | HIST1H4G | T | SLC35E3 |  |  |
| CARD11 | IL10RA | ASAH1 | EXT1 |  |  |
| RNF217 | ACOT11 | MFAP5 | CEP63 |  |  |
| SLC25A36 | MIER2 | CEP164 | ZNF777 |  |  |
| CALCOCO1 | SLC17A8 | IZUMO4 | GBP4 |  |  |
| SORBS2 | ACAD10 | PARK2 | GABARAP |  |  |
| USP21 | CCDC7 | GTPBP1 | KATNAL1 |  |  |
| KLHL24 | UGT3A1 | EGFL8 | TMEM260 |  |  |
| PFDN1 | UCN3 | DDIT3 | GTF2I |  |  |
| PTPN13 | C17orf99 | EPS15L1 | PHGDH |  |  |
| MTMR6 | EPHB2 | FYB | HADH |  |  |
| INPPL1 | PGF | SLCO3A1 | LRFN3 |  |  |
| DOCK10 | LINC00965 | GFRA2 | RCOR2 |  |  |
| LOC100129447 | LOC100127955 | ADAM6 | NDE1 |  |  |
| DKFZP434I0714 | PDCD1 | NPM2 | ZNF667-AS1 |  |  |
| SMG1P5 | PRKCZ | ADORA2A-AS1 | SLC4A7 |  |  |
| DDX55 | SV2A | AGER | BMP2K |  |  |
| TMPRSS5 | CA11 | KLHL4 | STT3A |  |  |
| RABGAP1 | CD1A | LCN1 | CERS2 |  |  |
| ZNF136 | CHAC1 | PCNX2 | FUNDC2 |  |  |
| LINC00472 | ZMIZ2 | PDE4C | RHOC |  |  |
| INPP5A | ANKRD36B | BCLAF1 | AFF4 |  |  |
| NEPRO | DLX3 | SH3RF2 | ALDH1A2 |  |  |
| LOC399716 | RREB1 | PLA2G4C | HIST3H3 |  |  |
| ZCCHC24 | CRYL1 | HSD11B1L | RBM15B |  |  |
| DENND4C | ADAM12 | HID1 | TBC1D23 |  |  |
| PSORS1C1 | TMEM229A | NUMBL | AHCTF1 |  |  |
| LINC01138 | STC2 | SLC22A3 | ZNF148 |  |  |
| MTMR9LP | THOC6 | BHLHE22 | C9orf24 |  |  |
| PLEKHG3 | HGS | RAX2 | CD276 |  |  |
| IFNE | SNX22 | SIGLEC5 | ZNF395 |  |  |
| SULT1A4 | NPTX1 | IQSEC3 | EIF4A2 |  |  |
| ZNF37A | CBX2 | KLHL21 | TSPAN1 |  |  |
| NRXN3 | TNNT2 | TMEM239 | COMMD4 |  |  |
| KLHL8 | LOC100131232 | LSMEM1 | C5orf24 |  |  |
| NTAN1 | QDPR | LMNTD2 | RPL39L |  |  |
| CPSF7 | CLVS1 | SRRM3 | IFI27L1 |  |  |
| PTPRB | NPY5R | CASP14 | MACF1 |  |  |
| ZNF781 | C22orf23 | NAXD | RPSAP36 |  |  |
| PTPN9 | POLR1C | GCM1 | ARID1A |  |  |
| FAM210A | HTR4 | SNCG | TDG |  |  |
| CNR1 | MAGEB1 | TRAF1 | BAGE |  |  |
| IGF2BP2 | FOXL2 | TNFSF15 | NT5C |  |  |
| C6orf136 | E2F8 | ALX3 | KAZN |  |  |
| ZNF800 | TMC2 | GUCY1B3 | KIAA1671 |  |  |
| UBAP1L | ZNF668 | SYVN1 | DGCR8 |  |  |
| XIAP | GRM7 | FFAR2 | TMSB4X |  |  |
| GRIK2 | GNG7 | MEGF11 | LAPTM4B |  |  |
| EPSTI1 | HSPC081 | IPO5P1 | SMG1P1 |  |  |
| ZNF571 | CTSV | OR1F2P | AMFR |  |  |
| CNOT4 | SEL1L3 | CCDC68 | YTHDF2 |  |  |
| NT5DC1 | C2orf70 | CHCHD5 | HLA-C |  |  |
| PLIN5 | SV2C | KSR1 | BRD4 |  |  |
| RMDN2 | LOXHD1 | RHOF | MPPED2 |  |  |
| GSTCD | GTSF1 | LOC101927598 | RSAD1 |  |  |
| ADAM20P1 | FOXB1 | JUNB | HNRNPUL2 |  |  |
| ADGRD1 | GLRA1 | C9orf135-AS1 | ZNF800 |  |  |
| MYCBP2 | LINC01547 | TTTY13 | ZNF445 |  |  |
| IRAK4 | LOC101927181 | SLC5A5 | ARPC5L |  |  |
| ZNF345 | TOR4A | PVRIG | OTUD4 |  |  |
| LRRK1 | MCM10 | ZNF594 | LIMCH1 |  |  |
| MT1F | KLC2 | CELSR2 | EHBP1 |  |  |
| MALT1 | TLX1 | EWSR1 | CIB2 |  |  |
| TTC17 | SERPINB3 | SNTB1 | EIF4G1 |  |  |
| CELF6 | ZNF630 | ACTRT2 | CDK3 |  |  |
| DGKB | SLC38A10 | VAC14 | TNIP1 |  |  |
| KRBOX4 | PDXK | ANXA8L1 | KRAS |  |  |
| SCAND2P | ADAM10 | PSMB2 | TRIB2 |  |  |
| CRYAB | FAM181A | CMTM1 | TST |  |  |
| MME | KRTAP19-1 | KLRF1 | CDK6 |  |  |
| MSL1 | AHSP | TPRG1-AS1 | DDRGK1 |  |  |
| WDFY3 | WDR3 | PCDH1 | GPC1 |  |  |
| NSD1 | ARX | ABI3BP | PRNP |  |  |
| TBC1D24 | KLK2 | HSD11B1 | ZNF629 |  |  |
| BRWD1 | LINC00599 | HMGA1 | HIST1H2AB |  |  |
| RAD54L2 | FAM3D | NGF | TACC1 |  |  |
| NT5DC3 | GJB5 | JAKMIP3 | GALR3 |  |  |
| CPT1B | PLXNA3 | ARC | MBNL1 |  |  |
| FMO5 | TSPAN33 | CACNG3 | TMEM159 |  |  |
| GLS | C17orf50 | RHCG | C17orf49 |  |  |
| NGLY1 | MBD3L1 | BEST3 | LSM2 |  |  |
| SESN1 | LMX1A | PAPOLG | CERS6 |  |  |
| MARCH6 | TCL6 | MDGA1 | APP |  |  |
| MKLN1 | SSTR5-AS1 | SMDT1 | LYPLA1 |  |  |
| KLF12 | SBSPON | MMRN1 | SYDE2 |  |  |
| ZNF595 | GPR25 | GRHL3 | CCDC85B |  |  |
| TSPOAP1 | DRAXIN | DKFZp434L192 | MRFAP1 |  |  |
| USP24 | LINC01126 | BANK1 | EEF1A1 |  |  |
| LINC01000 | CYP26B1 | SERPINB7 | WDR92 |  |  |
| DUOXA1 | SERPINB4 | C19orf66 | LRP6 |  |  |
| EIF2A | SKA3 | RMDN2 | HES7 |  |  |
| MCOLN2 | TMEM38B | SLIT1 | LOC101928433 |  |  |
| TMEM86B | RSPH9 | HCG25 | COX4I1 |  |  |
| RAB11FIP2 | NME5 | ELMOD3 | MAGED4B |  |  |
| WDR59 | DHH | ITGA2B | LASP1 |  |  |
| RAB24 | IL23A | SLC6A6 | NCAPD2 |  |  |
| ZNF791 | GJD3 | CCDC181 | CDCP1 |  |  |
| GABARAPL3 | LINC00661 | SLC16A14 | KIF6 |  |  |
| CNTN5 | HEBP1 | DRD4 | ZFC3H1 |  |  |
| DLEU2 | FOLH1B | FAM178B | GXYLT1 |  |  |
| WDFY1 | PFDN2 | TBXAS1 | GCHFR |  |  |
| EMD | SLC5A10 | GPSM3 | GUSBP1 |  |  |
| ZNF652 | TNFSF15 | RXRA | MFSD14A |  |  |
| PTPRG | PIFO | NKX6-3 | SEMA6A |  |  |
| ZNF761 | LINC00608 | ZMYM1 | PANK3 |  |  |
| WASH1 | TP73 | HECW2 | MARVELD2 |  |  |
| CLASRP | MEIOC | UBTD1 | YY1 |  |  |
| CUZD1 | WNT9A | JCHAIN | IFRD2 |  |  |
| FRMD6 | CPN2 | LOC100289470 | CRTC1 |  |  |
| DDX60 | PSG6 | RGS18 | WNT4 |  |  |
| SECISBP2L | PGM5 | BICC1 | CKAP4 |  |  |
| ADH1A | FLJ44715 | RIPK4 | SMCHD1 |  |  |
| IKZF2 | LOC105375092 | ADGRV1 | NTMT1 |  |  |
| BRWD3 | CCDC28B | USP36 | SH3PXD2A |  |  |
| IGIP | ZNF296 | TMEM252 | PSMD3 |  |  |
| HIST1H2BK | BLM | MUC5AC | UGCG |  |  |
| FAM133B | RAB36 | HRK | SYK |  |  |
| ZNF676 | PTPRK | SHC3 | RAB12 |  |  |
| GPATCH8 | CNTNAP5 | HEPACAM | RPL8 |  |  |
| TMEM184A | SPC25 | SLC13A2 | NAA38 |  |  |
| LMBRD2 | SHH | CHAD | PDE7A |  |  |
| ITGB8 | LYPD3 | MEIS3 | GPR153 |  |  |
| SUSD4 | PRIMA1 | KIR2DS1 | UBC |  |  |
| UBE2V1 | LYPD4 | MYO1A | RPS3 |  |  |
| C8G | ARSI | TRAF7 | HIST1H3A |  |  |
| MAP3K14 | C20orf85 | KDM6B | GAS2L3 |  |  |
| FAM27E2 | C16orf92 | REG1A | AGPAT3 |  |  |
| FAM46B | NMB | SCAMP1 | DYRK1A |  |  |
| EBLN2 | PACSIN1 | FERD3L | MRPL38 |  |  |
| CD44 | HNF1B | ZBP1 | IVD |  |  |
| FCHO2 | ADCY9 | CCRL2 | TBC1D5 |  |  |
| MRPS11 | SIPA1L3 | SLC46A1 | PDLIM5 |  |  |
| SLC39A14 | KRT13 | OR4C46 | TMEM11 |  |  |
| PRDM6 | PTCRA | PXN | SAC3D1 |  |  |
| ZNF283 | H6PD | LOC102723968 | LRRC75A-AS1 |  |  |
| YAE1D1 | HUS1B | CCDC9 | SLC39A3 |  |  |
| PBLD | CSPG5 | ATF6B | CSNK1A1P1 |  |  |
| SLC1A2 | HIST2H3A | SPNS2 | EEF1B2 |  |  |
| ZNF407 | QTRT1 | ANGPT4 | MIA2 |  |  |
| ZNF660 | CLN3 | LINC00837 | IER5 |  |  |
| RGPD2 | MAN2B1 | KCND2 | LSG1 |  |  |
| FAM214A | PARP1 | RBMXL1 | SUMO2 |  |  |
| FLCN | TDH | TMEM139 | ING4 |  |  |
| ZNF137P | SLC34A2 | TNFRSF9 | TK1 |  |  |
| MOB3B | LTA | SFRP2 | HGS |  |  |
| CEP104 | GUCA1A | NEK8 | CDT1 |  |  |
| TAPT1 | RCAN3 | C10orf54 | GOLGA6L9 |  |  |
| ATPAF1 | FAM110D | SIX3 | FADS1 |  |  |
| FOCAD | CFI | NTSR2 | MMAB |  |  |
| ZNF436-AS1 | PRSS21 | SPG20 | BAZ1B |  |  |
| LINC00857 | FLT4 | FGF18 | LOC389705 |  |  |
| KCNMB3 | IL1R2 | HS6ST3 | FAM173A |  |  |
| MTERF4 | NPM2 | SPATA1 | FAM193A |  |  |
| RGN | RAB26 | POLR2A | CYHR1 |  |  |
| TLR5 | BTG4 | SHISA7 | CSRP1 |  |  |
| GPR180 | SEMA3D | TUSC5 | YBX3 |  |  |
| SCRN2 | IRX1 | C17orf50 | MRPL28 |  |  |
| ZNF547 | SPACA6 | PRDM8 | EIF6 |  |  |
| LINC00265 | FBLL1 | SCD5 | PHF23 |  |  |
| VTI1A | SYCP1 | SHISA5 | PHLDA3 |  |  |
| PLPPR2 | MBP | CCL19 | ORAI1 |  |  |
| TERF1 | PLEC | SELPLG | MEIS3P1 |  |  |
| LOC100128988 | PLK4 | IL36A | MBOAT7 |  |  |
| ZNF211 | NHLH2 | TCF23 | SPAG7 |  |  |
| ULK3 | PLA2G2E | IGSF22 | MGC10814 |  |  |
| ZNF433 | CIDEA | ZNRF4 | BRWD3 |  |  |
| POLK | CRABP1 | PRKCA | ZNF726 |  |  |
| MBD5 | MARCO | FMNL1 | ATP2B1 |  |  |
| FAM9B | GPR62 | BGN | WDR36 |  |  |
| ANKRD13B | TANC2 | ALDH1A3 | QSOX1 |  |  |
| PGC | GFRA3 | TMEM71 | SLC39A7 |  |  |
| ULK4 | LBH | C8A | SERPINF1 |  |  |
| WDR75 | KLHL30-AS1 | CDH6 | NDUFS7 |  |  |
| CYP4V2 | DBN1 | GPX5 | MMP16 |  |  |
| NPR1 | C16orf86 | KCNE3 | DNAJC10 |  |  |
| POLI | MEX3B | AK1 | TMEM190 |  |  |
| DVL3 | AANAT | TLL2 | MRTO4 |  |  |
| ZNF117 | GRAMD1B | LPL | RANGRF |  |  |
| FBXL13 | S1PR5 | NKX2-3 | PXK |  |  |
| KCTD9 | SLC2A10 | DAB1 | IGF1R |  |  |
| TAF1 | AGPS | LINC00504 | GUK1 |  |  |
| HAUS6 | KRTAP4-9 | MKL2 | SMIM11A |  |  |
| MAMDC2 | MED24 | IL10RB-AS1 | TRIM2 |  |  |
| RPL21P44 | GPR52 | TNFAIP8 | UBA6 |  |  |
| FLVCR2 | CNR2 | HOXA2 | ABI2 |  |  |
| LNX1 | DAND5 | VAMP2 | MAP7D1 |  |  |
| ZNF513 | PRODH | LINC00115 | TET2 |  |  |
| S100A3 | ZNF341 | FZD1 | SDAD1P1 |  |  |
| TRMT13 | CA14 | GOLM1 | KATNBL1 |  |  |
| ANGPT1 | CD300C | OXT | PRPF6 |  |  |
| MARK1 | NEK2 | SELP | GNG12 |  |  |
| BCL2L2 | ATF6 | PRKCB | MRPL54 |  |  |
| KCNN3 | HNF4A | ATP7A | GNL3L |  |  |
| C9orf72 | CADM3 | APLNR | HIST1H4A |  |  |
| VPS37A | LOC649294 | SIGLEC7 | FUS |  |  |
| COLGALT2 | FAM181B | SNCA | CCND1 |  |  |
| ZNF44 | TMPRSS2 | VGLL2 | SMARCD2 |  |  |
| TET2 | FGD1 | PER2 | TPPP3 |  |  |
| MPP3 | EWSAT1 | ANKRD20A2 | HNRNPK |  |  |
| STS | LINC01234 | LRRCC1 | SBNO1 |  |  |
| SFSWAP | IL18BP | OLA1 | CENPC |  |  |
| HPCAL4 | MUC3A | PRRG3 | TPI1P2 |  |  |
| CD99P1 | SSTR2 | RUNX1 | ANXA2P1 |  |  |
| ZNF346 | SPDYE2 | RDH16 | ETFB |  |  |
| ZSCAN26 | MAP3K9 | USP15 | FAM161A |  |  |
| GP1BB | CKAP2 | RAB26 | USP10 |  |  |
| ARSG | KLHL34 | PAPLN | TMEM177 |  |  |
| NSUN5 | DCANP1 | GSG1L | B2M |  |  |
| SGK494 | LTBP2 | VSIG10L | PRKDC |  |  |
| F8 | KRTAP1-1 | SH3BP1 | HIST1H4J |  |  |
| IFT80 | SLC39A9 | TF | PHF19 |  |  |
| DKFZP586I1420 | SPRR2C | GJB4 | RALB |  |  |
| MT2A | STXBP1 | PRSS21 | UBXN6 |  |  |
| UBR1 | BEND3 | AHSG | RHBDD3 |  |  |
| ATF2 | KRTAP4-6 | DIO3OS | ARL5B |  |  |
| CSRNP2 | FABP2 | HNF1B | B4GALT1 |  |  |
| ASXL1 | LRRC72 | SLC25A47 | RXRA |  |  |
| MIR99AHG | RHO | PCDH8 | GBP3 |  |  |
| ZNF225 | SH3D21 | H2AFJ | TACC3 |  |  |
| MIGA1 | SIPA1L1 | GDNF | CALR |  |  |
| KPNA4 | IRX6 | LOC105376360 | TMPO |  |  |
| KMT5B | PKMYT1 | LOC100129397 | PEMT |  |  |
| ESYT3 | KIF19 | GALP | SGSM3 |  |  |
| LOC729603 | TGFBI | PCDH20 | IPO4 |  |  |
| C16orf70 | CDCA8 | ACOT11 | LIMD1 |  |  |
| ZNF880 | SCNN1B | GFAP | UBL3 |  |  |
| EBF1 | CDKN2AIPNL | TSACC | INSIG1 |  |  |
| ZMYM5 | HSPA1A | SSUH2 | RAB22A |  |  |
| RBMS3 | ISM2 | LINC01146 | B4GALT2 |  |  |
| ARHGEF37 | LOC101929450 | PEAR1 | YIPF5 |  |  |
| SLAIN2 | ALPI | SULT4A1 | KIF16B |  |  |
| UBE2Q2P1 | FAM81B | HSD17B1 | MIB1 |  |  |
| PIK3C3 | LOC100130156 | ZMYM5 | GOLGA8F |  |  |
| SPG20 | SMIM6 | SLAMF8 | TRPM7 |  |  |
| S1PR1 | LOC105370792 | SAMSN1 | PCBP1 |  |  |
| ZNF737 | SPSB4 | LENG9 | GOLGA3 |  |  |
| SYNE1 | KRBA1 | LINC00301 | EIF4G3 |  |  |
| CRHR1-IT1 | ERICH5 | FUT9 | HSPA12B |  |  |
| EIF4G3 | FAM20C | MGA | TOE1 |  |  |
| CHM | RRM2 | SFI1 | KAT6B |  |  |
| ZNF596 | LINC00684 | CYP2C18 | PPP6R1 |  |  |
| ZNF419 | SIN3A | RAB17 | LAGE3 |  |  |
| WHSC1L1 | MATN1 | CASQ1 | SNRPN |  |  |
| INE1 | SIGLEC5 | PPP6R2 | FAM134C |  |  |
| ANKRD12 | GPR18 | ANAPC2 | PRSS12 |  |  |
| SERPINF1 | C1orf64 | CCDC178 | RNF216 |  |  |
| ZNF17 | CPA5 | MID1IP1 | RPL7A |  |  |
| LOC221272 | FBRS | NODAL | TPI1 |  |  |
| KLRA1P | SCNN1G | KLC4 | MPDU1 |  |  |
| NABP1 | ST6GAL2 | FAM182B | RECQL4 |  |  |
| ADAMTSL3 | PPP1R9B | CYP4F2 | TAF1 |  |  |
| PDXDC1 | KCNN2 | COL15A1 | SCAMP1 |  |  |
| USP18 | SYT12 | PTPRU | HLA-H |  |  |
| DGCR8 | GATA5 | UROS | ATP11B |  |  |
| CCDC84 | C17orf64 | TMEM214 | ACP5 |  |  |
| TVP23C-CDRT4 | S100A12 | OR6W1P | APEH |  |  |
| DLST | PCDH10 | MRC1 | C3orf58 |  |  |
| BOLA2-SMG1P6 | WDR13 | SCIMP | THSD4 |  |  |
| ZNF730 | KLK15 | RELL2 | NSF |  |  |
| RSAD1 | PLAU | PRR3 | SAMD11 |  |  |
| LOC146880 | C7orf33 | PRUNE2 | PRMT1 |  |  |
| AES | GIPC3 | KLHL28 | RFXANK |  |  |
| C1QTNF3 | LRP2BP | DUSP5 | VPS28 |  |  |
| KIAA0907 | NPVF | LMF1 | THOP1 |  |  |
| TULP3 | ZFYVE1 | MIGA2 | FCHO2 |  |  |
| SPPL2B | CANX | MAPK8IP2 | ITSN1 |  |  |
| ACADSB | C14orf144 | ZNF229 | DCBLD1 |  |  |
| COX20 | MGC27382 | TSPAN10 | SLC39A1 |  |  |
| ZNF736 | IER5 | PLCD4 | ARHGAP35 |  |  |
| PLB1 | TGM7 | GDPD1 | CASP6 |  |  |
| ASAP1-IT1 | KLC4 | OTUD6A | PHB |  |  |
| ZBTB25 | MS4A1 | LINC00302 | PRPF4 |  |  |
| TMEM260 | LOC107985946 | PHKA1 | C21orf33 |  |  |
| ADAMTS6 | EMID1 | MTSS1L | ZNF587 |  |  |
| TMEM147-AS1 | FAM131A | DCT | PIH1D1 |  |  |
| SLC9B2 | MBOAT7 | MED23 | NMRAL1 |  |  |
| IRF3 | FEN1 | LRRC18 | FAU |  |  |
| C10orf54 | SI | CHGA | CCDC59 |  |  |
| MLLT6 | VSIG10L | RASGRP1 | ZNF664 |  |  |
| WBP2 | GNS | ABHD1 | HADHA |  |  |
| USP34 | PPIF | NUDT4 | FBL |  |  |
| ZNF716 | FAM71E2 | ALPPL2 | ELAC2 |  |  |
| OTUD4 | CHGA | HECTD4 | MPRIP |  |  |
| PHF10 | HEPACAM | KRTAP11-1 | GPX1 |  |  |
| DNAH3 | LINC00477 | PTPRN | AIF1L |  |  |
| SP3 | ATRX | LHFPL5 | PTRH1 |  |  |
| OIP5-AS1 | MGAT5B | DLX2 | STK25 |  |  |
| CLN8 | TONSL | CPLX1 | GRN |  |  |
| CACNA2D1 | OSGIN1 | CCDC134 | SLC35E4 |  |  |
| SDHAP2 | GBP5 | MUM1 | GATA2 |  |  |
| SELO | PNMA3 | CBFA2T3 | FUNDC2P2 |  |  |
| MBOAT2 | FLJ40536 | IL6 | POP5 |  |  |
| MST1 | MSI1 | ACPT | RFT1 |  |  |
| DKK2 | GJD4 | BCL2A1 | SNRNP40 |  |  |
| KIAA1644 | GOT1L1 | TNNT3 | CNOT7 |  |  |
| PRR22 | ALPP | MUC4 | TRAP1 |  |  |
| RORB | RUNX2 | GOLGA6A | OVCA2 |  |  |
| RGL3 | LOC644852 | KIF13A | ZFR |  |  |
| MAGI2-AS3 | PLEK2 | LYZL1 | PPM1A |  |  |
| FAM110C | TMEM30B | PCDHB18P | PDE12 |  |  |
| ESRRB | GK | SECTM1 | TMED2 |  |  |
| PIGB | NCF1 | RCC1 | ZADH2 |  |  |
| TLE1 | LRRC20 | CSMD2 | DBN1 |  |  |
| FAM193A | C14orf177 | SNX21 | SAMM50 |  |  |
| TLR6 | ZDHHC13 | PTOV1-AS2 | XXYLT1 |  |  |
| TUBD1 | SLC27A6 | OXER1 | KLF13 |  |  |
| POM121 | NRTN | ABTB1 | PAK2 |  |  |
| PLN | DRD2 | OR1S2 | GRPEL1 |  |  |
| WDR86-AS1 | CCDC65 | SH2B2 | BCL2L12 |  |  |
| PAN2 | SH3BGR | PRKCG | PRKRIP1 |  |  |
| GCH1 | PLEKHG6 | FBXL14 | FAM207A |  |  |
| LOC730183 | OIP5 | SNRNP48 | EXOC5 |  |  |
| KIAA1456 | SLC22A11 | ARHGEF39 | PSMB9 |  |  |
| TRAPPC11 | KDM5D | TAPBP | SNF8 |  |  |
| TATDN3 | CHD2 | SHOX | POTEKP |  |  |
| PROSER2-AS1 | LGALS7B | DLG4 | GREM2 |  |  |
| DISC1 | ZFR2 | XKR4 | AGPS |  |  |
| ZNF430 | C4orf17 | DENND6B | MARCH6 |  |  |
| KCNB1 | AP3S2 | STAT5B | ZKSCAN1 |  |  |
| CCDC149 | KRTAP11-1 | MYEOV | KHSRP |  |  |
| ZC3H11A | IGLJ3 | TNS1 | PAF1 |  |  |
| BHMT | CT47B1 | LOC143286 | C1orf123 |  |  |
| SETX | LOC157740 | CFAP57 | ABHD17A |  |  |
| ZNF197 | CCRL2 | SORCS3 | HMGN2P46 |  |  |
| PARGP1 | F2 | ENTPD8 | QARS |  |  |
| FAM184B | OR52K2 | CHI3L1 | SLC2A4RG |  |  |
| SGSM2 | TBX21 | FBXL7 | RABAC1 |  |  |
| ARIH1 | TNFRSF10D | GIPR | TMCC1 |  |  |
| ANKFN1 | LINC00574 | SPAG9 | NPTN |  |  |
| NOS1AP | XAB2 | DZIP1L | DYNC1LI2 |  |  |
| GDI1 | CSH1 | CACTIN | HRH1 |  |  |
| SMA5 | C19orf25 | KIR2DL5A | WDR74 |  |  |
| LOC286191 | XYLB | ENOX1 | TNFRSF12A |  |  |
| PARP11 | FAM71B | LOC643406 | TRAPPC2L |  |  |
| ERC1 | TMPRSS3 | DOK2 | CREB3 |  |  |
| PPARGC1B | ST6GALNAC3 | MRE11A | TMC4 |  |  |
| BTN3A1 | MCM3 | SCAMP4 | SSBP4 |  |  |
| CGN | CSN1S2AP | DMD | MRPS18A |  |  |
| LOC101927178 | FADS2 | ST3GAL2 | PLEKHJ1 |  |  |
| CCDC191 | HES4 | DRD2 | SNRNP25 |  |  |
| NRP1 | CHID1 | NDUFAF4 | RHOA |  |  |
| PGM5-AS1 | FAM155A | GCGR | PPP1R37 |  |  |
| ABCA5 | MON1B | AQP8 | EXT2 |  |  |
| CAMSAP1 | TES | ZMYND15 | TBL1Y |  |  |
| PNPLA2 | KLHL21 | HNF4A | LGALS3 |  |  |
| TAL1 | ARMC6 | UBL4A | MSH3 |  |  |
| CCDC68 | FEV | HIVEP3 | CCT3 |  |  |
| AMT | SGO1 | C16orf52 | ZNF92 |  |  |
| STEAP2 | GPI | RGMB-AS1 | MAP2K3 |  |  |
| DCAF1 | HIST1H3F | HCG8 | KMT2A |  |  |
| CEP290 | CKMT1A | ARHGAP30 | STX16 |  |  |
| ZNF506 | TWSG1 | KRT13 | RAX2 |  |  |
| TF | JAK3 | PHLDB2 | STARD7 |  |  |
| NUP153 | LOC105371763 | MYOM2 | ZNF746 |  |  |
| DENND3 | ICOS | TSEN15 | PLA1A |  |  |
| GOLT1B | OPCML | KRT3 | WWC1 |  |  |
| CEP57L1 | GRAMD2 | GPR65 | LIFR |  |  |
| TBC1D22B | GPR173 | C7 | TMBIM6 |  |  |
| PSMD11 | GRHL3 | PFDN2 | MGLL |  |  |
| ZNF665 | WDR62 | NPY5R | LRRC58 |  |  |
| GLCCI1 | TBX5-AS1 | MAPK3 | DYRK4 |  |  |
| ZC3H15 | CDCA5 | CHRNA5 | MRPS33 |  |  |
| NEIL1 | HMGA2 | EIF4E3 | TOR4A |  |  |
| RNF216 | PLA1A | GAB2 | TUBB4B |  |  |
| C19orf53 | LRRC42 | CEP44 | YTHDC2 |  |  |
| ZNF429 | DAZ2 | PRKAR1B | TADA3 |  |  |
| PYGM | CT45A5 | RASA2 | SORD |  |  |
| SLIT3 | SNCG | ARHGEF15 | LRRC45 |  |  |
| DAPK2 | HAR1A | TMCC3 | NPM3 |  |  |
| SPNS1 | NUDT3 | PHC2 | BDP1 |  |  |
| LINC01002 | SNTB1 | OR10P1 | NTHL1 |  |  |
| MAP3K13 | OR4D2 | FLJ31356 | MXD4 |  |  |
| ZNF564 | EFCAB12 | KRT9 | C14orf37 |  |  |
| MLX | THEM5 | HGF | LRFN4 |  |  |
| NOTCH2NL | PNMA6A | CDHR5 | PNPLA8 |  |  |
| STK36 | CCR3 | PIAS3 | BFAR |  |  |
| PRL | ZNF608 | WFDC10B | AGGF1 |  |  |
| IMPAD1 | EYA2 | ZNF423 | RNH1 |  |  |
| RBM14 | PRO2958 | LINC00526 | SLC34A2 |  |  |
| UBXN11 | HRH2 | OR4A15 | FANCM |  |  |
| LOC100506472 | SLC31A1 | C2CD4B | DHX9 |  |  |
| ACTG2 | ZAP70 | DNAJC27 | SLF2 |  |  |
| NAA16 | CDCP2 | CELA2A | LONP1 |  |  |
| ZNF486 | CHADL | PAFAH2 | LOC93622 |  |  |
| SERHL2 | NGEF | PINK1-AS | CGNL1 |  |  |
| NDUFAF5 | NPHS1 | ADGRF3 | FAM192A |  |  |
| ABCE1 | RASSF2 | DNM1P46 | TUBGCP2 |  |  |
| CAMK2G | DOK3 | KLF6 | WDR13 |  |  |
| VSTM4 | C5orf66-AS2 | KLK2 | RN7SL737P |  |  |
| HTATIP2 | HBA2 | KIF1A | PAPD5 |  |  |
| VCPKMT | TSACC | CARD14 | RBM4 |  |  |
| SEMA6D | NXF5 | C6orf25 | ARHGAP28 |  |  |
| MORN4 | NUP93 | FRZB | SPECC1L |  |  |
| EPM2A | ZBTB33 | NPY4R | ASB13 |  |  |
| DMD | ERICH6B | GCFC2 | ZC3H6 |  |  |
| DOCK7 | OTOF | SLC22A7 | AMBRA1 |  |  |
| HSD17B11 | PFN1P2 | PRF1 | BAX |  |  |
| ITPR1 | SERPINH1 | SLC22A1 | NR2C2AP |  |  |
| SMTN | LILRA2 | LOC729815 | RBFOX2 |  |  |
| ZNF442 | FAM187B | KIAA1656 | CPSF3L |  |  |
| ACVR1C | WASH7P | MOS | RPL23AP53 |  |  |
| C8orf37 | SDF2L1 | SERTAD4 | RPSAP52 |  |  |
| ZBED9 | RBKS | ZNF142 | ALMS1 |  |  |
| FERMT2 | HGC6.3 | FAM181A | HCN2 |  |  |
| PIK3R1 | RLN3 | RSG1 | ATG101 |  |  |
| PMS1 | FBXO15 | GPR142 | PDLIM4 |  |  |
| PAPD4 | SLC25A31 | PIPOX | EHD4 |  |  |
| S100A1 | LOC101060524 | POU6F2 | ASPHD1 |  |  |
| SON | FNDC8 | HRASLS5 | LOC100130331 |  |  |
| CEP162 | RAET1K | CCDC166 | TMEM9 |  |  |
| CHST7 | BPESC1 | GOLGA8IP | ABCF1 |  |  |
| POU6F1 | ULBP2 | ADM5 | BMP8B |  |  |
| LRP1B | FBLIM1 | CTRC | DOK4 |  |  |
| GPR37 | CNIH3 | FAM104A | HLA-E |  |  |
| PPP1R12A | 1-Dec | DUSP2 | AGPAT2 |  |  |
| VCX | MYC | SLC25A45 | C19orf25 |  |  |
| NR3C1 | LOC100291666 | FOXL1 | DDX49 |  |  |
| APOL6 | KCNG2 | AMER3 | UQCC3 |  |  |
| USB1 | CHST15 | GLRA3 | CAP2 |  |  |
| TRAPPC8 | FBXO17 | IKZF4 | CSNK1E |  |  |
| FAM13C | ETS1 | CLPS | TKT |  |  |
| ASMTL-AS1 | CDC45 | ETV1 | TLK2 |  |  |
| KMT5A | CCDC113 | STXBP5 | ZDHHC3 |  |  |
| UBE2B | QPRT | SLC36A1 | HIST1H1A |  |  |
| ZFX | COX6B2 | PADI2 | CBY1 |  |  |
| RAD23B | SOX3 | CNGB1 | RBX1 |  |  |
| SHTN1 | NKAIN2 | LINC01095 | PPRC1 |  |  |
| SLC10A7 | LRRC8B | FPR2 | SF3B5 |  |  |
| PTPN1 | UTY | DMRTC1B | ELOF1 |  |  |
| ZNF732 | HOXC13-AS | TLR8 | ANXA5 |  |  |
| LOC105379426 | PAX3 | CCDC90B | KIF21A |  |  |
| SYDE2 | DEFB103B | POM121L8P | KRI1 |  |  |
| SNHG6 | CSNK1G2 | TBL1XR1 | SULT1A4 |  |  |
| CDCP1 | TSPAN14 | LOC100130924 | CTCF |  |  |
| ARL17A | CRELD2 | SETMAR | PDAP1 |  |  |
| MKL1 | WFDC8 | TPK1 | HOXA3 |  |  |
| IKZF5 | OR8G2 | ABCC6 | WNT11 |  |  |
| ADIPOR2 | PWAR5 | MEGF6 | HIST2H2AB |  |  |
| DNHD1 | EPHX4 | PITX3 | DNAJB2 |  |  |
| CAMKK1 | TNXB | OR1D2 | NDUFA9 |  |  |
| LACC1 | LGALSL | KLHL6 | CYC1 |  |  |
| C1orf132 | HJURP | DUX3 | YIF1A |  |  |
| RBM6 | GSTA3 | ANKAR | PDPK1 |  |  |
| RNF148 | CMTM2 | ASB10 | PRDM1 |  |  |
| FXYD5 | LOC400794 | MNX1 | POMGNT1 |  |  |
| UBE2D2 | VSTM2A | MROH6 | MED29 |  |  |
| ACKR4 | ALOXE3 | VEGFA | ITPA |  |  |
| ULK4P1 | CDH3 | MALAT1 | PI4KB |  |  |
| ZBTB20 | RAB38 | MTMR11 | ZBTB20 |  |  |
| ID2 | GS1-124K5.11 | LILRA4 | ANKRD36BP2 |  |  |
| OSGIN2 | SPA17 | MAMDC4 | ATF2 |  |  |
| STX16 | KRT75 | OR6Y1 | PLPP1 |  |  |
| PDPR | FBRSL1 | RBFOX1 | SMARCA2 |  |  |
| C3orf70 | RBBP4 | BRINP3 | GRK3 |  |  |
| TBC1D23 | NKX3-1 | IGSF3 | CSPP1 |  |  |
| SLC2A12 | UCHL1 | ASPG | CRIP2 |  |  |
| ZDHHC6 | ELMO2 | MSR1 | TRIM25 |  |  |
| SETD4 | PPP2R2B | SCN4B | U2AF1 |  |  |
| NFIX | MEST | FNDC11 | APH1A |  |  |
| MLLT10 | LRFN2 | LOC441666 | GALM |  |  |
| CBLB | FAM41C | LOC107161159 | TAF3 |  |  |
| MACROD2 | LOC101927305 | TRABD2A | WIPF2 |  |  |
| SHF | EVA1B | GATA6 | SF1 |  |  |
| ZBTB26 | COL22A1 | PGA3 | TXNDC5 |  |  |
| TSC2 | MAP1S | CGB2 | GDE1 |  |  |
| ATP11B | LOC101928651 | GLP2R | IL1RAP |  |  |
| EWSR1 | KHSRP | ARHGEF1 | PLXNB2 |  |  |
| RHOU | KRT1 | PRCD | TRIM16L |  |  |
| LOC55338 | HSPA5 | USP30 | PCOLCE |  |  |
| TM9SF3 | C9orf24 | TAF7L | EPHB4 |  |  |
| DNAJB14 | ANHX | KDM4C | TMEM261 |  |  |
| ZSCAN18 | CDH24 | SEPN1 | NSD1 |  |  |
| C19orf12 | EBPL | FZD10-AS1 | PEG3 |  |  |
| TXNRD2 | CD3E | ADAP1 | CDC20 |  |  |
| CRHBP | B9D1 | HSFX1 | RPL10A |  |  |
| ITGB1BP2 | WNT4 | LOC105370027 | SNAI1 |  |  |
| THUMPD2 | LOC105372645 | ALG12 | SLC25A5 |  |  |
| POLR3E | ARPIN | LINC00346 | BCAM |  |  |
| HSF4 | PELP1 | LBX2 | CIRBP |  |  |
| RAMP2 | HS6ST2 | TBC1D26 | HOOK1 |  |  |
| TLE2 | SGCZ | RNF157 | CELSR1 |  |  |
| ZFP28 | PHACTR1 | SLITRK1 | EXOC3L2 |  |  |
| BAGE4 | SCGB3A1 | GSDMA | FAF1 |  |  |
| ZZZ3 | TRIM13 | TCTE3 | HMGN2 |  |  |
| SLX1A | CD209 | SPEF2 | PHOX2A |  |  |
| GNLY | CYB561D1 | ZDHHC22 | PRMT5 |  |  |
| KIF16B | ICOSLG | PP14571 | RAE1 |  |  |
| SRGAP1 | SPCS2 | SPATA32 | NFATC2IP |  |  |
| ENOX2 | SPERT | TBC1D27 | ZNF451 |  |  |
| SETDB2 | SERPINB8 | XIRP1 | GGT6 |  |  |
| SNX9 | HLA-DOA | MDM4 | AARS |  |  |
| NAPG | CXCR6 | ZMIZ2 | EIF2S1 |  |  |
| CCDC50 | FBN3 | FAM83F | MYH9 |  |  |
| SNHG12 | TLL2 | FAM78B | RPS19BP1 |  |  |
| BIRC2 | GPR27 | LOC100129648 | IFITM4P |  |  |
| UBA6 | OPTC | PDCD1 | RUNX1T1 |  |  |
| ACSF3 | RGL4 | CYB561D1 | PSMB5 |  |  |
| MRPL30 | HCRTR1 | KIR2DS2 | PHF13 |  |  |
| LOC100507131 | MAGEA9 | NIM1K | TNIP2 |  |  |
| LDLRAD4 | LECT2 | FAM201A | SARNP |  |  |
| TRIO | MYOD1 | CCDC85A | MCM5 |  |  |
| ZNF347 | FLG | CCR3 | MUM1 |  |  |
| RRN3P3 | SOX1 | PF4 | P4HTM |  |  |
| FAM156A | LOC284933 | CPNE5 | COL6A1 |  |  |
| PDZRN3 | USH1C | RIN3 | ZNF660 |  |  |
| ATXN7L1 | MLF1 | LINC00622 | GXYLT2 |  |  |
| EPC1 | GJA1 | VPREB1 | HPGD |  |  |
| KCTD2 | KNCN | KIAA1161 | ZNF414 |  |  |
| KCTD10 | CD244 | AANAT | FZD2 |  |  |
| INTS6L | KRT17P5 | BHLHA15 | ATP5B |  |  |
| OSER1-AS1 | PDS5B | PRORSD1P | VDAC3 |  |  |
| DIO3 | KRT37 | MUSTN1 | APEX1 |  |  |
| NPIPB3 | KIRREL3-AS3 | SYT2 | ARFGAP1 |  |  |
| YTHDC1 | BRINP2 | RCSD1 | PATJ |  |  |
| UVRAG | LOC101929622 | SLC22A17 | SEPT2 |  |  |
| RXFP1 | LINC00839 | PLCH2 | DEAF1 |  |  |
| CCDC142 | CORO7 | CROCC | UBE2W |  |  |
| PHIP | UBD | PSORS1C1 | MAP4K4 |  |  |
| KLF3-AS1 | NRN1 | CACNG8 | MRPL14 |  |  |
| KIAA1033 | TRIB3 | LOC101926935 | PRELID2 |  |  |
| GPBAR1 | PBLD | LINC00944 | TGFBRAP1 |  |  |
| MAP9 | SLC27A5 | IP6K1 | BTN2A3P |  |  |
| ZNF121 | SIX3 | SMARCC2 | GLUD1 |  |  |
| SLC30A4 | MYBPC3 | TXK | RPL21P44 |  |  |
| DENND1A | ISL2 | SIPA1L1 | SLC11A2 |  |  |
| QRSL1 | ORM2 | LAMA1 | GSR |  |  |
| IGSF11 | B3GNT6 | MYH3 | THUMPD1 |  |  |
| CDK5RAP3 | LPCAT1 | SBK1 | SETX |  |  |
| HNMT | DUSP21 | SOWAHA | RASA2 |  |  |
| PRELID2 | ORF1 | CAMK1G | MTFP1 |  |  |
| STON1-GTF2A1L | ZMIZ1 | FOXE1 | TUT1 |  |  |
| SERINC5 | LOC101928068 | LINC00900 | RPL13P5 |  |  |
| FAM86B3P | DFFA | SFTPA1 | GADD45GIP1 |  |  |
| SHPRH | ATP2B3 | ANKHD1 | HP1BP3 |  |  |
| DNASE2B | SFRP5 | SFXN3 | HIST1H1C |  |  |
| MAGOHB | LOC100507277 | CD40 | PKD2 |  |  |
| SH3KBP1 | FFAR2 | LGI4 | FAM83B |  |  |
| KCNK17 | RPN1 | LINC00942 | HDGFRP2 |  |  |
| LINC00950 | ACAN | B4GALNT1 | STXBP4 |  |  |
| ATP5I | POM121L10P | SDS | STRA13 |  |  |
| TNRC18 | UBL4A | FAM209A | ZRANB1 |  |  |
| LSS | CD79A | ATP2B3 | PMS2 |  |  |
| ZBTB1 | TAF9B | AQP5 | KLHL20 |  |  |
| CHKA | CARD18 | GLIDR | RUFY2 |  |  |
| KMT2B | OTOR | IZUMO1R | TSR1 |  |  |
| CYBRD1 | C16orf46 | OR2B2 | TARS2 |  |  |
| PWWP2A | FBXL20 | CCDC114 | SLC35E1 |  |  |
| KCNA5 | CBFA2T3 | LINC00839 | RPS10 |  |  |
| LRCH3 | SSC4D | REXO1 | FOXO3 |  |  |
| PGBD1 | RPUSD2 | LOC100128922 | ZNF444 |  |  |
| RBPMS | PSG8 | COX11 | YPEL2 |  |  |
| KAT2A | FGF13 | TNXB | SDC1 |  |  |
| TPCN1 | SYN1 | LY6G5C | SERF2 |  |  |
| NUP188 | HUNK | NFE2 | SFSWAP |  |  |
| KCNG1 | LRP3 | BCL11B | WDR83 |  |  |
| PPP1R3B | POLR2A | IRF2 | NKAPP1 |  |  |
| IDUA | COX6A2 | PRB4 | DIAPH1 |  |  |
| CYP4F8 | ITGAL | SRCIN1 | PMS2P5 |  |  |
| NPAT | TRBV5-4 | EVI5L | ECI1 |  |  |
| GCOM1 | PKLR | CYP17A1 | MPND |  |  |
| TAF8 | NPY2R | MC3R | N4BP2 |  |  |
| RECQL5 | ELFN1 | CEACAM7 | SLC26A7 |  |  |
| ADAMTS5 | DHRS7C | CYP2W1 | LOC100128079 |  |  |
| FRYL | LOC100131864 | ZNF222 | MRPL20 |  |  |
| WHRN | CLEC10A | TAF1C | CMTM4 |  |  |
| ATG2B | C6orf62 | KCNB1 | AAR2 |  |  |
| CCDC66 | MNX1 | FAM184A | TUBB6 |  |  |
| GOLGA6L10 | CCER1 | ABCA13 | CUTA |  |  |
| SP5 | CEACAM8 | SLC1A1 | RRP7A |  |  |
| PSMD5-AS1 | ATP1B4 | CD200R1 | PDDC1 |  |  |
| CA13 | HOXB1 | ADAM20P1 | ZYG11B |  |  |
| SPOCK1 | C11orf72 | RAPGEF6 | ARIH2 |  |  |
| CFL2 | ATXN3L | ZNF181 | GRB2 |  |  |
| SGK1 | CALHM3 | TRMT13 | HTT |  |  |
| CCDC186 | LOC100506571 | TMEM198B | TAOK1 |  |  |
| TTTY14 | ALKBH2 | PGPEP1L | MAGI3 |  |  |
| NPHP1 | C19orf73 | DAAM1 | TUG1 |  |  |
| RBBP6 | ANKRD30A | MLXIP | BCAS3 |  |  |
| ZNF492 | TMED1 | TAS2R5 | TALDO1 |  |  |
| PTPRZ1 | SMO | TTC33 | JUN |  |  |
| SPRYD7 | LINC00643 | MPP3 | IRF2BP2 |  |  |
| GPD1L | PDX1 | PON3 | DTX3L |  |  |
| BHMT2 | CDS2 | RNF215 | KRT18P55 |  |  |
| SOX13 | C6orf47 | TBC1D32 | CHTF18 |  |  |
| SLC38A11 | INO80D | FFAR4 | CDC42BPB |  |  |
| ANKRD23 | TBATA | PDE1B | EPC1 |  |  |
| URB1 | DGKZ | SEMA6D | SMS |  |  |
| ARC | PAEP | ZNF333 | RBM10 |  |  |
| LOC283701 | SNF8 | GALNT15 | TNXB |  |  |
| SPAG8 | MYPOP | ANO8 | ACVR2B |  |  |
| TGFB2 | GM2A | ATG9B | AHCY |  |  |
| TMEM249 | COL27A1 | WFIKKN2 | ALG8 |  |  |
| NPPA | TCF3 | ZNF597 | USP42 |  |  |
| DNAJB12 | HRH3 | ETV3 | MANBAL |  |  |
| FGFR1 | RLBP1 | VPS26B | DTX2P1-UPK3BP1-PMS2P11 |  |  |
| ULK1 | PPP1CA | GPR84 | MAU2 |  |  |
| ZFP2 | RAB3D | SAMD8 | SURF4 |  |  |
| SRGAP3 | CDX2 | SFT2D3 | EBLN3P |  |  |
| LINC01354 | RRP7BP | LOC399851 | BLOC1S5 |  |  |
| PARD3B | EEF1A2 | TNR | TRIM27 |  |  |
| RNMT | ADAM22 | LOC100129455 | HNRNPUL1 |  |  |
| TBRG1 | FOXI3 | NFATC2IP | ULK4 |  |  |
| SNX13 | VNN2 | CNTNAP5 | SLC3A2 |  |  |
| CLK2 | B3GALT5-AS1 | C5orf64 | GMEB1 |  |  |
| LOC100507103 | GNG2 | EMB | COQ4 |  |  |
| ZNF841 | RNASEH2A | FCRL2 | SDK1 |  |  |
| ENY2 | DUSP7 | RFK | TMEM33 |  |  |
| MST1P2 | EIF4E | UNC93B1 | RNF144A |  |  |
| GFOD1 | TTTY23 | ACBD5 | RIPK1 |  |  |
| CYP20A1 | CD300LB | TPTE2P6 | PDZD7 |  |  |
| MICA | HRH4 | KLK9 | ZNF429 |  |  |
| MYPOP | MGAT5 | PYGM | WASL |  |  |
| TMEM79 | LRP12 | MLXIPL | OXLD1 |  |  |
| ARRDC3 | FSIP2 | PGLYRP2 | SMAD1 |  |  |
| DDX46 | PCSK5 | LDB1 | RBM17 |  |  |
| VPS13A | SGSM2 | EEFSEC | ZC3H13 |  |  |
| ACTA1 | TMEM89 | CHRM1 | CDK10 |  |  |
| TMEM106A | SCG5 | WIPF3 | CBL |  |  |
| CALCR | PDIA6 | NCF1 | PHC2 |  |  |
| NOXA1 | LINC01249 | EMCN | P4HB |  |  |
| DGKA | INS-IGF2 | NPB | MOGAT1 |  |  |
| LOC100996345 | DEFA6 | C16orf86 | SLC35E2 |  |  |
| PRSS30P | ASB16 | LOC100132363 | TMEM223 |  |  |
| PRKAB2 | BIRC7 | LEXM | TFB1M |  |  |
| TECTA | CD177 | DHH | DOHH |  |  |
| ZNF292 | SYNE4 | LATS2 | CYP51A1 |  |  |
| IP6K2 | LAGE3 | LOC100132147 | NDUFA13 |  |  |
| MT1A | GGT2 | IER3IP1 | PPP1CA |  |  |
| SLC2A8 | KCNA7 | MED26 | RPS6KA5 |  |  |
| CLK3 | UNG | CHL1-AS1 | ERCC1 |  |  |
| PLD6 | LOC643441 | SCGB2B2 | NDUFAF3 |  |  |
| ZNF84 | SMARCD3 | SFTPD | FEN1 |  |  |
| SOGA1 | NDUFB2-AS1 | MUC2 | CPNE3 |  |  |
| PLCXD3 | DEFT1P | ZP3 | BTF3 |  |  |
| NFIB | SLCO1B7 | MAG | HIST1H2BD |  |  |
| EMC3-AS1 | TTTY9A | LCE1F | PTPN4 |  |  |
| TFE3 | MFAP2 | UGT2A1 | UBE2I |  |  |
| EPT1 | DMRTC2 | CLDN15 | FAM160B1 |  |  |
| SLC24A4 | CNN3 | LRCH2 | RNF157 |  |  |
| GATAD1 | GPHA2 | SIRPB1 | FARSA |  |  |
| ZNF626 | SALL3 | LOC105371184 | THADA |  |  |
| MAP3K2 | POLR3D | EVI5 | MAN1B1 |  |  |
| KIAA0485 | C10orf67 | LINC00965 | PURB |  |  |
| SHROOM1 | ASB4 | CTNNA3 | SPINT2 |  |  |
| RPS6KB1 | MUC2 | DPEP1 | MIGA1 |  |  |
| SPATA33 | UNC93B1 | ICAM5 | RALGAPA2 |  |  |
| RASGRP3 | WHSC1 | KLHL5 | CASP10 |  |  |
| ZNF141 | NCK2 | OR4C15 | FIBP |  |  |
| ICE1 | LINC00690 | FAM53B | PTGER4P2-CDK2AP2P2 |  |  |
| TMEM245 | FDX1L | ING5 | GOLGA7 |  |  |
| CRTC2 | MED12L | NF1 | PARM1 |  |  |
| MAOB | CREB3L3 | UGT3A1 | CHMP1A |  |  |
| LOC286254 | SSBP3 | C1QL1 | COA7 |  |  |
| MRPL53 | WNT10A | RTN4RL1 | CAP1 |  |  |
| PPP2R5A | TP53I13 | SEPT8 | FUT10 |  |  |
| DDI2 | EMX1 | IRX6 | PQBP1 |  |  |
| FAM35A | ACTL7B | ACE2 | TSPAN5 |  |  |
| SOX12 | NAG18 | SDCBP2 | FES |  |  |
| TPM2 | KAAG1 | FCHO1 | SENP3 |  |  |
| PLA2G12A | TMEM65 | LINC00482 | C22orf29 |  |  |
| MYH11 | DLGAP3 | CNDP1 | ADCYAP1R1 |  |  |
| ERICH1 | CD300A | KRT222 | PSME1 |  |  |
| YLPM1 | CBFA2T2 | SASH3 | NGRN |  |  |
| RIF1 | S1PR4 | FCRL5 | UNC119 |  |  |
| HIST1H1E | GTF3C4 | TAGAP | UBL7-AS1 |  |  |
| RGS1 | PTGER4P2-CDK2AP2P2 | CNOT2 | MGAT1 |  |  |
| STIM1 | F7 | ZFR2 | POLD3 |  |  |
| FAM63B | SPP2 | ONECUT2 | ATG13 |  |  |
| DST | ERC2-IT1 | MORN1 | RAB31 |  |  |
| DGKE | SHOX | KCNG4 | EMID1 |  |  |
| CDK10 | IL36RN | FBXL18 | LOC100132831 |  |  |
| RNF115 | ATP10A | DBH | MARK3 |  |  |
| GSTA4 | HOXB7 | ENHO | FOXK2 |  |  |
| KLHDC2 | LOC100131195 | DPH6 | MED9 |  |  |
| ZNF850 | LOC101927948 | COPE | ICA1 |  |  |
| SYT15 | TTL | SLC25A37 | CDC42 |  |  |
| ERCC5 | CCR7 | APOBEC3A | MGST3 |  |  |
| ZNF519 | CD70 | DOCK4 | MSN |  |  |
| CHRNA10 | TRIM62 | IGHV3-48 | NCOA4 |  |  |
| GUF1 | MASP2 | NTRK3 | LOC407835 |  |  |
| ABCB9 | HN1 | OXCT2 | FBXO25 |  |  |
| ARHGEF6 | TUBB | IGLV1-44 | MRPL43 |  |  |
| LAMTOR3 | ACOT7 | BET1L | PSMB2 |  |  |
| RABEP1 | CA6 | PRPH | C11orf68 |  |  |
| QKI | SNRNP25 | IGHG4 | ATPAF1 |  |  |
| RASGRF2 | CAMK4 | PAQR8 | DRD4 |  |  |
| SH3BGRL2 | OVGP1 | VWA5B2 | TTC37 |  |  |
| CBR3 | DEFB4A | TTBK2 | WDR34 |  |  |
| ENTPD4 | HEYL | ANKRD6 | PRPF40A |  |  |
| FAM135A | E2F1 | MROH7 | FAM63A |  |  |
| ICE2 | CD300E | MPPED1 | IRX5 |  |  |
| ZNF7 | TMX1 | KRT14 | ZBTB41 |  |  |
| CCDC144NL | DLAT | NPHS2 | CLPP |  |  |
| FGF2 | FLJ23867 | ATCAY | AHCYL1 |  |  |
| SMC5 | SASH3 | ZNF248 | PMS2P1 |  |  |
| DMGDH | CELF2 | C2orf74 | MAVS |  |  |
| FAM124A | HOXB4 | ELOVL7 | UBR1 |  |  |
| RERG | BFSP2-AS1 | SOHLH2 | FBXW5 |  |  |
| ZNF226 | LGALS16 | CXCL17 | SYNE2 |  |  |
| MFSD8 | ATP2B2-IT2 | CRABP1 | SERP1 |  |  |
| SLC25A43 | ZSCAN10 | ZNF440 | HECTD4 |  |  |
| TBC1D12 | RFX1 | APCS | RYK |  |  |
| MAPT | HMX2 | ELANE | LOC440461 |  |  |
| COL6A1 | ANKRD52 | PCNX3 | VPS13A |  |  |
| LINC00649 | MMD2 | NBPF18P | CAMK2N1 |  |  |
| LOC100132363 | CYP3A5 | FAM47A | GTF2IRD2 |  |  |
| CDC42BPA | MECP2 | ERBB2 | DENND2C |  |  |
| ZNF252P-AS1 | PYM1 | TNK2 | UTP23 |  |  |
| ZMYM1 | APOA4 | IGK | ZDHHC5 |  |  |
| LOC101928433 | ENPP7 | NAV2 | IFITM3 |  |  |
| OTUD3 | YEATS2 | MFSD2A | SUN2 |  |  |
| DUSP3 | HIST1H4J | SLC32A1 | PROSER2 |  |  |
| OR5P1P | TPM4 | PDCD4-AS1 | PDXDC2P |  |  |
| TUBGCP6 | LRRC4B | HRC | UQCRH |  |  |
| SMAD5 | ECT2 | ZNF738 | GANAB |  |  |
| CYP1A2 | SLC6A1 | MAX | AKR1B1 |  |  |
| LOC401433 | HHIPL2 | HVCN1 | OTUD7A |  |  |
| PHTF2 | FLYWCH2 | KDM5D | IMPDH1 |  |  |
| THEM4 | SPRR2D | EFHC2 | MGA |  |  |
| ATP11A | PSIP1 | IGHV3-30 | DDHD1 |  |  |
| CYP26A1 | SPDYE1 | CSH2 | SSU72 |  |  |
| BICD2 | PHF12 | LMAN1L | NFIB |  |  |
| PMS2P1 | AMER2 | LOC400756 | TLCD2 |  |  |
| TTN | RCBTB1 | TRDMT1 | ZNF609 |  |  |
| CDC14C | HSPBP1 | NLRC3 | HYLS1 |  |  |
| MYOF | SLC36A1 | PCDHB2 | TSEN34 |  |  |
| SMA4 | EHD1 | CTBP1 | ISY1 |  |  |
| KLHL42 | SMIM23 | MIR22HG | LOC100133050 |  |  |
| LRRC37A2 | SSU72 | L3MBTL1 | MOGS |  |  |
| PRELP | LINC00326 | TMEM30B | CYB5R3 |  |  |
| C1orf204 | C1orf61 | GNAZ | MEPCE |  |  |
| DCUN1D2 | FAIM2 | CCL3 | MIIP |  |  |
| CPB1 | UST-AS1 | GPR34 | USP7 |  |  |
| FAM35BP | IL17C | MIAT | HPSE2 |  |  |
| RAD54B | CTSS | GLIS1 | NOB1 |  |  |
| RBM28 | PNLIPRP1 | DENND4A | GRIPAP1 |  |  |
| SIK1 | CNTNAP2 | SV2B | DAP |  |  |
| FMNL3 | AGXT | GGN | NDUFA7 |  |  |
| ACPP | IZUMO4 | FOXD2-AS1 | KDELR2 |  |  |
| LTN1 | SCD5 | HERC5 | TPSG1 |  |  |
| ERC2-IT1 | DPP9 | LOC100287704 | PATL1 |  |  |
| SMURF2 | NPIPA1 | GRID1 | KIF13B |  |  |
| ZNF776 | FAM134C | NR5A2 | DLST |  |  |
| PEA15 | AHDC1 | FOXP4 | S100A16 |  |  |
| HCN3 | GCSAML | TBX18 | STX18 |  |  |
| VPS39 | GMCL1 | MBD2 | TSPO |  |  |
| LMLN | PAMR1 | TAS2R19 | CNDP2 |  |  |
| MICAL1 | VDR | PRSS50 | MON1B |  |  |
| FAM114A1 | SH3BP2 | SPRYD7 | SPSB3 |  |  |
| ARHGAP44 | CDKAL1 | PLD4 | TUBGCP3 |  |  |
| SVEP1 | LLGL2 | LILRB4 | NEO1 |  |  |
| LOC101930370 | APOF | HDX | PURA |  |  |
| ODAM | CLPS | LINGO4 | PEF1 |  |  |
| ZNF678 | APOBEC1 | COX4I2 | CEP152 |  |  |
| TRMT2A | NAIF1 | FCGR2A | PDRG1 |  |  |
| MAPRE3 | LINC00337 | OR2A20P | MYLIP |  |  |
| TICRR | SLC2A4RG | PCIF1 | LOC101927550 |  |  |
| RAPGEF4 | ASGR2 | ASAP1-IT2 | SEPT7P2 |  |  |
| SNORD114-3 | ZIC1 | PEX5 | NIP7 |  |  |
| AHNAK | GPR182 | CYP1A2 | CLCN7 |  |  |
| CHKB | APBA1 | SYTL5 | VHL |  |  |
| LINC01554 | MIR7-3HG | NTRK2 | BAD |  |  |
| ACSS2 | SART3 | TOLLIP | SMPD4 |  |  |
| ALS2 | CRISPLD2 | MEP1A | LDLRAD1 |  |  |
| DCAF4 | LBX2 | TRIM10 | RPL5 |  |  |
| DLGAP1 | CASP4 | SCAMP5 | HOXA5 |  |  |
| ALPK1 | KIF5A | SLC6A13 | MKI67 |  |  |
| LINC01590 | RAX | LOC100132790 | P3H3 |  |  |
| ZNF107 | LIPA | DNASE1L2 | RPS6P6 |  |  |
| MAPKBP1 | RNASE1 | LINC01420 | GDAP1 |  |  |
| ZNF43 | TUBA1C | STAT5A | PRTFDC1 |  |  |
| ZNF583 | ITGB7 | USH1C | L3MBTL2 |  |  |
| VILL | R3HDM2 | C5orf52 | GABPA |  |  |
| NDUFA4L2 | PTPRN2 | RNASE7 | LOC643802 |  |  |
| ACSL4 | NCL | PAX6 | STAG2 |  |  |
| SLC25A37 | ETV3L | PGAM5 | ZRANB3 |  |  |
| PLRG1 | KRT80 | FBXL8 | ZNF24 |  |  |
| MFGE8 | ZNF467 | LOC105373942 | RPL13 |  |  |
| ZNF552 | CAPN13 | MLC1 | RAB11FIP2 |  |  |
| PXK | EAPP | SIT1 | AKR7A2 |  |  |
| PRICKLE1 | AGPAT2 | COTL1 | HM13 |  |  |
| ALAD | FMN2 | GUCA1A | ZFAND2B |  |  |
| MORC4 | MYADML2 | C4BPA | MRPS2 |  |  |
| GOLGA6C | LOC100130256 | EXOC2 | DCAF12 |  |  |
| MYOM2 | KDELC1 | ELL | ANKRD18A |  |  |
| UBALD1 | S100A9 | ARHGAP24 | PRDM5 |  |  |
| C15orf65 | NGF | KLKB1 | DDX41 |  |  |
| CEP126 | AQP4 | RAPGEF4 | USP15 |  |  |
| HKR1 | LINC00303 | CARMIL3 | TMEM104 |  |  |
| MACC1 | BMP8B | RBP5 | ANKRD35 |  |  |
| MRPL35 | SCGB3A2 | XAB2 | B3GAT3 |  |  |
| TUBAL3 | FAM64A | NECAB2 | MIS18BP1 |  |  |
| EVL | POU3F4 | ZBTB46-AS1 | ANAPC11 |  |  |
| FGD5 | PRDM9 | CYP24A1 | NUDC |  |  |
| PNN | CFAP46 | SSX2IP | FN3KRP |  |  |
| KLHL20 | CHST10 | ADRB3 | TMEM184B |  |  |
| CSNK2A1 | CTDP1 | TOB2P1 | CD248 |  |  |
| EHBP1 | CHRD | TKTL1 | RNF10 |  |  |
| ROCK2 | TMEM52 | SMURF1 | HIST1H2BI |  |  |
| TRMT10B | SH3BP1 | AXDND1 | INF2 |  |  |
| ZNF41 | CHEK1 | CT55 | GNA11 |  |  |
| PTH1R | ANXA8L1 | GRID2 | RANBP1 |  |  |
| TMEM133 | KCNE5 | STARD10 | BIRC5 |  |  |
| TGFBR3 | SSX7 | TACR2 | ALDH6A1 |  |  |
| LYRM2 | FOXN1 | STXBP1 | TBCB |  |  |
| MCF2L | TERT | MUC5B | TXN |  |  |
| SETD2 | CRISPLD1 | CNIH2 | STIP1 |  |  |
| ABLIM3 | BPIFB2 | FNDC3B | ZNF717 |  |  |
| AVPI1 | ZNF124 | RAD9A | PKP2 |  |  |
| LRRC70 | NFKB2 | ZDHHC21 | ACTR1B |  |  |
| SEPSECS | GRM8 | ITGA5 | NSUN5 |  |  |
| COG3 | TP53AIP1 | PRKACB | QPRT |  |  |
| LINC00597 | CACNB3 | MREG | C10orf2 |  |  |
| FIGF | MYPN | C4orf32 | NOC2L |  |  |
| RAB1B | PAG1 | HOXB13 | DDX3X |  |  |
| PPFIBP1 | ZIC4 | NOP14-AS1 | UBXN1 |  |  |
| PFN4 | AKR1D1 | EPN1 | KIF3A |  |  |
| APLF | LINGO4 | CDADC1 | SYT11 |  |  |
| CASC1 | LOC645984 | HIF3A | HIPK2 |  |  |
| HMGN1 | RAB8B | KCNK16 | MRPL53 |  |  |
| STON1 | SLC16A12 | TBC1D2 | ZNF318 |  |  |
| TMEM132C | F12 | TSHB | GAA |  |  |
| VAMP2 | LINC00052 | VIP | MCM4 |  |  |
| FLT1 | ADD2 | ATXN7L1 | HLA-J |  |  |
| MTERF2 | SUMO2 | CLU | GPATCH1 |  |  |
| ITGA11 | LRIT2 | AQP10 | TRAM2 |  |  |
| TSHZ2 | PLIN3 | GOLGA8F | ROCK1 |  |  |
| HORMAD1 | TNC | GABARAPL3 | CDC34 |  |  |
| EPO | PRR21 | TRIM62 | TMBIM1 |  |  |
| CEP95 | AKNAD1 | TNFSF9 | SAP30BP |  |  |
| LRIG3 | FLJ36000 | GPR155 | SLC25A39 |  |  |
| BCL6 | GRK3 | GH2 | OGG1 |  |  |
| CUL3 | CCDC96 | ARHGAP18 | CLK3 |  |  |
| CLIP2 | DEFB126 | HSD3B7 | UBE2Q2P1 |  |  |
| TMEM69 | LINC00939 | DPP10-AS1 | SART3 |  |  |
| RBPMS2 | FUBP1 | ANKDD1A | RCN1 |  |  |
| SUPT7L | GUSBP10 | ZSCAN2 | A4GALT |  |  |
| GRIPAP1 | TCOF1 | MYO15B | MIEF1 |  |  |
| FAM193B | SPTB | KIDINS220 | NXF1 |  |  |
| URM1 | GNAZ | CHRNA1 | TMEM141 |  |  |
| NAA60 | COL2A1 | MCM9 | NECTIN1 |  |  |
| ALB | SFTA3 | RERG | SHMT2 |  |  |
| SAMD9 | BHLHE23 | LOC107985946 | HARS |  |  |
| MAP4 | UGDH | SZT2 | ATF5 |  |  |
| ZBTB11 | ZIC5 | BOD1L1 | ATP1A3 |  |  |
| PPP1R14A | TMEM98 | DAPP1 | FAM21A |  |  |
| NR2F2-AS1 | FEZ2 | PTGS2 | DCAF11 |  |  |
| PGGT1B | LY6D | ERICH6 | GOSR1 |  |  |
| PTPRR | PRO1596 | PCYT2 | PSMD8 |  |  |
| HACD1 | MUC12 | EPOR | BRD3 |  |  |
| CCNT1 | FGF2 | LOC100507537 | CCDC65 |  |  |
| PKN2 | LOC647115 | LOC101927770 | RPP25L |  |  |
| SFTA2 | MR1 | TRMT61A | TERF1 |  |  |
| TMUB2 | FAM212A | ANKRD40 | PPP1R12A |  |  |
| CDHR3 | TCHH | CPB1 | CTBP2 |  |  |
| KDM5C | MEFV | DNAH3 | STK40 |  |  |
| PCDH20 | TRPS1 | GHRHR | CAMLG |  |  |
| SNX19 | ANKRD24 | FRA10AC1 | RPL41 |  |  |
| HERC6 | ASTN1 | SPATC1L | HERPUD2 |  |  |
| STARD9 | MAF | LBX2-AS1 | OPLAH |  |  |
| METTL7A | GTPBP6 | CCDC30 | LINC01023 |  |  |
| HAVCR2 | GIPR | SLC52A1 | SMARCA4 |  |  |
| DKFZp547J222 | ACPT | PLN | MPST |  |  |
| HAND2 | RBMY1B | DRC7 | ARHGEF39 |  |  |
| PRTG | KRTAP12-2 | MUC3 | ARPC1A |  |  |
| INSIG1 | LILRA4 | CXCR2P1 | SDF2L1 |  |  |
| GRAMD1C | CMTM6 | PLEK | LATS2 |  |  |
| MIB2 | SLC22A18 | ME1 | ENKD1 |  |  |
| ZNF234 | ZNF557 | ZNF395 | PREPL |  |  |
| ZNF561 | MIR663AHG | KRTAP19-1 | ERGIC2 |  |  |
| KIR3DL2 | ZP4 | TH | HIST1H3J |  |  |
| SERTAD2 | SOWAHD | NPBWR2 | MRPL11 |  |  |
| B4GALT5 | EPN3 | GATAD2B | MAN2B1 |  |  |
| ANKRD46 | C1orf229 | DAPK2 | AREL1 |  |  |
| STOM | FOXS1 | PCDHGC5 | MAD2L2 |  |  |
| FAM41C | NMU | PNMAL2 | FAH |  |  |
| HSD17B7P2 | PRCAT47 | C14orf79 | FAM234A |  |  |
| ERICH6 | BTN2A3P | FAM43A | SPIN1 |  |  |
| CARD8 | PON1 | DMGDH | VPS72 |  |  |
| CYLD | OLFML2A | CNTROB | CDK13 |  |  |
| ZFP14 | MANF | OR8H1 | HIST1H2BB |  |  |
| MYO9B | CALY | RIMS4 | IQUB |  |  |
| KIAA0232 | KLK9 | SLC17A7 | EWSR1 |  |  |
| DNMBP | MSX2P1 | TKTL2 | ACTR2 |  |  |
| GNGT1 | AMMECR1 | PTGR2 | CCDC15 |  |  |
| LOC100131929 | NUTM1 | PIK3AP1 | STK19 |  |  |
| LARP4 | SLC46A1 | TLE6 | RSF1 |  |  |
| REV1 | KLK14 | CLDN11 | B4GALT3 |  |  |
| C2CD5 | IQCF1 | PRAP1 | DNAJC30 |  |  |
| ING5 | EXOC3L4 | TMEM47 | PUM1 |  |  |
| KIAA0368 | GATA1 | AMBN | NAV1 |  |  |
| ZBTB43 | WDR70 | LOC100288798 | HIST1H2BH |  |  |
| HPS5 | GLRA2 | EPHA10 | OGFOD3 |  |  |
| ZNF580 | CTRB1 | RAB29 | LRCOL1 |  |  |
| RBM39 | BIRC8 | RNF207 | CRMP1 |  |  |
| NUP58 | RNF24 | LOC100233156 | CTNNBIP1 |  |  |
| SEC31B | C1orf94 | XAF1 | POLR3A |  |  |
| ZNF181 | ERVK13-1 | IRX2 | LRP12 |  |  |
| IFIT2 | ZBTB34 | MOBP | FLYWCH2 |  |  |
| ORAOV1 | ZNF629 | SEMA4B | PTP4A2 |  |  |
| SAP18 | OTUD6A | DUSP16 | FDPSP2 |  |  |
| TRIM33 | CD300LD | BRF1 | VEZF1 |  |  |
| SETD5 | KCNJ11 | SPSB3 | WNT3 |  |  |
| TMEM179B | LRP2 | LIN9 | MFSD12 |  |  |
| MEIS2 | IQCF5 | USB1 | SNU13 |  |  |
| NF1 | ARMC3 | CABP4 | TWISTNB |  |  |
| ZNF621 | KRT79 | TRIM74 | CHADL |  |  |
| ZNF566 | LOX | ITGB2 | PSMA5 |  |  |
| MAN2B2 | KAZALD1 | SPAG5-AS1 | POTEM |  |  |
| FAM198A | CHRM1 | HAMP | RNASET2 |  |  |
| LINC00545 | DCBLD2 | GUCY1A3 | KIAA0141 |  |  |
| EGFL7 | DOCK8 | KLF17 | SLC19A1 |  |  |
| LINC00944 | SLC52A3 | ZDHHC8 | GPAT2 |  |  |
| TCF21 | OR6M1 | GPR31 | UHMK1 |  |  |
| SOX7 | XKRY2 | USP27X-AS1 | ACOX1 |  |  |
| AIMP1 | CHST14 | FOXR1 | GPER1 |  |  |
| KMT5C | KCNJ4 | TICAM1 | C19orf73 |  |  |
| MASP1 | PCYT1B | PRDM15 | CEBPG |  |  |
| SDCCAG8 | IGHG4 | TAF5 | ZER1 |  |  |
| ZP3 | KDELR2 | UNKL | CITED4 |  |  |
| WNK1 | ZWINT | ZBTB24 | IMP3 |  |  |
| ZNF426 | CACNG4 | CD3EAP | STAP2 |  |  |
| SAMD4A | LOC100505841 | HCAR2 | SUGT1 |  |  |
| ZNF727 | GALNT5 | MEGF10 | PLRG1 |  |  |
| DTWD1 | LCE2D | DBF4B | SSNA1 |  |  |
| DISP1 | GPR83 | PLA2G4F | SUN1 |  |  |
| SCIMP | DCAF4 | USP25 | EPS8L1 |  |  |
| ZNF441 | GAPDHS | LRFN2 | CACNG6 |  |  |
| ROCK1 | SLC1A3 | MROH1 | PRR14 |  |  |
| PRDM2 | EYS | B3GNT7 | ATP5G1 |  |  |
| APPL1 | ZAR1 | LOC257396 | NPLOC4 |  |  |
| CBWD5 | CFAP57 | TERF1 | ZNF486 |  |  |
| RAD51-AS1 | LYPD8 | HESX1 | S1PR2 |  |  |
| LOC400684 | SOHLH1 | EGR2 | NDUFS6 |  |  |
| CRIP2 | LINC00442 | FAM46D | HIST1H2BC |  |  |
| ZNF782 | SNCA | RPAP3 | PCBP2 |  |  |
| WSB1 | TAAR5 | DUSP4 | BCAP29 |  |  |
| GPALPP1 | AJUBA | NKAIN4 | COL5A2 |  |  |
| C14orf79 | CDC20B | FOXJ1 | DNPEP |  |  |
| DAPK1 | ITGA2 | GGT8P | VAMP8 |  |  |
| ABHD14B | GPR149 | KRTAP3-1 | DHX30 |  |  |
| LINC01279 | IGK | IKZF3 | HIST3H2BB |  |  |
| NANOG | LINC01109 | MCEMP1 | LAMTOR2 |  |  |
| SOX10 | PRMT1 | OBSCN | AGO2 |  |  |
| PGM5 | GPSM3 | ACBD7 | TRMT13 |  |  |
| RALGPS1 | LINC00403 | HIPK1-AS1 | RNMT |  |  |
| DNM3OS | ZNF367 | LPCAT1 | BCL2L2 |  |  |
| MCL1 | GRAP | SEPSECS | WDR18 |  |  |
| C15orf52 | TEAD2 | CORT | SNX1 |  |  |
| FTSJ3 | DMRTB1 | ZCCHC4 | CDC25A |  |  |
| CMIP | TSGA10 | OR4F4 | LOC101928837 |  |  |
| PVR | PFN2 | ADAMTS17 | CLTB |  |  |
| ZNF808 | OTOL1 | CCNT2 | PYCARD |  |  |
| EML3 | PTMA | KCNE2 | HSPB1 |  |  |
| CDK13 | TMEM105 | PPIEL | ODC1 |  |  |
| LCMT1-AS2 | ATP2C1 | TEX37 | MAP7D3 |  |  |
| SMAD7 | CENPVL2 | KNDC1 | TMED3 |  |  |
| EPB41L5 | ZFP69B | MMEL1 | SIPA1L1 |  |  |
| C14orf178 | SLC6A15 | CDKL3 | HIST1H2BM |  |  |
| ZNF570 | FOXL1 | KCP | EIF3L |  |  |
| THAP6 | ADGRG1 | BCAR4 | PHBP19 |  |  |
| MTMR9 | WDR76 | STRN | LSM7 |  |  |
| NLRP5 | NPSA | FSCN3 | SEC14L1 |  |  |
| C11orf63 | CA12 | C8orf48 | TBCC |  |  |
| HSPB3 | WNT8A | BBIP1 | OXA1L |  |  |
| CREB1 | BORCS8-MEF2B | GRB7 | P2RY1 |  |  |
| HYAL1 | LOC105378499 | HAPLN3 | RPL7 |  |  |
| COL4A5 | ALS2CL | IGFBPL1 | NOMO1 |  |  |
| ANKRD20A8P | KRT4 | CYP4Z1 | SSR2 |  |  |
| C14orf159 | ZBED9 | DNAJC5G | RPL36 |  |  |
| KLHL41 | GPHB5 | SFTPC | AGR2 |  |  |
| ENAH | PRSS58 | ECSCR | PTH1R |  |  |
| HFM1 | PAPLN | STK17A | AKR1A1 |  |  |
| TTC8 | F2RL3 | KLK5 | BAG6 |  |  |
| C1orf162 | SEC14L4 | ABHD14B | TWF2 |  |  |
| DCX | RRS1 | ALOX12 | MAPK1IP1L |  |  |
| KCNMA1 | C9orf163 | D2HGDH | CCDC117 |  |  |
| UBE2G2 | KCNK4 | TAS2R9 | NBEAL1 |  |  |
| CTBS | GABRA6 | UBE2Q2L | AUP1 |  |  |
| NUDT12 | NUTF2 | FGFR4 | ILF3 |  |  |
| NUTM2B-AS1 | BARX1 | EFNA2 | CLIP1 |  |  |
| C16orf58 | DHTKD1 | C12orf56 | MNT |  |  |
| FASTKD1 | KIAA1549L | MATN1-AS1 | RAB18 |  |  |
| GPR157 | LCAL1 | KMT5C | PSMC3 |  |  |
| ADAMTS13 | LOC100129648 | RHBG | ST14 |  |  |
| ZNF223 | KIAA0922 | HSF5 | LIG1 |  |  |
| HIP1 | LTK | ADAMTSL5 | BSG |  |  |
| CHODL | PPBP | FOSB | BASP1 |  |  |
| ZDHHC17 | TLX3 | FAM197Y2P | RASAL2 |  |  |
| AUH | GUSB | CA1 | MRPL55 |  |  |
| FER | PLK1 | FAM134A | HSPA2 |  |  |
| PRKX | SLC4A11 | NR2F1-AS1 | FLRT1 |  |  |
| LOC105370109 | PRLHR | CNKSR1 | BBS4 |  |  |
| C10orf88 | PLAC8 | LOC105372881 | LRRC73 |  |  |
| CDC14B | OR12D2 | GPX3 | EIF2AK4 |  |  |
| EDA | RIPPLY1 | LPA | MLX |  |  |
| PPP1R12C | TTTY15 | FAM63A | CCDC22 |  |  |
| IL6 | DCTN1 | MOSPD3 | PACS1 |  |  |
| PGK1 | CCNE1 | MLN | MGME1 |  |  |
| TTC3 | SIK1 | LOC100419920 | NME2 |  |  |
| ZNF135 | GRIN3B | KRTAP9-9 | LRRCC1 |  |  |
| MGEA5 | KIF21B | ATXN3 | IDH3B |  |  |
| EIF4H | HNRNPL | TOX3 | ARID3A |  |  |
| ZNF625 | SLC22A7 | SLC16A11 | AKAP12 |  |  |
| HOOK3 | UBE2U | STAT2 | USP51 |  |  |
| DFNA5 | PRDM8 | ZNF862 | MSL1 |  |  |
| NDEL1 | RAP1GAP2 | CCNB3 | PRDX6 |  |  |
| STBD1 | LRRC15 | ST8SIA4 | FAM96B |  |  |
| ERP27 | EPHA8 | KCNA10 | FBXO41 |  |  |
| ADAM23 | ZDHHC24 | ADARB2-AS1 | RAD23B |  |  |
| DRAIC | LINC00654 | ASIP | CSNK1G2 |  |  |
| SRRM2 | DUSP26 | CSGALNACT2 | SFRP4 |  |  |
| LEMD3 | PRLH | ARHGAP27 | ERICH3 |  |  |
| POLM | RTN4RL2 | F7 | RPS8 |  |  |
| AFTPH | SH2D5 | HCG26 | CDK5RAP3 |  |  |
| ZNF681 | ATP13A5 | MNDA | TRMT2A |  |  |
| SNHG10 | PHPT1 | UCKL1 | LOC221272 |  |  |
| MECOM | DLX2 | TMPRSS13 | RPS2P45 |  |  |
| LOC100128288 | CNFN | ZADH2 | IFT46 |  |  |
| SLFN13 | AIPL1 | PDE4B | SRSF6 |  |  |
| ANKHD1 | DIP2B | DCLK1 | GNL1 |  |  |
| IPO13 | VWA5B1 | NOS1AP | SRA1 |  |  |
| HM13 | IL1A | E2F2 | NR2F6 |  |  |
| COL6A2 | MRGPRF | COL4A3 | TMEM248 |  |  |
| ZNF311 | PXN-AS1 | PNMA6A | MAGOH |  |  |
| XDH | ARID3A | NCF2 | POGZ |  |  |
| ATOH7 | SND1 | ZNF99 | NDUFA2 |  |  |
| TPP1 | FCN2 | ENPP7 | CWF19L1 |  |  |
| LINC00926 | FNDC11 | PABPC1L2B | CERK |  |  |
| EIF3J-AS1 | LINC01105 | COL13A1 | UBTF |  |  |
| NBPF14 | DAG1 | ERC2-IT1 | KIAA0100 |  |  |
| MAPKAPK5-AS1 | PSMD8 | ADM | CPT1A |  |  |
| LOC101929384 | REN | MDM1 | CALD1 |  |  |
| GNE | SYT13 | TNFSF8 | TCF7L1 |  |  |
| EBLN3P | INAFM1 | SOX12 | DKK3 |  |  |
| RBM15 | TAS1R2 | SRPK3 | PARP10 |  |  |
| TRAPPC12 | TEX13A | TNPO2 | TBCCD1 |  |  |
| PTBP2 | S100A2 | AFAP1 | PHRF1 |  |  |
| ZNF654 | MORC1 | AATBC | ATP6V0C |  |  |
| ZNF785 | MMP27 | TMUB2 | ASB1 |  |  |
| ZNF799 | CHCHD5 | PVALB | RPL37A |  |  |
| RGPD5 | CCDC47 | NR1I3 | C6orf132 |  |  |
| PRIMA1 | LOC619427 | DNAJC5B | HIST1H2BO |  |  |
| STXBP4 | SPNS3 | CIB3 | INAFM1 |  |  |
| FUT10 | MRPS34 | ARHGAP1 | PSMD11 |  |  |
| MTHFR | FOXK1 | SMG1P2 | TPX2 |  |  |
| LOC100129312 | MDH1 | ZNF112 | NUCB1 |  |  |
| PTGER3 | EVX1 | DMPK | TMUB2 |  |  |
| UQCRB | LARP1B | ABCA1 | DDT |  |  |
| HNRNPR | CYGB | ZNF614 | ASB3 |  |  |
| TSTD2 | NUSAP1 | LAPTM5 | NEDD8 |  |  |
| CCDC53 | SPATA19 | TBC1D20 | PRDX1 |  |  |
| ZNF251 | BRI3BP | RITA1 | JOSD1 |  |  |
| COL6A5 | POU4F3 | SAP18 | IMPAD1 |  |  |
| CLIP1 | MPZL1 | OR7D4 | SORL1 |  |  |
| C10orf10 | TUBA1A | LOC202181 | NR2C2 |  |  |
| USF3 | KCNV1 | RHBDL3 | ADIPOR1 |  |  |
| NBEAL1 | HLA-E | TNFRSF4 | IPO9 |  |  |
| LOC389834 | CYP4A11 | OLIG3 | RPL28 |  |  |
| ZBTB7B | HIGD1A | SLC7A1 | SNRPF |  |  |
| ZNF154 | PPOX | SPIN3 | HMBOX1 |  |  |
| MMP23B | SLC16A10 | KLHL7 | HIST2H3A |  |  |
| MITF | RUNDC3A | PPM1E | CKAP5 |  |  |
| IFRD1 | ARPP21 | C9orf139 | LOC441455 |  |  |
| LOC441666 | ARHGEF39 | TMEM147-AS1 | EIF3F |  |  |
| USP6 | LOC100130370 | EXD1 | VGLL4 |  |  |
| ZBED8 | DNAJC22 | TRPV1 | LTBR |  |  |
| TCTA | AFF1 | OSCAR | UNC119B |  |  |
| CXCL14 | HAPLN2 | IRX5 | PPHLN1 |  |  |
| CDC73 | BCL2L14 | PROKR1 | GRHPR |  |  |
| GPR135 | SPAG11B | DDX11L2 | ARL5A |  |  |
| OXR1 | PPP1R16B | ITGA9 | DNASE2 |  |  |
| TPRG1 | TRIOBP | TOR1AIP2 | QSER1 |  |  |
| CBX7 | KCNK18 | MON2 | PPM1H |  |  |
| SLC46A3 | KRTAP9-8 | NFE2L3 | RGPD2 |  |  |
| ZNF302 | AGBL3 | LINC01260 | DNAAF5 |  |  |
| COMMD6 | PAPD4 | SRF | TIPRL |  |  |
| FHL1 | CD101 | TAF6L | FAM210B |  |  |
| DCBLD2 | RP1 | TMEM217 | CDS2 |  |  |
| PPP3R1 | SPC24 | HELQ | HLA-DQB1 |  |  |
| DNAH14 | PGA3 | BRSK2 | PALMD |  |  |
| CLEC16A | INSRR | F8 | SCNN1A |  |  |
| ARRDC2 | TFAP2A | C1QL2 | NAV2 |  |  |
| VEGFA | MN1 | LOC101927648 | TRAPPC5 |  |  |
| CRK | ABCC4 | HYAL1 | PGAP3 |  |  |
| NEURL4 | MAP6D1 | ABT1 | RPS15 |  |  |
| IFI44L | HR | LSM11 | CCDC47 |  |  |
| CITED2 | SOX17 | MYOCD | SLC2A8 |  |  |
| ZNF333 | LY86 | UBR4 | RSU1 |  |  |
| AAED1 | GRIN2B | CYP2A7P1 | CIB1 |  |  |
| SYNJ2 | KRT12 | ZXDA | ANO6 |  |  |
| SZRD1 | FGF18 | CBWD5 | VCAN |  |  |
| C18orf21 | IL1F10 | CEP126 | GRWD1 |  |  |
| ATG14 | CENPO | LINC00337 | PBLD |  |  |
| TMEM161B | HSFY2 | PCGF6 | LRPAP1 |  |  |
| PANK3 | PFAS | NEDD4 | PHPT1 |  |  |
| ZNF714 | SMIM12 | SIGLEC9 | ATP6V0E1 |  |  |
| OTUD6B | MMP13 | SRP9 | BABAM1 |  |  |
| PTGS2 | LOC101929998 | MAP1S | MTF1 |  |  |
| FABP5 | TTLL6 | HDLBP | PPP1CB |  |  |
| JUN | KRT38 | CYSLTR1 | DNAJC15 |  |  |
| NR2F1 | CXCL3 | KPNA1 | CMTM6 |  |  |
| SENP5 | KLHDC7B | NEU4 | SH3BP5L |  |  |
| PCGF3 | GJD2 | LOC400043 | ERCC6L2 |  |  |
| LETMD1 | WNT7B | TRIM14 | NAGK |  |  |
| MLXIP | BORCS8 | NAA50 | C12orf57 |  |  |
| LOC100128398 | ANGPTL2 | HOXB3 | POLR3K |  |  |
| GRK3 | TAS2R41 | TAP2 | ECSIT |  |  |
| CHIC1 | SLC1A7 | GPR25 | GTPBP6 |  |  |
| CSNK1G1 | TBC1D20 | SNORA62 | ARL16 |  |  |
| ZNF432 | TSEN34 | KIAA0040 | NHP2 |  |  |
| CTBP1-AS2 | CSRP3 | CD33 | HAND2-AS1 |  |  |
| LLPH-AS1 | COA4 | ACSM2B | MASTL |  |  |
| NOC2L | GAMT | ACKR2 | DZANK1 |  |  |
| EHD2 | FGF3 | RPL23AP82 | FAM177A1 |  |  |
| FAM21A | CDH12 | ADAM21 | FADS2 |  |  |
| ABHD15 | KCNJ9 | CLEC4G | SEC13 |  |  |
| ITGA9 | MFRP | GRM2 | PPIA |  |  |
| WIF1 | MBTPS2 | STAC3 | MB21D1 |  |  |
| GOLGB1 | BATF | LINC01061 | RAP1GAP |  |  |
| PTPN14 | TMEM35A | TBXA2R | CCDC146 |  |  |
| FXR1 | RAB43 | ACP7 | TOR3A |  |  |
| KANK2 | STEAP1 | URB1 | CDK4 |  |  |
| SIGLEC1 | FAM163A | KCNH3 | BMI1 |  |  |
| RC3H2 | TNIP3 | LOC101928035 | MAN2A2 |  |  |
| FAM172A | DACH1 | CCDC91 | CDC37 |  |  |
| KLF7 | AASDH | GUSBP4 | ANKRD36 |  |  |
| HOXA5 | ACTL6B | DUSP8 | HNRNPA2B1 |  |  |
| PIK3C2A | LILRB4 | UBE2O | ACAA1 |  |  |
| RBM25 | ARHGEF18 | UCMA | HCFC1R1 |  |  |
| LY6G5B | OR5AP2 | TRIM41 | RPAIN |  |  |
| AHCTF1 | FAT1 | OR8U1 | CDK2AP1 |  |  |
| TBC1D5 | OLR1 | KDR | CENPM |  |  |
| LRP1 | SFRP4 | AMER2 | LRRC56 |  |  |
| KAT6A | TMEM125 | LCORL | PINK1 |  |  |
| CNIH1 | TRIM59 | OR6M1 | SNRPA |  |  |
| ZFP41 | FZR1 | CLVS1 | MTX1 |  |  |
| SRPRA | ZIM3 | SLC2A3 | APBB1IP |  |  |
| DNAJC21 | SEC14L3 | GAST | FIS1 |  |  |
| SS18L1 | MIER3 | IQCH | FOXJ1 |  |  |
| TAGLN | SOX14 | PAX9 | FADD |  |  |
| GBP3 | FER1L6-AS2 | INTS5 | NENF |  |  |
| NIPBL | TCF20 | ZDHHC17 | FPGS |  |  |
| ZNF565 | OR4D5 | PSG7 | H2AFZ |  |  |
| GEN1 | TGFA | PIBF1 | ANXA2 |  |  |
| ALDH1L2 | DMRTC1 | SYN1 | TRIM14 |  |  |
| MARK4 | CYAT1 | IGFN1 | AK1 |  |  |
| CAPS2 | IGHA1 | SAMD10 | SUGP2 |  |  |
| IGBP1 | DRD5P2 | DUSP26 | BORCS6 |  |  |
| SEPT5 | INS | RANBP3 | CLN6 |  |  |
| PPIG | IGLV3-21 | WWC2 | MXRA5 |  |  |
| PCBP3 | IGH | NEUROD2 | RRP12 |  |  |
| YTHDC2 | IGF2 | SLC26A6 | MYCBP2 |  |  |
| SAMD8 |  | OR2H1 | ENO1 |  |  |
| TAF9B |  | CYP26B1 | MAST2 |  |  |
| SASH1 |  | TRIM49 | MEAF6 |  |  |
| RDH12 |  | TLN1 | CBWD5 |  |  |
| WDR66 |  | RBM26 | FAM81A |  |  |
| ARHGAP29 |  | SPATA9 | POLRMT |  |  |
| NFKBID |  | TMEM176A | ZNF526 |  |  |
| CBFB |  | ARL8A | FOXL2 |  |  |
| ADAMTSL5 |  | HOTAIRM1 | NELFCD |  |  |
| ZNF420 |  | WDR20 | SCAP |  |  |
| ZNF600 |  | ZNF462 | GATS |  |  |
| ZNF559 |  | SLC25A25 | B9D2 |  |  |
| SUPT6H |  | PCM1 | IGHMBP2 |  |  |
| KIAA1586 |  | ATP8B4 | SQSTM1 |  |  |
| MARCH3 |  | SFTPB | PMPCA |  |  |
| LYPLAL1 |  | KRT17P5 | GPANK1 |  |  |
| ZNF93 |  | PGM5-AS1 | ARHGAP26 |  |  |
| ZNF335 |  | GPR61 | A1BG |  |  |
| GGT8P |  | RSPH9 | SDHA |  |  |
| CD46 |  | TSPAN12 | ARHGAP10 |  |  |
| C2orf68 |  | TMIGD2 | MTURN |  |  |
| GTF3C3 |  | KLK14 | MAST4 |  |  |
| ACVRL1 |  | ANK2 | NUDT16L1 |  |  |
| MOGAT1 |  | CPPED1 | CANT1 |  |  |
| KIAA2013 |  | NOL4L | RPRD1B |  |  |
| RCC1L |  | KRT35 | KIAA1586 |  |  |
| FAN1 |  | SYT8 | GLRX5 |  |  |
| ILDR1 |  | RBPMS | MFAP2 |  |  |
| PRKRIP1 |  | ABCD1 | ENOX2 |  |  |
| PYGL |  | BICD1 | LDLR |  |  |
| VPS50 |  | ADAMTS3 | FBXO18 |  |  |
| DNAJC30 |  | CATSPER2 | AK4 |  |  |
| ZNF12 |  | ZBTB47 | CPM |  |  |
| UHMK1 |  | NEBL | DDX52 |  |  |
| HOXB5 |  | ZNF678 | BMPR1B |  |  |
| ACRC |  | LOC100507494 | CCDC91 |  |  |
| SYNJ1 |  | LINC01530 | USP47 |  |  |
| IFNAR1 |  | PPM1L | BSPRY |  |  |
| RBMXL1 |  | LRRTM2 | POC1A |  |  |
| SCRN3 |  | PHF20L1 | SLC31A1 |  |  |
| NBN |  | SERPINB8 | ZNF74 |  |  |
| FLT3LG |  | LOC146880 | SSR4 |  |  |
| PUM2 |  | BTBD19 | PREB |  |  |
| C16orf86 |  | MSX2 | ATF4 |  |  |
| JPX |  | FAM222A-AS1 | EMC6 |  |  |
| NFYC-AS1 |  | ERVW-1 | FAM53B |  |  |
| MCPH1 |  | ANXA13 | HIST1H1E |  |  |
| KHNYN |  | APBA1 | KIAA0825 |  |  |
| TIGD6 |  | NAV3 | ATP8A2 |  |  |
| KIR2DS2 |  | IRX4 | PRPF8 |  |  |
| EMP1 |  | RASA3 | NDUFS8 |  |  |
| KIAA0586 |  | MOV10L1 | AKT2 |  |  |
| MED15 |  | HLX | TMEM107 |  |  |
| GALNT18 |  | LOC100127955 | RBBP4 |  |  |
| CD33 |  | CYP2C9 | KRT18 |  |  |
| PHF8 |  | OR10J5 | POLR1A |  |  |
| TLE4 |  | PROS1 | RAB35 |  |  |
| RAB11FIP1 |  | ITK | CPXM1 |  |  |
| LINC00933 |  | SEL1L | WWTR1 |  |  |
| CREBRF |  | STX6 | SLC26A2 |  |  |
| USP36 |  | KCNAB2 | MDH2 |  |  |
| DPY19L1P1 |  | SGCA | MYL5 |  |  |
| CEP350 |  | METTL22 | FKBP10 |  |  |
| GADD45B |  | ACOX2 | ALDH1A1 |  |  |
| BCL6B |  | TEPP | NPAT |  |  |
| IL15 |  | DCST2 | RPLP1 |  |  |
| LARGE1 |  | HTR3D | GNB2 |  |  |
| LOC389765 |  | FBXW2 | RAB11FIP3 |  |  |
| DBF4B |  | ZFYVE1 | CNNM3 |  |  |
| IRX4 |  | FLJ42627 | SUCLG2 |  |  |
| LOC100132790 |  | C5AR2 | RIPPLY3 |  |  |
| BBS7 |  | GDF10 | LRRC20 |  |  |
| DBIL5P2 |  | ATPAF1 | COQ9 |  |  |
| AHNAK2 |  | LOC284241 | IBTK |  |  |
| LOC100128164 |  | PAX1 | CAMK1D |  |  |
| MAU2 |  | STEAP1 | ZNF37A |  |  |
| PRPF4B |  | FAM129C | RPS17 |  |  |
| RPRM |  | ZNF143 | IRF3 |  |  |
| FKTN |  | CEP83-AS1 | PSIP1 |  |  |
| CCP110 |  | CHMP3 | NUP58 |  |  |
| GATA6-AS1 |  | FBXO3 | XAF1 |  |  |
| C8orf58 |  | GRAMD1A | CAPNS1 |  |  |
| NUS1 |  | CHRND | FBXO38 |  |  |
| MZT2B |  | CD163L1 | MIF-AS1 |  |  |
| PRMT9 |  | ATP2B2-IT2 | SEC14L2 |  |  |
| TTYH2 |  | ZHX2 | BRCA2 |  |  |
| ZNF562 |  | ADGRG6 | ACSF3 |  |  |
| BCOR |  | MMRN2 | PPIG |  |  |
| HIPK3 |  | SYNJ1 | ZSCAN18 |  |  |
| RPF2 |  | GBP4 | RPL14 |  |  |
| BCAR1 |  | GRAP2 | MFSD4B |  |  |
| EFCAB7 |  | TAAR8 | A2ML1 |  |  |
| DNAH1 |  | SMCP | LOC439951 |  |  |
| WDR11 |  | LMO7DN | ATRN |  |  |
| HSPA12B |  | CIDEB | RPS6KA2 |  |  |
| BRSK1 |  | ADGRE1 | CCDC85C |  |  |
| MTMR1 |  | LINC01138 | INPP5F |  |  |
| CRTAP |  | MGC27345 | H2AFJ |  |  |
| SRSF5 |  | TRIM64 | ZFHX3 |  |  |
| SLC25A25 |  | SLC38A2 | SH3GL1 |  |  |
| MTERF3 |  | LOC285957 | SPDEF |  |  |
| ATF4 |  | ACKR4 | EXOSC9 |  |  |
| SHE |  | IGKV1-5 | CECR5 |  |  |
| PTPRM |  | PADI4 | SRXN1 |  |  |
| FBXO11 |  | RHAG | TIMM44 |  |  |
| SCN7A |  | NPVF | CENPJ |  |  |
| CCDC122 |  | STK32C | LOC153811 |  |  |
| HAUS2 |  | MYBPH | ZNF576 |  |  |
| B4GALT4 |  | NSMF | FANCG |  |  |
| RIMKLB |  | GLI2 | PTPMT1 |  |  |
| RASSF4 |  | CXCL5 | CXorf40B |  |  |
| DHX29 |  | PRR21 | IFITM1 |  |  |
| ADIRF |  | CYP39A1 | DMAP1 |  |  |
| MOCS2 |  | ENPP5 | P3H4 |  |  |
| LOC107133515 |  | ORM1 | SERTAD2 |  |  |
| TPK1 |  | PIK3R6 | ITGA8 |  |  |
| IL1RAP |  | AGO1 | NDRG3 |  |  |
| XAGE3 |  | AQP9 | FIP1L1 |  |  |
| FBXO33 |  | LARP4 | SLC27A5 |  |  |
| ADI1 |  | LRRC70 | HOXA11-AS |  |  |
| ZNF667 |  | PRY2 | PRDX2 |  |  |
| RPL31 |  | RFPL2 | RPUSD3 |  |  |
| SPRY2 |  | GP1BA | RFWD3 |  |  |
| DIP2A |  | IQCF2 | PCBP4 |  |  |
| CILP |  | DPY19L4 | PUF60 |  |  |
| LOC100133131 |  | PLIN4 | ACO2 |  |  |
| RPL15 |  | LINC01547 | TFDP1 |  |  |
| EME2 |  | LOC101928377 | COL5A1 |  |  |
| LAMP2 |  | CRHR1 | DSTYK |  |  |
| FAM219B |  | LOC55338 | MEX3A |  |  |
| PLAGL1 |  | HBEGF | UBE4B |  |  |
| RAD9A |  | ZNF135 | C7orf49 |  |  |
| CNTRL |  | TDRKH | ATP1A4 |  |  |
| CCDC82 |  | LOC338620 | ATP10D |  |  |
| FNTA |  | CASC9 | GFOD2 |  |  |
| PIAS2 |  | CCNO | GJB1 |  |  |
| MFSD2A |  | PRO1804 | SENP5 |  |  |
| BTBD3 |  | LOC283665 | RAB3D |  |  |
| LINGO2 |  | SH3BGRL2 | TMEM123 |  |  |
| SLC26A2 |  | TMIE | ZNF672 |  |  |
| NEK1 |  | PPP1R3C | CLDN3 |  |  |
| ATRX |  | CRCT1 | PTGES2 |  |  |
| LCOR |  | FLG | RTFDC1 |  |  |
| ARMC2 |  | MANEA | TCIRG1 |  |  |
| MAPK14 |  | LILRB5 | CMC4 |  |  |
| CHST12 |  | RDH5 | FAF2 |  |  |
| ATP5E |  | CCNT1 | SNX21 |  |  |
| ANAPC5 |  | FBXO27 | SH3BP4 |  |  |
| RPL12 |  | NCOA7 | POLR2G |  |  |
| GPATCH11 |  | GATS | GSTO1 |  |  |
| SUGT1P3 |  | CCL20 | MRPL49 |  |  |
| BOD1L1 |  | FAM30A | MRFAP1L1 |  |  |
| SERF2 |  | SLA | ADRA2C |  |  |
| FNBP1 |  | FSIP2 | XKR8 |  |  |
| NEK11 |  | RABL6 | PFDN5 |  |  |
| CECR7 |  | IL34 | WAC |  |  |
| YPEL3 |  | LINC01559 | C4orf33 |  |  |
| WWC3 |  | CCL23 | PYCR1 |  |  |
| METAP2 |  | CCDC50 | LOXL1 |  |  |
| ABCC13 |  | GABRG3 | ATAD3A |  |  |
| ERVW-1 |  | SYT3 | RNF167 |  |  |
| ANGEL2 |  | RNF182 | BRWD1 |  |  |
| KAT5 |  | AGO4 | PPIAL4A |  |  |
| ELOVL5 |  | LRAT | DCTN2 |  |  |
| JADE2 |  | CNTD2 | MYC |  |  |
| PDK1 |  | C10orf62 | DCAF13 |  |  |
| RUNX1T1 |  | GYG2 | HIST1H2BG |  |  |
| ZNF300 |  | FAM13C | MAPKBP1 |  |  |
| TRABD2B |  | APLF | COG3 |  |  |
| ZFYVE16 |  | DUSP13 | YIPF6 |  |  |
| PPP2R2A |  | CLCN6 | CD2BP2 |  |  |
| CTAGE5 |  | RNF24 | PSMG3 |  |  |
| SMCHD1 |  | GPBAR1 | CTDP1 |  |  |
| CTC1 |  | BARHL1 | PAFAH1B2 |  |  |
| GNG4 |  | GNA15 | DMKN |  |  |
| FAM8A1 |  | LRRC61 | RPP21 |  |  |
| RIOK2 |  | LILRA2 | TEKT2 |  |  |
| CEP152 |  | CARMIL2 | KXD1 |  |  |
| TMEM144 |  | PRKAA2 | MYBBP1A |  |  |
| CHD6 |  | NR4A1 | POLR2I |  |  |
| PLCL2 |  | SMPD3 | TPM1 |  |  |
| PPIL2 |  | CEP162 | KDM2B |  |  |
| CATSPERB |  | SERPINI1 | TMA7 |  |  |
| INVS |  | LINC00341 | TMEM170A |  |  |
| GPR22 |  | PGM5 | CDK2AP2 |  |  |
| NOL10 |  | LOC105371352 | NOXA1 |  |  |
| APOOL |  | BMX | PHF2 |  |  |
| NEBL |  | HHLA1 | MRPS34 |  |  |
| LPAR5 |  | THSD7A | ZC3H15 |  |  |
| LINC00889 |  | ANKRD33 | CC2D2A |  |  |
| CNTN3 |  | TAF5L | ZMYND10 |  |  |
| UCP2 |  | FAM171B | UNC45A |  |  |
| CRIM1 |  | KCTD13 | BEST4 |  |  |
| NLRP2 |  | DICER1-AS1 | CENPO |  |  |
| GBP2 |  | EVI2A | ZNF839 |  |  |
| ANKRD10 |  | LOC105379704 | HINT1 |  |  |
| LHX4-AS1 |  | NPCDR1 | MRPL27 |  |  |
| HIST1H2BE |  | FAM163B | FKBP5 |  |  |
| MGST1 |  | OR51D1 | CLSPN |  |  |
| ZNF146 |  | DOK3 | ZNF254 |  |  |
| FGF10 |  | TCERG1L | UBA52 |  |  |
| BAX |  | CD1D | SEPT6 |  |  |
| NBPF9 |  | PANK1 | RPN2 |  |  |
| TMEM64 |  | ZNF507 | PUS1 |  |  |
| STRADA |  | OSTM1 | ACADVL |  |  |
| PTPN11 |  | CD19 | NADK |  |  |
| TCEA3 |  | MAGEA10 | INPP4B |  |  |
| EGLN1 |  | MBD5 | CTSZ |  |  |
| KHDRBS3 |  | PIK3R3 | TRMT6 |  |  |
| ZNF780A |  | LOC101929998 | HMOX2 |  |  |
| ANK3 |  | ZNF778 | LETMD1 |  |  |
| SLC25A41 |  | GJA5 | GPR150 |  |  |
| FAM98C |  | G6PC2 | TAX1BP3 |  |  |
| JUP |  | DIP2A | ID3 |  |  |
| SMPD4 |  | SNX10 | PNISR |  |  |
| PQLC3 |  | ZNF491 | SPOPL |  |  |
| PRKY |  | ROBO3 | FAM64A |  |  |
| FASTKD2 |  | MYLPF | B9D1 |  |  |
| CXorf57 |  | LY75 | RORB |  |  |
| RGS6 |  | LOC401320 | RRP1 |  |  |
| ZNF738 |  | AHCTF1 | ARMCX6 |  |  |
| FAM160B1 |  | ASPDH | THAP5 |  |  |
| S100A14 |  | DPT | ARPC1B |  |  |
| STXBP3 |  | PITPNM2 | WDR55 |  |  |
| SLC43A1 |  | ITIH3 | STMN1 |  |  |
| CCDC102B |  | TEX40 | APOOL |  |  |
| RRN3P2 |  | DLGAP4 | DRG1 |  |  |
| ACAP1 |  | LOC649294 | OBSL1 |  |  |
| KIAA1147 |  | MLNR | ZNF273 |  |  |
| CCDC120 |  | C9orf50 | RPL9 |  |  |
| AXDND1 |  | PLA2G1B | NCKAP5 |  |  |
| SH3BGRL |  | HAGLR | LDB2 |  |  |
| LOC554206 |  | SLC22A15 | CHMP2A |  |  |
| LOC100130193 |  | C10orf76 | MYO1C |  |  |
| STAG3L4 |  | GPD1 | FUBP1 |  |  |
| SPATA6L |  | CYP3A7 | HSPA8 |  |  |
| LINC01001 |  | CPLX2 | NAT10 |  |  |
| ESYT2 |  | C20orf144 | RPS7 |  |  |
| GOLGA2P2Y |  | RS1 | C19orf53 |  |  |
| ZNF320 |  | ZNF625 | KLHDC8B |  |  |
| NDUFA10 |  | PRSS33 | CCAR2 |  |  |
| OTUD7B |  | NANOG | RSRP1 |  |  |
| PEG10 |  | GRIK2 | RGL2 |  |  |
| VPS11 |  | HIST2H2BE | SLC17A5 |  |  |
| SAP30L |  | PPY | COPS6 |  |  |
| DECR2 |  | LGALS4 | MYL6B |  |  |
| ZNF175 |  | CRYGD | MRPL23 |  |  |
| ATG5 |  | FOXD4 | EXO1 |  |  |
| BACH2 |  | SLC38A10 | SPPL2B |  |  |
| COLCA2 |  | ZFPM2 | FOSL2 |  |  |
| PARP14 |  | NUP58 | SARS |  |  |
| ANKK1 |  | OR12D3 | RCOR1 |  |  |
| STARD5 |  | LOC728613 | FAAP20 |  |  |
| MMS22L |  | C11orf42 | FLJ36840 |  |  |
| MECP2 |  | TTI1 | TRAPPC12 |  |  |
| LIMD1 |  | SPDYE8P | DCTD |  |  |
| KSR2 |  | ORMDL3 | CDC25B |  |  |
| ARHGEF12 |  | TNAP | ARHGEF16 |  |  |
| MCAM |  | ASB9 | SNTB2 |  |  |
| C7orf31 |  | PTPRE | HYI |  |  |
| C1orf27 |  | HAPLN2 | MARVELD3 |  |  |
| ANAPC4 |  | POM121L9P | VPS51 |  |  |
| COQ4 |  | TNFRSF8 | MED16 |  |  |
| GABRP |  | LRRC39 | USP27X |  |  |
| PPM1H |  | TMEM38A | FRG1JP |  |  |
| FAM150B |  | SLC9A8 | TTC17 |  |  |
| C17orf78 |  | MDFIC | TBC1D4 |  |  |
| MAP4K4 |  | FADS2 | KAZALD1 |  |  |
| GBP1 |  | RASA4CP | GSTP1 |  |  |
| ARNTL |  | MX2 | NSA2 |  |  |
| FAM133CP |  | HS6ST2 | SAMD12 |  |  |
| DCLK1 |  | CCDC106 | PIGQ |  |  |
| LINC00578 |  | TMEM182 | PYGB |  |  |
| MATN3 |  | OPN1LW | ZBTB48 |  |  |
| TAS2R13 |  | COL4A6 | SAPCD2 |  |  |
| ATG16L2 |  | MPL | PTPRF |  |  |
| GOLGA2P5 |  | EGR1 | KLHL11 |  |  |
| MRPL50 |  | HIGD1A | HEATR3 |  |  |
| CRIP1 |  | NDFIP2 | ARHGEF2 |  |  |
| UBE3B |  | DYX1C1 | RNF25 |  |  |
| PAPD7 |  | NLRC5 | NEFM |  |  |
| RAB2A |  | PKP1 | NDC80 |  |  |
| TPT1-AS1 |  | CSTL1 | ISLR |  |  |
| CFAP97 |  | TREML2 | ZRANB2 |  |  |
| LPCAT2 |  | INHA | PEX11B |  |  |
| GABRB3 |  | IKZF5 | KRR1 |  |  |
| HDAC10 |  | FAM218A | FLOT2 |  |  |
| C4orf47 |  | LDLR | IFI27 |  |  |
| PAQR6 |  | NPAT | CRACR2B |  |  |
| GMFB |  | FAM86B3P | TCTA |  |  |
| USP15 |  | HLA-DOA | AAMP |  |  |
| EIF2B1 |  | SH2D2A | PAK1IP1 |  |  |
| GXYLT2 |  | GNG7 | IQGAP1 |  |  |
| TROVE2 |  | RGS14 | FAM21C |  |  |
| THSD7A |  | BCL7A | ADAR |  |  |
| CHD8 |  | DPCR1 | LOC100128398 |  |  |
| DDX56 |  | CRHR2 | ELOVL5 |  |  |
| FAM215A |  | CAMLG | DPAGT1 |  |  |
| LMO4 |  | CCDC155 | RAI1 |  |  |
| NFE2L2 |  | PLA2G2D | C16orf45 |  |  |
| MRPL12 |  | PYCR1 | ATP6V1G1 |  |  |
| EP300 |  | DNMT3A | HIST1H2BL |  |  |
| CDHR1 |  | NEURL2 | PPIL4 |  |  |
| ZNF326 |  | KIAA1755 | CNOT11 |  |  |
| STXBP1 |  | CXCR1 | GID8 |  |  |
| SMURF1 |  | LINC01088 | SARS2 |  |  |
| FAM21C |  | GIN1 | AK2 |  |  |
| CHRD |  | ZBTB41 | TOPBP1 |  |  |
| NCKAP1 |  | NUFIP1 | NOA1 |  |  |
| CBLN2 |  | ZNF665 | RAB13 |  |  |
| POPDC2 |  | ACTA1 | RNPEP |  |  |
| SLC35A3 |  | NOX4 | PANK2 |  |  |
| ATP7A |  | ZNF205-AS1 | PNP |  |  |
| DDT |  | KIF20B | PPP2R2C |  |  |
| C8orf31 |  | GJC3 | AP4M1 |  |  |
| EXOC1 |  | RASSF3 | ELFN1 |  |  |
| ELP6 |  | CCL4 | PDIA6 |  |  |
| C5 |  | B3GAT2 | CPT1C |  |  |
| SNAPC3 |  | KRTAP4-5 | FBRSL1 |  |  |
| ERVH-3 |  | TMEM63B | RING1 |  |  |
| RASA2 |  | CD58 | TMEM37 |  |  |
| AK9 |  | GUCY2GP | KLC1 |  |  |
| GPATCH2 |  | LRRN2 | MRPS12 |  |  |
| TRERF1 |  | TTYH1 | STRBP |  |  |
| TBX19 |  | SDAD1 | LDHA |  |  |
| KLRF1 |  | SALL4 | ZNF607 |  |  |
| GLB1L2 |  | LINC00052 | CMTM3 |  |  |
| FAM106A |  | PTPN7 | TMX2 |  |  |
| SCLT1 |  | SLMAP | RPL12 |  |  |
| MTHFD2L |  | ARHGEF28 | ARHGAP1 |  |  |
| TK2 |  | TMCO5A | CCDC186 |  |  |
| MTSS1 |  | PCMTD2 | TRIM44 |  |  |
| BMP3 |  | RAB6B | NOL10 |  |  |
| TAS2R19 |  | MS4A2 | PRRT2 |  |  |
| GABRA2 |  | MYL3 | RBM6 |  |  |
| ZNF664 |  | ALS2CL | EDEM2 |  |  |
| ASB3 |  | GRAPL | USP37 |  |  |
| RSC1A1 |  | LOC286254 | CAV1 |  |  |
| KDR |  | DSG1 | COL18A1 |  |  |
| ABL2 |  | NAA15 | PPCDC |  |  |
| A4GALT |  | DZIP1 | HNRNPA1 |  |  |
| SPG20-AS1 |  | TEK | SDF4 |  |  |
| GATA6 |  | FOXP2 | GALNT12 |  |  |
| ASH1L |  | PRORY | ALDH5A1 |  |  |
| SP100 |  | USP18 | ATP6V1D |  |  |
| COQ2 |  | LINC00654 | LINC00998 |  |  |
| SH3PXD2A |  | TBC1D3P5 | TRMT112 |  |  |
| ZC3HAV1 |  | LIF | PTTG1 |  |  |
| MAN1A2 |  | ADAMTSL3 | BRD2 |  |  |
| LOC142937 |  | MFNG | ZNF853 |  |  |
| HIGD1B |  | RIT2 | FBXW9 |  |  |
| GAPVD1 |  | MSI1 | ICAM3 |  |  |
| OSMR |  | UMODL1-AS1 | MRPL51 |  |  |
| WHAMM |  | IL31RA | TRA2B |  |  |
| PPP2R5C |  | GMPR2 | NAXE |  |  |
| MYLIP |  | HHIPL1 | PLXDC2 |  |  |
| PIP4K2B |  | OGFRL1 | PLEKHB2 |  |  |
| NCOA2 |  | RRN3P2 | EDIL3 |  |  |
| SULT1A2 |  | ADAM11 | RHBDD2 |  |  |
| GOLGA2 |  | RNF41 | SCAMP2 |  |  |
| SPESP1 |  | SFXN5 | ADH5 |  |  |
| ROS1 |  | GPT | E2F2 |  |  |
| ADAMTS8 |  | RLBP1 | RNF26 |  |  |
| AMIGO1 |  | USP46-AS1 | UBE2NL |  |  |
| MAT2A |  | ERC2 | CAD |  |  |
| GUCY1A3 |  | LRRK1 | ZMYM6 |  |  |
| ZNF548 |  | ASPHD2 | FKRP |  |  |
| AKAP6 |  | LINC01615 | CHCHD2 |  |  |
| RNFT1 |  | FAM74A1 | RGAG4 |  |  |
| OSBPL11 |  | ACVR1C | FNDC3B |  |  |
| FILIP1 |  | ZSCAN5A | GUF1 |  |  |
| SORBS3 |  | ASTN1 | METTL13 |  |  |
| PGS1 |  | LEF1-AS1 | MED10 |  |  |
| C1GALT1 |  | GATA4 | CHD3 |  |  |
| BNIP3L |  | DMRTC1 | LRRC41 |  |  |
| RBAK-RBAKDN |  | LRP5L | SGSH |  |  |
| MXI1 |  | TARP | PSMD9 |  |  |
| MED6 |  | IGSF11 | MOCS2 |  |  |
| C8orf59 |  | MC1R | SPATA33 |  |  |
| GUSBP1 |  | LINC00652 | HEATR6 |  |  |
| PAQR3 |  | CTSW | C14orf80 |  |  |
| CATSPER3 |  | UBR3 | SPEN |  |  |
| SFTPA1 |  | STRC | USP4 |  |  |
| FABP3 |  | PCDHB7 | MAT2A |  |  |
| MICB |  | ACE | ILF2 |  |  |
| MYO1C |  | CCDC150 | RPL19 |  |  |
| RBM43 |  | STAC | DNAJC13 |  |  |
| RNF150 |  | ZFX | SERINC3 |  |  |
| TEC |  | GPHB5 | RPP25 |  |  |
| WWC2 |  | PIP5K1P1 | METRN |  |  |
| FAM217B |  | SLC22A23 | SPON2 |  |  |
| FAM132A |  | CBX6 | NPM1 |  |  |
| SAFB |  | SERPINA10 | NR2F1 |  |  |
| ATF6B |  | ATG4C | SPHK2 |  |  |
| RPAIN |  | AADACL2 | FBXO32 |  |  |
| GCNT4 |  | GK5 | ANKRD54 |  |  |
| PCLO |  | ENO2 | PPP6C |  |  |
| SH3BGRL3 |  | PRR30 | DBNL |  |  |
| SORT1 |  | CD6 | USP14 |  |  |
| EHF |  | TMPRSS11D | ENTPD4 |  |  |
| NOS1 |  | GNG2 | INPPL1 |  |  |
| FAM169A |  | HUS1B | TXNL4A |  |  |
| CEP97 |  | AMZ2P1 | JMJD8 |  |  |
| TRIQK |  | LDB3 | SUGP1 |  |  |
| NPEPPS |  | DPF3 | PINX1 |  |  |
| FAF2 |  | PCED1A | PCBP1-AS1 |  |  |
| CEACAM1 |  | LOC100134317 | PYY2 |  |  |
| ZNF99 |  | TLDC1 | NDRG2 |  |  |
| MAVS |  | NPY6R | ECHDC3 |  |  |
| EXD3 |  | MYH15 | ASXL2 |  |  |
| PARN |  | AGRP | FARP1 |  |  |
| THAP9 |  | MAP3K14-AS1 | EDA |  |  |
| SPG7 |  | TXLNB | CEP95 |  |  |
| GABARAPL1 |  | ERCC2 | SPIDR |  |  |
| DBT |  | PAPPA | BTBD9 |  |  |
| LOC220729 |  | TMEM108 | EXOSC6 |  |  |
| ZRANB2 |  | RFFL | OAF |  |  |
| ZNF227 |  | KRT4 | RPS4X |  |  |
| HOXB3 |  | SOX30 | MOV10 |  |  |
| CAMP |  | MBD1 | COASY |  |  |
| RGCC |  | ACTL7A | FKBP11 |  |  |
| ZNF614 |  | ERVH-6 | NUDT14 |  |  |
| PPFIBP2 |  | PURG | FTL |  |  |
| TCEAL2 |  | FPGT | THOC5 |  |  |
| CLDN1 |  | CCL27 | PKP3 |  |  |
| MPC2 |  | HPF1 | DECR2 |  |  |
| IL23R |  | ZNF764 | RPL10 |  |  |
| CUBN |  | CEACAM8 | PAMR1 |  |  |
| TUB |  | CHRNB4 | SNRNP35 |  |  |
| GNAO1 |  | ENPEP | DDX23 |  |  |
| FOLR3 |  | GPRIN2 | ST6GALNAC2 |  |  |
| GYG2 |  | LOC728705 | HHAT |  |  |
| DEF8 |  | PPARGC1A | C1orf43 |  |  |
| PTGR2 |  | RNASE2 | ADCK5 |  |  |
| ABCD1 |  | SULT1C2 | NFATC4 |  |  |
| C10orf128 |  | HHEX | FKBP9 |  |  |
| ASCC3 |  | NAA11 | MYL3 |  |  |
| FAM200A |  | SERTAD2 | VARS2 |  |  |
| BBS5 |  | CR1 | DBT |  |  |
| TNPO1 |  | MTMR7 | TMEM109 |  |  |
| SLC35F1 |  | CCL2 | CEP131 |  |  |
| SNHG21 |  | LOC441204 | PPP2R5D |  |  |
| CGA |  | ZKSCAN3 | CADM1 |  |  |
| PKD1 |  | WDR48 | CACYBP |  |  |
| PSMA3-AS1 |  | HGS | CMTR2 |  |  |
| PTGES2-AS1 |  | TRIM40 | TMEM218 |  |  |
| PMS2 |  | FAM212B | ARPIN |  |  |
| CDK11B |  | PKIB | PTK2 |  |  |
| WDR48 |  | PHF14 | SMU1 |  |  |
| TRAF6 |  | PRSS57 | DNAH3 |  |  |
| MMAB |  | TUBB8 | S100A5 |  |  |
| GAB3 |  | ZSCAN30 | RPL27A |  |  |
| TCEAL1 |  | FCAR | NMT2 |  |  |
| EFHD1 |  | HNF1A | ST5 |  |  |
| CPEB4 |  | HMCES | MGST1 |  |  |
| CCDC88A |  | CDC20B | NME4 |  |  |
| GOLGA8S |  | VPS13C | TMEM203 |  |  |
| YPEL5 |  | NRN1 | HS3ST1 |  |  |
| DNAJC24 |  | TTC31 | SYNJ2 |  |  |
| EYA1 |  | EPM2A | NIFK-AS1 |  |  |
| METTL22 |  | SAMD9 | ZNF646 |  |  |
| KIAA1468 |  | PLK1 | SLC8A1 |  |  |
| GPM6B |  | PITPNC1 | POTEE |  |  |
| TP53BP1 |  | SNX22 | KIF2C |  |  |
| MDM1 |  | FAM169B | ANKZF1 |  |  |
| C20orf194 |  | TMEM133 | ZDHHC14 |  |  |
| ELOVL7 |  | RAB20 | BCAT2 |  |  |
| SH3YL1 |  | P4HA3 | DR1 |  |  |
| VLDLR |  | GULP1 | RIC8A |  |  |
| SH2B3 |  | CIITA | WWC2 |  |  |
| NUB1 |  | ICOSLG | ZMIZ2 |  |  |
| LLPH |  | TRIM34 | TTC38 |  |  |
|  |  | LOC101929122 | THBS3 |  |  |
|  |  | SLC25A51 | HIST1H2AL |  |  |
|  |  | GDF5 | PSMC5 |  |  |
|  |  | EFCAB12 | ZMYND19 |  |  |
|  |  | DAG1 | ERCC5 |  |  |
|  |  | ASAH2 | NCBP2-AS2 |  |  |
|  |  | C2 | EMD |  |  |
|  |  | LIPE | HDAC11 |  |  |
|  |  | HLA-DPB2 | SEC16A |  |  |
|  |  | FAM134B | PYGO1 |  |  |
|  |  | PI15 | VILL |  |  |
|  |  | ZNF490 | GALT |  |  |
|  |  | RDH12 | SPACA9 |  |  |
|  |  | KCNA2 | MEST |  |  |
|  |  | TRIM46 | ATP5G2 |  |  |
|  |  | ZNF385A | EIF1 |  |  |
|  |  | HRG | PSMB6 |  |  |
|  |  | LCE2A | FAM129B |  |  |
|  |  | PKI55 | TRAF3IP2 |  |  |
|  |  | PDE3B | PGRMC1 |  |  |
|  |  | ZNF546 | CAT |  |  |
|  |  | AGBL3 | C19orf33 |  |  |
|  |  | SPRR4 | MED13L |  |  |
|  |  | NOL10 | SMIM22 |  |  |
|  |  | CATSPERG | CNP |  |  |
|  |  | SRD5A3-AS1 | NSUN7 |  |  |
|  |  | PROSER2-AS1 | DERL1 |  |  |
|  |  | MAP2K7 | MXRA8 |  |  |
|  |  | SLC35E1 | THYN1 |  |  |
|  |  | ACSS1 | MCM8 |  |  |
|  |  | C14orf1 | KCTD1 |  |  |
|  |  | CXorf23 | SAV1 |  |  |
|  |  | CACNB3 | LSM14B |  |  |
|  |  | PSTPIP2 | COLGALT1 |  |  |
|  |  | LYSMD4 | IL1R1 |  |  |
|  |  | SCAND2P | ZNF775 |  |  |
|  |  | RPS6KB1 | HPN |  |  |
|  |  | ACSL1 | CLIC1 |  |  |
|  |  | COMMD7 | IFT27 |  |  |
|  |  | TNK1 | BRK1 |  |  |
|  |  | TIGD1 | RGS12 |  |  |
|  |  | BBS7 | WASH1 |  |  |
|  |  | CD99P1 | RALA |  |  |
|  |  | SPRTN | CDC6 |  |  |
|  |  | FAM210A | COX20 |  |  |
|  |  | EEF1A2 | SCNM1 |  |  |
|  |  | BCL6B | ERGIC3 |  |  |
|  |  | SLC15A1 | FGF10 |  |  |
|  |  | SEC62 | F10 |  |  |
|  |  | PSPH | TBC1D22A |  |  |
|  |  | ONECUT1 | LIN7C |  |  |
|  |  | MB21D2 | PNPLA3 |  |  |
|  |  | TNF | WDR90 |  |  |
|  |  | SLC35E4 | E2F7 |  |  |
|  |  | TFAP2E | TMLHE |  |  |
|  |  | DSCAM-AS1 | GPBP1L1 |  |  |
|  |  | VCX | POP7 |  |  |
|  |  | QRICH2 | SELO |  |  |
|  |  | ABHD10 | POLR2D |  |  |
|  |  | CDK5R2 | CD24 |  |  |
|  |  | SLC24A4 | PQLC1 |  |  |
|  |  | IDI2-AS1 | ABCA3 |  |  |
|  |  | TLR4 | PET100 |  |  |
|  |  | STX18-AS1 | NRBP1 |  |  |
|  |  | COLQ | WFS1 |  |  |
|  |  | SPATA6L | KDM3B |  |  |
|  |  | GPLD1 | RPS5 |  |  |
|  |  | RRP7A | ARPC2 |  |  |
|  |  | ZNF169 | ADAM17 |  |  |
|  |  | RBM12B-AS1 | HLA-K |  |  |
|  |  | ZNF567 | RPS3A |  |  |
|  |  | GRM7 | CUL1 |  |  |
|  |  | S100A1 | KMT5A |  |  |
|  |  | OR51G1 | SAP18 |  |  |
|  |  | TCL6 | STRIP1 |  |  |
|  |  | OSBPL6 | R3HCC1 |  |  |
|  |  | CRTC1 | SEMA3B |  |  |
|  |  | PRLH | CALHM2 |  |  |
|  |  | LOC100499194 | TIAL1 |  |  |
|  |  | CCR7 | TRAIP |  |  |
|  |  | SLC39A11 | ADCK2 |  |  |
|  |  | GSTA2 | CCM2 |  |  |
|  |  | WFDC3 | NUPR1 |  |  |
|  |  | CCDC126 | SNHG15 |  |  |
|  |  | LOC101060157 | TOMM22 |  |  |
|  |  | RAPGEF1 | ARL4C |  |  |
|  |  | COBL | AASDHPPT |  |  |
|  |  | ALX4 | ANKRD50 |  |  |
|  |  | FLJ12825 | FAM160A2 |  |  |
|  |  | PLA2G4D | AP2A2 |  |  |
|  |  | PNISR | AKIRIN1 |  |  |
|  |  | B4GALT2 | IFT20 |  |  |
|  |  | CLEC2D | CHMP3 |  |  |
|  |  | NMUR1 | CCNI |  |  |
|  |  | ATP11C | NDUFB10 |  |  |
|  |  | ITGAX | EGLN1 |  |  |
|  |  | FLJ38576 | RPL37 |  |  |
|  |  | GDAP2 | JTB |  |  |
|  |  | NUDT7 | CHKB |  |  |
|  |  | ASPA | SP1 |  |  |
|  |  | BMS1P20 | PICALM |  |  |
|  |  | RIN1 | BBS5 |  |  |
|  |  | WIF1 | LATS1 |  |  |
|  |  | PPP2R2A | HMGN3 |  |  |
|  |  | VPS41 | SRI |  |  |
|  |  | TSSK4 | ITGA1 |  |  |
|  |  | MDH1B | CTDSP2 |  |  |
|  |  | MAP10 | NDUFA3 |  |  |
|  |  | BMP15 | CDC42BPA |  |  |
|  |  | SLC7A14 | LYN |  |  |
|  |  | IKBKB | LINC00691 |  |  |
|  |  | CALHM1 | BCKDK |  |  |
|  |  | RUFY2 | CDKAL1 |  |  |
|  |  | MED14OS | MAGED1 |  |  |
|  |  | APLN | KLF14 |  |  |
|  |  | NEURL1 | CALM1 |  |  |
|  |  | NELL2 | PRDX4 |  |  |
|  |  | LOC401472 | CD9 |  |  |
|  |  | SIGLEC8 | MTG2 |  |  |
|  |  | PLA2G6 | TCAF1 |  |  |
|  |  | ARX | MRPL44 |  |  |
|  |  | CEP120 | BTBD3 |  |  |
|  |  | SLC35F1 | ZDHHC13 |  |  |
|  |  | PCDHGB2 | REV1 |  |  |
|  |  | EID2B | PKDCC |  |  |
|  |  | GPR4 | ZNF430 |  |  |
|  |  | SPTBN2 | GNAQ |  |  |
|  |  | GCC2-AS1 | SERTAD3 |  |  |
|  |  | GNMT | SPG7 |  |  |
|  |  | GLYATL1 | CNPY2 |  |  |
|  |  | RHO | C1orf122 |  |  |
|  |  | DCLRE1C | LOC389033 |  |  |
|  |  | IL24 | HDAC7 |  |  |
|  |  | RLN3 | KMT2C |  |  |
|  |  | LOC221814 | FGFR3 |  |  |
|  |  | CLPSL2 | MTA1 |  |  |
|  |  | LOC93622 | SYCE1L |  |  |
|  |  | LOC102723566 | CDC42EP2 |  |  |
|  |  | IL32 | MLF2 |  |  |
|  |  | MEI1 | RPS27 |  |  |
|  |  | NXPH1 | EFNB3 |  |  |
|  |  | LMOD1 | CDH24 |  |  |
|  |  | ACHE | PPARGC1B |  |  |
|  |  | HAB1 | ALG1L |  |  |
|  |  | PRRG1 | RBM43 |  |  |
|  |  | LINC00312 | ZNF33A |  |  |
|  |  | R3HDML | MRPL17 |  |  |
|  |  | WNT10A | AGPAT1 |  |  |
|  |  | FAM46B | COX6A1 |  |  |
|  |  | BMP10 | CPQ |  |  |
|  |  | LRRC8B | ABL1 |  |  |
|  |  | OSM | RAD54L |  |  |
|  |  | SNAP25 | CSNK2B |  |  |
|  |  | ZNF566 | CGN |  |  |
|  |  | TRAFD1 | STOML3 |  |  |
|  |  | RETN | MGMT |  |  |
|  |  | CLDN9 | TMEM241 |  |  |
|  |  | TRIB3 | ARRDC3 |  |  |
|  |  | SRMS | RUFY3 |  |  |
|  |  | ANKH | GLUL |  |  |
|  |  | RPL15 | PDXDC1 |  |  |
|  |  | APC | WBP1 |  |  |
|  |  | KRT24 | GNG11 |  |  |
|  |  | BBS5 | MSX1 |  |  |
|  |  | FECH | ZNF275 |  |  |
|  |  | LOC100509814 | ACTB |  |  |
|  |  | WISP1 | ATXN2L |  |  |
|  |  | C8orf17 | NMNAT1 |  |  |
|  |  | LOC100128164 | USP11 |  |  |
|  |  | SLC2A9 | COMMD7 |  |  |
|  |  | PRKAA1 | WDR1 |  |  |
|  |  | STON1-GTF2A1L | BPHL |  |  |
|  |  | STRN4 | SORBS2 |  |  |
|  |  | LPCAT2 | C11orf49 |  |  |
|  |  | NBEAL2 | LRWD1 |  |  |
|  |  | PSG6 | RAB3B |  |  |
|  |  | CHAT | PSMB1 |  |  |
|  |  | DEFB4A | MARVELD1 |  |  |
|  |  | DLEU2 | SPR |  |  |
|  |  | ABCC9 | C9orf78 |  |  |
|  |  | C12orf73 | ATP6AP1 |  |  |
|  |  | BCAS4 | APOPT1 |  |  |
|  |  | ZNF574 | TSPAN10 |  |  |
|  |  | C1orf116 | FAM110B |  |  |
|  |  | UBE2B | DDX51 |  |  |
|  |  | LINC01296 | RAN |  |  |
|  |  | NR2E1 | RPS11 |  |  |
|  |  | KAT2B | DEXI |  |  |
|  |  | CACNB2 | MICAL3 |  |  |
|  |  | GNB4 | ULK3 |  |  |
|  |  | LOC440934 | C14orf159 |  |  |
|  |  | DUOXA2 | CCDC130 |  |  |
|  |  | C21orf58 | SNRPB |  |  |
|  |  | C6orf10 | HLA-DRB3 |  |  |
|  |  | TMCC2 | RPS24 |  |  |
|  |  | GABRB1 | MFSD2A |  |  |
|  |  | SLC22A10 | NCLN |  |  |
|  |  | CAPN9 | ABCA7 |  |  |
|  |  | PGP | CCNK |  |  |
|  |  | B3GAT1 | BMF |  |  |
|  |  | FAM132A | IPP |  |  |
|  |  | LOC100506302 | UQCRQ |  |  |
|  |  | GBP3 | TRIM28 |  |  |
|  |  | CDH12 | VDAC1 |  |  |
|  |  | RPL36A | KIAA1522 |  |  |
|  |  | EHBP1 | GGNBP2 |  |  |
|  |  | GOLT1A | GPR180 |  |  |
|  |  | TCHH | GNG5 |  |  |
|  |  | IGKC | OVOL2 |  |  |
|  |  | LINC00852 | LBH |  |  |
|  |  | LYST | POLE4 |  |  |
|  |  | B3GNT9 | PFKP |  |  |
|  |  | GPR82 | PRR15 |  |  |
|  |  | GNB5 | ATP5A1 |  |  |
|  |  | CDKN1A | ZNF727 |  |  |
|  |  | C4orf17 | PPP1R16B |  |  |
|  |  | IRF4 | ALDOAP2 |  |  |
|  |  | TAB3 | TSR2 |  |  |
|  |  | SERHL2 | UBOX5 |  |  |
|  |  | OPRD1 | PFKL |  |  |
|  |  | TAS2R13 | ZNF331 |  |  |
|  |  | GNG4 | MSRB1 |  |  |
|  |  | CD209 | DRAP1 |  |  |
|  |  | MROH5 | NAP1L4 |  |  |
|  |  | SPRR2G | GRAMD1C |  |  |
|  |  | BTN2A3P | AKIRIN2 |  |  |
|  |  | PAG1 | FAM101B |  |  |
|  |  | NACA | TXNDC11 |  |  |
|  |  | CPA4 | TECR |  |  |
|  |  | KLHDC7B | AGAP1 |  |  |
|  |  | SPATA2 | TRRAP |  |  |
|  |  | DRP2 | TMEM213 |  |  |
|  |  | YIPF7 | NTN1 |  |  |
|  |  | TAPT1-AS1 | KRT8 |  |  |
|  |  | RGS5 | NT5C2 |  |  |
|  |  | TICRR | GIPR |  |  |
|  |  | LINC00152 | MRPS7 |  |  |
|  |  | PRSS54 | KIRREL |  |  |
|  |  | SEMA6A | TBCD |  |  |
|  |  | ITIH1 | MCC |  |  |
|  |  | CNR2 | CCDC189 |  |  |
|  |  | DCAF17 | CTSB |  |  |
|  |  | TTC23L | RRP7BP |  |  |
|  |  | OSBPL3 | UGDH |  |  |
|  |  | SOX2 | FANCC |  |  |
|  |  | ADAMTS6 | GOLGA8T |  |  |
|  |  | UBAP2L | HS6ST1 |  |  |
|  |  | ATP13A2 | ATP6AP2 |  |  |
|  |  | ZNF273 | ASH2L |  |  |
|  |  | PKD2L2 | ATP5C1 |  |  |
|  |  | SMAGP | FASTK |  |  |
|  |  | STXBP5L | CAPRIN1 |  |  |
|  |  | ATRNL1 | DNAJC27 |  |  |
|  |  | CCDC62 | STX10 |  |  |
|  |  | TP53TG3HP | SGTA |  |  |
|  |  | CCBE1 | ITM2C |  |  |
|  |  | PCP4 | GPD2 |  |  |
|  |  | ARMC4 | MAT2B |  |  |
|  |  | SCGN | MRPS5 |  |  |
|  |  | TFF1 | AES |  |  |
|  |  | EPB41L4A-AS2 | RFC2 |  |  |
|  |  | PTPN5 | STT3B |  |  |
|  |  | HOXC6 | TRIOBP |  |  |
|  |  | TBX3 | SERF1B |  |  |
|  |  | CYB5R3 | HOMER3 |  |  |
|  |  | HCN1 | LBHD1 |  |  |
|  |  | MORC3 | PPTC7 |  |  |
|  |  | CCDC88C | NR1D2 |  |  |
|  |  | MAGI2-AS3 | RFX1 |  |  |
|  |  | ICA1L | HOXA13 |  |  |
|  |  | FGF7 | SLC5A6 |  |  |
|  |  | PHOSPHO2-KLHL23 | POLR2J |  |  |
|  |  | SMARCA2 | PTPN18 |  |  |
|  |  | NGRN | TUBB4A |  |  |
|  |  | MEX3B | TIMP2 |  |  |
|  |  | C2orf27A | STX5 |  |  |
|  |  | MYO1B | CCDC122 |  |  |
|  |  | MNX1-AS1 | GABRP |  |  |
|  |  | HEATR5A | SLC27A3 |  |  |
|  |  | PNPLA4 | NOP2 |  |  |
|  |  | CCDC82 | EXOSC8 |  |  |
|  |  | LOC100507351 | NOLC1 |  |  |
|  |  | AGXT | SAP130 |  |  |
|  |  | GIT1 | ECHS1 |  |  |
|  |  | TCP10 | PAFAH1B3 |  |  |
|  |  | ADAMTSL1 | FAM46A |  |  |
|  |  | MTM1 | C11orf57 |  |  |
|  |  | HELB | ARMC1 |  |  |
|  |  | ZNF821 | UBE2T |  |  |
|  |  | BSN | VKORC1 |  |  |
|  |  | CASS4 | C19orf60 |  |  |
|  |  | MARCO | GSPT1 |  |  |
|  |  | SCNN1D | MRPL33 |  |  |
|  |  | RPF1 | BTBD18 |  |  |
|  |  | EBF2 | RPS18 |  |  |
|  |  | RLN2 | SND1 |  |  |
|  |  | JAK3 | WDR5 |  |  |
|  |  | MOSPD2 | TAF1B |  |  |
|  |  | ADAMTS2 | ESRRA |  |  |
|  |  | OR6N1 | PPT2 |  |  |
|  |  | GRAMD1C | PIN1 |  |  |
|  |  | AREG | CCNF |  |  |
|  |  | SPIB | SNHG19 |  |  |
|  |  | TSPAN14 | NDUFS1 |  |  |
|  |  | SLC39A3 | DENND5A |  |  |
|  |  | ZNF17 | ANK3 |  |  |
|  |  | LCMT1-AS2 | HELLS |  |  |
|  |  | UBE3D | MITF |  |  |
|  |  | USP30-AS1 | ZDHHC9 |  |  |
|  |  | BIRC5 | MDK |  |  |
|  |  | LOC100286922 | DSE |  |  |
|  |  | FAM226A | CACUL1 |  |  |
|  |  | DTWD1 | EIF4E |  |  |
|  |  | PDZD4 | IL17RA |  |  |
|  |  | OR5A1 | PPAT |  |  |
|  |  | MBL2 | MTHFD1 |  |  |
|  |  | SMIM13 | ACAD9 |  |  |
|  |  | DUSP19 | C1orf52 |  |  |
|  |  | WDR93 | GSDMD |  |  |
|  |  | INSIG2 | REEP4 |  |  |
|  |  | GPR87 | NDUFV2 |  |  |
|  |  | MAK | PWP2 |  |  |
|  |  | LMBRD2 | CYBA |  |  |
|  |  | SCN8A | ANKRD36B |  |  |
|  |  | GLCCI1 | EBPL |  |  |
|  |  | FUT4 | RNF5 |  |  |
|  |  | AKTIP | POLDIP3 |  |  |
|  |  | CCDC149 | POLA2 |  |  |
|  |  | LOC105379362 | LDLRAP1 |  |  |
|  |  | MYOD1 | EPS8 |  |  |
|  |  | MIXL1 | SDF2 |  |  |
|  |  | TRIM9 | ALDH7A1 |  |  |
|  |  | FAM153B | LAMP1 |  |  |
|  |  | TRAM1L1 | SLC16A5 |  |  |
|  |  | ZNF721 | STMN3 |  |  |
|  |  | ZNF214 | LOC644450 |  |  |
|  |  | SPIRE2 | SLC6A6 |  |  |
|  |  | KBTBD3 | ZSWIM8 |  |  |
|  |  | RGS8 | HS1BP3 |  |  |
|  |  | SERPINB2 | STAT6 |  |  |
|  |  | TAPBPL | RNF152 |  |  |
|  |  | IL17RE | COX7A2L |  |  |
|  |  | TFDP2 | LINC00968 |  |  |
|  |  | MCTS1 | C1orf159 |  |  |
|  |  | FOXC2 | FUT6 |  |  |
|  |  | LY96 | PFAS |  |  |
|  |  | PDE6G | PABPC4 |  |  |
|  |  | FOS | RER1 |  |  |
|  |  | HOXA-AS3 | PSMC2 |  |  |
|  |  | NRN1L | SFXN4 |  |  |
|  |  | DKFZP434K028 | PKN2 |  |  |
|  |  | TSPEAR | ALDH4A1 |  |  |
|  |  | SSTR4 | PFDN6 |  |  |
|  |  | CXCL8 | AMD1P3 |  |  |
|  |  | OR10H2 | PIDD1 |  |  |
|  |  | CEP85L | PCK2 |  |  |
|  |  | C1orf210 | DTWD1 |  |  |
|  |  | SARS | AHSA1 |  |  |
|  |  | DBIL5P2 | AFMID |  |  |
|  |  | VTA1 | UBE2L3 |  |  |
|  |  | TEAD3 | ZNF766 |  |  |
|  |  | PIH1D2 | PPP1CC |  |  |
|  |  | HMP19 | FBXO46 |  |  |
|  |  | SCT | PSMB7 |  |  |
|  |  | TESPA1 | PSPC1 |  |  |
|  |  | WFDC5 | DCTPP1 |  |  |
|  |  | SEZ6 | FUCA2 |  |  |
|  |  | THRB | UBAC2 |  |  |
|  |  | FGR | RNF220 |  |  |
|  |  | TRMT1L | PSMB10 |  |  |
|  |  | MIR7-3HG | GPR161 |  |  |
|  |  | STARD9 | CYBB |  |  |
|  |  | ZCCHC2 | FOLH1 |  |  |
|  |  | MC5R | C19orf52 |  |  |
|  |  | SLC2A14 | PCBD1 |  |  |
|  |  | HAUS3 | ZNF516 |  |  |
|  |  | UGT1A6 | GART |  |  |
|  |  | CUBN | NAAA |  |  |
|  |  | SREK1IP1 | NAA40 |  |  |
|  |  | ZBTB7C | TEF |  |  |
|  |  | CD55 | DPH2 |  |  |
|  |  | TBX21 | COG7 |  |  |
|  |  | FBN3 | HSD17B10 |  |  |
|  |  | ORMDL1 | THG1L |  |  |
|  |  | LOC101805491 | VCL |  |  |
|  |  | SLC25A51P1 | EXOC4 |  |  |
|  |  | COMMD6 | KIF20A |  |  |
|  |  | SLITRK2 | SNHG8 |  |  |
|  |  | ANKRD30BL | APH1B |  |  |
|  |  | TTC32 | ANXA11 |  |  |
|  |  | FAM204A | TCN2 |  |  |
|  |  | ACSBG2 | CENPF |  |  |
|  |  | CA14 | SURF1 |  |  |
|  |  | ABL2 | ZNF708 |  |  |
|  |  | GRM8 | EIF4E2 |  |  |
|  |  | AMN1 | CXADR |  |  |
|  |  | DMXL1 | HLA-DRB1 |  |  |
|  |  | SENP2 | POMZP3 |  |  |
|  |  | NKIRAS1 | ODF2L |  |  |
|  |  | HBE1 | HBS1L |  |  |
|  |  | FGD1 | PNPLA4 |  |  |
|  |  | KCNC2 | NOL9 |  |  |
|  |  | ZNF484 | PCNX1 |  |  |
|  |  | CLEC4A | RFWD2 |  |  |
|  |  | ZNF675 | RCN3 |  |  |
|  |  | ZIC4 | LTBP4 |  |  |
|  |  | ZNF154 | NANS |  |  |
|  |  | ZNF177 | RARRES2 |  |  |
|  |  | GPR32 | SH3BGRL2 |  |  |
|  |  | ERN2 | TTC1 |  |  |
|  |  | LOC253805 | PGRMC2 |  |  |
|  |  | CACNA1S | ABHD11 |  |  |
|  |  | PDXK | NABP1 |  |  |
|  |  | OTUD3 | DYNLL1 |  |  |
|  |  | AFAP1L1 | PPIH |  |  |
|  |  | CTXN3 | EFNB2 |  |  |
|  |  | FRMPD3 | AIMP2 |  |  |
|  |  | GNL1 | PTPRS |  |  |
|  |  | CLEC4E | SRRM2 |  |  |
|  |  | OR10H1 | RRM2 |  |  |
|  |  | XAGE3 | GGCX |  |  |
|  |  | WDR33 | B3GALT6 |  |  |
|  |  | LINC01506 | APCDD1 |  |  |
|  |  | COG3 | LY6E |  |  |
|  |  | BCHE | PREP |  |  |
|  |  | MVB12A | ZMYND8 |  |  |
|  |  | GPR52 | ARID5A |  |  |
|  |  | PRAM1 | DENND1A |  |  |
|  |  | SLC8A3 | KDF1 |  |  |
|  |  | YAE1D1 | NUP214 |  |  |
|  |  | C10orf25 | TMEM125 |  |  |
|  |  | LOC101928327 | ZNF503 |  |  |
|  |  | UBL4B | ZNF518A |  |  |
|  |  | LHB | TYK2 |  |  |
|  |  | GOLGA8S | DVL2 |  |  |
|  |  | LOC157740 | GATA2-AS1 |  |  |
|  |  | OMP | BICD2 |  |  |
|  |  | MFRP | NUDT18 |  |  |
|  |  | OXNAD1 | DNAJB11 |  |  |
|  |  | TMEM87B | DAPK1 |  |  |
|  |  | PRRC2B | TFG |  |  |
|  |  | LRRC37B | SMG5 |  |  |
|  |  | PDLIM2 | SNURF |  |  |
|  |  | LRRTM4 | NHLRC4 |  |  |
|  |  | OPTC | IDE |  |  |
|  |  | RSPH6A | TP53I3 |  |  |
|  |  | ZNF829 | TOR1AIP1 |  |  |
|  |  | CHIC1 | TSPAN6 |  |  |
|  |  | CNTFR | TYSND1 |  |  |
|  |  | TNP2 | FAM171B |  |  |
|  |  | OR10H3 | WDFY2 |  |  |
|  |  | OGDH | LMNA |  |  |
|  |  | OR5P1P | TMEM87A |  |  |
|  |  | CYYR1 | NELFE |  |  |
|  |  | ERV3-2 | HSD17B4 |  |  |
|  |  | CMA1 | ERAP2 |  |  |
|  |  | FBXO24 | LRRC8E |  |  |
|  |  | GGT7 | ERH |  |  |
|  |  | LRRC38 | EFTUD2 |  |  |
|  |  | PTGER4 | EMC4 |  |  |
|  |  | CCDC141 | CSDC2 |  |  |
|  |  | ABCC2 | MAK16 |  |  |
|  |  | MORN4 | TMEM39B |  |  |
|  |  | ZNF23 | FBXL4 |  |  |
|  |  | FLT3LG | WNK1 |  |  |
|  |  | NAB2 | SRSF1 |  |  |
|  |  | S100A12 | ARFIP1 |  |  |
|  |  | KIF16B | TIMELESS |  |  |
|  |  | NLRP14 | SLC35F6 |  |  |
|  |  | ZNF616 | IDH3A |  |  |
|  |  | ABHD16B | CCDC12 |  |  |
|  |  | SYNGR1 | ZNRD1 |  |  |
|  |  | GJA3 | ANKK1 |  |  |
|  |  | RNASE6 | EPRS |  |  |
|  |  | RNF151 | SULF2 |  |  |
|  |  | PLAC4 | CDCA3 |  |  |
|  |  | TTC37 | NFE2L1 |  |  |
|  |  | HMGB4 | PLOD1 |  |  |
|  |  | C1orf106 | BRF1 |  |  |
|  |  | SOCS3 | SLC50A1 |  |  |
|  |  | KATNBL1 | MFSD14B |  |  |
|  |  | FEM1B | IK |  |  |
|  |  | ATF7 | MSTO1 |  |  |
|  |  | CDRT1 | USP39 |  |  |
|  |  | GNG3 | MRPL18 |  |  |
|  |  | SYT6 | AAMDC |  |  |
|  |  | SCYL3 | LIMK2 |  |  |
|  |  | IL1B | POLR3H |  |  |
|  |  | CCDC88A | RPLP2 |  |  |
|  |  | FAM182A | RNF38 |  |  |
|  |  | LINC00574 | C1orf109 |  |  |
|  |  | DLK2 | BLVRB |  |  |
|  |  | GALR2 | ARHGAP11A |  |  |
|  |  | KRT18 | CHCHD3 |  |  |
|  |  | SPEN | MRPS16 |  |  |
|  |  | AIMP1 | C7orf26 |  |  |
|  |  | TRPC1 | GNPDA1 |  |  |
|  |  | NFAM1 | NAA50 |  |  |
|  |  | TCEB1 | RPL23A |  |  |
|  |  | C9orf72 | GSTA4 |  |  |
|  |  | NTRK1 | PMS2P4 |  |  |
|  |  | CDNF | VPS13B |  |  |
|  |  | HERC6 | UFC1 |  |  |
|  |  | SPATA22 | CFAP206 |  |  |
|  |  | WDR64 | ZNF680 |  |  |
|  |  | POFUT2 | CD99 |  |  |
|  |  | NTS | ZNF271P |  |  |
|  |  | LOC100130433 | TWIST1 |  |  |
|  |  | ADAMTS9 | CFAP57 |  |  |
|  |  | PRDM10 | SRSF10 |  |  |
|  |  | SLC6A15 | APITD1 |  |  |
|  |  | ZC3H12D | RAI14 |  |  |
|  |  | BPIFB2 | MTPAP |  |  |
|  |  | LOC100996345 | MCM2 |  |  |
|  |  | CHRNG | PSMA6 |  |  |
|  |  | WNK3 | PAGE4 |  |  |
|  |  | SMCHD1 | NT5DC2 |  |  |
|  |  | LGALS8 | CTSF |  |  |
|  |  | DDIT4L | CLDN5 |  |  |
|  |  | HAUS6 | SDHC |  |  |
|  |  | GNAT1 | STARD3 |  |  |
|  |  | ZNF682 | NME6 |  |  |
|  |  | GOLGA2P7 | ADCY3 |  |  |
|  |  | DPP9 | TMEM5 |  |  |
|  |  | CROCCP3 | FAM107B |  |  |
|  |  | PTPRS | SRSF7 |  |  |
|  |  | SEMA6B | ATP8B3 |  |  |
|  |  | LINC01572 | ZNF785 |  |  |
|  |  | C5orf56 | PSMG2 |  |  |
|  |  | IVL | ZNF397 |  |  |
|  |  | KRTAP10-10 | PRRC2C |  |  |
|  |  | ENY2 | RAB2A |  |  |
|  |  | ENO1-AS1 | UBE3C |  |  |
|  |  | VCX3A | ME3 |  |  |
|  |  | PRO2012 | MRPL22 |  |  |
|  |  | U2AF2 | HLA-G |  |  |
|  |  | SLC4A3 | CLSTN1 |  |  |
|  |  | ABCC13 | WSB1 |  |  |
|  |  | OPA3 | HOXA11 |  |  |
|  |  | LOC284412 | RPS26 |  |  |
|  |  | GRINA | CUX1 |  |  |
|  |  | C3orf22 | RPL22 |  |  |
|  |  | PLIN5 | AKT1S1 |  |  |
|  |  | PLA2G2A | ZNF219 |  |  |
|  |  | AP2A2 | ZFYVE19 |  |  |
|  |  | PDS5A | BICD1 |  |  |
|  |  | LRRN3 | NARF |  |  |
|  |  | C9orf129 | TFCP2L1 |  |  |
|  |  | RAB30 | ZNF770 |  |  |
|  |  | SLC29A3 | RPS13 |  |  |
|  |  | ZNF658 | SLC25A1 |  |  |
|  |  | KLHL33 | N4BP1 |  |  |
|  |  | QKI | MEA1 |  |  |
|  |  | SMCR5 | GLUD2 |  |  |
|  |  | KRTAP5-9 | PSD4 |  |  |
|  |  | ENTPD6 | DNAJB14 |  |  |
|  |  | NFASC | ERRFI1 |  |  |
|  |  | MIER3 | CD63 |  |  |
|  |  | GPR171 | PREX1 |  |  |
|  |  | ANKMY1 | ZNF658 |  |  |
|  |  | LINC01558 | ACLY |  |  |
|  |  | INSL5 | MUL1 |  |  |
|  |  | KCNK3 | RECQL5 |  |  |
|  |  | PPOX | DBR1 |  |  |
|  |  | GNA13 | UPF3A |  |  |
|  |  | PHF20 | RPS25 |  |  |
|  |  | NOP9 | PDXK |  |  |
|  |  | MOCS1 | RRP8 |  |  |
|  |  | PIWIL3 | FAM126B |  |  |
|  |  | TIAM1 | PRELID1 |  |  |
|  |  | SORBS1 | GPX4 |  |  |
|  |  | BGLAP | SLC25A3 |  |  |
|  |  | TEX261 | HIBADH |  |  |
|  |  | F11 | NREP |  |  |
|  |  | SALL3 | PRMT2 |  |  |
|  |  | ERO1B | SRP14 |  |  |
|  |  | PTH2 | CEP68 |  |  |
|  |  | GPR15 | LCMT1 |  |  |
|  |  | C1orf226 | HIRIP3 |  |  |
|  |  | CDKN2B | TMA16 |  |  |
|  |  | IFNA4 | COX14 |  |  |
|  |  | PCOLCE2 | POLR1E |  |  |
|  |  | IGHV5-78 | TRIM33 |  |  |
|  |  | NOS2 | TTC14 |  |  |
|  |  | ACTG1P4 | PPP2R5E |  |  |
|  |  | CWC27 | PTRHD1 |  |  |
|  |  | LCA5L | CISD3 |  |  |
|  |  | MUTYH | TLR4 |  |  |
|  |  | PIP | PIGC |  |  |
|  |  | PASK | RPL24 |  |  |
|  |  | RASSF2 | TM9SF1 |  |  |
|  |  | ADGRB3 | NLRP2 |  |  |
|  |  | ST7L | MFSD5 |  |  |
|  |  | SOX21 | SNX17 |  |  |
|  |  | RPS6KA6 | EXOSC5 |  |  |
|  |  | THAP10 | NT5C3A |  |  |
|  |  | MTMR14 | GAK |  |  |
|  |  | ADORA3 | ATP5L |  |  |
|  |  | SPOPL | EIF3K |  |  |
|  |  | GBP5 | PACS2 |  |  |
|  |  | MTMR9LP | TNPO3 |  |  |
|  |  | SLC12A9 | ITIH5 |  |  |
|  |  | TERT | RASSF7 |  |  |
|  |  | DPEP2 | PCDHB11 |  |  |
|  |  | DYNLT3 | LRRFIP2 |  |  |
|  |  | RASGEF1A | ADGRL1 |  |  |
|  |  | ERMAP | BTBD7 |  |  |
|  |  | OTUD4 | SH3D21 |  |  |
|  |  | SHROOM1 | LTA4H |  |  |
|  |  | PNKD | ATP5J |  |  |
|  |  | LINC00626 | CSNK1D |  |  |
|  |  | CEL | KCNG1 |  |  |
|  |  | TPRX1 | DCN |  |  |
|  |  | TUFT1 | CAST |  |  |
|  |  | CDHR1 | UTP4 |  |  |
|  |  | NPPB | MRPL10 |  |  |
|  |  | STK26 | CARD10 |  |  |
|  |  | FAM49A | C22orf39 |  |  |
|  |  | B4GALNT3 | TOMM7 |  |  |
|  |  | SGPP1 | CC2D1B |  |  |
|  |  | HFE | ARHGEF18 |  |  |
|  |  | C17orf105 | CEP290 |  |  |
|  |  | DFNB59 | ZC3HAV1 |  |  |
|  |  | FXR1 | ZSCAN10 |  |  |
|  |  | FSCN2 | MRPS11 |  |  |
|  |  | LANCL3 | C15orf40 |  |  |
|  |  | GAB1 | CPT2 |  |  |
|  |  | LTBP2 | TGFBR1 |  |  |
|  |  | GPR107 | ANGPTL2 |  |  |
|  |  | RBM46 | IQSEC1 |  |  |
|  |  | SND1-IT1 | RPS15A |  |  |
|  |  | KLHL30 | SDSL |  |  |
|  |  | DKFZP434C153 | EIF2AK1 |  |  |
|  |  | AHSP | MRM2 |  |  |
|  |  | C5orf46 | WDR83OS |  |  |
|  |  | GRIA1 | WHSC1 |  |  |
|  |  | SH2D1B | CD84 |  |  |
|  |  | C21orf62-AS1 | ARRDC2 |  |  |
|  |  | PARM1 | HNF1A |  |  |
|  |  | CEP290 | SFPQ |  |  |
|  |  | TMEM19 | GEMIN4 |  |  |
|  |  | ATP13A4 | BMS1 |  |  |
|  |  | FLCN | IRAK1 |  |  |
|  |  | PKIA | PAM |  |  |
|  |  | CUX2 | FBXO21 |  |  |
|  |  | KCND3 | ARL2 |  |  |
|  |  | NCAM2 | WDR45 |  |  |
|  |  | TRIM65 | NSFL1C |  |  |
|  |  | MAPT | C14orf169 |  |  |
|  |  | LINC01596 | DYNC1H1 |  |  |
|  |  | FAM27E5 | ZNF738 |  |  |
|  |  | CORO7 | FBXO7 |  |  |
|  |  | CHRNB3 | RTN4 |  |  |
|  |  | USP47 | RPL36A |  |  |
|  |  | SLC16A10 | MAML1 |  |  |
|  |  | LINC00545 | HNRNPAB |  |  |
|  |  | GLTSCR1 | POLE3 |  |  |
|  |  | LYL1 | ZNF326 |  |  |
|  |  | METTL21B | TAGLN2 |  |  |
|  |  | ALOX15B | SEC11A |  |  |
|  |  | LINC00347 | PITPNA |  |  |
|  |  | SLC8A1 | CANX |  |  |
|  |  | COX7B2 | SLC39A9 |  |  |
|  |  | GLS | NDUFS2 |  |  |
|  |  | GEM | PRKCDBP |  |  |
|  |  | IFNA21 | RITA1 |  |  |
|  |  | LINC01249 | GCDH |  |  |
|  |  | HSD17B6 | MRGBP |  |  |
|  |  | RAB8A | WFDC2 |  |  |
|  |  | ADGRA1 | DUS1L |  |  |
|  |  | GLRA1 | C15orf59 |  |  |
|  |  | FAM41C | JMJD7 |  |  |
|  |  | PGAP1 | ERP44 |  |  |
|  |  | VCX2 | TUSC2 |  |  |
|  |  | DPYS | OGFOD1 |  |  |
|  |  | C15orf52 | REC8 |  |  |
|  |  | GAS8 | IFT122 |  |  |
|  |  | STYX | MAP3K13 |  |  |
|  |  | DPYSL2 | RPL30 |  |  |
|  |  | LINC01001 | C6orf47 |  |  |
|  |  | SLC39A12 | HLA-A |  |  |
|  |  | SLC46A3 | LOC220729 |  |  |
|  |  | LYVE1 | FLCN |  |  |
|  |  | SRSF1 | EHMT2 |  |  |
|  |  | LINC00167 | IQGAP2 |  |  |
|  |  | KLF15 | RPL23 |  |  |
|  |  | GML | UBR2 |  |  |
|  |  | NADK | MTG1 |  |  |
|  |  | CXCL12 | FAM35A |  |  |
|  |  | SGK3 | PGAM1 |  |  |
|  |  | METTL25 | MEF2A |  |  |
|  |  | SPRY3 | CHMP4C |  |  |
|  |  | ALB | PAGR1 |  |  |
|  |  | FZD4 | GBA |  |  |
|  |  | REG1B | THAP4 |  |  |
|  |  | ZNF208 | WAC-AS1 |  |  |
|  |  | XIRP2 | POLR2E |  |  |
|  |  | AATK | ARF6 |  |  |
|  |  | LYSMD3 | KCTD17 |  |  |
|  |  | MAGI2 | CLDN7 |  |  |
|  |  | MOG | CNTN3 |  |  |
|  |  | NME9 | RSC1A1 |  |  |
|  |  | FBXL13 | TJP2 |  |  |
|  |  | HARBI1 | CHD9 |  |  |
|  |  | MCHR2-AS1 | AEN |  |  |
|  |  | DESI2 | SMPD1 |  |  |
|  |  | GIGYF1 | HLA-DRB5 |  |  |
|  |  | SSTR2 | THRA |  |  |
|  |  | XRCC2 | CTNNB1 |  |  |
|  |  | TRPM6 | TPM3P9 |  |  |
|  |  | PANK2 | BACE2 |  |  |
|  |  | MMP16 | EIF2B2 |  |  |
|  |  | AMPH | SPG21 |  |  |
|  |  | TTLL2 | TRMT5 |  |  |
|  |  | LTA | NAGLU |  |  |
|  |  | ZNF624 | SLFN13 |  |  |
|  |  | GNA12 | CHD8 |  |  |
|  |  | CES5A | SF3A1 |  |  |
|  |  | KLF8 | GDI2 |  |  |
|  |  | SYNDIG1L | SLC25A23 |  |  |
|  |  | TAS2R39 | IL6R |  |  |
|  |  | TLR9 | COL4A2 |  |  |
|  |  | CCDC179 | NDUFB8 |  |  |
|  |  | ICAM1 | CLDN11 |  |  |
|  |  | KHDC1 | SOX5 |  |  |
|  |  | KCNIP2 | UBAP2 |  |  |
|  |  | RXFP2 | TIMM8B |  |  |
|  |  | PTPRO | SSRP1 |  |  |
|  |  | NIPAL3 | NPRL3 |  |  |
|  |  | GAPLINC | ZNF786 |  |  |
|  |  | LOC100507205 | COA3 |  |  |
|  |  | NFRKB | JPX |  |  |
|  |  | ZNF506 | INTS3 |  |  |
|  |  | RFNG | TMUB1 |  |  |
|  |  | LOC100132099 | CENPW |  |  |
|  |  | CPA3 | SLC16A3 |  |  |
|  |  | PLEKHA7 | TFPI2 |  |  |
|  |  | DBIL5P | NDUFB7 |  |  |
|  |  | SCN4A | IQSEC2 |  |  |
|  |  | PTPRCAP | SETD6 |  |  |
|  |  | BSND | LFNG |  |  |
|  |  | SEMA6C | EFHC2 |  |  |
|  |  | COL26A1 | LOC107987020 |  |  |
|  |  | ARG2 | SAYSD1 |  |  |
|  |  | PGBD1 | H3F3B |  |  |
|  |  | AFAP1-AS1 | SLC39A13 |  |  |
|  |  | EGR3 | KLHL35 |  |  |
|  |  | HTR4 | WDR81 |  |  |
|  |  | KIF5C | ANGEL2 |  |  |
|  |  | CCDC185 | ARF3 |  |  |
|  |  | PEAK1 | DCTN3 |  |  |
|  |  | POU2F2 | CHTF8 |  |  |
|  |  | FGB | ZNF783 |  |  |
|  |  | MLKL | VPS26B |  |  |
|  |  | TEX29 | KIF18B |  |  |
|  |  | BMP3 | EYA3 |  |  |
|  |  | OR11H12 | C1QTNF2 |  |  |
|  |  | PRELP | PRKY |  |  |
|  |  | CEP104 | NDUFB2 |  |  |
|  |  | SOAT1 | TNRC18 |  |  |
|  |  | RNASE3 | FRAT2 |  |  |
|  |  | KRT76 | SLC35C2 |  |  |
|  |  | LINC01123 | FDFT1 |  |  |
|  |  | MOP-1 | ANKRA2 |  |  |
|  |  | KLHDC1 | RERE |  |  |
|  |  | ZNF418 | KCTD5 |  |  |
|  |  | NFATC2 | KLF5 |  |  |
|  |  | MAMDC2 | KRTCAP2 |  |  |
|  |  | LINC00319 | AMT |  |  |
|  |  | ZNF619 | TSPAN9 |  |  |
|  |  | PUS10 | DDX39A |  |  |
|  |  | SMAD5-AS1 | ANTXR2 |  |  |
|  |  | YOD1 | SMIM10 |  |  |
|  |  | PRNT | QKI |  |  |
|  |  | KCTD21 | SAT1 |  |  |
|  |  | RNF168 | ARHGEF40 |  |  |
|  |  | ZNF681 | RBBP6 |  |  |
|  |  | FEV | TCEA3 |  |  |
|  |  | KDM1B | CAPG |  |  |
|  |  | MCF2L | NKX2-1 |  |  |
|  |  | RAMP3 | SNRPC |  |  |
|  |  | CCNJL | TPT1 |  |  |
|  |  | SRP14-AS1 | CMTM7 |  |  |
|  |  | FAM221B | SLC38A10 |  |  |
|  |  | ATAD3B | NCAPD3 |  |  |
|  |  | IRX1 | ZNF384 |  |  |
|  |  | CAV3 | CAPN1 |  |  |
|  |  | ITGB1BP2 | DDB2 |  |  |
|  |  | VAT1 | BAK1 |  |  |
|  |  | RBM11 | ATP23 |  |  |
|  |  | EPB42 | TIMP1 |  |  |
|  |  | CILP2 | UMAD1 |  |  |
|  |  | LAX1 | COX5B |  |  |
|  |  | CACHD1 | TAPBP |  |  |
|  |  | EXOC8 | IRF9 |  |  |
|  |  | NDST2 | TJAP1 |  |  |
|  |  | CHGB | FNTA |  |  |
|  |  | PTGER2 | CFAP20 |  |  |
|  |  | SSPO | HMBS |  |  |
|  |  | GH1 | SLC41A3 |  |  |
|  |  | NYAP2 | SLC52A3 |  |  |
|  |  | PGC | RPL29P2 |  |  |
|  |  | ZNF879 | TTC39C |  |  |
|  |  | DAZ2 | CIAO1 |  |  |
|  |  | C8orf37 | CSRP2 |  |  |
|  |  | EPHA8 | EFL1 |  |  |
|  |  | C5orf38 | TARBP2 |  |  |
|  |  | SOX8 | DOK1 |  |  |
|  |  | PCDHB13 | CELF6 |  |  |
|  |  | CEMIP | TMEM230 |  |  |
|  |  | NGB | MEDAG |  |  |
|  |  | NDUFA10 | ZNF32 |  |  |
|  |  | CLTB | RASL11B |  |  |
|  |  | SOCS6 | SUPT4H1 |  |  |
|  |  | SPPL2C | NCOA3 |  |  |
|  |  | FAM95B1 | SUDS3 |  |  |
|  |  | LELP1 | CFL1 |  |  |
|  |  | COL11A2 | SRM |  |  |
|  |  | CDK18 | TRMT1 |  |  |
|  |  | STARD13-AS | DOLK |  |  |
|  |  | KCNMA1 | TRPV4 |  |  |
|  |  | ANKRD33B | CXCL16 |  |  |
|  |  | MAP3K1 | IGSF3 |  |  |
|  |  | POMK | BSCL2 |  |  |
|  |  | C9orf131 | RPL31 |  |  |
|  |  | TSPY26P | SLIRP |  |  |
|  |  | ANKRD20A12P | CCDC28B |  |  |
|  |  | OR51E2 | ZNF530 |  |  |
|  |  | CAPRIN2 | PRPSAP2 |  |  |
|  |  | SGIP1 | POP4 |  |  |
|  |  | RBMY2EP | E4F1 |  |  |
|  |  | TMEM185A | USP24 |  |  |
|  |  | PRDM13 | SNRPD1 |  |  |
|  |  | APBA2 | PML |  |  |
|  |  | LCA5 | PILRB |  |  |
|  |  | CNTN4 | INVS |  |  |
|  |  | ST3GAL6-AS1 | HAX1 |  |  |
|  |  | C2CD3 | SHC1 |  |  |
|  |  | CLC | ACCS |  |  |
|  |  | C2orf70 | VAPB |  |  |
|  |  | GUCA2A | PRCC |  |  |
|  |  | OPHN1 | H1FX |  |  |
|  |  | GALNT13 | SERINC2 |  |  |
|  |  | P2RY12 | LOC100505771 |  |  |
|  |  | RGS20 | LNPEP |  |  |
|  |  | OR10A4 | TNFRSF10A |  |  |
|  |  | FER1L6-AS1 | C20orf85 |  |  |
|  |  | IQCF1 | ZNRF1 |  |  |
|  |  | LOC100996385 | SCARB1 |  |  |
|  |  | SATB2 | REXO2 |  |  |
|  |  | SAMD3 | OSBPL11 |  |  |
|  |  | MYH6 | PFKM |  |  |
|  |  | ZNF223 | HOXA7 |  |  |
|  |  | TMOD4 | TOP1MT |  |  |
|  |  | NTM-AS1 | SHROOM1 |  |  |
|  |  | ZNF876P | ABCE1 |  |  |
|  |  | TOX | ABCF3 |  |  |
|  |  | TRIM52-AS1 | SS18L1 |  |  |
|  |  | B3GALT2 | SLC48A1 |  |  |
|  |  | IBA57 | WLS |  |  |
|  |  | CDKN2A-AS1 | HEATR1 |  |  |
|  |  | SH2D1A | C16orf62 |  |  |
|  |  | CD74 | ANKS1A |  |  |
|  |  | GNS | NACA |  |  |
|  |  | NR0B2 | LOC641746 |  |  |
|  |  | PPP1R15A | GSE1 |  |  |
|  |  | TMEM114 | RPS29 |  |  |
|  |  | ASB6 | IMMP2L |  |  |
|  |  | C1QTNF1 | RAMP1 |  |  |
|  |  | PYCR2 | ZSWIM1 |  |  |
|  |  | DDX3Y | RTCB |  |  |
|  |  | RRN3 | COQ7 |  |  |
|  |  | OTOP1 | GDF7 |  |  |
|  |  | SELO | UBR5 |  |  |
|  |  | TMEM200C | CARS2 |  |  |
|  |  | SPEG | ZFP64 |  |  |
|  |  | ZNF664-FAM101A | ADCY6 |  |  |
|  |  | C17orf78 | ARHGAP33 |  |  |
|  |  | LOC100287869 | SLC30A5 |  |  |
|  |  | LRRC57 | LONRF1 |  |  |
|  |  | SDR42E1 | NDC1 |  |  |
|  |  | LOC100287728 | TAP1 |  |  |
|  |  | CYP4F29P | TRNT1 |  |  |
|  |  | SDE2 | PSME4 |  |  |
|  |  | SLC12A3 | RPL11 |  |  |
|  |  | IL15RA | AFG3L2 |  |  |
|  |  | C2orf83 | SKIV2L |  |  |
|  |  | LRRC3 | YY1AP1 |  |  |
|  |  | CCL25 | DENND2D |  |  |
|  |  | TAS1R2 | SLC25A29 |  |  |
|  |  | ITPR1 | CLASRP |  |  |
|  |  | DNAJC24 | ATXN7L3B |  |  |
|  |  | FAM71B | TSFM |  |  |
|  |  | DDI2 | CLDN10 |  |  |
|  |  | BCL2L10 | SETD3 |  |  |
|  |  | CYR61 | RPL36AL |  |  |
|  |  | OR10A5 | TMEM161A |  |  |
|  |  | HSPA1L | KIF4A |  |  |
|  |  | XAGE5 | RPL34 |  |  |
|  |  | CASC15 | CFL1P1 |  |  |
|  |  | MAPK4 | SMIM10L2A |  |  |
|  |  | DYNC2H1 | SPRED1 |  |  |
|  |  | KIAA1549L | MAP3K2 |  |  |
|  |  | ERVV-1 | CLN3 |  |  |
|  |  | FAM106A | FXN |  |  |
|  |  | WIPF1 | CEACAM1 |  |  |
|  |  | FAM3D | C19orf48 |  |  |
|  |  | NKPD1 | PLXNA1 |  |  |
|  |  | SPOCD1 | SETDB1 |  |  |
|  |  | OPRM1 | PDCD6IP |  |  |
|  |  | C9 | CPSF6 |  |  |
|  |  | GPSM1 | UBE2B |  |  |
|  |  | ZFP57 | MESDC1 |  |  |
|  |  | SOX10 | CAPN15 |  |  |
|  |  | NKX2-5 | RPL35A |  |  |
|  |  | LY86 | ZDHHC7 |  |  |
|  |  | ZNF596 | KRT17 |  |  |
|  |  | TBC1D25 | BCL7A |  |  |
|  |  | IFNA2 | MKRN1 |  |  |
|  |  | ZNF740 | MKNK2 |  |  |
|  |  | OR52A1 | VPS29 |  |  |
|  |  | MPDZ | CSTF2T |  |  |
|  |  | FMR1NB | SLC52A2 |  |  |
|  |  | TMEM59L | GALE |  |  |
|  |  | SYNGAP1 | PODXL2 |  |  |
|  |  | ALOX12P2 | RPS20P27 |  |  |
|  |  | KNG1 | ASAP1 |  |  |
|  |  | RNF144A | MTHFR |  |  |
|  |  | CSGALNACT1 | CNOT10 |  |  |
|  |  | LAMP2 | PHKA2 |  |  |
|  |  | ADAL | LOC728392 |  |  |
|  |  | CST13P | SPINT1 |  |  |
|  |  | NKX3-2 | METTL15 |  |  |
|  |  | KRTAP4-2 | CNOT2 |  |  |
|  |  | PP7080 | LDAH |  |  |
|  |  | SLC38A11 | AAED1 |  |  |
|  |  | NRF1 | NGLY1 |  |  |
|  |  | CISD1 | ZMYM2 |  |  |
|  |  | LOC101928068 | UAP1L1 |  |  |
|  |  | SLC2A5 | PAPSS1 |  |  |
|  |  | SIGLEC15 | SDC2 |  |  |
|  |  | PLEKHH1 | HLA-DQA1 |  |  |
|  |  | SLC22A12 | FAM76A |  |  |
|  |  | SPINK1 | LAMA5 |  |  |
|  |  | JPH3 | RNF8 |  |  |
|  |  | KDSR | ATP7B |  |  |
|  |  | SGSM1 | BCAR1 |  |  |
|  |  | STK16 | TBC1D14 |  |  |
|  |  | SHISA3 | ENSA |  |  |
|  |  | SLITRK4 | RPS21 |  |  |
|  |  | SEC61A2 | PHTF2 |  |  |
|  |  | ZSCAN25 | SLC16A1-AS1 |  |  |
|  |  | SYNRG | DUOXA1 |  |  |
|  |  | KLK3 | CCDC30 |  |  |
|  |  | ZNF529-AS1 | CSDE1 |  |  |
|  |  | ZBED9 | MRPL39 |  |  |
|  |  | HCG27 | KLHL36 |  |  |
|  |  | DLG2 | VPS16 |  |  |
|  |  | SREBF1 | ERCC3 |  |  |
|  |  | TMC2 | CKS1B |  |  |
|  |  | EFHD1 | MROH1 |  |  |
|  |  | PRODH2 | PPP1R10 |  |  |
|  |  | UGT2B15 | SMIM7 |  |  |
|  |  | APOBEC2 | PRKCZ |  |  |
|  |  | INS-IGF2 | DTL |  |  |
|  |  | TBK1 | TUBB3 |  |  |
|  |  | IL2RG | RALGAPA1 |  |  |
|  |  | TRIM53AP | TRIM52 |  |  |
|  |  | MBNL3 | DDIT4 |  |  |
|  |  | LCE2B | FAM110A |  |  |
|  |  | KCNJ5 | GIGYF1 |  |  |
|  |  | TRAF3IP2-AS1 | TRAF3 |  |  |
|  |  | TRAPPC13 | SLC22A18 |  |  |
|  |  | SUGCT | FOXM1 |  |  |
|  |  | MAPK8IP1 | SMARCE1 |  |  |
|  |  | RTP5 | PCBD2 |  |  |
|  |  | SSX4B | HMGB3P1 |  |  |
|  |  | RLN1 | PAOX |  |  |
|  |  | PSG11 | FAR1 |  |  |
|  |  | C11orf44 | PITHD1 |  |  |
|  |  | OSBP2 | ECH1 |  |  |
|  |  | NAA16 | C17orf96 |  |  |
|  |  | IL16 | CABP7 |  |  |
|  |  | IRAK4 | PAICS |  |  |
|  |  | LDHAL6A | MTCH2 |  |  |
|  |  | BEND6 | EVL |  |  |
|  |  | SHANK1 | FAM160B2 |  |  |
|  |  | MITF | SZRD1 |  |  |
|  |  | KIAA0513 | ZNF714 |  |  |
|  |  | NMI | NUDT5 |  |  |
|  |  | SUMO3 | ZNF311 |  |  |
|  |  | GRIN3B | CYYR1 |  |  |
|  |  | DOCK8 | PPCS |  |  |
|  |  | LOC642862 | DPP3 |  |  |
|  |  | EGR4 | MTR |  |  |
|  |  | OR2T5 | ZNF462 |  |  |
|  |  | ZSWIM5 | SLC15A4 |  |  |
|  |  | FMO5 | WARS2 |  |  |
|  |  | ZNF404 | LOXL4 |  |  |
|  |  | PPIF | SLC44A4 |  |  |
|  |  | TAC1 | EVI5 |  |  |
|  |  | LOXHD1 | MPC2 |  |  |
|  |  | MIOX | SLC35F2 |  |  |
|  |  | RPS6KA3 | DLG5 |  |  |
|  |  | ELAVL3 | JUND |  |  |
|  |  | TIMP3 | B3GNT9 |  |  |
|  |  | CNOT4 | PTTG3P |  |  |
|  |  | ATP9A | SH3BP5 |  |  |
|  |  | FAM135A | XRCC5 |  |  |
|  |  | C1QTNF9B | TIMM23 |  |  |
|  |  | POU2F3 | AP1AR |  |  |
|  |  | GLP1R | FXR2 |  |  |
|  |  | TCAP | ZC2HC1A |  |  |
|  |  | NR5A1 | MYCNOS |  |  |
|  |  | MID2 | RPA2 |  |  |
|  |  | DZANK1 | NDUFS5 |  |  |
|  |  | PPIP5K2 | DFFA |  |  |
|  |  | ZBTB7B | TIGD6 |  |  |
|  |  | RCBTB1 | SPRY2 |  |  |
|  |  | ABCG5 | DFNA5 |  |  |
|  |  | PCDHGA9 | CELSR2 |  |  |
|  |  | IGSF23 | DNAJC1 |  |  |
|  |  | ERVFRD-1 | ARPC4 |  |  |
|  |  | KCNQ1DN | ENGASE |  |  |
|  |  | CHRM5 | DENND4C |  |  |
|  |  | CD300C | EMSY |  |  |
|  |  | SH3BGR | COL6A2 |  |  |
|  |  | ZNF492 | RAB38 |  |  |
|  |  | CYP7B1 | PPP1R8 |  |  |
|  |  | PDE1A | DDX21 |  |  |
|  |  | ABCC5 | ZNF18 |  |  |
|  |  | CORIN | HIST1H2BN |  |  |
|  |  | RIMKLA | TAF10 |  |  |
|  |  | PBLD | UBE2F |  |  |
|  |  | S100B | UPF3B |  |  |
|  |  | GNB3 | GORASP2 |  |  |
|  |  | POM121L12 | ASF1A |  |  |
|  |  | MIER2 | TRAPPC3 |  |  |
|  |  | TMEM61 | DIP2C |  |  |
|  |  | LBP | ESPL1 |  |  |
|  |  | LOC100505984 | PSEN2 |  |  |
|  |  | FNIP2 | ACY1 |  |  |
|  |  | RGSL1 | MAD1L1 |  |  |
|  |  | TGIF2LY | MMP11 |  |  |
|  |  | CFC1 | ALDH18A1 |  |  |
|  |  | CFTR | ZNF195 |  |  |
|  |  | RNF157-AS1 | DYRK2 |  |  |
|  |  | FLJ36000 | ARL8A |  |  |
|  |  | ESR1 | PHF20 |  |  |
|  |  | LOC101927958 | BRD7P3 |  |  |
|  |  | BMT2 | CEP70 |  |  |
|  |  | LINC00261 | HPS6 |  |  |
|  |  | G0S2 | RB1CC1 |  |  |
|  |  | KRT27 | OSTC |  |  |
|  |  | ZSCAN26 | CFAP53 |  |  |
|  |  | WASIR2 | TMEM132C |  |  |
|  |  | ATP2A1 | OSBPL3 |  |  |
|  |  | RASGRF2 | VAV2 |  |  |
|  |  | GPR88 | TMEM8A |  |  |
|  |  | ANK1 | HACD4 |  |  |
|  |  | SEC24B-AS1 | GRPEL2 |  |  |
|  |  | LCE3D | C11orf54 |  |  |
|  |  | LINC00684 | RPS10P7 |  |  |
|  |  | TRABD | TRIM11 |  |  |
|  |  | GPATCH2L | RBM3 |  |  |
|  |  | NEMF | DACT3 |  |  |
|  |  | TYMP | NOP10 |  |  |
|  |  | GPA33 | GTF2H2C_2 |  |  |
|  |  | KRT81 | HINFP |  |  |
|  |  | SMIM8 | ZNF12 |  |  |
|  |  | ZNF175 | SEMA3E |  |  |
|  |  | SCGB1C1 | PCDH17 |  |  |
|  |  | LRP5 | HINT2 |  |  |
|  |  | CALR3 | ZNF524 |  |  |
|  |  | NUP210 | CARD19 |  |  |
|  |  | FXYD2 | ZNF362 |  |  |
|  |  | SRSF12 | LOC344967 |  |  |
|  |  | RECK | PLK1 |  |  |
|  |  | FAM19A4 | TRADD |  |  |
|  |  | BRWD3 | SHISA5 |  |  |
|  |  | ZFYVE16 | NDUFB2-AS1 |  |  |
|  |  | YPEL2 | CASP9 |  |  |
|  |  | SNX20 | ADNP |  |  |
|  |  | TEX13A | SMAP1 |  |  |
|  |  | OOEP | S100A10 |  |  |
|  |  | PRAMEF1 | SRRT |  |  |
|  |  | ZNF577 | LMNB1 |  |  |
|  |  | RASGRF1 | MED11 |  |  |
|  |  | ACSM5 | UCP3 |  |  |
|  |  | BOLA2-SMG1P6 | PODXL |  |  |
|  |  | TTC30A | EIF4G2 |  |  |
|  |  | FLJ13224 | TRIM5 |  |  |
|  |  | ZMIZ1 | ZNF266 |  |  |
|  |  | CHRNE | GMPPB |  |  |
|  |  | ARRDC3 | MRPS21 |  |  |
|  |  | LINC00919 | USP36 |  |  |
|  |  | AMELY | LRRC17 |  |  |
|  |  | PPP6C | CREB3L4 |  |  |
|  |  | CCAT1 | RRS1 |  |  |
|  |  | RGL4 | PIAS3 |  |  |
|  |  | ABHD13 | SCAF1 |  |  |
|  |  | NOG | TWIST2 |  |  |
|  |  | MUC12 | PDCL3 |  |  |
|  |  | PAPD5 | GCC2 |  |  |
|  |  | MS4A6A | LTBP3 |  |  |
|  |  | KIF27 | C12orf75 |  |  |
|  |  | PARP8 | TMEM87B |  |  |
|  |  | LOC100288846 | PPP1R12C |  |  |
|  |  | LOC202025 | MAN1B1-AS1 |  |  |
|  |  | CEND1 | PRR11 |  |  |
|  |  | RAB11B | GCN1 |  |  |
|  |  | TDH | UGT2B10 |  |  |
|  |  | CPED1 | VAMP2 |  |  |
|  |  | CATSPER1 | TMEM134 |  |  |
|  |  | TAS2R7 | MOAP1 |  |  |
|  |  | C7orf34 | HN1 |  |  |
|  |  | DCD | HAUS8 |  |  |
|  |  | RBMS3 | CCDC92 |  |  |
|  |  | TTC26 | SHC2 |  |  |
|  |  | RDH14 | C2orf70 |  |  |
|  |  | ZNF518A | PNPO |  |  |
|  |  | TOMM40 | RWDD2B |  |  |
|  |  | OR8J1 | NDUFB5 |  |  |
|  |  | RASL10B | ZNF346 |  |  |
|  |  | ADGRF4 | SYTL1 |  |  |
|  |  | IFIT2 | NAXD |  |  |
|  |  | SPTY2D1 | NFAT5 |  |  |
|  |  | TMEM229A | GOLGA4 |  |  |
|  |  | PLB1 | TGFBI |  |  |
|  |  | B3GLCT | MYO9A |  |  |
|  |  | LDOC1 | TMEM150C |  |  |
|  |  | HOXB4 | STK35 |  |  |
|  |  | SDR9C7 | RBM8A |  |  |
|  |  | PRR5L | DHRS1 |  |  |
|  |  | EHBP1L1 | SEC61B |  |  |
|  |  | SIGLEC12 | BRAT1 |  |  |
|  |  | OSBPL1A | DALRD3 |  |  |
|  |  | TPTE2P5 | COX6B1 |  |  |
|  |  | ZKSCAN7 | COMMD1 |  |  |
|  |  | CHRNA7 | NADSYN1 |  |  |
|  |  | PCDHB4 | EFHD2 |  |  |
|  |  | DNAH1 | PRKAG2 |  |  |
|  |  | TTC16 | TNRC6A |  |  |
|  |  | PGM2 | HNRNPD |  |  |
|  |  | PAK4 | HPS5 |  |  |
|  |  | C14orf159 | C9orf142 |  |  |
|  |  | RGS16 | RPL26L1 |  |  |
|  |  | SOS1 | MRPL3 |  |  |
|  |  | SRSF2 | C1orf115 |  |  |
|  |  | FASTKD2 | SKP1 |  |  |
|  |  | POLR2F | MORF4L1 |  |  |
|  |  | ZMAT4 | PYM1 |  |  |
|  |  | GLIS2 | GAS5 |  |  |
|  |  | CFHR5 | SOCS7 |  |  |
|  |  | SAMD14 | SULT1A1 |  |  |
|  |  | MAN1A1 | EPHX2 |  |  |
|  |  | STARD5 | PPP1R16A |  |  |
|  |  | IQCF4 | CYP1B1 |  |  |
|  |  | SMKR1 | BCKDHA |  |  |
|  |  | LRRC25 | USP12 |  |  |
|  |  | LINC00599 | SLC7A1 |  |  |
|  |  | CSN1S2AP | DHRS13 |  |  |
|  |  | CLDN6 | CFAP45 |  |  |
|  |  | MAGEA8 | PKM |  |  |
|  |  | GPR68 | NEK4 |  |  |
|  |  | DUSP9 | CYTH2 |  |  |
|  |  | NPPC | FBXL6 |  |  |
|  |  | RFPL1S | PGM3 |  |  |
|  |  | ZC3HAV1L | SERGEF |  |  |
|  |  | C12orf54 | METTL25 |  |  |
|  |  | AHI1 | PABPC3 |  |  |
|  |  | C6orf58 | CLCC1 |  |  |
|  |  | CXCR2 | DHPS |  |  |
|  |  | USH1G | PRKAG1 |  |  |
|  |  | SNRK | HLA-DPB1 |  |  |
|  |  | CHAC2 | SERPINA1 |  |  |
|  |  | LMX1B | ST3GAL1 |  |  |
|  |  | STK24-AS1 | MXRA7 |  |  |
|  |  | PSIP1 | SEMA5A |  |  |
|  |  | LACC1 | ACTR3 |  |  |
|  |  | KCNK9 | DHX16 |  |  |
|  |  | ADCY10P1 | MAPKAPK3 |  |  |
|  |  | SOX3 | RABGAP1 |  |  |
|  |  | ADAMDEC1 | FAM13B |  |  |
|  |  | SPOCK1 | MREG |  |  |
|  |  | P2RX6P | BHMT2 |  |  |
|  |  | OTUB2 | ERLIN1 |  |  |
|  |  | PGLYRP1 | TMEM132A |  |  |
|  |  | ZSCAN12 | BIN1 |  |  |
|  |  | TNC | USP48 |  |  |
|  |  | ALDH1B1 | HN1L |  |  |
|  |  | SIMC1 | MKKS |  |  |
|  |  | RPL27A | DCTN4 |  |  |
|  |  | HTRA3 | ASXL1 |  |  |
|  |  | UNC80 | GNAI2 |  |  |
|  |  | CNIH3 | ATP1B3 |  |  |
|  |  | KLHDC7A | C9orf3 |  |  |
|  |  | HIC1 | LINC01000 |  |  |
|  |  | IRAK2 | USP40 |  |  |
|  |  | ZNF277 | CHMP1B |  |  |
|  |  | ACTN4 | XBP1 |  |  |
|  |  | FOLR2 | SLC1A5 |  |  |
|  |  | LHX6 | MUC20 |  |  |
|  |  | LOC100129973 | RABEP1 |  |  |
|  |  | EXD3 | CTBS |  |  |
|  |  | AGTR2 | MAGI2-AS3 |  |  |
|  |  | LOC105378732 | NNAT |  |  |
|  |  | IFT81 | SLC23A1 |  |  |
|  |  | NTF3 | KCTD20 |  |  |
|  |  | TMEM130 | SNRPA1 |  |  |
|  |  | CLIC2 | COX17 |  |  |
|  |  | WDR49 | PXN |  |  |
|  |  | PLD2 | FHIT |  |  |
|  |  | LOC100130950 | NLN |  |  |
|  |  | LOC100506557 | HMGXB4 |  |  |
|  |  | ZSCAN31 | STXBP6 |  |  |
|  |  | FIBIN | PIGM |  |  |
|  |  | SSX5 | MUC15 |  |  |
|  |  | DEFB126 | ZNF85 |  |  |
|  |  | HPSE | DHX8 |  |  |
|  |  | EBF3 | METTL1 |  |  |
|  |  | STOML1 | POLR3B |  |  |
|  |  | UPK1A-AS1 | PAQR4 |  |  |
|  |  | CD96 | TM7SF3 |  |  |
|  |  | CCDC171 | HNRNPH3 |  |  |
|  |  | TDO2 | RAB25 |  |  |
|  |  | INHBA | MRPL9 |  |  |
|  |  | NOC4L | PFN1P2 |  |  |
|  |  | ZNF439 | MEGF8 |  |  |
|  |  | MUSK | DVL1 |  |  |
|  |  | HCP5B | SCYL1 |  |  |
|  |  | CCDC120 | TRAK2 |  |  |
|  |  | SERPINA4 | SLC9A3R1 |  |  |
|  |  | SLC9B1 | SNX19 |  |  |
|  |  | RNF214 | ZFYVE9 |  |  |
|  |  | TRAPPC11 | PTPRE |  |  |
|  |  | ZNF44 | SP100 |  |  |
|  |  | NALCN | SMPD2 |  |  |
|  |  | TMEFF2 | PCSK1N |  |  |
|  |  | KCNH1 | C2CD2 |  |  |
|  |  | NRIP2 | MPZL1 |  |  |
|  |  | TMEM59 | ANKRD9 |  |  |
|  |  | RCAN2 | SPAG5 |  |  |
|  |  | NKX1-2 | ERBB2 |  |  |
|  |  | WNT2 | CRIM1 |  |  |
|  |  | FCER1A | NME3 |  |  |
|  |  | STOM | TRABD |  |  |
|  |  | HACD4 | RNF13 |  |  |
|  |  | PRSS36 | DSN1 |  |  |
|  |  | ESPL1 | SEPT9 |  |  |
|  |  | COL8A2 | FANCD2 |  |  |
|  |  | UBAP1L | SPARC |  |  |
|  |  | ZFAND4 | ASTE1 |  |  |
|  |  | ZFP41 | ELMSAN1 |  |  |
|  |  | RNF166 | PVR |  |  |
|  |  | STMN4 | CBLB |  |  |
|  |  | AADAC | CLEC11A |  |  |
|  |  | MROH3P | CAB39 |  |  |
|  |  | STEAP2 | MGC34796 |  |  |
|  |  | ADCYAP1 | ZNF280A |  |  |
|  |  | RASA4 | CHP2 |  |  |
|  |  | SYCP2 | CFLAR |  |  |
|  |  | KIRREL2 | UTP11 |  |  |
|  |  | SLC24A1 | TSNAXIP1 |  |  |
|  |  | QSOX2 | ABHD14B |  |  |
|  |  | ZNF432 | ITGA4 |  |  |
|  |  | TRIM48 | ZPR1 |  |  |
|  |  | CREM | CYP20A1 |  |  |
|  |  | C1orf228 | LCE1A |  |  |
|  |  | PRKAR2B | LOC645166 |  |  |
|  |  | DGKI | URB1 |  |  |
|  |  | FCGR3A | PTDSS1 |  |  |
|  |  | SH3BP5 | RAB3GAP1 |  |  |
|  |  | KIFC3 | EHMT1 |  |  |
|  |  | URB1-AS1 | HARS2 |  |  |
|  |  | ECM2 | DTYMK |  |  |
|  |  | C3orf52 | NEK2 |  |  |
|  |  | ARL17B | ZFYVE27 |  |  |
|  |  | NUDT12 | MTFR1L |  |  |
|  |  | CDHR4 | EIF3C |  |  |
|  |  | LOC728673 | FLNA |  |  |
|  |  | NHLH2 | ECE1 |  |  |
|  |  | COL6A5 | ADGRG1 |  |  |
|  |  | LINC01314 | MBOAT2 |  |  |
|  |  | GAS5-AS1 | C6orf48 |  |  |
|  |  | C17orf51 | BLCAP |  |  |
|  |  | COL4A1 | TMBIM4 |  |  |
|  |  | UBL3 | YWHAQ |  |  |
|  |  | ANP32A | FKBP9P1 |  |  |
|  |  | SPATA12 | MCL1 |  |  |
|  |  | C5orf47 | GNPTG |  |  |
|  |  | PLPP5 | RECQL |  |  |
|  |  | ZNF831 | IREB2 |  |  |
|  |  | ST6GALNAC4 | GTF3A |  |  |
|  |  | OR7G3 | ACAD11 |  |  |
|  |  | MATN3 | COG8 |  |  |
|  |  | TAC4 | KAT2A |  |  |
|  |  | ZNF366 | SEC31A |  |  |
|  |  | EDNRB | DPYSL4 |  |  |
|  |  | CADM3 | SUMF1 |  |  |
|  |  | HCG4B | TRA2A |  |  |
|  |  | HPS3 | INO80E |  |  |
|  |  | ERLIN2 | PDLIM2 |  |  |
|  |  | CLTC | NDUFA4 |  |  |
|  |  | SH2D3C | ZNF771 |  |  |
|  |  | SLC25A2 | TRIM38 |  |  |
|  |  | AFF4 | CPSF4 |  |  |
|  |  | AP3S2 | AFG3L1P |  |  |
|  |  | KRBOX4 | SFRP1 |  |  |
|  |  | ATP8A1 | THAP8 |  |  |
|  |  | CDR1 | VAMP5 |  |  |
|  |  | LOC643201 | KMT2D |  |  |
|  |  | LINC00442 | ZNF692 |  |  |
|  |  | PIFO | FDXR |  |  |
|  |  | DIXDC1 | HOXB6 |  |  |
|  |  | ERP27 | CCNL2 |  |  |
|  |  | CRYAA | HSD17B1 |  |  |
|  |  | TNNT2 | KIAA2013 |  |  |
|  |  | UMODL1 | HDAC3 |  |  |
|  |  | LOC100506472 | SSBP2 |  |  |
|  |  | HES2 | ARFGAP3 |  |  |
|  |  | PHLPP1 | SREBF2 |  |  |
|  |  | TBC1D21 | ETF1 |  |  |
|  |  | SVIP | HOMEZ |  |  |
|  |  | MAPKBP1 | ALG9 |  |  |
|  |  | ABCC3 | RPA4 |  |  |
|  |  | C18orf25 | PRIM2 |  |  |
|  |  | GALNT9 | C1orf174 |  |  |
|  |  | CENPT | P4HA2 |  |  |
|  |  | KCNQ2 | NUP85 |  |  |
|  |  | BCO2 | HK1 |  |  |
|  |  | GTF2H5 | FAM91A1 |  |  |
|  |  | TREH | SLC27A1 |  |  |
|  |  | STX17 | UBR7 |  |  |
|  |  | DUSP5P1 | GRAP |  |  |
|  |  | BAALC | ALPK1 |  |  |
|  |  | ARSB | DNASE1 |  |  |
|  |  | MESP1 | RPS27A |  |  |
|  |  | CGA | SLC8B1 |  |  |
|  |  | CIR1 | ZNF614 |  |  |
|  |  | OR5AK2 | ZBTB2 |  |  |
|  |  | PMS1 | ATF7IP2 |  |  |
|  |  | PVR | CHRNA3 |  |  |
|  |  | MAPK10 | CFAP36 |  |  |
|  |  | UBD | SOD1 |  |  |
|  |  | C1orf234 | CRADD |  |  |
|  |  | CMKLR1 | ARHGEF10 |  |  |
|  |  | BRINP2 | PDGFC |  |  |
|  |  | PCDH10 | EFS |  |  |
|  |  | TIRAP | RNF43 |  |  |
|  |  | SLC12A1 | PPP2R2D |  |  |
|  |  | LINC01350 | ENDOG |  |  |
|  |  | CREBZF | FAHD2A |  |  |
|  |  | USP2 | NPIPB5 |  |  |
|  |  | GRM3 | SMARCC2 |  |  |
|  |  | HS3ST3B1 | PSMC4 |  |  |
|  |  | MGC39584 | ZNF410 |  |  |
|  |  | KRT34 | APOL1 |  |  |
|  |  | ADORA2A | UBAC1 |  |  |
|  |  | ZNF835 | ATP5SL |  |  |
|  |  | DHDH | TAX1BP1 |  |  |
|  |  | SNORD22 | AATF |  |  |
|  |  | EEF1D | NFIC |  |  |
|  |  | RHBDD1 | COPRS |  |  |
|  |  | ZAK | SMAD2 |  |  |
|  |  | TAS2R43 | TCF3 |  |  |
|  |  | ELAVL2 | ADI1 |  |  |
|  |  | TRIM42 | ZNF716 |  |  |
|  |  | THAP9-AS1 | MRPS26 |  |  |
|  |  | OR8G2 | UACA |  |  |
|  |  | MRGPRD | PTDSS2 |  |  |
|  |  | FRG1CP | IKZF2 |  |  |
|  |  | PPBP | MFSD3 |  |  |
|  |  | TRAF6 | CD3G |  |  |
|  |  | LCE2C | SLC39A6 |  |  |
|  |  | ANGPTL1 | GMDS |  |  |
|  |  | TMUB1 | FAM63B |  |  |
|  |  | LGI2 | TIGD5 |  |  |
|  |  | FAM65B | EXOSC7 |  |  |
|  |  | APAF1 | ARHGAP17 |  |  |
|  |  | COL10A1 | YARS |  |  |
|  |  | VSNL1 | TRNP1 |  |  |
|  |  | OPRK1 | CNST |  |  |
|  |  | EFR3B | NCOR2 |  |  |
|  |  | FGFR3 | POLR2C |  |  |
|  |  | LOC100132287 | RPL27 |  |  |
|  |  | ZNF818P | MGRN1 |  |  |
|  |  | KY | CARD9 |  |  |
|  |  | LOC152048 | TMEM209 |  |  |
|  |  | RTP1 | NPR3 |  |  |
|  |  | SAMD9L | PDHB |  |  |
|  |  | MED25 | ECE2 |  |  |
|  |  | ZNF215 | PDE4C |  |  |
|  |  | SH2D3A | AKR7A2P1 |  |  |
|  |  | B3GALT1 | TRPT1 |  |  |
|  |  | MMP24 | ASPSCR1 |  |  |
|  |  | TRDN | MCM6 |  |  |
|  |  | DKFZP434I0714 | RPS23 |  |  |
|  |  | KCNMB1 | DIS3L |  |  |
|  |  | NCR3 | NEUROG3 |  |  |
|  |  | CLDN17 | MDP1 |  |  |
|  |  | OR7E24 | CYTH3 |  |  |
|  |  | ZSCAN22 | ZNF512B |  |  |
|  |  | REEP1 | RBM19 |  |  |
|  |  | KRT71 | UNC5B |  |  |
|  |  | LINC00518 | RNF216P1 |  |  |
|  |  | GPCPD1 | EFCC1 |  |  |
|  |  | POTEB3 | LINC00623 |  |  |
|  |  | CD86 | RPL32 |  |  |
|  |  | SUFU | TGIF1 |  |  |
|  |  | DOC2B | SEPP1 |  |  |
|  |  | ARHGEF7 | ZNF845 |  |  |
|  |  | TRIM21 | TMEM258 |  |  |
|  |  | DPYD | MTMR12 |  |  |
|  |  | MAGEC3 | ING3 |  |  |
|  |  | CCDC26 | SDHAF4 |  |  |
|  |  | SPHK1 | F2R |  |  |
|  |  | CLIP4 | PGM1 |  |  |
|  |  | TTTY22 | MYL12B |  |  |
|  |  | PDZD3 | SLC18A2 |  |  |
|  |  | SH2B1 | DUSP3 |  |  |
|  |  | ROR1 | S100A6 |  |  |
|  |  | CSF3 | MTERF3 |  |  |
|  |  | RORC | HLA-F |  |  |
|  |  | N4BP2L1 | PWWP2B |  |  |
|  |  | LOC100131195 | TACSTD2 |  |  |
|  |  | UMOD | GTPBP1 |  |  |
|  |  | EIF4H | TBRG4 |  |  |
|  |  | TP73 | KIAA1033 |  |  |
|  |  | C6orf99 | CRYL1 |  |  |
|  |  | ERAP1 | RGPD6 |  |  |
|  |  | BRSK1 | CLK2 |  |  |
|  |  | PCLO | PLP2 |  |  |
|  |  | OR7A17 | GAS6 |  |  |
|  |  | MIR924HG | STARD7-AS1 |  |  |
|  |  | TRANK1 | UQCR11 |  |  |
|  |  | CA5BP1 | ZFHX2 |  |  |
|  |  | SMTNL2 | EGLN2 |  |  |
|  |  | ACYP2 | C3orf18 |  |  |
|  |  | KLB | NEK3 |  |  |
|  |  | MAST1 | ZNF217 |  |  |
|  |  | CYTIP | RAB4A |  |  |
|  |  | HTN1 | STXBP5 |  |  |
|  |  | SIRPD | ZNF37BP |  |  |
|  |  | NMRAL1P1 | DNAJC17 |  |  |
|  |  | DPH3P1 | HIST1H4G |  |  |
|  |  | GRIN2A | FXR1 |  |  |
|  |  | ALPK2 | CDK9 |  |  |
|  |  | TET1 | PALM |  |  |
|  |  | HAVCR1 | SLC1A4 |  |  |
|  |  | GIPC2 | LOX |  |  |
|  |  | CPXM2 | SMA4 |  |  |
|  |  | COL5A3 | ATP5G3 |  |  |
|  |  | SLCO2A1 | PRR13 |  |  |
|  |  | RBP4 | NDUFS3 |  |  |
|  |  | NAP1L2 | BANP |  |  |
|  |  | PSG8 | MORF4L2 |  |  |
|  |  | LOC285696 | MARK2 |  |  |
|  |  | FAM122B | REPIN1 |  |  |
|  |  | AQP12B | PRC1 |  |  |
|  |  | ZNF767P | HNRNPF |  |  |
|  |  | HDAC9 | KIAA1549 |  |  |
|  |  | JAM2 | MCUR1 |  |  |
|  |  | FAM109A | CDC42SE2 |  |  |
|  |  | MMP12 | C19orf70 |  |  |
|  |  | MYF6 | PPP1R13B |  |  |
|  |  | CSNK1G2-AS1 | PLEKHA2 |  |  |
|  |  | MALT1 | KAT7 |  |  |
|  |  | IDO2 | DEFB1 |  |  |
|  |  | GBP1 | PRKAR2A |  |  |
|  |  | LCAT | NSUN4 |  |  |
|  |  | FGD3 | CPZ |  |  |
|  |  | PNKP | FOXD2 |  |  |
|  |  | CA5A | WDFY3 |  |  |
|  |  | NBPF22P | APOL6 |  |  |
|  |  | WBP4 | MYO1B |  |  |
|  |  | IGLV6-57 | SYNE1 |  |  |
|  |  | LRP1B | CEP250 |  |  |
|  |  | PDZK1 | ZGRF1 |  |  |
|  |  | GRIP2 | FAM193B |  |  |
|  |  | ALPK1 | EBF4 |  |  |
|  |  | EXOC3L4 | GSK3B |  |  |
|  |  | RUSC2 | BCL2L13 |  |  |
|  |  | TSNAX | SMAD4 |  |  |
|  |  | FOXF2 | BAG5 |  |  |
|  |  | LOC554207 | RPP30 |  |  |
|  |  | TRPC3 | TYMS |  |  |
|  |  | SPESP1 | GOLGA6L10 |  |  |
|  |  | LALBA | BCRP2 |  |  |
|  |  | YPEL1 | PHYKPL |  |  |
|  |  | SPRN | SOCS2 |  |  |
|  |  | NR1H2 | TMEM184C |  |  |
|  |  | MTA1 | MICALL2 |  |  |
|  |  | LOC100128988 | FUT4 |  |  |
|  |  | RNF17 | MOB1A |  |  |
|  |  | EMX1 | KCMF1 |  |  |
|  |  | HTATIP2 | BOD1 |  |  |
|  |  | FGFR1OP2 | MAN2C1 |  |  |
|  |  | PRSS42 | LOC100128775 |  |  |
|  |  | ZNF680 | GBA2 |  |  |
|  |  | LINC01539 | BSDC1 |  |  |
|  |  | LOC100506122 | IQCA1 |  |  |
|  |  | LOC440040 | BOLA3 |  |  |
|  |  | RNF128 | ZNF787 |  |  |
|  |  | OTX2 | LOC643454 |  |  |
|  |  | ABCA5 | SON |  |  |
|  |  | LOC100506125 | FAM45A |  |  |
|  |  | MYCN | RHEB |  |  |
|  |  | VGLL3 | LINS1 |  |  |
|  |  | KLRB1 | GOPC |  |  |
|  |  | LYG2 | DISC1 |  |  |
|  |  | SNN | MRPL24 |  |  |
|  |  | ZNF610 | SLC10A3 |  |  |
|  |  | U2SURP | MACROD2 |  |  |
|  |  | HAO1 | CENPN |  |  |
|  |  | UNC5CL | TK2 |  |  |
|  |  | PKNOX2 | HIST1H3C |  |  |
|  |  | TGFB2 | KMT5B |  |  |
|  |  | PDE10A | RPL10L |  |  |
|  |  | CXCL11 | GALNT11 |  |  |
|  |  | DNPEP | CHTOP |  |  |
|  |  | TECTA | TMEM106C |  |  |
|  |  | LOC101930085 | PRKAR1A |  |  |
|  |  | IDI2 | MTFR1 |  |  |
|  |  | ARMC8 | FBXO17 |  |  |
|  |  | SLC16A4 | NRXN2 |  |  |
|  |  | FBXL22 | RHBDF1 |  |  |
|  |  | AOC3 | MRPL21 |  |  |
|  |  | ASTN2 | NUP93 |  |  |
|  |  | SATB2-AS1 | MAPK14 |  |  |
|  |  | OR10A2 | SWI5 |  |  |
|  |  | RABIF | ANKHD1 |  |  |
|  |  | TRAK1 | METTL9 |  |  |
|  |  | OLFML2A | WBP11 |  |  |
|  |  | DHRS7C | HAND2 |  |  |
|  |  | EME2 | ZNF91 |  |  |
|  |  | FAM3B | KDSR |  |  |
|  |  | PHF12 | SLC37A4 |  |  |
|  |  | TNNI3K | AGBL5 |  |  |
|  |  | F2 | SMIM14 |  |  |
|  |  | FBXO36 | MED12 |  |  |
|  |  | CCDC36 | CRHR2 |  |  |
|  |  | PCSK7 | NSMCE4A |  |  |
|  |  | HSPA4L | ABHD16A |  |  |
|  |  | CERCAM | PRDM6 |  |  |
|  |  | SLC18A3 | TRIM4 |  |  |
|  |  | KHDRBS2 | PGM2L1 |  |  |
|  |  | SSR4P1 | FAM220A |  |  |
|  |  | SLC4A7 | RBM39 |  |  |
|  |  | FAM124A | CDC45 |  |  |
|  |  | HEATR5B | YTHDC1 |  |  |
|  |  | CHD5 | EMILIN1 |  |  |
|  |  | LOC100129406 | ZNF345 |  |  |
|  |  | ERBB3 | ODF2 |  |  |
|  |  | LOC646513 | APOE |  |  |
|  |  | OR7A10 | MED6 |  |  |
|  |  | DCHS2 | GUSB |  |  |
|  |  | CXorf38 | ZEB1 |  |  |
|  |  | GFRA1 | FAM174B |  |  |
|  |  | RORB | ATP6V1F |  |  |
|  |  | LOC286382 | UBE2D2 |  |  |
|  |  | RAVER2 | TMEM55B |  |  |
|  |  | SERPINB11 | KPNA2 |  |  |
|  |  | TMCO2 | POLR2F |  |  |
|  |  | MFSD8 | RAF1 |  |  |
|  |  | SLIT1-AS1 | NFKBIB |  |  |
|  |  | LPP | SPOUT1 |  |  |
|  |  | RNF112 | LSM4 |  |  |
|  |  | SYT4 | MCM3 |  |  |
|  |  | PAPD4 | CHIC2 |  |  |
|  |  | TPT1-AS1 | EDC3 |  |  |
|  |  | BEX5 | BTG3 |  |  |
|  |  | KLRD1 | ZFP90 |  |  |
|  |  | MESTIT1 | SDK2 |  |  |
|  |  | GLS2 | CDKN1C |  |  |
|  |  | SAXO1 | STRA6 |  |  |
|  |  | LYPD3 | KBTBD4 |  |  |
|  |  | WNK1 | MTF2 |  |  |
|  |  | KRTAP9-4 | GTF2F1 |  |  |
|  |  | OR6K2 | FHOD1 |  |  |
|  |  | TXNDC2 | DDX10 |  |  |
|  |  | DNAI1 | GPS1 |  |  |
|  |  | MAGEE1 | RBPMS |  |  |
|  |  | CST1 | ATP5E |  |  |
|  |  | IDUA | TSSC1 |  |  |
|  |  | NUDT11 | TSR3 |  |  |
|  |  | GABBR1 | SPPL2A |  |  |
|  |  | LINC00892 | AFF1 |  |  |
|  |  | FILIP1 | HCCS |  |  |
|  |  | PSAPL1 | ZNF720 |  |  |
|  |  | WFDC9 | HGD |  |  |
|  |  | CFAP65 | RPL38 |  |  |
|  |  | FLJ40194 | DIS3L2 |  |  |
|  |  | RASSF5 | HAUS2 |  |  |
|  |  | MAP3K13 | H3F3C |  |  |
|  |  | TBC1D3B | FOLR1 |  |  |
|  |  | CPN2 | ZEB2 |  |  |
|  |  | SMTN | ROCK2 |  |  |
|  |  | KRT28 | PFKFB4 |  |  |
|  |  | BMP5 | MRPL57 |  |  |
|  |  | APOA5 | NUP88 |  |  |
|  |  | ZNF100 | KIF1C |  |  |
|  |  | UVSSA | RPL26 |  |  |
|  |  | SLC6A5 | VWC2 |  |  |
|  |  | NOTCH2NL | NANOS3 |  |  |
|  |  | DZIP3 | PMVK |  |  |
|  |  | DHRS13 | GCOM1 |  |  |
|  |  | ZNF2 | PRPF19 |  |  |
|  |  | TRIM58 | DDX5 |  |  |
|  |  | ANKLE1 | ANKRD65 |  |  |
|  |  | TTN | CYP1A2 |  |  |
|  |  | RCOR2 | LOC341056 |  |  |
|  |  | OGT | SRSF11 |  |  |
|  |  | PSMD8 | IFIH1 |  |  |
|  |  | DHRS2 | R3HDM2 |  |  |
|  |  | SERPINE1 | FJX1 |  |  |
|  |  | SPATA19 | GSTK1 |  |  |
|  |  | ZNF155 | ALPL |  |  |
|  |  | CD1A | PRELID3B |  |  |
|  |  | ABCG4 | ATRAID |  |  |
|  |  | SMPDL3A | TMEM14A |  |  |
|  |  | HAND1 | TPM2 |  |  |
|  |  | LOC100133331 | FAM204A |  |  |
|  |  | PSD3 | RPL23AP87 |  |  |
|  |  | LOC645321 | USP22 |  |  |
|  |  | ITGA7 | ARMCX3 |  |  |
|  |  | FAM187B | SNX27 |  |  |
|  |  | CACNA1H | DNAJC11 |  |  |
|  |  | CXorf57 | ACBD6 |  |  |
|  |  | ICK | SMDT1 |  |  |
|  |  | CD14 | IFI27L2 |  |  |
|  |  | CC2D1A | BOLA2B |  |  |
|  |  | IGHA1 | UBE2E3 |  |  |
|  |  | DRD5P2 | UQCRFS1 |  |  |
|  |  | CYAT1 | FAM133B |  |  |
|  |  | FAM157A | TBL2 |  |  |
|  |  | IGHV1OR15-1 | GALNT10 |  |  |
|  |  | CCDC144A | TNFRSF21 |  |  |
|  |  | SMA4 | RBM23 |  |  |
|  |  | IGHV3-69-1 | ATG2A |  |  |
|  |  | IGKV3-20 | FARSB |  |  |
|  |  | RPSA | PJA2 |  |  |
|  |  | ZNF664 | SEPW1 |  |  |
|  |  | INS | SLC25A4 |  |  |
|  |  | ARL17A | DYNLRB1 |  |  |
|  |  | IGH | IP6K2 |  |  |
|  |  | IGLV3-21 | CHMP7 |  |  |
|  |  | IGHV3-23 | ZNF428 |  |  |
|  |  |  | TCF25 |  |  |
|  |  |  | LOC100287590 |  |  |
|  |  |  | MRPS30 |  |  |
|  |  |  | BRE |  |  |
|  |  |  | RAB10 |  |  |
|  |  |  | PHF5A |  |  |
|  |  |  | CCDC153 |  |  |
|  |  |  | TPST2 |  |  |
|  |  |  | GAMT |  |  |
|  |  |  | CFI |  |  |
|  |  |  | MYLK |  |  |
|  |  |  | KIAA1551 |  |  |
|  |  |  | UBE2G1 |  |  |
|  |  |  | C15orf57 |  |  |
|  |  |  | ICMT |  |  |
|  |  |  | TECPR1 |  |  |
|  |  |  | UGT2B11 |  |  |
|  |  |  | VPS50 |  |  |
|  |  |  | LAMC2 |  |  |
|  |  |  | RAB1B |  |  |
|  |  |  | MYADM |  |  |
|  |  |  | USP43 |  |  |
|  |  |  | S100A13 |  |  |
|  |  |  | P3H1 |  |  |
|  |  |  | VMA21 |  |  |
|  |  |  | RTEL1 |  |  |
|  |  |  | LPIN2 |  |  |
|  |  |  | FLII |  |  |
|  |  |  | SRPRB |  |  |
|  |  |  | PTPN1 |  |  |
|  |  |  | FAM127B |  |  |
|  |  |  | UBALD2 |  |  |
|  |  |  | FOXP1 |  |  |
|  |  |  | PPA2 |  |  |
|  |  |  | ANO4 |  |  |
|  |  |  | LYRM4 |  |  |
|  |  |  | RBM28 |  |  |
|  |  |  | HBA2 |  |  |
|  |  |  | PTPRA |  |  |
|  |  |  | AACS |  |  |
|  |  |  | AEBP1 |  |  |
|  |  |  | ORMDL2 |  |  |
|  |  |  | NFKBIE |  |  |
|  |  |  | ISCA1 |  |  |
|  |  |  | RANBP9 |  |  |
|  |  |  | LRRC8A |  |  |
|  |  |  | SCCPDH |  |  |
|  |  |  | ELP2 |  |  |
|  |  |  | PAFAH1B1 |  |  |
|  |  |  | ENTPD6 |  |  |
|  |  |  | CLASP2 |  |  |
|  |  |  | EIF4H |  |  |
|  |  |  | CDC16 |  |  |
|  |  |  | GULP1 |  |  |
|  |  |  | KLHL23 |  |  |
|  |  |  | SCO2 |  |  |
|  |  |  | PRR12 |  |  |
|  |  |  | FNDC10 |  |  |
|  |  |  | EPB41 |  |  |
|  |  |  | SMUG1 |  |  |
|  |  |  | FOXO1 |  |  |
|  |  |  | EIF1AY |  |  |
|  |  |  | ADAL |  |  |
|  |  |  | TMEM138 |  |  |
|  |  |  | UBE2J2 |  |  |
|  |  |  | ARHGEF5 |  |  |
|  |  |  | MICU3 |  |  |
|  |  |  | HOXD8 |  |  |
|  |  |  | CDCA5 |  |  |
|  |  |  | SERPINA5 |  |  |
|  |  |  | BAG1 |  |  |
|  |  |  | SLC27A4 |  |  |
|  |  |  | SPATS2L |  |  |
|  |  |  | HLA-DRB4 |  |  |
|  |  |  | PBX1 |  |  |
|  |  |  | MAD2L1 |  |  |
|  |  |  | DYNLT3 |  |  |
|  |  |  | PIAS2 |  |  |
|  |  |  | KIF23 |  |  |
|  |  |  | PDE4A |  |  |
|  |  |  | THOC3 |  |  |
|  |  |  | TXNRD1 |  |  |
|  |  |  | ELAVL1 |  |  |
|  |  |  | GINS2 |  |  |
|  |  |  | ARL4A |  |  |
|  |  |  | CNTNAP2 |  |  |
|  |  |  | TMEM238 |  |  |
|  |  |  | BRD9 |  |  |
|  |  |  | POLDIP2 |  |  |
|  |  |  | WWP1 |  |  |
|  |  |  | FBRS |  |  |
|  |  |  | PSME2 |  |  |
|  |  |  | CTPS1 |  |  |
|  |  |  | PPIL1 |  |  |
|  |  |  | RAB42 |  |  |
|  |  |  | MME |  |  |
|  |  |  | PARL |  |  |
|  |  |  | SAT2 |  |  |
|  |  |  | ATG2B |  |  |
|  |  |  | SIKE1 |  |  |
|  |  |  | FAM58A |  |  |
|  |  |  | FAM133CP |  |  |
|  |  |  | RASEF |  |  |
|  |  |  | LOC100287525 |  |  |
|  |  |  | NRM |  |  |
|  |  |  | SLC47A1 |  |  |
|  |  |  | HOXB4 |  |  |
|  |  |  | KRT5 |  |  |
|  |  |  | CENPBD1 |  |  |
|  |  |  | PTTG2 |  |  |
|  |  |  | PDLIM1 |  |  |
|  |  |  | RBMS1 |  |  |
|  |  |  | KLHL22 |  |  |
|  |  |  | GLYR1 |  |  |
|  |  |  | VEGFB |  |  |
|  |  |  | DECR1 |  |  |
|  |  |  | DENND4A |  |  |
|  |  |  | FAM92B |  |  |
|  |  |  | HEXIM1 |  |  |
|  |  |  | TSKU |  |  |
|  |  |  | PPARA |  |  |
|  |  |  | TNFAIP1 |  |  |
|  |  |  | AGPAT4 |  |  |
|  |  |  | KIAA0907 |  |  |
|  |  |  | NDUFAB1 |  |  |
|  |  |  | PRRC2B |  |  |
|  |  |  | FANCE |  |  |
|  |  |  | GPRC5B |  |  |
|  |  |  | ZNF622 |  |  |
|  |  |  | MARS |  |  |
|  |  |  | CD82 |  |  |
|  |  |  | WDR70 |  |  |
|  |  |  | SRPRA |  |  |
|  |  |  | FUOM |  |  |
|  |  |  | ELF2 |  |  |
|  |  |  | NUDT8 |  |  |
|  |  |  | PSEN1 |  |  |
|  |  |  | MORC4 |  |  |
|  |  |  | SIRT7 |  |  |
|  |  |  | TRUB2 |  |  |
|  |  |  | ZNF675 |  |  |
|  |  |  | PHYHD1 |  |  |
|  |  |  | HMGN4 |  |  |
|  |  |  | SDHB |  |  |
|  |  |  | GFPT1 |  |  |
|  |  |  | ORC5 |  |  |
|  |  |  | SNRNP200 |  |  |
|  |  |  | TMEM160 |  |  |
|  |  |  | RABIF |  |  |
|  |  |  | CDC14A |  |  |
|  |  |  | RAD54L2 |  |  |
|  |  |  | GOLGB1 |  |  |
|  |  |  | ATXN7L3 |  |  |
|  |  |  | SAMD1 |  |  |
|  |  |  | SLC9A8 |  |  |
|  |  |  | CCDC113 |  |  |
|  |  |  | PXMP2 |  |  |
|  |  |  | XPO7 |  |  |
|  |  |  | VAT1 |  |  |
|  |  |  | CPNE2 |  |  |
|  |  |  | SLC44A1 |  |  |
|  |  |  | CTAGE1 |  |  |
|  |  |  | ALMS1P1 |  |  |
|  |  |  | PCCB |  |  |
|  |  |  | MMACHC |  |  |
|  |  |  | ZSWIM6 |  |  |
|  |  |  | ZNF598 |  |  |
|  |  |  | PSMD4 |  |  |
|  |  |  | PPP1R26 |  |  |
|  |  |  | SLC7A8 |  |  |
|  |  |  | TNKS |  |  |
|  |  |  | DGCR2 |  |  |
|  |  |  | TEX264 |  |  |
|  |  |  | FBN2 |  |  |
|  |  |  | KCNN4 |  |  |
|  |  |  | RPL17 |  |  |
|  |  |  | TLE2 |  |  |
|  |  |  | CUEDC2 |  |  |
|  |  |  | PILRA |  |  |
|  |  |  | CH17-340M24.3 |  |  |
|  |  |  | ITPKC |  |  |
|  |  |  | AUNIP |  |  |
|  |  |  | VPS33B |  |  |
|  |  |  | BDH2 |  |  |
|  |  |  | MTRF1 |  |  |
|  |  |  | ZBTB47 |  |  |
|  |  |  | SOD3 |  |  |
|  |  |  | PAPSS2 |  |  |
|  |  |  | DIAPH2 |  |  |
|  |  |  | SESN2 |  |  |
|  |  |  | RPS6KA4 |  |  |
|  |  |  | FAM3A |  |  |
|  |  |  | RXFP1 |  |  |
|  |  |  | ZFP3 |  |  |
|  |  |  | KCNQ1 |  |  |
|  |  |  | RPS20 |  |  |
|  |  |  | RPS12 |  |  |
|  |  |  | TUBA1B |  |  |
|  |  |  | CCT8 |  |  |
|  |  |  | STK10 |  |  |
|  |  |  | STYX |  |  |
|  |  |  | B4GALNT3 |  |  |
|  |  |  | HAUS7 |  |  |
|  |  |  | UBQLN1 |  |  |
|  |  |  | CA5B |  |  |
|  |  |  | GPR89B |  |  |
|  |  |  | EDN1 |  |  |
|  |  |  | SS18 |  |  |
|  |  |  | RPL39 |  |  |
|  |  |  | ELOVL1 |  |  |
|  |  |  | ATP5O |  |  |
|  |  |  | PROSC |  |  |
|  |  |  | TEX10 |  |  |
|  |  |  | TOP3A |  |  |
|  |  |  | PCNA |  |  |
|  |  |  | GOT2 |  |  |
|  |  |  | SNX3 |  |  |
|  |  |  | CDK19 |  |  |
|  |  |  | STOM |  |  |
|  |  |  | LARP7 |  |  |
|  |  |  | ASL |  |  |
|  |  |  | MFSD11 |  |  |
|  |  |  | MASP1 |  |  |
|  |  |  | MFSD6 |  |  |
|  |  |  | CHRNB2 |  |  |
|  |  |  | COA4 |  |  |
|  |  |  | INTS10 |  |  |
|  |  |  | ME2 |  |  |
|  |  |  | PDIA5 |  |  |
|  |  |  | ZSCAN29 |  |  |
|  |  |  | BRI3 |  |  |
|  |  |  | NLK |  |  |
|  |  |  | ITGAE |  |  |
|  |  |  | FNIP1 |  |  |
|  |  |  | CDKN2C |  |  |
|  |  |  | ATP6V0A1 |  |  |
|  |  |  | HMGCL |  |  |
|  |  |  | ACOT11 |  |  |
|  |  |  | LRRTM1 |  |  |
|  |  |  | RNF135 |  |  |
|  |  |  | TOP1 |  |  |
|  |  |  | SUPT20H |  |  |
|  |  |  | SOX17 |  |  |
|  |  |  | POLG |  |  |
|  |  |  | VEPH1 |  |  |
|  |  |  | MAPT |  |  |
|  |  |  | KCNMA1 |  |  |
|  |  |  | JMJD6 |  |  |
|  |  |  | FLNB |  |  |
|  |  |  | RANBP17 |  |  |
|  |  |  | TSPAN17 |  |  |
|  |  |  | SLBP |  |  |
|  |  |  | EIF2B1 |  |  |
|  |  |  | MRPS6 |  |  |
|  |  |  | ANXA6 |  |  |
|  |  |  | SAR1A |  |  |
|  |  |  | ARMC7 |  |  |
|  |  |  | HIST4H4 |  |  |
|  |  |  | PFDN2 |  |  |
|  |  |  | EIF1AD |  |  |
|  |  |  | TMEM42 |  |  |
|  |  |  | MBP |  |  |
|  |  |  | RAD51 |  |  |
|  |  |  | HBA1 |  |  |
|  |  |  | PBXIP1 |  |  |
|  |  |  | S100A4 |  |  |
|  |  |  | SHOC2 |  |  |
|  |  |  | SHMT1 |  |  |
|  |  |  | ANP32D |  |  |
|  |  |  | METTL12 |  |  |
|  |  |  | ZNF512 |  |  |
|  |  |  | SYT1 |  |  |
|  |  |  | DUSP23 |  |  |
|  |  |  | HDAC1 |  |  |
|  |  |  | PGGHG |  |  |
|  |  |  | PALM2-AKAP2 |  |  |
|  |  |  | CBX7 |  |  |
|  |  |  | SEPHS1 |  |  |
|  |  |  | DLG1 |  |  |
|  |  |  | ZNFX1 |  |  |
|  |  |  | TTI2 |  |  |
|  |  |  | LOC105369230 |  |  |
|  |  |  | C5orf51 |  |  |
|  |  |  | ANPEP |  |  |
|  |  |  | VWA3B |  |  |
|  |  |  | RSRC2 |  |  |
|  |  |  | MAGEF1 |  |  |
|  |  |  | MLH1 |  |  |
|  |  |  | ZNF324 |  |  |
|  |  |  | ZNF568 |  |  |
|  |  |  | MSANTD3 |  |  |
|  |  |  | STAG3L1 |  |  |
|  |  |  | MAN2B2 |  |  |
|  |  |  | HIRA |  |  |
|  |  |  | ACACA |  |  |
|  |  |  | RAVER1 |  |  |
|  |  |  | GATSL2 |  |  |
|  |  |  | SCAMP3 |  |  |
|  |  |  | ACTL6A |  |  |
|  |  |  | TP53BP1 |  |  |
|  |  |  | LOC100126784 |  |  |
|  |  |  | PTOV1 |  |  |
|  |  |  | YWHAEP1 |  |  |
|  |  |  | USMG5 |  |  |
|  |  |  | PPIAP30 |  |  |
|  |  |  | EIF2B4 |  |  |
|  |  |  | CYB5R2 |  |  |
|  |  |  | SYBU |  |  |
|  |  |  | CCNA2 |  |  |
|  |  |  | ZCCHC6 |  |  |
|  |  |  | DCUN1D3 |  |  |
|  |  |  | EVX1 |  |  |
|  |  |  | RTN4IP1 |  |  |
|  |  |  | RBSN |  |  |
|  |  |  | PLEKHA5 |  |  |
|  |  |  | CYBRD1 |  |  |
|  |  |  | NBPF14 |  |  |
|  |  |  | TNS3 |  |  |
|  |  |  | VPS35 |  |  |
|  |  |  | PAAF1 |  |  |
|  |  |  | HPS4 |  |  |
|  |  |  | DHX15 |  |  |
|  |  |  | ZNF562 |  |  |
|  |  |  | THAP7 |  |  |
|  |  |  | TSTD3 |  |  |
|  |  |  | SLC38A2 |  |  |
|  |  |  | USP9X |  |  |
|  |  |  | ABCA5 |  |  |
|  |  |  | SEPT8 |  |  |
|  |  |  | HOXD10 |  |  |
|  |  |  | ADAT1 |  |  |
|  |  |  | EN2 |  |  |
|  |  |  | GPSM2 |  |  |
|  |  |  | CREBRF |  |  |
|  |  |  | HOOK2 |  |  |
|  |  |  | CHMP4A |  |  |
|  |  |  | LYRM2 |  |  |
|  |  |  | AIFM1 |  |  |
|  |  |  | DERL2 |  |  |
|  |  |  | SMARCAL1 |  |  |
|  |  |  | SRP68 |  |  |
|  |  |  | AP5S1 |  |  |
|  |  |  | CLN8 |  |  |
|  |  |  | LOC648987 |  |  |
|  |  |  | TMED9 |  |  |
|  |  |  | EIF2D |  |  |
|  |  |  | CDPF1 |  |  |
|  |  |  | DUSP6 |  |  |
|  |  |  | CBFB |  |  |
|  |  |  | TRIP6 |  |  |
|  |  |  | INPP5A |  |  |
|  |  |  | MTMR3 |  |  |
|  |  |  | DHX33 |  |  |
|  |  |  | PPT1 |  |  |
|  |  |  | SHPRH |  |  |
|  |  |  | MIF4GD |  |  |
|  |  |  | ITPR3 |  |  |
|  |  |  | MCAT |  |  |
|  |  |  | SRRM3 |  |  |
|  |  |  | SLC25A38 |  |  |
|  |  |  | SH3YL1 |  |  |
|  |  |  | NIPSNAP3B |  |  |
|  |  |  | TSN |  |  |
|  |  |  | JAG2 |  |  |
|  |  |  | SDHAF2 |  |  |
|  |  |  | UCA1 |  |  |
|  |  |  | FAXDC2 |  |  |
|  |  |  | GRHL2 |  |  |
|  |  |  | ZNF732 |  |  |
|  |  |  | DDX60L |  |  |
|  |  |  | UQCR10 |  |  |
|  |  |  | RASD1 |  |  |
|  |  |  | MDC1 |  |  |
|  |  |  | ETFA |  |  |
|  |  |  | CHRNB1 |  |  |
|  |  |  | CTNS |  |  |
|  |  |  | LARP4B |  |  |
|  |  |  | AIP |  |  |
|  |  |  | MCU |  |  |
|  |  |  | SAMHD1 |  |  |
|  |  |  | MAP2K5 |  |  |
|  |  |  | MPLKIP |  |  |
|  |  |  | IFT52 |  |  |
|  |  |  | ZNF605 |  |  |
|  |  |  | GLIPR1L2 |  |  |
|  |  |  | FAM105A |  |  |
|  |  |  | ZBTB18 |  |  |
|  |  |  | FAM134A |  |  |
|  |  |  | ZNF768 |  |  |
|  |  |  | LDHB |  |  |
|  |  |  | SOWAHC |  |  |
|  |  |  | SCPEP1 |  |  |
|  |  |  | MOB3B |  |  |
|  |  |  | C14orf119 |  |  |
|  |  |  | NUAK1 |  |  |
|  |  |  | SLC25A17 |  |  |
|  |  |  | MMS22L |  |  |
|  |  |  | LOC729887 |  |  |
|  |  |  | THOC6 |  |  |
|  |  |  | SIDT2 |  |  |
|  |  |  | HMHB1 |  |  |
|  |  |  | RRP36 |  |  |
|  |  |  | GTPBP3 |  |  |
|  |  |  | PPFIA1 |  |  |
|  |  |  | PLLP |  |  |
|  |  |  | MRPL42P5 |  |  |
|  |  |  | TRPM4 |  |  |
|  |  |  | CMIP |  |  |
|  |  |  | ITGA10 |  |  |
|  |  |  | TSNAX |  |  |
|  |  |  | HLA-DMB |  |  |
|  |  |  | RMI2 |  |  |
|  |  |  | STK11IP |  |  |
|  |  |  | RBBP7 |  |  |
|  |  |  | POR |  |  |
|  |  |  | CCDC142 |  |  |
|  |  |  | SMARCD3 |  |  |
|  |  |  | RPL23AP82 |  |  |
|  |  |  | SUPV3L1 |  |  |
|  |  |  | GALNT2 |  |  |
|  |  |  | BAIAP2L2 |  |  |
|  |  |  | FEM1A |  |  |
|  |  |  | C17orf80 |  |  |
|  |  |  | LSS |  |  |
|  |  |  | DCHS1 |  |  |
|  |  |  | ST6GALNAC1 |  |  |
|  |  |  | GLB1 |  |  |
|  |  |  | PHKG2 |  |  |
|  |  |  | FXYD3 |  |  |
|  |  |  | PCDH18 |  |  |
|  |  |  | HJURP |  |  |
|  |  |  | CLEC3B |  |  |
|  |  |  | VPS39 |  |  |
|  |  |  | HSPA14 |  |  |
|  |  |  | SCUBE2 |  |  |
|  |  |  | ALKBH2 |  |  |
|  |  |  | MAPRE2 |  |  |
|  |  |  | NUP205 |  |  |
|  |  |  | STAT3 |  |  |
|  |  |  | FAM171A1 |  |  |
|  |  |  | PCSK5 |  |  |
|  |  |  | SNX12 |  |  |
|  |  |  | GPAT4 |  |  |
|  |  |  | KIAA1429 |  |  |
|  |  |  | MTBP |  |  |
|  |  |  | ECHDC2 |  |  |
|  |  |  | NUFIP2 |  |  |
|  |  |  | FLJ10038 |  |  |
|  |  |  | CHKA |  |  |
|  |  |  | GOLGA2P2Y |  |  |
|  |  |  | SIRT3 |  |  |
|  |  |  | IARS2 |  |  |
|  |  |  | PNKD |  |  |
|  |  |  | GPSM1 |  |  |
|  |  |  | SLC25A37 |  |  |
|  |  |  | PPIL2 |  |  |
|  |  |  | LIMK1 |  |  |
|  |  |  | F8A1 |  |  |
|  |  |  | ATRIP |  |  |
|  |  |  | EMC3 |  |  |
|  |  |  | TBC1D7 |  |  |
|  |  |  | TRPC4AP |  |  |
|  |  |  | ALG11 |  |  |
|  |  |  | FUK |  |  |
|  |  |  | TSPAN3 |  |  |
|  |  |  | COX7C |  |  |
|  |  |  | LOC105274304 |  |  |
|  |  |  | MRPL52 |  |  |
|  |  |  | ANKS3 |  |  |
|  |  |  | LOC101928659 |  |  |
|  |  |  | ZNF76 |  |  |
|  |  |  | NRAV |  |  |
|  |  |  | SMO |  |  |
|  |  |  | NIPAL3 |  |  |
|  |  |  | VWA9 |  |  |
|  |  |  | CORO1C |  |  |
|  |  |  | RBM14 |  |  |
|  |  |  | KHK |  |  |
|  |  |  | RPS6KC1 |  |  |
|  |  |  | ZNF654 |  |  |
|  |  |  | FAM135A |  |  |
|  |  |  | BEX3 |  |  |
|  |  |  | COQ2 |  |  |
|  |  |  | TOMM40L |  |  |
|  |  |  | LPAR2 |  |  |
|  |  |  | LARS2 |  |  |
|  |  |  | AR |  |  |
|  |  |  | B3GALT4 |  |  |
|  |  |  | STRN |  |  |
|  |  |  | ZMYM5 |  |  |
|  |  |  | RPUSD2 |  |  |
|  |  |  | ZHX3 |  |  |
|  |  |  | ARL1 |  |  |
|  |  |  | CEP104 |  |  |
|  |  |  | ZNF581 |  |  |
|  |  |  | THEM6 |  |  |
|  |  |  | TRIM23 |  |  |
|  |  |  | PTRF |  |  |
|  |  |  | C17orf53 |  |  |
|  |  |  | PRPF38A |  |  |
|  |  |  | TCP1 |  |  |
|  |  |  | ALKBH4 |  |  |
|  |  |  | SRSF5 |  |  |
|  |  |  | C9orf69 |  |  |
|  |  |  | MTOR |  |  |
|  |  |  | LOC148413 |  |  |
|  |  |  | HIST2H2BC |  |  |
|  |  |  | RPS7P5 |  |  |
|  |  |  | SLC25A19 |  |  |
|  |  |  | CHURC1 |  |  |
|  |  |  | SRP19 |  |  |
|  |  |  | MESP1 |  |  |
|  |  |  | RAD51C |  |  |
|  |  |  | NAA60 |  |  |
|  |  |  | RNF31 |  |  |
|  |  |  | MMS19 |  |  |
|  |  |  | NDN |  |  |
|  |  |  | SHCBP1 |  |  |
|  |  |  | IGBP1 |  |  |
|  |  |  | METTL21B |  |  |
|  |  |  | EPS15 |  |  |
|  |  |  | NEDD4L |  |  |
|  |  |  | FN1 |  |  |
|  |  |  | LRRC47 |  |  |
|  |  |  | COX7A1 |  |  |
|  |  |  | OVGP1 |  |  |
|  |  |  | ZNF347 |  |  |
|  |  |  | CACNB3 |  |  |
|  |  |  | FAM149A |  |  |
|  |  |  | SF3A3 |  |  |
|  |  |  | STK4 |  |  |
|  |  |  | OTUD5 |  |  |
|  |  |  | PSME3 |  |  |
|  |  |  | OFD1 |  |  |
|  |  |  | CDK5 |  |  |
|  |  |  | DGKH |  |  |
|  |  |  | DGAT1 |  |  |
|  |  |  | MTHFS |  |  |
|  |  |  | DTD1 |  |  |
|  |  |  | IQCG |  |  |
|  |  |  | ARHGEF17 |  |  |
|  |  |  | SLC25A33 |  |  |
|  |  |  | CELF1 |  |  |
|  |  |  | C1QB |  |  |
|  |  |  | TUFT1 |  |  |
|  |  |  | CD36 |  |  |
|  |  |  | LIN54 |  |  |
|  |  |  | BHLHB9 |  |  |
|  |  |  | SEMA4C |  |  |
|  |  |  | MED8 |  |  |
|  |  |  | SMIM10L2B |  |  |
|  |  |  | KCNB1 |  |  |
|  |  |  | TMEM50A |  |  |
|  |  |  | BNIP3 |  |  |
|  |  |  | TEAD4 |  |  |
|  |  |  | SORBS3 |  |  |
|  |  |  | MRPL19 |  |  |
|  |  |  | PHLDB1 |  |  |
|  |  |  | VMP1 |  |  |
|  |  |  | FBLN2 |  |  |
|  |  |  | RRP1B |  |  |
|  |  |  | NICN1 |  |  |
|  |  |  | FGD1 |  |  |
|  |  |  | IFNGR2 |  |  |
|  |  |  | MAOB |  |  |
|  |  |  | NSMCE3 |  |  |
|  |  |  | CACNA1G |  |  |
|  |  |  | SCARB2 |  |  |
|  |  |  | ZNF513 |  |  |
|  |  |  | ATXN1 |  |  |
|  |  |  | SDC4 |  |  |
|  |  |  | TNFSF13 |  |  |
|  |  |  | ACSS3 |  |  |
|  |  |  | PMM1 |  |  |
|  |  |  | XPO6 |  |  |
|  |  |  | MFSD9 |  |  |
|  |  |  | FBXO34 |  |  |
|  |  |  | FH |  |  |
|  |  |  | FANCB |  |  |
|  |  |  | IFT22 |  |  |
|  |  |  | VPS4B |  |  |
|  |  |  | FAHD1 |  |  |
|  |  |  | CLEC16A |  |  |
|  |  |  | CD59 |  |  |
|  |  |  | PER1 |  |  |
|  |  |  | SMIM6 |  |  |
|  |  |  | JAG1 |  |  |
|  |  |  | SCRIB |  |  |
|  |  |  | LACC1 |  |  |
|  |  |  | VPS37C |  |  |
|  |  |  | SERPINB8 |  |  |
|  |  |  | CRLF1 |  |  |
|  |  |  | MANSC1 |  |  |
|  |  |  | JAGN1 |  |  |
|  |  |  | LAMB2 |  |  |
|  |  |  | ARHGAP20 |  |  |
|  |  |  | ARFGAP2 |  |  |
|  |  |  | BAZ2A |  |  |
|  |  |  | FOCAD |  |  |
|  |  |  | GFRA2 |  |  |
|  |  |  | EPB41L4B |  |  |
|  |  |  | DDAH2 |  |  |
|  |  |  | FBXO45 |  |  |
|  |  |  | RPL23AP7 |  |  |
|  |  |  | OTUB1 |  |  |
|  |  |  | PDPR |  |  |
|  |  |  | SETD7 |  |  |
|  |  |  | UBE4A |  |  |
|  |  |  | ELP6 |  |  |
|  |  |  | TYRO3 |  |  |
|  |  |  | POLR3E |  |  |
|  |  |  | CHAF1B |  |  |
|  |  |  | CCT6A |  |  |
|  |  |  | IFFO1 |  |  |
|  |  |  | CUL4B |  |  |
|  |  |  | DDX54 |  |  |
|  |  |  | C11orf24 |  |  |
|  |  |  | TMEM44 |  |  |
|  |  |  | SYS1 |  |  |
|  |  |  | TGM2 |  |  |
|  |  |  | TMEM147 |  |  |
|  |  |  | TMCO3 |  |  |
|  |  |  | PKMYT1 |  |  |
|  |  |  | KIAA0232 |  |  |
|  |  |  | PAPOLG |  |  |
|  |  |  | ZBTB5 |  |  |
|  |  |  | ITGB3 |  |  |
|  |  |  | LOC81691 |  |  |
|  |  |  | KLK10 |  |  |
|  |  |  | HEIH |  |  |
|  |  |  | SPCS3 |  |  |
|  |  |  | GPN1 |  |  |
|  |  |  | CRELD1 |  |  |
|  |  |  | GAPDHP62 |  |  |
|  |  |  | FSTL1 |  |  |
|  |  |  | PRAC1 |  |  |
|  |  |  | RASL12 |  |  |
|  |  |  | ROPN1L |  |  |
|  |  |  | FBXW4 |  |  |
|  |  |  | ADSL |  |  |
|  |  |  | WRNIP1 |  |  |
|  |  |  | CNOT4 |  |  |
|  |  |  | MVB12B |  |  |
|  |  |  | NID1 |  |  |
|  |  |  | VIMP |  |  |
|  |  |  | ELK4 |  |  |
|  |  |  | HDAC5 |  |  |
|  |  |  | DUSP18 |  |  |
|  |  |  | SLIT3 |  |  |
|  |  |  | DARS |  |  |
|  |  |  | MORN5 |  |  |
|  |  |  | TRH |  |  |
|  |  |  | RPGRIP1L |  |  |
|  |  |  | MRPL15 |  |  |
|  |  |  | DPM2 |  |  |
|  |  |  | JUP |  |  |
|  |  |  | AKR7L |  |  |
|  |  |  | CDC123 |  |  |
|  |  |  | CALU |  |  |
|  |  |  | HSD11B2 |  |  |
|  |  |  | PGK1 |  |  |
|  |  |  | TPD52 |  |  |
|  |  |  | LOC100129034 |  |  |
|  |  |  | ATAD1 |  |  |
|  |  |  | NIN |  |  |
|  |  |  | BCL7C |  |  |
|  |  |  | NCDN |  |  |
|  |  |  | BTN3A2 |  |  |
|  |  |  | TUBGCP5 |  |  |
|  |  |  | USP54 |  |  |
|  |  |  | TMEM222 |  |  |
|  |  |  | POLR2H |  |  |
|  |  |  | DDAH1 |  |  |
|  |  |  | C18orf21 |  |  |
|  |  |  | TREX1 |  |  |
|  |  |  | ZW10 |  |  |
|  |  |  | DGCR14 |  |  |
|  |  |  | PLCB3 |  |  |
|  |  |  | SLC39A14 |  |  |
|  |  |  | TROVE2 |  |  |
|  |  |  | MICAL1 |  |  |
|  |  |  | KRT14 |  |  |
|  |  |  | TRAPPC2 |  |  |
|  |  |  | NEK9 |  |  |
|  |  |  | HLA-DQA2 |  |  |
|  |  |  | STOML1 |  |  |
|  |  |  | IDH1 |  |  |
|  |  |  | KIAA0930 |  |  |
|  |  |  | C11orf63 |  |  |
|  |  |  | C16orf70 |  |  |
|  |  |  | CPSF3 |  |  |
|  |  |  | CCDC17 |  |  |
|  |  |  | PRIM1 |  |  |
|  |  |  | SLC2A10 |  |  |
|  |  |  | FBLIM1 |  |  |
|  |  |  | INTS9 |  |  |
|  |  |  | NUP210 |  |  |
|  |  |  | ZNF585A |  |  |
|  |  |  | TMEM59 |  |  |
|  |  |  | PBDC1 |  |  |
|  |  |  | TSC22D2 |  |  |
|  |  |  | MECP2 |  |  |
|  |  |  | SIN3A |  |  |
|  |  |  | CYFIP1 |  |  |
|  |  |  | ILDR1 |  |  |
|  |  |  | CKS2 |  |  |
|  |  |  | FBXL17 |  |  |
|  |  |  | SNX5 |  |  |
|  |  |  | NINJ1 |  |  |
|  |  |  | VWA8 |  |  |
|  |  |  | NUP98 |  |  |
|  |  |  | FTO |  |  |
|  |  |  | TMEM139 |  |  |
|  |  |  | ABHD12 |  |  |
|  |  |  | ZNF25 |  |  |
|  |  |  | EIF3E |  |  |
|  |  |  | GUSBP11 |  |  |
|  |  |  | VSIG2 |  |  |
|  |  |  | PHLDB3 |  |  |
|  |  |  | MAEA |  |  |
|  |  |  | PIK3R2 |  |  |
|  |  |  | SGCB |  |  |
|  |  |  | MANF |  |  |
|  |  |  | SEC23A |  |  |
|  |  |  | SLC22A5 |  |  |
|  |  |  | RSPH10B |  |  |
|  |  |  | CCDC107 |  |  |
|  |  |  | NQO2 |  |  |
|  |  |  | NBAS |  |  |
|  |  |  | ARAF |  |  |
|  |  |  | CD99L2 |  |  |
|  |  |  | BAGE4 |  |  |
|  |  |  | NAA30 |  |  |
|  |  |  | HIPK3 |  |  |
|  |  |  | ANKRD39 |  |  |
|  |  |  | TIMMDC1 |  |  |
|  |  |  | PUDP |  |  |
|  |  |  | MAN2A1 |  |  |
|  |  |  | LRRC59 |  |  |
|  |  |  | NRDE2 |  |  |
|  |  |  | PIN1P1 |  |  |
|  |  |  | TMEM101 |  |  |
|  |  |  | DNAJA1 |  |  |
|  |  |  | NOSTRIN |  |  |
|  |  |  | SEC11C |  |  |
|  |  |  | GCNT3 |  |  |
|  |  |  | LRRC4 |  |  |
|  |  |  | RDH10 |  |  |
|  |  |  | CENPL |  |  |
|  |  |  | PRKCQ |  |  |
|  |  |  | DHCR7 |  |  |
|  |  |  | SRRD |  |  |
|  |  |  | SYT6 |  |  |
|  |  |  | TCTN3 |  |  |
|  |  |  | ECHDC1 |  |  |
|  |  |  | ZNF589 |  |  |
|  |  |  | PMEPA1 |  |  |
|  |  |  | CCDC97 |  |  |
|  |  |  | MAP4K5 |  |  |
|  |  |  | MANEAL |  |  |
|  |  |  | ZNF70 |  |  |
|  |  |  | DLC1 |  |  |
|  |  |  | CTSC |  |  |
|  |  |  | IL6ST |  |  |
|  |  |  | ANAPC5 |  |  |
|  |  |  | ERCC2 |  |  |
|  |  |  | DPEP3 |  |  |
|  |  |  | SNHG1 |  |  |
|  |  |  | EIF4EBP2 |  |  |
|  |  |  | ITGB1BP1 |  |  |
|  |  |  | LSM5 |  |  |
|  |  |  | CCNB1 |  |  |
|  |  |  | C17orf75 |  |  |
|  |  |  | HSD17B7 |  |  |
|  |  |  | PIAS1 |  |  |
|  |  |  | TICRR |  |  |
|  |  |  | POTEF |  |  |
|  |  |  | EXOC7 |  |  |
|  |  |  | NSDHL |  |  |
|  |  |  | ZGPAT |  |  |
|  |  |  | OTUD7B |  |  |
|  |  |  | FOXN3 |  |  |
|  |  |  | TPD52L1 |  |  |
|  |  |  | CDCA8 |  |  |
|  |  |  | ANKRD2 |  |  |
|  |  |  | PRDM15 |  |  |
|  |  |  | TOP2A |  |  |
|  |  |  | ADGRG2 |  |  |
|  |  |  | PIGZ |  |  |
|  |  |  | MYD88 |  |  |
|  |  |  | MRPS14 |  |  |
|  |  |  | NOL12 |  |  |
|  |  |  | PSMB8 |  |  |
|  |  |  | ARL3 |  |  |
|  |  |  | MRAP2 |  |  |
|  |  |  | COPS5 |  |  |
|  |  |  | GSTM3 |  |  |
|  |  |  | ZNF761 |  |  |
|  |  |  | MDM2 |  |  |
|  |  |  | AMOT |  |  |
|  |  |  | ATP13A2 |  |  |
|  |  |  | ZNF652 |  |  |
|  |  |  | PPDPF |  |  |
|  |  |  | C11orf95 |  |  |
|  |  |  | RRP9 |  |  |
|  |  |  | GINS4 |  |  |
|  |  |  | PECR |  |  |
|  |  |  | DOK7 |  |  |
|  |  |  | U2AF1L4 |  |  |
|  |  |  | WDR19 |  |  |
|  |  |  | PRKAB1 |  |  |
|  |  |  | PLCB4 |  |  |
|  |  |  | MT1A |  |  |
|  |  |  | TMEM161B |  |  |
|  |  |  | SECISBP2 |  |  |
|  |  |  | SCARA3 |  |  |
|  |  |  | PRH1-PRR4 |  |  |
|  |  |  | ADIRF |  |  |
|  |  |  | POSTN |  |  |
|  |  |  | RNF24 |  |  |
|  |  |  | UQCC1 |  |  |
|  |  |  | PHF14 |  |  |
|  |  |  | SP5 |  |  |
|  |  |  | TRIM65 |  |  |
|  |  |  | KIF7 |  |  |
|  |  |  | ULK1 |  |  |
|  |  |  | GINS1 |  |  |
|  |  |  | SCRN3 |  |  |
|  |  |  | NAPSA |  |  |
|  |  |  | PRSS30P |  |  |
|  |  |  | CHDH |  |  |
|  |  |  | MKL1 |  |  |
|  |  |  | ZSWIM7 |  |  |
|  |  |  | MKLN1 |  |  |
|  |  |  | LOC101927204 |  |  |
|  |  |  | WRB |  |  |
|  |  |  | YDJC |  |  |
|  |  |  | USP5 |  |  |
|  |  |  | FAM76B |  |  |
|  |  |  | RIBC2 |  |  |
|  |  |  | NPTX2 |  |  |
|  |  |  | FDX1L |  |  |
|  |  |  | CYB5D2 |  |  |
|  |  |  | LOC107133515 |  |  |
|  |  |  | METTL14 |  |  |
|  |  |  | GSTCD |  |  |
|  |  |  | LY6K |  |  |
|  |  |  | RUNX1 |  |  |
|  |  |  | CEP57L1 |  |  |
|  |  |  | COX7A2 |  |  |
|  |  |  | NSUN5P2 |  |  |
|  |  |  | MT1X |  |  |
|  |  |  | EBAG9 |  |  |
|  |  |  | ZNF773 |  |  |
|  |  |  | KIAA1661 |  |  |
|  |  |  | NEIL2 |  |  |
|  |  |  | FAM183A |  |  |
|  |  |  | KHNYN |  |  |
|  |  |  | ARAP1 |  |  |
|  |  |  | MSRB2 |  |  |
|  |  |  | MIPEP |  |  |
|  |  |  | C15orf39 |  |  |
|  |  |  | SPTSSA |  |  |
|  |  |  | STAT5A |  |  |
|  |  |  | CREB1 |  |  |
|  |  |  | CDK5RAP2 |  |  |
|  |  |  | TMSB15A |  |  |
|  |  |  | CKAP2 |  |  |
|  |  |  | UPF2 |  |  |
|  |  |  | TMEM69 |  |  |
|  |  |  | KRT33A |  |  |
|  |  |  | SNUPN |  |  |
|  |  |  | CEP350 |  |  |
|  |  |  | DRC3 |  |  |
|  |  |  | RBCK1 |  |  |
|  |  |  | SETD1B |  |  |
|  |  |  | SPIN3 |  |  |
|  |  |  | BTBD6 |  |  |
|  |  |  | ALG3 |  |  |
|  |  |  | ZNF638 |  |  |
|  |  |  | B4GALT6 |  |  |
|  |  |  | KRBA1 |  |  |
|  |  |  | UBE2A |  |  |
|  |  |  | DCAF10 |  |  |
|  |  |  | MGST2 |  |  |
|  |  |  | OPA3 |  |  |
|  |  |  | LMAN1 |  |  |
|  |  |  | SNAPC4 |  |  |
|  |  |  | ERBB3 |  |  |
|  |  |  | NECTIN2 |  |  |
|  |  |  | AASDH |  |  |
|  |  |  | DACT1 |  |  |
|  |  |  | ZNRF3 |  |  |
|  |  |  | OSTCP1 |  |  |
|  |  |  | IWS1 |  |  |
|  |  |  | MBNL2 |  |  |
|  |  |  | GRIP1 |  |  |
|  |  |  | NECAP2 |  |  |
|  |  |  | WDR73 |  |  |
|  |  |  | TNFAIP8L1 |  |  |
|  |  |  | SSTR1 |  |  |
|  |  |  | ABR |  |  |
|  |  |  | TMEM192 |  |  |
|  |  |  | TP73-AS1 |  |  |
|  |  |  | BUB1B |  |  |
|  |  |  | WASH2P |  |  |
|  |  |  | TMCO1 |  |  |
|  |  |  | TSPAN4 |  |  |
|  |  |  | RPS11P6 |  |  |
|  |  |  | CRBN |  |  |
|  |  |  | MRPS24 |  |  |
|  |  |  | VPS37B |  |  |
|  |  |  | LMNB2 |  |  |
|  |  |  | ZNF580 |  |  |
|  |  |  | FBXL15 |  |  |
|  |  |  | BCL2 |  |  |
|  |  |  | ABCB10 |  |  |
|  |  |  | ESR1 |  |  |
|  |  |  | CCT2 |  |  |
|  |  |  | TSC2 |  |  |
|  |  |  | PRADC1 |  |  |
|  |  |  | ATG7 |  |  |
|  |  |  | PSMC1 |  |  |
|  |  |  | NHLRC2 |  |  |
|  |  |  | IDH3G |  |  |
|  |  |  | RBPJ |  |  |
|  |  |  | KIF11 |  |  |
|  |  |  | ZCCHC14 |  |  |
|  |  |  | PLPP2 |  |  |
|  |  |  | BAG3 |  |  |
|  |  |  | TMEM119 |  |  |
|  |  |  | AK3 |  |  |
|  |  |  | CYB561D2 |  |  |
|  |  |  | UBE2D4 |  |  |
|  |  |  | MED19 |  |  |
|  |  |  | SNAPIN |  |  |
|  |  |  | SNAPC5 |  |  |
|  |  |  | STAMBP |  |  |
|  |  |  | HSPB8 |  |  |
|  |  |  | KRIT1 |  |  |
|  |  |  | ZNF354C |  |  |
|  |  |  | C1orf168 |  |  |
|  |  |  | ANAPC7 |  |  |
|  |  |  | STK24 |  |  |
|  |  |  | OSBPL2 |  |  |
|  |  |  | FAM19A5 |  |  |
|  |  |  | ZNF552 |  |  |
|  |  |  | ACSL5 |  |  |
|  |  |  | ATG4B |  |  |
|  |  |  | DBP |  |  |
|  |  |  | TBC1D16 |  |  |
|  |  |  | ATP6V0B |  |  |
|  |  |  | KCTD11 |  |  |
|  |  |  | LOC729080 |  |  |
|  |  |  | NARS2 |  |  |
|  |  |  | ZNF681 |  |  |
|  |  |  | MGAT3 |  |  |
|  |  |  | MRPS10 |  |  |
|  |  |  | VAPA |  |  |
|  |  |  | ACD |  |  |
|  |  |  | OPN3 |  |  |
|  |  |  | NDEL1 |  |  |
|  |  |  | EIF5 |  |  |
|  |  |  | ZNF682 |  |  |
|  |  |  | CDYL |  |  |
|  |  |  | IAH1 |  |  |
|  |  |  | EIF2B5 |  |  |
|  |  |  | EYA2 |  |  |
|  |  |  | PHLDA1 |  |  |
|  |  |  | NDUFC1 |  |  |
|  |  |  | LINC00304 |  |  |
|  |  |  | PRKCE |  |  |
|  |  |  | EP400 |  |  |
|  |  |  | SH3BGRL3 |  |  |
|  |  |  | ZNF544 |  |  |
|  |  |  | FZD5 |  |  |
|  |  |  | COA5 |  |  |
|  |  |  | ANO10 |  |  |
|  |  |  | SNX24 |  |  |
|  |  |  | NPRL2 |  |  |
|  |  |  | RAB11FIP4 |  |  |
|  |  |  | CCDC89 |  |  |
|  |  |  | GEN1 |  |  |
|  |  |  | TMEM120A |  |  |
|  |  |  | NAMPT |  |  |
|  |  |  | LOC286191 |  |  |
|  |  |  | USP34 |  |  |
|  |  |  | C17orf89 |  |  |
|  |  |  | VPS25 |  |  |
|  |  |  | WDR45B |  |  |
|  |  |  | KRTAP4-1 |  |  |
|  |  |  | DHCR24 |  |  |
|  |  |  | PURG |  |  |
|  |  |  | LEMD3 |  |  |
|  |  |  | NECTIN4 |  |  |
|  |  |  | GTF2H4 |  |  |
|  |  |  | HAUS4 |  |  |
|  |  |  | PAX8 |  |  |
|  |  |  | DUSP14 |  |  |
|  |  |  | CCL5 |  |  |
|  |  |  | ROMO1 |  |  |
|  |  |  | TAOK2 |  |  |
|  |  |  | IFT172 |  |  |
|  |  |  | ITPR2 |  |  |
|  |  |  | HIP1R |  |  |
|  |  |  | COMMD2 |  |  |
|  |  |  | PARK7 |  |  |
|  |  |  | RBM48 |  |  |
|  |  |  | RMDN1 |  |  |
|  |  |  | LINC00937 |  |  |
|  |  |  | MUC1 |  |  |
|  |  |  | ITPK1 |  |  |
|  |  |  | JAML |  |  |
|  |  |  | QSOX2 |  |  |
|  |  |  | BCL2A1 |  |  |
|  |  |  | GOLPH3 |  |  |
|  |  |  | DEF8 |  |  |
|  |  |  | LOC105370792 |  |  |
|  |  |  | SNX18 |  |  |
|  |  |  | GMPS |  |  |
|  |  |  | PACSIN1 |  |  |
|  |  |  | MMP7 |  |  |
|  |  |  | POLR3GL |  |  |
|  |  |  | PEX14 |  |  |
|  |  |  | SLC16A1 |  |  |
|  |  |  | LOC100505851 |  |  |
|  |  |  | BTF3P11 |  |  |
|  |  |  | ACOT2 |  |  |
|  |  |  | SMAP2 |  |  |
|  |  |  | TP53INP1 |  |  |
|  |  |  | AP2M1 |  |  |
|  |  |  | SULT1A2 |  |  |
|  |  |  | GTF2A2 |  |  |
|  |  |  | ACOT4 |  |  |
|  |  |  | TRAF7 |  |  |
|  |  |  | UBE2V2 |  |  |
|  |  |  | NOTCH2 |  |  |
|  |  |  | IPMK |  |  |
|  |  |  | ACAT1 |  |  |
|  |  |  | IFI16 |  |  |
|  |  |  | ZNHIT3 |  |  |
|  |  |  | C11orf31 |  |  |
|  |  |  | ADCY4 |  |  |
|  |  |  | ZC3H7B |  |  |
|  |  |  | VWF |  |  |
|  |  |  | ZFP36L2 |  |  |
|  |  |  | LANCL1 |  |  |
|  |  |  | DNAJA4 |  |  |
|  |  |  | FUNDC1 |  |  |
|  |  |  | CCDC18-AS1 |  |  |
|  |  |  | FAM103A1 |  |  |
|  |  |  | NUP155 |  |  |
|  |  |  | PPP1R12B |  |  |
|  |  |  | SHISA6 |  |  |
|  |  |  | ABCB6 |  |  |
|  |  |  | GEMIN7 |  |  |
|  |  |  | MYBL1 |  |  |
|  |  |  | SLC35A5 |  |  |
|  |  |  | SLC38A5 |  |  |
|  |  |  | CCNB2 |  |  |
|  |  |  | POMP |  |  |
|  |  |  | KRT10 |  |  |
|  |  |  | MED14 |  |  |
|  |  |  | CORO1A |  |  |
|  |  |  | TTF2 |  |  |
|  |  |  | TMEM208 |  |  |
|  |  |  | LRTOMT |  |  |
|  |  |  | TADA2B |  |  |
|  |  |  | ZFPL1 |  |  |
|  |  |  | STXBP2 |  |  |
|  |  |  | CYP27A1 |  |  |
|  |  |  | MLXIP |  |  |
|  |  |  | ZNF48 |  |  |
|  |  |  | COG2 |  |  |
|  |  |  | HIST1H2AE |  |  |
|  |  |  | NPC2 |  |  |
|  |  |  | DTX1 |  |  |
|  |  |  | NAA10 |  |  |
|  |  |  | ZDHHC24 |  |  |
|  |  |  | COPZ1 |  |  |
|  |  |  | LOC101928068 |  |  |
|  |  |  | FAM155A |  |  |
|  |  |  | NCBP2 |  |  |
|  |  |  | NES |  |  |
|  |  |  | GPRC5C |  |  |
|  |  |  | ST3GAL5 |  |  |
|  |  |  | PSMG1 |  |  |
|  |  |  | ICE2 |  |  |
|  |  |  | SNX8 |  |  |
|  |  |  | LGALS1 |  |  |
|  |  |  | AGR3 |  |  |
|  |  |  | FAM228A |  |  |
|  |  |  | GLTSCR1L |  |  |
|  |  |  | DNTTIP1 |  |  |
|  |  |  | SAP30L |  |  |
|  |  |  | TICAM1 |  |  |
|  |  |  | TRAM1 |  |  |
|  |  |  | C21orf2 |  |  |
|  |  |  | CCDC24 |  |  |
|  |  |  | SETDB2 |  |  |
|  |  |  | GNL2 |  |  |
|  |  |  | NPR2 |  |  |
|  |  |  | UNC79 |  |  |
|  |  |  | KIAA1652 |  |  |
|  |  |  | TMEM43 |  |  |
|  |  |  | DIS3 |  |  |
|  |  |  | WDR61 |  |  |
|  |  |  | HLA-DPB2 |  |  |
|  |  |  | DET1 |  |  |
|  |  |  | CHP1 |  |  |
|  |  |  | PIEZO1 |  |  |
|  |  |  | FAM20B |  |  |
|  |  |  | CCDC88B |  |  |
|  |  |  | ATP5D |  |  |
|  |  |  | BTG1 |  |  |
|  |  |  | TMEM165 |  |  |
|  |  |  | CCT5 |  |  |
|  |  |  | TPT1P8 |  |  |
|  |  |  | LOC90768 |  |  |
|  |  |  | SCOC |  |  |
|  |  |  | BMP2 |  |  |
|  |  |  | SLC35A2 |  |  |
|  |  |  | MRPL45 |  |  |
|  |  |  | LPAR3 |  |  |
|  |  |  | ARHGDIA |  |  |
|  |  |  | MBD6 |  |  |
|  |  |  | RREB1 |  |  |
|  |  |  | PTGDS |  |  |
|  |  |  | DOLPP1 |  |  |
|  |  |  | GYPC |  |  |
|  |  |  | ANP32A-IT1 |  |  |
|  |  |  | AP1M2 |  |  |
|  |  |  | PMAIP1 |  |  |
|  |  |  | SAMD9 |  |  |
|  |  |  | SMIM19 |  |  |
|  |  |  | PSMB4 |  |  |
|  |  |  | WFDC1 |  |  |
|  |  |  | COL6A3 |  |  |
|  |  |  | L3MBTL4 |  |  |
|  |  |  | NDUFC2 |  |  |
|  |  |  | ACOX3 |  |  |
|  |  |  | INPP5K |  |  |
|  |  |  | UQCRC1 |  |  |
|  |  |  | NT5M |  |  |
|  |  |  | TXNDC9 |  |  |
|  |  |  | COX6C |  |  |
|  |  |  | IFFO2 |  |  |
|  |  |  | CFDP1 |  |  |
|  |  |  | HADHB |  |  |
|  |  |  | ANKRD13D |  |  |
|  |  |  | TPBG |  |  |
|  |  |  | C16orf52 |  |  |
|  |  |  | PPP4R1 |  |  |
|  |  |  | KAT8 |  |  |
|  |  |  | TCTN1 |  |  |
|  |  |  | KLHL5 |  |  |
|  |  |  | DAP3 |  |  |
|  |  |  | ACSS1 |  |  |
|  |  |  | PNMAL1 |  |  |
|  |  |  | NOL7 |  |  |
|  |  |  | ZNF543 |  |  |
|  |  |  | ATP2C1 |  |  |
|  |  |  | NSMCE1 |  |  |
|  |  |  | DKC1 |  |  |
|  |  |  | LOC729680 |  |  |
|  |  |  | ZNF446 |  |  |
|  |  |  | C20orf194 |  |  |
|  |  |  | AURKB |  |  |
|  |  |  | LOC100131929 |  |  |
|  |  |  | VPS52 |  |  |
|  |  |  | TMED10P1 |  |  |
|  |  |  | UCK1 |  |  |
|  |  |  | FLAD1 |  |  |
|  |  |  | VIPR2 |  |  |
|  |  |  | PLAA |  |  |
|  |  |  | CLSTN2 |  |  |
|  |  |  | ZMAT5 |  |  |
|  |  |  | PLCD1 |  |  |
|  |  |  | RNF168 |  |  |
|  |  |  | SEMA3G |  |  |
|  |  |  | USP32P1 |  |  |
|  |  |  | ZNF43 |  |  |
|  |  |  | LINC00649 |  |  |
|  |  |  | ERAP1 |  |  |
|  |  |  | PSMD6 |  |  |
|  |  |  | AMIGO2 |  |  |
|  |  |  | TNFAIP2 |  |  |
|  |  |  | AFTPH |  |  |
|  |  |  | GYG1 |  |  |
|  |  |  | CHAT |  |  |
|  |  |  | FMOD |  |  |
|  |  |  | ZNF146 |  |  |
|  |  |  | MYL12A |  |  |
|  |  |  | THAP6 |  |  |
|  |  |  | TM9SF4 |  |  |
|  |  |  | STRN3 |  |  |
|  |  |  | PARD3B |  |  |
|  |  |  | EXOG |  |  |
|  |  |  | MRPS25 |  |  |
|  |  |  | RAI2 |  |  |
|  |  |  | SUV39H1 |  |  |
|  |  |  | NNT |  |  |
|  |  |  | ARNT |  |  |
|  |  |  | SNRPD2 |  |  |
