# Supplementary material for: Using systems biology and drug repositioning approaches to discover FDA-approved drugs candidates for endometriosis treatment
Source: PLoS One. 2025 Sep 12;20(9):e0330841. doi: 10.1371/journal.pone.0330841 (PMC12431326; doi:10.1371/journal.pone.0330841)
Supplement: S2 Table — (DOCX) [file pone.0330841.s002.docx]

**Table S3**

The list of functional analysis of enriched GO BP terms of common up-regulated DEGs between the FE and IE groups.

| **Number** | **Enrichment FDR** | **nGenes** | **Pathway** |
| --- | --- | --- | --- |
| 1 | 2.48E-14 | 12 | GO:0002222 stimulatory killer cell immunoglobulin-like receptor signaling pathway |
| 2 | 3.60E-09 | 12 | GO:1900452 reg. of long-term synaptic depression |
| 3 | 1.65E-08 | 14 | GO:0002220 innate immune response activating cell surface receptor signaling pathway |
| 4 | 1.65E-08 | 7 | GO:1904470 reg. of endothelin production |
| 5 | 1.65E-08 | 7 | GO:1990775 endothelin production |
| 6 | 1.66E-08 | 14 | GO:0002758 innate immune response-activating signal transduction |
| 7 | 9.64E-08 | 7 | GO:0071929 alpha-tubulin acetylation |
| 8 | 3.76E-07 | 7 | GO:1900453 negative reg. of long-term synaptic depression |
| 9 | 5.24E-07 | 17 | GO:0050805 negative reg. of synaptic transmission |
| 10 | 5.24E-07 | 13 | GO:0060292 long-term synaptic depression |
| 11 | 8.45E-07 | 7 | GO:2000439 positive reg. of monocyte extravasation |
| 12 | 8.45E-07 | 7 | GO:2001200 positive reg. of dendritic cell differentiation |
| 13 | 1.07E-06 | 11 | GO:0048143 astrocyte activation |
| 14 | 1.73E-06 | 13 | GO:0014002 astrocyte development |
| 15 | 1.73E-06 | 7 | GO:0150099 neuron-glial cell signaling |
| 16 | 3.83E-06 | 7 | GO:0150003 reg. of spontaneous synaptic transmission |
| 17 | 6.61E-06 | 7 | GO:0009757 hexose mediated signaling |
| 18 | 6.61E-06 | 7 | GO:0010182 sugar mediated signaling pathway |
| 19 | 6.61E-06 | 7 | GO:0010255 glucose mediated signaling pathway |
| 20 | 6.61E-06 | 7 | GO:1902961 positive reg. of aspartic-type endopeptidase activity involved in amyloid precursor prote |
| 21 | 1.17E-05 | 14 | GO:0002218 activation of innate immune response |
| 22 | 1.17E-05 | 7 | GO:0035696 monocyte extravasation |
| 23 | 1.17E-05 | 7 | GO:1905247 positive reg. of aspartic-type peptidase activity |
| 24 | 1.86E-05 | 13 | GO:0001774 microglial cell activation |
| 25 | 1.92E-05 | 7 | GO:0009756 carbohydrate mediated signaling |
| 26 | 2.10E-05 | 18 | GO:0021782 glial cell development |
| 27 | 2.35E-05 | 9 | GO:1903523 negative reg. of blood circulation |
| 28 | 2.59E-05 | 15 | GO:0048708 astrocyte differentiation |
| 29 | 2.64E-05 | 13 | GO:0002269 leukocyte activation involved in inflammatory response |
| 30 | 4.08E-05 | 8 | GO:0002693 positive reg. of cellular extravasation |
| 31 | 4.08E-05 | 13 | GO:0061900 glial cell activation |
| 32 | 4.28E-05 | 25 | GO:1903522 reg. of blood circulation |
| 33 | 4.79E-05 | 179 | GO:0048583 reg. of response to stimulus |
| 34 | 5.54E-05 | 106 | GO:0048468 cell development |
| 35 | 5.54E-05 | 14 | GO:0150076 neuroinflammatory response |
| 36 | 9.12E-05 | 7 | GO:0098814 spontaneous synaptic transmission |
| 37 | 9.12E-05 | 10 | GO:1904646 cellular response to amyloid-beta |
| 38 | 0.000125915 | 8 | GO:0045056 transcytosis |
| 39 | 0.000137022 | 64 | GO:0048666 neuron development |
| 40 | 0.000284042 | 9 | GO:0034114 reg. of heterotypic cell-cell adhesion |
| 41 | 0.000293697 | 5 | GO:0060023 soft palate development |
| 42 | 0.000293697 | 78 | GO:0120036 plasma membrane bounded cell projection organization |
| 43 | 0.000343432 | 8 | GO:0034116 positive reg. of heterotypic cell-cell adhesion |
| 44 | 0.000405002 | 79 | GO:0030030 cell projection organization |
| 45 | 0.000405002 | 4 | GO:1903892 negative reg. of ATF6-mediated unfolded protein response |
| 46 | 0.000415467 | 10 | GO:1900271 reg. of long-term synaptic potentiation |
| 47 | 0.000415467 | 10 | GO:1904645 response to amyloid-beta |
| 48 | 0.000467256 | 128 | GO:0007166 cell surface receptor signaling pathway |
| 49 | 0.000472996 | 43 | GO:0040008 reg. of growth |
| 50 | 0.000536733 | 20 | GO:0048167 reg. of synaptic plasticity |
| 51 | 0.000607813 | 8 | GO:1900745 positive reg. of p38MAPK cascade |
| 52 | 0.000931292 | 54 | GO:0070925 organelle assembly |
| 53 | 0.001281261 | 38 | GO:0003013 circulatory system proc. |
| 54 | 0.001443231 | 34 | GO:0008015 blood circulation |
| 55 | 0.001443231 | 72 | GO:0010648 negative reg. of cell communication |
| 56 | 0.001538493 | 72 | GO:0023057 negative reg. of signaling |
| 57 | 0.001538493 | 39 | GO:0051345 positive reg. of hydrolase activity |
| 58 | 0.001545202 | 173 | GO:0019219 reg. of nucleobase-containing compound metabolic proc. |
| 59 | 0.001658425 | 18 | GO:0010950 positive reg. of endopeptidase activity |
| 60 | 0.001682511 | 7 | GO:0032693 negative reg. of interleukin-10 production |
| 61 | 0.001682511 | 73 | GO:0048699 generation of neurons |
| 62 | 0.001821151 | 132 | GO:0009966 reg. of signal transduction |
| 63 | 0.001837984 | 12 | GO:0010232 vascular transport |
| 64 | 0.001837984 | 27 | GO:0060271 cilium assembly |
| 65 | 0.001837984 | 12 | GO:0150104 transport across blood-brain barrier |
| 66 | 0.001838218 | 3 | GO:0039020 pronephric nephron tubule development |
| 67 | 0.001838218 | 3 | GO:0072114 pronephros morphogenesis |
| 68 | 0.001838218 | 3 | GO:1905687 reg. of diacylglycerol kinase activity |
| 69 | 0.001889018 | 178 | GO:0048731 system development |
| 70 | 0.001903583 | 53 | GO:0040007 growth |
| 71 | 0.001985233 | 145 | GO:0023051 reg. of signaling |
| 72 | 0.002227813 | 12 | GO:0060291 long-term synaptic potentiation |
| 73 | 0.002335839 | 176 | GO:0048869 cellular developmental proc. |
| 74 | 0.002516672 | 175 | GO:0030154 cell differentiation |
| 75 | 0.002532891 | 24 | GO:0048638 reg. of developmental growth |
| 76 | 0.003024613 | 7 | GO:0007214 gamma-aminobutyric acid signaling pathway |
| 77 | 0.003024613 | 20 | GO:0010001 glial cell differentiation |
| 78 | 0.003024613 | 80 | GO:0022008 neurogenesis |
| 79 | 0.003024613 | 172 | GO:0031326 reg. of cellular biosynthetic proc. |
| 80 | 0.003024613 | 155 | GO:0032774 RNA biosynthetic proc. |
| 81 | 0.003024613 | 7 | GO:0042104 positive reg. of activated T cell proliferation |
| 82 | 0.003024613 | 47 | GO:0042327 positive reg. of phosphorylation |
| 83 | 0.003024613 | 4 | GO:1904428 negative reg. of tubulin deacetylation |
| 84 | 0.003123281 | 25 | GO:0030198 extracellular matrix organization |
| 85 | 0.003168332 | 154 | GO:0006351 transcription DNA-templated |
| 86 | 0.003168332 | 143 | GO:0010646 reg. of cell communication |
| 87 | 0.003168332 | 25 | GO:0043062 extracellular structure organization |
| 88 | 0.003168332 | 154 | GO:0097659 nucleic acid-templated transcription |
| 89 | 0.00334576 | 16 | GO:0050806 positive reg. of synaptic transmission |
| 90 | 0.00334576 | 43 | GO:0080135 reg. of cellular response to stress |
| 91 | 0.003443555 | 25 | GO:0045229 external encapsulating structure organization |
| 92 | 0.003519247 | 149 | GO:0006355 reg. of transcription DNA-templated |
| 93 | 0.003519247 | 159 | GO:0065008 reg. of biological quality |
| 94 | 0.003519247 | 149 | GO:1903506 reg. of nucleic acid-templated transcription |
| 95 | 0.003530964 | 18 | GO:0010952 positive reg. of peptidase activity |
| 96 | 0.003616762 | 13 | GO:0002709 reg. of T cell mediated immunity |
| 97 | 0.003616762 | 80 | GO:0007267 cell-cell signaling |
| 98 | 0.003616762 | 14 | GO:0035710 CD4-positive alpha-beta T cell activation |
| 99 | 0.003616762 | 159 | GO:0051252 reg. of RNA metabolic proc. |
| 100 | 0.003616762 | 71 | GO:0080134 reg. of response to stress |
| 101 | 0.003616762 | 149 | GO:2001141 reg. of RNA biosynthetic proc. |
| 102 | 0.003678063 | 9 | GO:0032735 positive reg. of interleukin-12 production |
| 103 | 0.003717808 | 68 | GO:0030182 neuron differentiation |
| 104 | 0.003888123 | 27 | GO:0044782 cilium organization |
| 105 | 0.003888123 | 4 | GO:0048793 pronephros development |
| 106 | 0.003888123 | 4 | GO:0072386 plus-end-directed organelle transport along microtubule |
| 107 | 0.003926209 | 165 | GO:0010556 reg. of macromolecule biosynthetic proc. |
| 108 | 0.004504109 | 9 | GO:0051932 synaptic transmission GABAergic |
| 109 | 0.004517651 | 3 | GO:0039019 pronephric nephron development |
| 110 | 0.005505441 | 34 | GO:0044057 reg. of system proc. |
| 111 | 0.005650177 | 13 | GO:0042116 macrophage activation |
| 112 | 0.005703933 | 108 | GO:0007399 nervous system development |
| 113 | 0.005703933 | 37 | GO:0007610 behavior |
| 114 | 0.005918263 | 110 | GO:0050793 reg. of developmental proc. |
| 115 | 0.006446001 | 55 | GO:0051336 reg. of hydrolase activity |
| 116 | 0.007299231 | 19 | GO:0006473 protein acetylation |
| 117 | 0.007489611 | 21 | GO:0043543 protein acylation |
| 118 | 0.007627754 | 28 | GO:0001558 reg. of cell growth |
| 119 | 0.007627754 | 81 | GO:1902531 reg. of intracellular signal transduction |
| 120 | 0.007805732 | 16 | GO:2001257 reg. of cation channel activity |
| 121 | 0.008135315 | 20 | GO:0003018 vascular proc. in circulatory system |
| 122 | 0.008265128 | 34 | GO:0051272 positive reg. of cellular component movement |
| 123 | 0.008265128 | 6 | GO:0062009 secondary palate development |
| 124 | 0.00829702 | 15 | GO:0002687 positive reg. of leukocyte migration |
| 125 | 0.008617554 | 60 | GO:0043085 positive reg. of catalytic activity |
| 126 | 0.009026898 | 111 | GO:0006357 reg. of transcription by RNA polymerase II |
| 127 | 0.009026898 | 48 | GO:0010562 positive reg. of phosphorus metabolic proc. |
| 128 | 0.009026898 | 23 | GO:0042063 gliogenesis |
| 129 | 0.009032705 | 54 | GO:0007417 central nervous system development |
| 130 | 0.009032705 | 3 | GO:0021563 glossopharyngeal nerve development |
| 131 | 0.009032705 | 8 | GO:0050798 activated T cell proliferation |
| 132 | 0.009032705 | 3 | GO:0071211 protein targeting to vacuole involved in autophagy |
| 133 | 0.009032705 | 3 | GO:1902988 neurofibrillary tangle assembly |
| 134 | 0.009032705 | 11 | GO:2000514 reg. of CD4-positive alpha-beta T cell activation |
| 135 | 0.009032705 | 3 | GO:2000660 negative reg. of interleukin-1-mediated signaling pathway |
| 136 | 0.009114368 | 36 | GO:0048589 developmental growth |
| 137 | 0.009197582 | 16 | GO:0009746 response to hexose |
| 138 | 0.009504709 | 23 | GO:0060249 anatomical structure homeostasis |
| 139 | 0.009529779 | 27 | GO:0003012 muscle system proc. |
| 140 | 0.009617462 | 114 | GO:0035556 intracellular signal transduction |
| 141 | 0.01010544 | 27 | GO:0042391 reg. of membrane potential |
| 142 | 0.010424615 | 32 | GO:0030335 positive reg. of cell migration |
| 143 | 0.010482763 | 4 | GO:0010917 negative reg. of mitochondrial membrane potential |
| 144 | 0.010482763 | 4 | GO:0036500 ATF6-mediated unfolded protein response |
| 145 | 0.010482763 | 33 | GO:2000147 positive reg. of cell motility |
| 146 | 0.010567754 | 41 | GO:0001934 positive reg. of protein phosphorylation |
| 147 | 0.010567754 | 8 | GO:0038066 p38MAPK cascade |
| 148 | 0.010653851 | 20 | GO:0001894 tissue homeostasis |
| 149 | 0.011230322 | 23 | GO:0006936 muscle contraction |
| 150 | 0.011970945 | 33 | GO:0120031 plasma membrane bounded cell projection assembly |
| 151 | 0.012415953 | 5 | GO:0010288 response to lead ion |
| 152 | 0.012415953 | 30 | GO:0016049 cell growth |
| 153 | 0.012828267 | 20 | GO:0007611 learning or memory |
| 154 | 0.012828267 | 10 | GO:0032731 positive reg. of interleukin-1 beta production |
| 155 | 0.012828267 | 16 | GO:0034284 response to monosaccharide |
| 156 | 0.013716985 | 70 | GO:0007010 cytoskeleton organization |
| 157 | 0.013835528 | 167 | GO:0019438 aromatic compound biosynthetic proc. |
| 158 | 0.013970574 | 8 | GO:0044458 motile cilium assembly |
| 159 | 0.014408483 | 9 | GO:0030199 collagen fibril organization |
| 160 | 0.015031971 | 3 | GO:0090258 negative reg. of mitochondrial fission |
| 161 | 0.01507509 | 81 | GO:0042592 homeostatic proc. |
| 162 | 0.015385432 | 33 | GO:0040017 positive reg. of locomotion |
| 163 | 0.015839544 | 16 | GO:0006475 internal protein amino acid acetylation |
| 164 | 0.016913009 | 9 | GO:0034113 heterotypic cell-cell adhesion |
| 165 | 0.016955578 | 33 | GO:0030031 cell projection assembly |
| 166 | 0.018270822 | 73 | GO:0045595 reg. of cell differentiation |
| 167 | 0.018270822 | 10 | GO:0071677 positive reg. of mononuclear cell migration |
| 168 | 0.01828302 | 18 | GO:0002833 positive reg. of response to biotic stimulus |
| 169 | 0.018490985 | 13 | GO:0002456 T cell mediated immunity |
| 170 | 0.018490985 | 22 | GO:0050890 cognition |
| 171 | 0.018823307 | 18 | GO:0003015 heart proc. |
| 172 | 0.018925584 | 163 | GO:0034654 nucleobase-containing compound biosynthetic proc. |
| 173 | 0.020228611 | 16 | GO:0002822 reg. of adaptive immune response based on somatic recombination of immune receptors built |
| 174 | 0.020805853 | 12 | GO:0032874 positive reg. of stress-activated MAPK cascade |
| 175 | 0.020894237 | 39 | GO:0099537 trans-synaptic signaling |
| 176 | 0.021280206 | 72 | GO:0006468 protein phosphorylation |
| 177 | 0.021451839 | 165 | GO:0018130 heterocycle biosynthetic proc. |
| 178 | 0.021688613 | 112 | GO:0009653 anatomical structure morphogenesis |
| 179 | 0.021724545 | 59 | GO:0042325 reg. of phosphorylation |
| 180 | 0.02198002 | 4 | GO:1990440 positive reg. of transcription from RNA polymerase II promoter in response to endoplasmic |
| 181 | 0.022476449 | 27 | GO:0016055 Wnt signaling pathway |
| 182 | 0.022702748 | 12 | GO:0070304 positive reg. of stress-activated protein kinase signaling cascade |
| 183 | 0.022770235 | 8 | GO:0097028 dendritic cell differentiation |
| 184 | 0.023571986 | 27 | GO:0198738 cell-cell signaling by wnt |
| 185 | 0.023683492 | 25 | GO:0010959 reg. of metal ion transport |
| 186 | 0.023837627 | 14 | GO:1990138 neuron projection extension |
| 187 | 0.024835432 | 136 | GO:0031325 positive reg. of cellular metabolic proc. |
| 188 | 0.026046967 | 7 | GO:1902991 reg. of amyloid precursor protein catabolic proc. |
| 189 | 0.026693419 | 47 | GO:0031175 neuron projection development |
| 190 | 0.026693419 | 9 | GO:0032615 interleukin-12 production |
| 191 | 0.026693419 | 5 | GO:0070498 interleukin-1-mediated signaling pathway |
| 192 | 0.026693419 | 31 | GO:1905114 cell surface receptor signaling pathway involved in cell-cell signaling |
| 193 | 0.026976551 | 17 | GO:0060047 heart contraction |
| 194 | 0.027067801 | 22 | GO:0070371 ERK1 and ERK2 cascade |
| 195 | 0.027639314 | 33 | GO:0006897 endocytosis |
| 196 | 0.027639314 | 38 | GO:0007268 chemical synaptic transmission |
| 197 | 0.027639314 | 64 | GO:0019220 reg. of phosphate metabolic proc. |
| 198 | 0.027639314 | 15 | GO:0045089 positive reg. of innate immune response |
| 199 | 0.027639314 | 64 | GO:0051174 reg. of phosphorus metabolic proc. |
| 200 | 0.027639314 | 38 | GO:0098916 anterograde trans-synaptic signaling |
| 201 | 0.029784094 | 35 | GO:0000226 microtubule cytoskeleton organization |
| 202 | 0.029860108 | 169 | GO:1901362 organic cyclic compound biosynthetic proc. |
| 203 | 0.030001171 | 5 | GO:0007288 sperm axoneme assembly |
| 204 | 0.030016404 | 18 | GO:0032412 reg. of ion transmembrane transporter activity |
| 205 | 0.03082855 | 12 | GO:0042102 positive reg. of T cell proliferation |
| 206 | 0.031152621 | 3 | GO:0038089 positive reg. of cell migration by vascular endothelial growth factor signaling pathway |
| 207 | 0.031428743 | 11 | GO:0032755 positive reg. of interleukin-6 production |
| 208 | 0.031526711 | 8 | GO:0001914 reg. of T cell mediated cytotoxicity |
| 209 | 0.031526711 | 8 | GO:0032613 interleukin-10 production |
| 210 | 0.031526711 | 8 | GO:0032653 reg. of interleukin-10 production |
| 211 | 0.031627538 | 128 | GO:0051173 positive reg. of nitrogen compound metabolic proc. |
| 212 | 0.031668872 | 6 | GO:1901385 reg. of voltage-gated calcium channel activity |
| 213 | 0.031780137 | 16 | GO:0009743 response to carbohydrate |
| 214 | 0.032334834 | 40 | GO:0000165 MAPK cascade |
| 215 | 0.03250253 | 15 | GO:0008016 reg. of heart contraction |
| 216 | 0.032665717 | 17 | GO:0007160 cell-matrix adhesion |
| 217 | 0.033931914 | 17 | GO:0051403 stress-activated MAPK cascade |
| 218 | 0.034023502 | 11 | GO:0048675 axon extension |
| 219 | 0.036585282 | 17 | GO:0045926 negative reg. of growth |
| 220 | 0.037556964 | 52 | GO:0001932 reg. of protein phosphorylation |
| 221 | 0.037556964 | 5 | GO:0034063 stress granule assembly |
| 222 | 0.037556964 | 5 | GO:0036003 positive reg. of transcription from RNA polymerase II promoter in response to stress |
| 223 | 0.037556964 | 48 | GO:0050776 reg. of immune response |
| 224 | 0.037556964 | 7 | GO:0050879 multicellular organismal movement |
| 225 | 0.037556964 | 7 | GO:0050881 musculoskeletal movement |
| 226 | 0.037868457 | 10 | GO:0043473 pigmentation |
| 227 | 0.037966451 | 39 | GO:0099536 synaptic signaling |
| 228 | 0.039746359 | 17 | GO:0002685 reg. of leukocyte migration |
| 229 | 0.039746359 | 14 | GO:0009749 response to glucose |
| 230 | 0.039805598 | 139 | GO:0048513 animal organ development |
| 231 | 0.040174379 | 20 | GO:0022604 reg. of cell morphogenesis |
| 232 | 0.040174379 | 44 | GO:0030001 metal ion transport |
| 233 | 0.040339137 | 29 | GO:0032103 positive reg. of response to external stimulus |
| 234 | 0.040339137 | 3 | GO:0048312 intracellular distribution of mitochondria |
| 235 | 0.040339137 | 3 | GO:1902474 positive reg. of protein localization to synapse |
| 236 | 0.0408657 | 16 | GO:0002819 reg. of adaptive immune response |
| 237 | 0.041573508 | 17 | GO:0031098 stress-activated protein kinase signaling cascade |
| 238 | 0.041643445 | 71 | GO:0044093 positive reg. of molecular function |
| 239 | 0.042416339 | 15 | GO:0018394 peptidyl-lysine acetylation |
| 240 | 0.042424511 | 141 | GO:0010604 positive reg. of macromolecule metabolic proc. |
| 241 | 0.042424511 | 48 | GO:0031401 positive reg. of protein modification proc. |
| 242 | 0.043551434 | 26 | GO:0052548 reg. of endopeptidase activity |
| 243 | 0.043949602 | 11 | GO:0032611 interleukin-1 beta production |
| 244 | 0.043949602 | 11 | GO:0032651 reg. of interleukin-1 beta production |
| 245 | 0.044672737 | 6 | GO:0003009 skeletal muscle contraction |
| 246 | 0.045069462 | 9 | GO:1901019 reg. of calcium ion transmembrane transporter activity |
| 247 | 0.046221311 | 5 | GO:0090322 reg. of superoxide metabolic proc. |
| 248 | 0.0476275 | 13 | GO:0070507 reg. of microtubule cytoskeleton organization |
| 249 | 0.048044094 | 20 | GO:0070372 reg. of ERK1 and ERK2 cascade |
| 250 | 0.04825951 | 11 | GO:0071331 cellular response to hexose stimulus |
| 251 | 0.048679993 | 97 | GO:0003008 system proc. |
| 252 | 0.048747458 | 35 | GO:0043408 reg. of MAPK cascade |
| 253 | 0.049022017 | 2 | GO:0006742 NADP catabolic proc. |
| 254 | 0.049022017 | 2 | GO:0021564 vagus nerve development |
| 255 | 0.049022017 | 2 | GO:0030207 chondroitin sulfate catabolic proc. |
| 256 | 0.049022017 | 13 | GO:0030307 positive reg. of cell growth |
| 257 | 0.049022017 | 4 | GO:0031998 reg. of fatty acid beta-oxidation |
| 258 | 0.049022017 | 4 | GO:0048569 post-embryonic animal organ development |
| 259 | 0.049022017 | 9 | GO:0050909 sensory perception of taste |
| 260 | 0.049022017 | 2 | GO:0060083 smooth muscle contraction involved in micturition |
| 261 | 0.049022017 | 2 | GO:0061753 substrate localization to autophagosome |
| 262 | 0.049022017 | 2 | GO:0090107 reg. of high-density lipoprotein particle assembly |
| 263 | 0.049022017 | 2 | GO:0110077 vesicle-mediated intercellular transport |
| 264 | 0.049022017 | 8 | GO:1901224 positive reg. of NIK/NF-kappaB signaling |
| 265 | 0.049022017 | 4 | GO:1902259 reg. of delayed rectifier potassium channel activity |
| 266 | 0.049022017 | 2 | GO:1902336 positive reg. of retinal ganglion cell axon guidance |
| 267 | 0.049022017 | 2 | GO:2000367 reg. of acrosomal vesicle exocytosis |
| 268 | 0.04957738 | 3 | GO:1903748 negative reg. of establishment of protein localization to mitochondrion |
| 269 | 0.04957738 | 3 | GO:1990416 cellular response to brain-derived neurotrophic factor stimulus |
