# Supplementary material for: Using systems biology and drug repositioning approaches to discover FDA-approved drugs candidates for endometriosis treatment
Source: PLoS One. 2025 Sep 12;20(9):e0330841. doi: 10.1371/journal.pone.0330841 (PMC12431326; doi:10.1371/journal.pone.0330841)
Supplement: S3 Table — (DOCX) [file pone.0330841.s003.docx]

**Table S4**

The list of functional analysis of enriched GO MF terms of common up-regulated DEGs between the FE and IE groups.

| **Number** | **Enrichment FDR** | **nGenes** | **Pathway** |
| --- | --- | --- | --- |
| 1 | 9.09E-09 | 7 | GO:0019799 tubulin N-acetyltransferase activity |
| 2 | 9.09E-09 | 7 | GO:0050785 advanced glycation end-product receptor activity |
| 3 | 2.67E-08 | 8 | GO:0004468 lysine N-acetyltransferase activity acting on acetyl phosphate as donor |
| 4 | 1.55E-07 | 7 | GO:0004965 G protein-coupled GABA receptor activity |
| 5 | 2.41E-06 | 81 | GO:0000981 DNA-binding transcription factor activity RNA polymerase II-specific |
| 6 | 4.00E-06 | 82 | GO:0000977 RNA polymerase II transcription regulatory region sequence-specific DNA binding |
| 7 | 5.15E-06 | 82 | GO:0003700 DNA-binding transcription factor activity |
| 8 | 5.19E-06 | 85 | GO:0000976 transcription cis-regulatory region binding |
| 9 | 5.19E-06 | 85 | GO:0001067 transcription regulatory region nucleic acid binding |
| 10 | 5.19E-06 | 92 | GO:0003690 double-stranded DNA binding |
| 11 | 5.19E-06 | 8 | GO:0044548 S100 protein binding |
| 12 | 2.15E-05 | 85 | GO:1990837 sequence-specific double-stranded DNA binding |
| 13 | 2.17E-05 | 89 | GO:0043565 sequence-specific DNA binding |
| 14 | 0.000111296 | 67 | GO:0000978 RNA polymerase II cis-regulatory region sequence-specific DNA binding |
| 15 | 0.000143354 | 188 | GO:0043169 cation binding |
| 16 | 0.000163226 | 67 | GO:0000987 cis-regulatory region sequence-specific DNA binding |
| 17 | 0.000163226 | 13 | GO:0038024 cargo receptor activity |
| 18 | 0.000216476 | 10 | GO:0005044 scavenger receptor activity |
| 19 | 0.000258058 | 95 | GO:0140110 transcription regulator activity |
| 20 | 0.000260154 | 183 | GO:0046872 metal ion binding |
| 21 | 0.000496325 | 18 | GO:0005201 extracellular matrix structural constituent |
| 22 | 0.00057263 | 7 | GO:0016917 GABA receptor activity |
| 23 | 0.00098813 | 12 | GO:0016407 acetyltransferase activity |
| 24 | 0.00098813 | 9 | GO:0034212 peptide N-acetyltransferase activity |
| 25 | 0.00098813 | 3 | GO:0099077 histone-dependent DNA binding |
| 26 | 0.00098813 | 3 | GO:0099609 microtubule lateral binding |
| 27 | 0.001612328 | 8 | GO:0030020 extracellular matrix structural constituent conferring tensile strength |
| 28 | 0.001834353 | 8 | GO:0061733 peptide-lysine-N-acetyltransferase activity |
| 29 | 0.002942559 | 112 | GO:0003677 DNA binding |
| 30 | 0.004773643 | 17 | GO:0005516 calmodulin binding |
| 31 | 0.008057028 | 9 | GO:0008080 N-acetyltransferase activity |
| 32 | 0.009340155 | 5 | GO:0034185 apolipoprotein binding |
| 33 | 0.010749907 | 6 | GO:0051721 protein phosphatase 2A binding |
| 34 | 0.018304574 | 30 | GO:0030695 GTPase regulator activity |
| 35 | 0.018304574 | 30 | GO:0060589 nucleoside-triphosphatase regulator activity |
| 36 | 0.030248616 | 17 | GO:0005085 guanyl-nucleotide exchange factor activity |
| 37 | 0.034867569 | 21 | GO:0001227 DNA-binding transcription repressor activity RNA polymerase II-specific |
| 38 | 0.034867569 | 160 | GO:0003676 nucleic acid binding |
| 39 | 0.037211974 | 8 | GO:0003777 microtubule motor activity |
| 40 | 0.038188983 | 21 | GO:0001217 DNA-binding transcription repressor activity |
| 41 | 0.041097479 | 5 | GO:0051959 dynein light intermediate chain binding |
| 42 | 0.048566492 | 4 | GO:0008569 minus-end-directed microtubule motor activity |
