# Supplementary material for: Using systems biology and drug repositioning approaches to discover FDA-approved drugs candidates for endometriosis treatment
Source: PLoS One. 2025 Sep 12;20(9):e0330841. doi: 10.1371/journal.pone.0330841 (PMC12431326; doi:10.1371/journal.pone.0330841)
Supplement: S4 Table — (DOCX) [file pone.0330841.s004.docx]

**Table S5**

The list of functional analysis of enriched GO CC terms of common up-regulated DEGs between the FE and IE groups.

| **Number** | **Enrichment FDR** | **nGenes** | **Pathway** |
| --- | --- | --- | --- |
| 1 | 9.23E-08 | 7 | GO:1902712 G protein-coupled GABA receptor complex |
| 2 | 2.02E-07 | 7 | GO:0038039 G protein-coupled receptor heterodimeric complex |
| 3 | 5.81E-06 | 7 | GO:0038037 G protein-coupled receptor dimeric complex |
| 4 | 0.000113894 | 109 | GO:0120025 plasma membrane bounded cell projection |
| 5 | 0.000181836 | 112 | GO:0042995 cell projection |
| 6 | 0.000218506 | 5 | GO:0005592 collagen type XI trimer |
| 7 | 0.000218506 | 42 | GO:0098794 postsynapse |
| 8 | 0.00025486 | 6 | GO:0005583 fibrillar collagen trimer |
| 9 | 0.00025486 | 6 | GO:0098643 banded collagen fibril |
| 10 | 0.000435742 | 7 | GO:0098644 complex of collagen trimers |
| 11 | 0.000436438 | 17 | GO:0001650 fibrillar center |
| 12 | 0.000485259 | 7 | GO:1902710 GABA receptor complex |
| 13 | 0.000939438 | 70 | GO:0043005 neuron projection |
| 14 | 0.001114862 | 3 | GO:0045298 tubulin complex |
| 15 | 0.001134046 | 58 | GO:0099081 supramolecular polymer |
| 16 | 0.001628969 | 57 | GO:0099512 supramolecular fiber |
| 17 | 0.002281525 | 68 | GO:0015630 microtubule cytoskeleton |
| 18 | 0.002281525 | 64 | GO:0098590 plasma membrane region |
| 19 | 0.002378794 | 28 | GO:0043235 receptor complex |
| 20 | 0.004826989 | 10 | GO:0005905 clathrin-coated pit |
| 21 | 0.008044577 | 16 | GO:0098802 plasma membrane signaling receptor complex |
| 22 | 0.008044577 | 68 | GO:0099080 supramolecular complex |
| 23 | 0.008313106 | 4 | GO:0005614 interstitial matrix |
| 24 | 0.012526697 | 28 | GO:0005874 microtubule |
| 25 | 0.012583119 | 14 | GO:0098862 cluster of actin-based cell projections |
| 26 | 0.019852306 | 3 | GO:0097418 neurofibrillary tangle |
| 27 | 0.020873184 | 10 | GO:0005581 collagen trimer |
| 28 | 0.025387382 | 37 | GO:0005929 cilium |
| 29 | 0.025387382 | 9 | GO:0016528 sarcoplasm |
| 30 | 0.025387382 | 8 | GO:0016529 sarcoplasmic reticulum |
| 31 | 0.025387382 | 4 | GO:0030673 axolemma |
| 32 | 0.025387382 | 22 | GO:0031253 cell projection membrane |
| 33 | 0.025387382 | 62 | GO:0045202 synapse |
| 34 | 0.025387382 | 15 | GO:0072686 mitotic spindle |
| 35 | 0.029633162 | 20 | GO:0005788 endoplasmic reticulum lumen |
| 36 | 0.029633162 | 13 | GO:0005814 centriole |
| 37 | 0.029633162 | 34 | GO:0030424 axon |
| 38 | 0.029633162 | 31 | GO:0031012 extracellular matrix |
| 39 | 0.029633162 | 21 | GO:0098857 membrane microdomain |
| 40 | 0.029658068 | 31 | GO:0030312 external encapsulating structure |
| 41 | 0.036519675 | 25 | GO:0005819 spindle |
| 42 | 0.042415009 | 8 | GO:0042641 actomyosin |
| 43 | 0.044488728 | 4 | GO:0042827 platelet dense granule |
| 44 | 0.045018468 | 35 | GO:0098797 plasma membrane protein complex |
