# Supplementary material for: Using systems biology and drug repositioning approaches to discover FDA-approved drugs candidates for endometriosis treatment
Source: PLoS One. 2025 Sep 12;20(9):e0330841. doi: 10.1371/journal.pone.0330841 (PMC12431326; doi:10.1371/journal.pone.0330841)
Supplement: S5 Table — (DOCX) [file pone.0330841.s005.docx]

**Table S6**

The list of functional analysis of enriched GO BP terms of common down-regulated DEGs between the FE and IE groups.

| **Number** | **Enrichment FDR** | **nGenes** | **Pathway** |
| --- | --- | --- | --- |
| 1 | 1.13E-19 | 15 | GO:0045653 negative reg. of megakaryocyte differentiation |
| 2 | 1.06E-17 | 14 | GO:0061644 protein localization to CENP-A containing chromatin |
| 3 | 6.02E-17 | 17 | GO:0006336 DNA replication-independent chromatin assembly |
| 4 | 8.40E-17 | 17 | GO:0034724 DNA replication-independent chromatin organization |
| 5 | 2.88E-15 | 16 | GO:0045652 reg. of megakaryocyte differentiation |
| 6 | 2.73E-14 | 15 | GO:0006335 DNA replication-dependent chromatin assembly |
| 7 | 2.73E-14 | 15 | GO:0034723 DNA replication-dependent chromatin organization |
| 8 | 3.99E-13 | 10 | GO:0000379 tRNA-type intron splice site recognition and cleavage |
| 9 | 3.22E-12 | 15 | GO:0071459 protein localization to chromosome centromeric region |
| 10 | 4.13E-12 | 16 | GO:0071168 protein localization to chromatin |
| 11 | 4.24E-12 | 10 | GO:0036149 phosphatidylinositol acyl-chain remodeling |
| 12 | 1.58E-10 | 11 | GO:0021819 layer formation in cerebral cortex |
| 13 | 1.58E-10 | 16 | GO:0030219 megakaryocyte differentiation |
| 14 | 4.61E-10 | 7 | GO:0001815 positive reg. of antibody-dependent cellular cytotoxicity |
| 15 | 1.74E-09 | 10 | GO:0006388 tRNA splicing via endonucleolytic cleavage and ligation |
| 16 | 1.97E-09 | 154 | GO:0048869 cellular developmental proc. |
| 17 | 2.21E-09 | 153 | GO:0030154 cell differentiation |
| 18 | 2.21E-09 | 7 | GO:0032639 TRAIL production |
| 19 | 2.21E-09 | 7 | GO:0032679 reg. of TRAIL production |
| 20 | 2.21E-09 | 10 | GO:0036151 phosphatidylcholine acyl-chain remodeling |
| 21 | 2.80E-09 | 17 | GO:0045638 negative reg. of myeloid cell differentiation |
| 22 | 3.62E-09 | 12 | GO:0060563 neuroepithelial cell differentiation |
| 23 | 7.74E-09 | 7 | GO:0001798 positive reg. of type IIa hypersensitivity |
| 24 | 7.74E-09 | 7 | GO:0002519 natural killer cell tolerance induction |
| 25 | 7.74E-09 | 18 | GO:0034502 protein localization to chromosome |
| 26 | 1.15E-08 | 8 | GO:0071895 odontoblast differentiation |
| 27 | 1.18E-08 | 11 | GO:0021801 cerebral cortex radial glia-guided migration |
| 28 | 1.18E-08 | 11 | GO:0022030 telencephalon glial cell migration |
| 29 | 1.58E-08 | 151 | GO:0048731 system development |
| 30 | 1.99E-08 | 7 | GO:0001788 antibody-dependent cellular cytotoxicity |
| 31 | 1.99E-08 | 7 | GO:0002892 reg. of type II hypersensitivity |
| 32 | 1.99E-08 | 7 | GO:2000566 positive reg. of CD8-positive alpha-beta T cell proliferation |
| 33 | 3.76E-08 | 8 | GO:2001187 positive reg. of CD8-positive alpha-beta T cell activation |
| 34 | 4.29E-08 | 18 | GO:0006334 nucleosome assembly |
| 35 | 4.84E-08 | 7 | GO:0001794 type IIa hypersensitivity |
| 36 | 4.84E-08 | 7 | GO:0002445 type II hypersensitivity |
| 37 | 5.06E-08 | 10 | GO:0032616 interleukin-13 production |
| 38 | 5.06E-08 | 10 | GO:0032656 reg. of interleukin-13 production |
| 39 | 6.26E-08 | 105 | GO:0009653 anatomical structure morphogenesis |
| 40 | 1.04E-07 | 7 | GO:0002885 positive reg. of hypersensitivity |
| 41 | 1.18E-07 | 11 | GO:0021799 cerebral cortex radially oriented cell migration |
| 42 | 1.29E-07 | 12 | GO:0021795 cerebral cortex cell migration |
| 43 | 1.89E-07 | 22 | GO:0031497 chromatin assembly |
| 44 | 2.36E-07 | 22 | GO:0045637 reg. of myeloid cell differentiation |
| 45 | 2.61E-07 | 19 | GO:0006352 DNA-templated transcription initiation |
| 46 | 3.57E-07 | 11 | GO:0090207 reg. of triglyceride metabolic proc. |
| 47 | 3.63E-07 | 7 | GO:0002866 positive reg. of acute inflammatory response to antigenic stimulus |
| 48 | 3.63E-07 | 7 | GO:0035740 CD8-positive alpha-beta T cell proliferation |
| 49 | 3.76E-07 | 8 | GO:0032736 positive reg. of interleukin-13 production |
| 50 | 4.51E-07 | 19 | GO:0034728 nucleosome organization |
| 51 | 6.23E-07 | 7 | GO:0002477 antigen processing and presentation of exogenous peptide antigen via MHC class Ib |
| 52 | 6.23E-07 | 7 | GO:0002883 reg. of hypersensitivity |
| 53 | 8.80E-07 | 49 | GO:0051093 negative reg. of developmental proc. |
| 54 | 1.05E-06 | 7 | GO:0032819 positive reg. of natural killer cell proliferation |
| 55 | 1.08E-06 | 16 | GO:0021987 cerebral cortex development |
| 56 | 1.29E-06 | 16 | GO:0042267 natural killer cell mediated cytotoxicity |
| 57 | 1.29E-06 | 24 | GO:0071824 protein-DNA complex subunit organization |
| 58 | 1.46E-06 | 22 | GO:0065004 protein-DNA complex assembly |
| 59 | 1.72E-06 | 16 | GO:0002228 natural killer cell mediated immunity |
| 60 | 1.78E-06 | 12 | GO:0022029 telencephalon cell migration |
| 61 | 1.79E-06 | 19 | GO:0032200 telomere organization |
| 62 | 2.72E-06 | 12 | GO:0021885 forebrain cell migration |
| 63 | 3.52E-06 | 7 | GO:0002524 hypersensitivity |
| 64 | 3.52E-06 | 12 | GO:0030199 collagen fibril organization |
| 65 | 3.52E-06 | 7 | GO:0042270 protection from natural killer cell mediated cytotoxicity |
| 66 | 3.52E-06 | 13 | GO:0046470 phosphatidylcholine metabolic proc. |
| 67 | 4.08E-06 | 9 | GO:0032633 interleukin-4 production |
| 68 | 4.08E-06 | 9 | GO:0032673 reg. of interleukin-4 production |
| 69 | 4.69E-06 | 37 | GO:0051301 cell division |
| 70 | 5.48E-06 | 13 | GO:0090502 RNA phosphodiester bond hydrolysis endonucleolytic |
| 71 | 5.91E-06 | 93 | GO:0050793 reg. of developmental proc. |
| 72 | 6.06E-06 | 64 | GO:0007010 cytoskeleton organization |
| 73 | 6.33E-06 | 120 | GO:0048513 animal organ development |
| 74 | 6.43E-06 | 24 | GO:0006338 chromatin remodeling |
| 75 | 6.91E-06 | 7 | GO:0001787 natural killer cell proliferation |
| 76 | 7.38E-06 | 130 | GO:0006996 organelle organization |
| 77 | 8.43E-06 | 11 | GO:0008347 glial cell migration |
| 78 | 1.00E-05 | 8 | GO:0032753 positive reg. of interleukin-4 production |
| 79 | 1.12E-05 | 38 | GO:0045596 negative reg. of cell differentiation |
| 80 | 1.15E-05 | 66 | GO:0045595 reg. of cell differentiation |
| 81 | 1.31E-05 | 14 | GO:0002065 columnar/cuboidal epithelial cell differentiation |
| 82 | 1.36E-05 | 18 | GO:0001909 leukocyte mediated cytotoxicity |
| 83 | 2.09E-05 | 17 | GO:0021543 pallium development |
| 84 | 2.18E-05 | 45 | GO:0000278 mitotic cell cycle |
| 85 | 2.48E-05 | 8 | GO:0036037 CD8-positive alpha-beta T cell activation |
| 86 | 2.72E-05 | 13 | GO:0006641 triglyceride metabolic proc. |
| 87 | 3.14E-05 | 7 | GO:0002888 positive reg. of myeloid leukocyte mediated immunity |
| 88 | 5.78E-05 | 74 | GO:0009888 tissue development |
| 89 | 8.79E-05 | 40 | GO:0097435 supramolecular fiber organization |
| 90 | 0.00010455 | 57 | GO:2000026 reg. of multicellular organismal development |
| 91 | 0.00013649 | 64 | GO:0022008 neurogenesis |
| 92 | 0.00013663 | 8 | GO:0045954 positive reg. of natural killer cell mediated cytotoxicity |
| 93 | 0.00016096 | 8 | GO:0001916 positive reg. of T cell mediated cytotoxicity |
| 94 | 0.00028177 | 47 | GO:0051276 chromosome organization |
| 95 | 0.00033424 | 25 | GO:1903706 reg. of hemopoiesis |
| 96 | 0.00036672 | 3 | GO:1905935 positive reg. of cell fate determination |
| 97 | 0.00036928 | 5 | GO:0048251 elastic fiber assembly |
| 98 | 0.00038336 | 47 | GO:0009790 embryo development |
| 99 | 0.00039903 | 67 | GO:0007049 cell cycle |
| 100 | 0.00045833 | 13 | GO:0006639 acylglycerol metabolic proc. |
| 101 | 0.00046424 | 23 | GO:0030900 forebrain development |
| 102 | 0.00048036 | 13 | GO:0006638 neutral lipid metabolic proc. |
| 103 | 0.0005175 | 34 | GO:0030855 epithelial cell differentiation |
| 104 | 0.00060905 | 18 | GO:0001906 cell killing |
| 105 | 0.00066007 | 14 | GO:0090501 RNA phosphodiester bond hydrolysis |
| 106 | 0.00068098 | 32 | GO:0006325 chromatin organization |
| 107 | 0.00076213 | 40 | GO:0033365 protein localization to organelle |
| 108 | 0.00076213 | 4 | GO:1904026 reg. of collagen fibril organization |
| 109 | 0.0009154 | 8 | GO:0002639 positive reg. of immunoglobulin production |
| 110 | 0.00093346 | 47 | GO:0048646 anatomical structure formation involved in morphogenesis |
| 111 | 0.0009498 | 9 | GO:0002711 positive reg. of T cell mediated immunity |
| 112 | 0.00126347 | 43 | GO:0002520 immune system development |
| 113 | 0.00139801 | 9 | GO:0001912 positive reg. of leukocyte mediated cytotoxicity |
| 114 | 0.00148621 | 9 | GO:0001913 T cell mediated cytotoxicity |
| 115 | 0.00148621 | 7 | GO:0002714 positive reg. of B cell mediated immunity |
| 116 | 0.00148621 | 7 | GO:0002891 positive reg. of immunoglobulin mediated immune response |
| 117 | 0.00148621 | 20 | GO:0030198 extracellular matrix organization |
| 118 | 0.00149777 | 20 | GO:0043062 extracellular structure organization |
| 119 | 0.00164941 | 7 | GO:0002370 natural killer cell cytokine production |
| 120 | 0.00164941 | 20 | GO:0045229 external encapsulating structure organization |
| 121 | 0.00164941 | 47 | GO:0060429 epithelium development |
| 122 | 0.00170937 | 23 | GO:0030099 myeloid cell differentiation |
| 123 | 0.00174016 | 17 | GO:0021537 telencephalon development |
| 124 | 0.00183908 | 7 | GO:0002507 tolerance induction |
| 125 | 0.0019902 | 110 | GO:0010604 positive reg. of macromolecule metabolic proc. |
| 126 | 0.00220227 | 9 | GO:0046635 positive reg. of alpha-beta T cell activation |
| 127 | 0.00222821 | 12 | GO:0008033 tRNA processing |
| 128 | 0.00222821 | 78 | GO:0051128 reg. of cellular component organization |
| 129 | 0.00236189 | 9 | GO:0031343 positive reg. of cell killing |
| 130 | 0.00237165 | 34 | GO:1903047 mitotic cell cycle proc. |
| 131 | 0.00239107 | 30 | GO:0000226 microtubule cytoskeleton organization |
| 132 | 0.00306346 | 80 | GO:0007399 nervous system development |
| 133 | 0.00317574 | 32 | GO:0030036 actin cytoskeleton organization |
| 134 | 0.00326666 | 30 | GO:0009792 embryo development ending in birth or egg hatching |
| 135 | 0.00332175 | 6 | GO:0033260 nuclear DNA replication |
| 136 | 0.00356945 | 23 | GO:0044772 mitotic cell cycle phase transition |
| 137 | 0.00375231 | 20 | GO:0002449 lymphocyte mediated immunity |
| 138 | 0.00395358 | 7 | GO:0032814 reg. of natural killer cell activation |
| 139 | 0.00395358 | 59 | GO:0070727 cellular macromolecule localization |
| 140 | 0.0041906 | 29 | GO:0043009 chordate embryonic development |
| 141 | 0.00439147 | 19 | GO:0019216 reg. of lipid metabolic proc. |
| 142 | 0.00441642 | 15 | GO:0046474 glycerophospholipid biosynthetic proc. |
| 143 | 0.00445726 | 34 | GO:0030029 actin filament-based proc. |
| 144 | 0.00457682 | 16 | GO:0090068 positive reg. of cell cycle proc. |
| 145 | 0.0046311 | 3 | GO:1904028 positive reg. of collagen fibril organization |
| 146 | 0.00480916 | 9 | GO:1901992 positive reg. of mitotic cell cycle phase transition |
| 147 | 0.00505306 | 10 | GO:0007088 reg. of mitotic nuclear division |
| 148 | 0.00506253 | 45 | GO:0033043 reg. of organelle organization |
| 149 | 0.00511944 | 7 | GO:0002673 reg. of acute inflammatory response |
| 150 | 0.00525681 | 6 | GO:0044786 cell cycle DNA replication |
| 151 | 0.00529677 | 4 | GO:0030174 reg. of DNA-templated DNA replication initiation |
| 152 | 0.00559979 | 7 | GO:0045953 negative reg. of natural killer cell mediated cytotoxicity |
| 153 | 0.0056512 | 58 | GO:0034613 cellular protein localization |
| 154 | 0.00582849 | 8 | GO:0021536 diencephalon development |
| 155 | 0.00582943 | 79 | GO:0008104 protein localization |
| 156 | 0.00593669 | 96 | GO:0022607 cellular component assembly |
| 157 | 0.00652602 | 11 | GO:0051783 reg. of nuclear division |
| 158 | 0.00657663 | 7 | GO:0002483 antigen processing and presentation of endogenous peptide antigen |
| 159 | 0.00657663 | 4 | GO:0009886 post-embryonic animal morphogenesis |
| 160 | 0.00657663 | 90 | GO:0051641 cellular localization |
| 161 | 0.00658218 | 18 | GO:0042063 gliogenesis |
| 162 | 0.00671834 | 51 | GO:0048699 generation of neurons |
| 163 | 0.00704033 | 40 | GO:0009887 animal organ morphogenesis |
| 164 | 0.00735592 | 27 | GO:0048598 embryonic morphogenesis |
| 165 | 0.00832847 | 96 | GO:0051173 positive reg. of nitrogen compound metabolic proc. |
| 166 | 0.00900025 | 19 | GO:0051098 reg. of binding |
| 167 | 0.00930597 | 9 | GO:0032963 collagen metabolic proc. |
| 168 | 0.00945811 | 33 | GO:0060322 head development |
| 169 | 0.00984847 | 23 | GO:0022411 cellular component disassembly |
| 170 | 0.00988974 | 7 | GO:0001911 negative reg. of leukocyte mediated cytotoxicity |
| 171 | 0.00988974 | 10 | GO:0045931 positive reg. of mitotic cell cycle |
| 172 | 0.01041264 | 3 | GO:0048050 post-embryonic eye morphogenesis |
| 173 | 0.01041264 | 3 | GO:0048341 paraxial mesoderm formation |
| 174 | 0.01041264 | 3 | GO:1903896 positive reg. of IRE1-mediated unfolded protein response |
| 175 | 0.0107709 | 9 | GO:0002377 immunoglobulin production |
| 176 | 0.01126299 | 8 | GO:0000281 mitotic cytokinesis |
| 177 | 0.01186978 | 24 | GO:0001501 skeletal system development |
| 178 | 0.01227103 | 7 | GO:0031342 negative reg. of cell killing |
| 179 | 0.01256607 | 13 | GO:0030705 cytoskeleton-dependent intracellular transport |
| 180 | 0.0127069 | 9 | GO:0021761 limbic system development |
| 181 | 0.01343061 | 56 | GO:0007267 cell-cell signaling |
| 182 | 0.01357095 | 20 | GO:0001503 ossification |
| 183 | 0.01367992 | 10 | GO:0007498 mesoderm development |
| 184 | 0.01378056 | 31 | GO:0007420 brain development |
| 185 | 0.01390229 | 30 | GO:0010564 reg. of cell cycle proc. |
| 186 | 0.01390229 | 36 | GO:0030097 hemopoiesis |
| 187 | 0.01390229 | 4 | GO:1901970 positive reg. of mitotic sister chromatid separation |
| 188 | 0.01390966 | 7 | GO:0019731 antibacterial humoral response |
| 189 | 0.01516599 | 8 | GO:0009791 post-embryonic development |
| 190 | 0.01546597 | 88 | GO:0033036 macromolecule localization |
| 191 | 0.01582989 | 39 | GO:0035295 tube development |
| 192 | 0.01603584 | 15 | GO:0045017 glycerolipid biosynthetic proc. |
| 193 | 0.01603584 | 18 | GO:0045787 positive reg. of cell cycle |
| 194 | 0.01618755 | 10 | GO:0006661 phosphatidylinositol biosynthetic proc. |
| 195 | 0.01624527 | 19 | GO:0001701 in utero embryonic development |
| 196 | 0.01624527 | 4 | GO:0007063 reg. of sister chromatid cohesion |
| 197 | 0.01634464 | 20 | GO:0046486 glycerolipid metabolic proc. |
| 198 | 0.01825437 | 85 | GO:0051239 reg. of multicellular organismal proc. |
| 199 | 0.01878142 | 13 | GO:0006399 tRNA metabolic proc. |
| 200 | 0.01905267 | 17 | GO:0006650 glycerophospholipid metabolic proc. |
| 201 | 0.01911425 | 6 | GO:0072132 mesenchyme morphogenesis |
| 202 | 0.01951636 | 29 | GO:0001944 vasculature development |
| 203 | 0.01976842 | 5 | GO:0006270 DNA replication initiation |
| 204 | 0.01976842 | 8 | GO:2001251 negative reg. of chromosome organization |
| 205 | 0.01997111 | 15 | GO:0008654 phospholipid biosynthetic proc. |
| 206 | 0.02083704 | 17 | GO:1901990 reg. of mitotic cell cycle phase transition |
| 207 | 0.02089921 | 12 | GO:0007369 gastrulation |
| 208 | 0.02095856 | 28 | GO:0001568 blood vessel development |
| 209 | 0.02161325 | 12 | GO:0046631 alpha-beta T cell activation |
| 210 | 0.02180178 | 7 | GO:0001707 mesoderm formation |
| 211 | 0.02236068 | 9 | GO:0032760 positive reg. of tumor necrosis factor production |
| 212 | 0.02309824 | 12 | GO:0000070 mitotic sister chromatid segregation |
| 213 | 0.02309824 | 13 | GO:0000819 sister chromatid segregation |
| 214 | 0.02309824 | 13 | GO:0001649 osteoblast differentiation |
| 215 | 0.02309824 | 10 | GO:0002456 T cell mediated immunity |
| 216 | 0.02309824 | 21 | GO:0008380 RNA splicing |
| 217 | 0.02309824 | 3 | GO:0031642 negative reg. of myelination |
| 218 | 0.02309824 | 3 | GO:0048340 paraxial mesoderm morphogenesis |
| 219 | 0.02309824 | 2 | GO:0052746 inositol phosphorylation |
| 220 | 0.02309824 | 2 | GO:0071109 superior temporal gyrus development |
| 221 | 0.02309824 | 2 | GO:0071163 DNA replication preinitiation complex assembly |
| 222 | 0.02309824 | 3 | GO:1904672 reg. of somatic stem cell population maintenance |
| 223 | 0.02309824 | 2 | GO:1905162 reg. of phagosome maturation |
| 224 | 0.0234602 | 98 | GO:0044085 cellular component biogenesis |
| 225 | 0.0236147 | 36 | GO:0048534 hematopoietic or lymphoid organ development |
| 226 | 0.02363647 | 7 | GO:0048332 mesoderm morphogenesis |
| 227 | 0.02452243 | 6 | GO:1903053 reg. of extracellular matrix organization |
| 228 | 0.02522622 | 22 | GO:0007346 reg. of mitotic cell cycle |
| 229 | 0.02589155 | 21 | GO:0002443 leukocyte mediated immunity |
| 230 | 0.02676748 | 22 | GO:0006397 mRNA processing |
| 231 | 0.02692245 | 9 | GO:0002824 positive reg. of adaptive immune response based on somatic recombination of immune recept |
| 232 | 0.02711701 | 12 | GO:0007051 spindle organization |
| 233 | 0.02712107 | 4 | GO:0021854 hypothalamus development |
| 234 | 0.02712107 | 15 | GO:0045165 cell fate commitment |
| 235 | 0.02795854 | 9 | GO:0030282 bone mineralization |
| 236 | 0.02812788 | 3 | GO:0000727 double-strand break repair via break-induced replication |
| 237 | 0.02812788 | 3 | GO:0006020 inositol metabolic proc. |
| 238 | 0.02812788 | 46 | GO:0030182 neuron differentiation |
| 239 | 0.02812788 | 3 | GO:0048563 post-embryonic animal organ morphogenesis |
| 240 | 0.02812788 | 3 | GO:0070940 dephosphorylation of RNA polymerase II C-terminal domain |
| 241 | 0.02923417 | 11 | GO:0072331 signal transduction by p53 class mediator |
| 242 | 0.03103903 | 78 | GO:0051246 reg. of protein metabolic proc. |
| 243 | 0.03187208 | 14 | GO:0033044 reg. of chromosome organization |
| 244 | 0.03187208 | 18 | GO:1902903 reg. of supramolecular fiber organization |
| 245 | 0.03330169 | 16 | GO:0140014 mitotic nuclear division |
| 246 | 0.03379301 | 14 | GO:0090305 nucleic acid phosphodiester bond hydrolysis |
| 247 | 0.03384412 | 50 | GO:0043067 reg. of programmed cell death |
| 248 | 0.03430962 | 9 | GO:0002821 positive reg. of adaptive immune response |
| 249 | 0.03437473 | 4 | GO:0030262 apoptotic nuclear changes |
| 250 | 0.03465106 | 3 | GO:0021794 thalamus development |
| 251 | 0.03471067 | 7 | GO:0002437 inflammatory response to antigenic stimulus |
| 252 | 0.03548468 | 12 | GO:0043281 reg. of cysteine-type endopeptidase activity involved in apoptotic proc. |
| 253 | 0.03654736 | 49 | GO:0042981 reg. of apoptotic proc. |
| 254 | 0.03840596 | 42 | GO:0045944 positive reg. of transcription by RNA polymerase II |
| 255 | 0.03840596 | 13 | GO:0048863 stem cell differentiation |
| 256 | 0.03840596 | 4 | GO:0090382 phagosome maturation |
| 257 | 0.03840596 | 4 | GO:1903055 positive reg. of extracellular matrix organization |
| 258 | 0.03869716 | 28 | GO:0008610 lipid biosynthetic proc. |
| 259 | 0.03943935 | 13 | GO:2000116 reg. of cysteine-type endopeptidase activity |
| 260 | 0.03950236 | 96 | GO:0031325 positive reg. of cellular metabolic proc. |
| 261 | 0.03950236 | 2 | GO:1905643 positive reg. of DNA methylation |
| 262 | 0.03968897 | 62 | GO:0008283 cell population proliferation |
| 263 | 0.0397375 | 15 | GO:0001822 kidney development |
| 264 | 0.0397375 | 37 | GO:0007417 central nervous system development |
| 265 | 0.0397375 | 42 | GO:0022402 cell cycle proc. |
| 266 | 0.04078939 | 5 | GO:0008608 attachment of spindle microtubules to kinetochore |
| 267 | 0.04089849 | 3 | GO:1902969 mitotic DNA replication |
| 268 | 0.04111792 | 10 | GO:0002708 positive reg. of lymphocyte mediated immunity |
| 269 | 0.04111792 | 72 | GO:0032268 reg. of cellular protein metabolic proc. |
| 270 | 0.04124414 | 25 | GO:0090407 organophosphate biosynthetic proc. |
| 271 | 0.04146872 | 4 | GO:0006921 cellular component disassembly involved in execution phase of apoptosis |
| 272 | 0.04146872 | 4 | GO:0010971 positive reg. of G2/M transition of mitotic cell cycle |
| 273 | 0.04146872 | 4 | GO:1905820 positive reg. of chromosome separation |
| 274 | 0.04282553 | 5 | GO:0007094 mitotic spindle assembly checkpoint signaling |
| 275 | 0.04282553 | 29 | GO:0016071 mRNA metabolic proc. |
| 276 | 0.04282553 | 58 | GO:0051254 positive reg. of RNA metabolic proc. |
| 277 | 0.04282553 | 8 | GO:0061640 cytoskeleton-dependent cytokinesis |
| 278 | 0.04483664 | 31 | GO:0035239 tube morphogenesis |
| 279 | 0.04494706 | 9 | GO:0042102 positive reg. of T cell proliferation |
| 280 | 0.04609899 | 5 | GO:0007019 microtubule depolymerization |
| 281 | 0.04609899 | 5 | GO:0031577 spindle checkpoint signaling |
| 282 | 0.04609899 | 10 | GO:0046488 phosphatidylinositol metabolic proc. |
| 283 | 0.04609899 | 5 | GO:0085029 extracellular matrix assembly |
| 284 | 0.04742864 | 3 | GO:1903543 positive reg. of exosomal secretion |
| 285 | 0.04753737 | 7 | GO:0030071 reg. of mitotic metaphase/anaphase transition |
| 286 | 0.04772224 | 15 | GO:0072001 renal system development |
| 287 | 0.04773928 | 61 | GO:0012501 programmed cell death |
| 288 | 0.04773928 | 9 | GO:0035107 appendage morphogenesis |
| 289 | 0.04773928 | 9 | GO:0035108 limb morphogenesis |
| 290 | 0.04897863 | 33 | GO:0043069 negative reg. of programmed cell death |
| 291 | 0.04897863 | 5 | GO:0045841 negative reg. of mitotic metaphase/anaphase transition |
| 292 | 0.04897863 | 24 | GO:0048514 blood vessel morphogenesis |
| 293 | 0.04933172 | 9 | GO:0010212 response to ionizing radiation |
| 294 | 0.04990606 | 113 | GO:0034654 nucleobase-containing compound biosynthetic proc. |
