# Supplementary material for: Using systems biology and drug repositioning approaches to discover FDA-approved drugs candidates for endometriosis treatment
Source: PLoS One. 2025 Sep 12;20(9):e0330841. doi: 10.1371/journal.pone.0330841 (PMC12431326; doi:10.1371/journal.pone.0330841)
Supplement: S6 Table — (DOCX) [file pone.0330841.s006.docx]

**Table S7**

The list of functional analysis of enriched GO MF terms of common down-regulated DEGs between the FE and IE groups.

| **Number** | **Enrichment FDR** | **nGenes** | **Pathway** |
| --- | --- | --- | --- |
| 1 | 8.68E-14 | 10 | GO:0000213 tRNA-intron endonuclease activity |
| 2 | 2.89E-11 | 10 | GO:0016892 endoribonuclease activity producing 3-prime-phosphomonoesters |
| 3 | 5.96E-11 | 15 | GO:0042288 MHC class I protein binding |
| 4 | 7.61E-11 | 10 | GO:0047144 2-acylglycerol-3-phosphate O-acyltransferase activity |
| 5 | 2.45E-09 | 10 | GO:0016894 endonuclease activity active with either ribo- or deoxyribonucleic acids and producing 3- |
| 6 | 1.41E-08 | 11 | GO:0003841 1-acylglycerol-3-phosphate O-acyltransferase activity |
| 7 | 1.63E-08 | 10 | GO:0004549 tRNA-specific ribonuclease activity |
| 8 | 2.40E-08 | 11 | GO:0042171 lysophosphatidic acid acyltransferase activity |
| 9 | 2.40E-08 | 11 | GO:0071617 lysophospholipid acyltransferase activity |
| 10 | 1.07E-07 | 15 | GO:0042287 MHC protein binding |
| 11 | 1.66E-07 | 11 | GO:0016411 acylglycerol O-acyltransferase activity |
| 12 | 1.81E-06 | 45 | GO:0005198 structural molecule activity |
| 13 | 1.81E-06 | 12 | GO:0008374 O-acyltransferase activity |
| 14 | 1.81E-06 | 14 | GO:0030527 structural constituent of chromatin |
| 15 | 1.81E-06 | 7 | GO:0030881 beta-2-microglobulin binding |
| 16 | 5.17E-06 | 12 | GO:0004521 endoribonuclease activity |
| 17 | 5.54E-06 | 7 | GO:0046703 natural killer cell lectin-like receptor binding |
| 18 | 7.26E-06 | 4 | GO:0098633 collagen fibril binding |
| 19 | 1.01E-05 | 7 | GO:0042608 T cell receptor binding |
| 20 | 2.09E-05 | 13 | GO:0005200 structural constituent of cytoskeleton |
| 21 | 8.33E-05 | 23 | GO:0046982 protein heterodimerization activity |
| 22 | 0.0001107 | 13 | GO:0004540 ribonuclease activity |
| 23 | 0.00042764 | 126 | GO:0003676 nucleic acid binding |
| 24 | 0.00081864 | 33 | GO:0019904 protein domain specific binding |
| 25 | 0.00088567 | 12 | GO:0004519 endonuclease activity |
| 26 | 0.0008902 | 63 | GO:0003723 RNA binding |
| 27 | 0.00261529 | 43 | GO:0046983 protein dimerization activity |
| 28 | 0.00383764 | 19 | GO:0044389 ubiquitin-like protein ligase binding |
| 29 | 0.00524674 | 15 | GO:0016747 acyltransferase activity transferring groups other than amino-acyl groups |
| 30 | 0.01187536 | 79 | GO:0003677 DNA binding |
| 31 | 0.01325665 | 11 | GO:0140101 catalytic activity acting on a tRNA |
| 32 | 0.01473741 | 17 | GO:0031625 ubiquitin protein ligase binding |
| 33 | 0.01578461 | 15 | GO:0016746 acyltransferase activity |
| 34 | 0.01782391 | 48 | GO:0044877 protein-containing complex binding |
| 35 | 0.01925979 | 13 | GO:0004518 nuclease activity |
| 36 | 0.02343983 | 66 | GO:0019899 enzyme binding |
| 37 | 0.02406893 | 2 | GO:0000825 inositol tetrakisphosphate 6-kinase activity |
| 38 | 0.02406893 | 2 | GO:0004347 glucose-6-phosphate isomerase activity |
| 39 | 0.02406893 | 2 | GO:0004461 lactose synthase activity |
| 40 | 0.02406893 | 2 | GO:0051765 inositol tetrakisphosphate kinase activity |
| 41 | 0.02622828 | 5 | GO:0043028 cysteine-type endopeptidase regulator activity involved in apoptotic proc. |
| 42 | 0.03144808 | 7 | GO:0042605 peptide antigen binding |
| 43 | 0.04418149 | 2 | GO:0003831 beta-N-acetylglucosaminylglycopeptide beta-1,4-galactosyltransferase activity |
| 44 | 0.04574646 | 3 | GO:0008420 RNA polymerase II CTD heptapeptide repeat phosphatase activity |
