# Supplementary material for: Using systems biology and drug repositioning approaches to discover FDA-approved drugs candidates for endometriosis treatment
Source: PLoS One. 2025 Sep 12;20(9):e0330841. doi: 10.1371/journal.pone.0330841 (PMC12431326; doi:10.1371/journal.pone.0330841)
Supplement: S7 Table — (DOCX) [file pone.0330841.s007.docx]

**Table S8**

The list of functional analysis of enriched GO CC terms of common down-regulated DEGs between the FE and IE groups.

| **Number** | **Enrichment FDR** | **nGenes** | **Pathway** |
| --- | --- | --- | --- |
| 1 | 1.32E-18 | 14 | GO:0043505 CENP-A containing nucleosome |
| 2 | 1.32E-18 | 14 | GO:0061638 CENP-A containing chromatin |
| 3 | 1.07E-17 | 14 | GO:0034506 chromosome centromeric core domain |
| 4 | 4.20E-13 | 10 | GO:0000214 tRNA-intron endonuclease complex |
| 5 | 1.40E-12 | 13 | GO:0044233 mitochondria-associated endoplasmic reticulum membrane |
| 6 | 1.22E-11 | 100 | GO:0070062 extracellular exosome |
| 7 | 1.66E-11 | 100 | GO:0043230 extracellular organelle |
| 8 | 1.66E-11 | 100 | GO:0065010 extracellular membrane-bounded organelle |
| 9 | 1.66E-11 | 100 | GO:1903561 extracellular vesicle |
| 10 | 3.61E-10 | 27 | GO:0000775 chromosome centromeric region |
| 11 | 4.66E-10 | 27 | GO:0000228 nuclear chromosome |
| 12 | 4.66E-10 | 34 | GO:0098687 chromosomal region |
| 13 | 1.57E-09 | 22 | GO:0000781 chromosome telomeric region |
| 14 | 5.88E-09 | 13 | GO:0044232 organelle membrane contact site |
| 15 | 3.87E-08 | 8 | GO:0005641 nuclear envelope lumen |
| 16 | 4.70E-08 | 122 | GO:0005615 extracellular space |
| 17 | 7.26E-08 | 7 | GO:0032398 MHC class Ib protein complex |
| 18 | 3.69E-07 | 143 | GO:0005654 nucleoplasm |
| 19 | 1.26E-06 | 150 | GO:0031981 nuclear lumen |
| 20 | 1.26E-06 | 10 | GO:1902555 endoribonuclease complex |
| 21 | 1.60E-06 | 76 | GO:0005694 chromosome |
| 22 | 2.16E-06 | 137 | GO:0031982 vesicle |
| 23 | 3.78E-06 | 20 | GO:0032993 protein-DNA complex |
| 24 | 5.68E-06 | 140 | GO:0005576 extracellular region |
| 25 | 2.00E-05 | 7 | GO:0042612 MHC class I protein complex |
| 26 | 2.10E-05 | 14 | GO:0000786 nucleosome |
| 27 | 2.88E-05 | 12 | GO:0045171 intercellular bridge |
| 28 | 3.13E-05 | 55 | GO:0000785 chromatin |
| 29 | 4.42E-05 | 31 | GO:0031012 extracellular matrix |
| 30 | 4.42E-05 | 31 | GO:0030312 external encapsulating structure |
| 31 | 0.00017899 | 14 | GO:0005766 primary lysosome |
| 32 | 0.00017899 | 11 | GO:0035578 azurophil granule lumen |
| 33 | 0.00017899 | 14 | GO:0042582 azurophil granule |
| 34 | 0.00022332 | 16 | GO:0044815 DNA packaging complex |
| 35 | 0.00035806 | 15 | GO:0072686 mitotic spindle |
| 36 | 0.00069807 | 18 | GO:0035770 ribonucleoprotein granule |
| 37 | 0.00111546 | 10 | GO:0031970 organelle envelope lumen |
| 38 | 0.00208999 | 50 | GO:0015630 microtubule cytoskeleton |
| 39 | 0.00310464 | 16 | GO:0036464 cytoplasmic ribonucleoprotein granule |
| 40 | 0.00481656 | 13 | GO:0005775 vacuolar lumen |
| 41 | 0.00511433 | 22 | GO:0005874 microtubule |
| 42 | 0.00541186 | 40 | GO:0005730 nucleolus |
| 43 | 0.00541186 | 10 | GO:0055038 recycling endosome membrane |
| 44 | 0.00541186 | 9 | GO:0071556 integral component of lumenal side of endoplasmic reticulum membrane |
| 45 | 0.00541186 | 50 | GO:0099080 supramolecular complex |
| 46 | 0.0056504 | 33 | GO:0099513 polymeric cytoskeletal fiber |
| 47 | 0.00641371 | 23 | GO:0005635 nuclear envelope |
| 48 | 0.00641371 | 14 | GO:0055037 recycling endosome |
| 49 | 0.00808055 | 9 | GO:0098576 lumenal side of membrane |
| 50 | 0.00987881 | 9 | GO:0042470 melanosome |
| 51 | 0.00987881 | 9 | GO:0048770 pigment granule |
| 52 | 0.01121577 | 20 | GO:0062023 collagen-containing extracellular matrix |
| 53 | 0.01270979 | 16 | GO:0005788 endoplasmic reticulum lumen |
| 54 | 0.01270979 | 20 | GO:0005819 spindle |
| 55 | 0.01315871 | 3 | GO:0071162 CMG complex |
| 56 | 0.01430868 | 73 | GO:0005856 cytoskeleton |
| 57 | 0.01487044 | 66 | GO:0005783 endoplasmic reticulum |
| 58 | 0.0149402 | 9 | GO:0030670 phagocytic vesicle membrane |
| 59 | 0.0149402 | 52 | GO:0031984 organelle subcompartment |
| 60 | 0.01586692 | 3 | GO:0031261 DNA replication preinitiation complex |
| 61 | 0.02043982 | 23 | GO:0044297 cell body |
| 62 | 0.02141382 | 11 | GO:0045335 phagocytic vesicle |
| 63 | 0.02291949 | 2 | GO:0061574 ASAP complex |
| 64 | 0.0243352 | 37 | GO:0099512 supramolecular fiber |
| 65 | 0.02734855 | 37 | GO:0099081 supramolecular polymer |
| 66 | 0.0392982 | 30 | GO:0005815 microtubule organizing center |
| 67 | 0.04738667 | 24 | GO:0005813 centrosome |
