# Supplementary material for: Using systems biology and drug repositioning approaches to discover FDA-approved drugs candidates for endometriosis treatment
Source: PLoS One. 2025 Sep 12;20(9):e0330841. doi: 10.1371/journal.pone.0330841 (PMC12431326; doi:10.1371/journal.pone.0330841)
Supplement: S8 Table — (DOCX) [file pone.0330841.s008.docx]

**Table S9**

The list of KEGG pathways involving common up-regulated DEGs between the FE and IE groups.

| **Number** | **Term** | **Count** | **P-value** | **Genes** |
| --- | --- | --- | --- | --- |
| 1 | hsa05168: Herpes simplex virus 1 infection | 43 | 4.7816E-09 | ZNF155, ZNF595, ZNF177, ZNF154, ZNF175, ZNF273, ZNF492, ZNF44, ZNF23, ZNF25, ZNF506, ZNF625, ZNF548, ZNF107, ZNF404, ZNF248, ZNF665, ZNF566, ZNF169, ZNF223, ZNF585A, ZNF222, ZNF17, ZNF440, ZNF681, ZNF680, ZNF181, ZNF99, IRAK4, IL6, ZNF738, TRAF6, ZNF439, ZNF614, FAS, ZNF678, ZNF780A, ZNF577, ZNF675, ZNF432, ZNF135, ZNF333, ZNF596 |
| 2 | hsa04010: MAPK signaling pathway | 20 | 0.00192411 | TGFB2, PTPRR, FLT1, RASGRF2, FLT3LG, FOS, IRAK4, DUSP8, RASGRP3, VEGFA, MAPK10, CACNB2, FGF7, TRAF6, RASA2, KDR, NF1, FAS, MAPT, MAP3K13 |
| 3 | hsa05142: Chagas disease | 10 | 0.00397348 | MAPK10, IL6, TGFB2, TRAF6, SERPINE1, FAS, PPP2R2A, FOS, TLR6, IRAK4 |
| 4 | hsa04820: Cytoskeleton in muscle cells | 15 | 0.01101951 | NEBL, ELN, COL11A2, MYOM2, TTN, ACTA1, FMNL1, DAAM1, COL5A3, COL4A6, DMD, COL6A5, MYOZ2, PDLIM7, ITGA9 |
| 5 | hsa05205: Proteoglycans in cancer | 13 | 0.02141854 | TGFB2, PXN, GAB1, ITPR1, VEGFA, RPS6KB1, KDR, FAS, TIMP3, NANOG, CAMK2G, CD44, HBEGF |
| 6 | hsa04151: PI3K-Akt signaling pathway | 19 | 0.02689249 | FLT1, PRKAA2, ATF6B, FLT3LG, PPP2R2A, PRL, OSMR, EPOR, VEGFA, FGF7, IL6, BCL2L11, RPS6KB1, GNG4, COL4A6, KDR, GNB3, COL6A5, ITGA9 |
| 7 | hsa04014: Ras signaling pathway | 14 | 0.02909467 | FLT1, RASGRF2, GAB1, FLT3LG, RASGRP3, VEGFA, MAPK10, FGF7, GNG4, RASA2, KDR, NF1, GNB3, ABL2 |
| 8 | hsa05410: Hypertrophic cardiomyopathy | 8 | 0.0305832 | CACNB2, IL6, TGFB2, PRKAA2, DMD, NOS1, ITGA9, TTN |
| 9 | hsa01521: EGFR tyrosine kinase inhibitor resistance | 7 | 0.03421574 | IL6, BCL2L11, RPS6KB1, GAB1, NF1, KDR, VEGFA |
| 10 | hsa04978: Mineral absorption | 6 | 0.03712065 | TF, ATP7A, TRPM6, STEAP2, SLC26A6, MT1E |
| 11 | hsa05417: Lipid and atherosclerosis | 12 | 0.06453638 | MAPK10, ABCA1, CYP2C9, IL6, TRAF6, ITPR1, FAS, FOS, TLR6, IRAK4, AGER, CAMK2G |
| 12 | hsa04722: Neurotrophin signaling pathway | 8 | 0.07228722 | MAPK10, KIDINS220, NTRK3, TRAF6, GAB1, IRAK4, CAMK2G, SH2B1 |
| 13 | hsa04713: Circadian entrainment | 7 | 0.07414187 | GNG4, NOS1AP, ITPR1, GNB3, FOS, NOS1, CAMK2G |
| 14 | hsa04640: Hematopoietic cell lineage | 7 | 0.08314032 | IL6, THPO, FLT3LG, CD44, CD33, CD55, EPOR |
| 15 | hsa04933: AGE-RAGE signaling pathway in diabetic complications | 7 | 0.08626915 | MAPK10, IL6, TGFB2, SERPINE1, COL4A6, AGER, VEGFA |
| 16 | hsa05133: Pertussis | 6 | 0.0877612 | MAPK10, IL6, TRAF6, FOS, SFTPA1, IRAK4 |
| 17 | hsa05414: Dilated cardiomyopathy | 7 | 0.09942244 | CACNB2, TGFB2, PLN, DMD, NOS1, ITGA9, TTN |
| 18 | hsa04974: Protein digestion and absorption | 7 | 0.09942244 | CPB1, ELN, COL5A3, COL11A2, COL4A6, COL6A5, SLC38A2 |
