# Supplementary material for: Using systems biology and drug repositioning approaches to discover FDA-approved drugs candidates for endometriosis treatment
Source: PLoS One. 2025 Sep 12;20(9):e0330841. doi: 10.1371/journal.pone.0330841 (PMC12431326; doi:10.1371/journal.pone.0330841)
Supplement: S9 Table — (DOCX) [file pone.0330841.s009.docx]

**Table S10**

The list of KEGG pathways involving common down-regulated DEGs between the FE and IE groups.

| **Number** | **Term** | **Count** | **P-value** | **Genes** |
| --- | --- | --- | --- | --- |
| 1 | hsa04110: Cell cycle | 16 | 8.89E-07 | CDT1, CDKN2C, CDCA5, ATRX, PLK1, PKMYT1, CDC25A, TICRR, CDC45, CCND2, ESPL1, CCND1, MYC, MCM3, E2F2, MCM2 |
| 2 | hsa04310: Wnt signaling pathway | 14 | 6.40658E-05 | MMP7, CTBP1, LEF1, CBY1, WNT6, SFRP4, SFRP1, WNT11, CCND2, SOX17, CCND1, TBL1XR1, MYC, WNT4 |
| 3 | hsa04390: Hippo signaling pathway | 13 | 9.88641E-05 | LEF1, BMP8B, PRKCZ, BBC3, PPP1CA, WNT6, WNT11, CCND2, PPP2R2C, CCND1, MYC, BIRC5, WNT4 |
| 4 | hsa04210: Apoptosis | 12 | 0.000116842 | HRK, TUBA1C, DFFA, TUBA1A, BCL2A1, BAX, BIRC5, FADD, CTSC, LMNB2, LMNB1, BBC3 |
| 5 | hsa05166: Human T-cell leukemia virus 1 infection | 12 | 0.006943385 | NRP1, CDKN2C, MMP7, CCND2, CCND1, ESPL1, MYC, CANX, BAX, E2F2, TCF3, HLA-E |
| 6 | hsa04530: Tight junction | 10 | 0.008874316 | TUBA1C, CLDN10, TUBA1A, PPP2R2C, CCND1, ARHGEF18, MYH10, PRKCZ, RAB8A, RUNX1 |
| 7 | hsa05217: Basal cell carcinoma | 6 | 0.009702041 | WNT6, WNT11, SMO, LEF1, BAX, WNT4 |
| 8 | hsa04218: Cellular senescence | 9 | 0.016423336 | CCND2, CCND1, RBBP4, MYC, E2F2, SQSTM1, CDC25A, PPP1CA, HLA-E |
| 9 | hsa04115: p53 signaling pathway | 6 | 0.019547995 | RRM2, CCND2, CCND1, SHISA5, BAX, BBC3 |
| 10 | hsa05220: Chronic myeloid leukemia | 6 | 0.021654785 | CCND1, CTBP1, MYC, BAX, E2F2, RUNX1 |
| 11 | hsa05202: Transcriptional misregulation in cancer | 10 | 0.02217356 | CDKN2C, CCND2, BCL2A1, KMT2A, SIN3A, MYC, BAX, TCF3, AFF1, RUNX1 |
| 12 | hsa05225: Hepatocellular carcinoma | 9 | 0.025185904 | WNT6, WNT11, SMARCD3, CCND1, MYC, LEF1, BAX, E2F2, WNT4 |
| 13 | hsa05205: Proteoglycans in cancer | 10 | 0.026176108 | WNT6, WNT11, SMO, CCND1, MYC, TWIST1, SDC1, NUDT16L1, WNT4, PPP1CA |
| 14 | hsa04215: Apoptosis - multiple species | 4 | 0.028067992 | BAX, BIRC5, FADD, BBC3 |
| 15 | hsa04340: Hedgehog signaling pathway | 5 | 0.028555802 | GRK3, CCND2, SMO, CCND1, CSNK1G2 |
| 16 | hsa05224: Breast cancer | 8 | 0.034313858 | WNT6, WNT11, CCND1, MYC, LEF1, BAX, E2F2, WNT4 |
| 17 | hsa05210: Colorectal cancer | 6 | 0.034384569 | CCND1, MYC, LEF1, BAX, BIRC5, BBC3 |
| 18 | hsa05226: Gastric cancer | 8 | 0.036507441 | WNT6, WNT11, CCND1, MYC, LEF1, BAX, E2F2, WNT4 |
| 19 | hsa03030: DNA replication | 4 | 0.03805758 | POLD3, FEN1, MCM3, MCM2 |
| 20 | hsa05216: Thyroid cancer | 4 | 0.040802371 | CCND1, MYC, LEF1, BAX |
| 21 | hsa04934: Cushing syndrome | 8 | 0.042391568 | WNT6, CDKN2C, WNT11, CCND1, KMT2A, LEF1, E2F2, WNT4 |
| 22 | hsa05200: Pathways in cancer | 18 | 0.050706468 | CTBP1, LEF1, LAMC2, BBC3, RUNX1, WNT6, WNT11, CCND2, SMO, CCND1, MYC, GNB1, BAX, BIRC5, E2F2, FADD, FGFR3, WNT4 |
| 23 | hsa05221: Acute myeloid leukemia | 5 | 0.052416848 | CCND1, BCL2A1, MYC, LEF1, RUNX1 |
| 24 | hsa05219: Bladder cancer | 4 | 0.052744905 | CCND1, MYC, E2F2, FGFR3 |
| 25 | hsa05130: Pathogenic Escherichia coli infection | 9 | 0.06064468 | TUBA1C, CLDN10, TUBA1A, TUBB3, NCL, TUBB, BAX, FADD, MYH10 |
| 26 | hsa05169: Epstein-Barr virus infection | 9 | 0.062073137 | PSMD8, CCND2, CCND1, SIN3A, MYC, BAX, E2F2, FADD, HLA-E |
| 27 | hsa03015: mRNA surveillance pathway | 6 | 0.062826666 | PPP2R2C, ALYREF, PYM1, SSU72, RNPS1, PPP1CA |
| 28 | hsa01524: Platinum drug resistance | 5 | 0.070127476 | SLC31A1, BAX, BIRC5, FADD, BBC3 |
| 29 | hsa03013: Nucleocytoplasmic transport | 6 | 0.073801045 | NUP93, ALYREF, SUMO2, PYM1, RNPS1, THOC6 |
| 30 | hsa04550: Signaling pathways regulating pluripotency of stem cells | 7 | 0.079032453 | WNT6, WNT11, MYC, TCF3, FGFR3, ACVR2B, WNT4 |
| 31 | hsa05165: Human papillomavirus infection | 12 | 0.086129485 | WNT6, WNT11, TNXB, CCND2, PPP2R2C, CCND1, BAX, LAMC2, FADD, PRKCZ, WNT4, HLA-E |
