# Supplementary material for: Using systems biology and drug repositioning approaches to discover FDA-approved drugs candidates for endometriosis treatment
Source: PLoS One. 2025 Sep 12;20(9):e0330841. doi: 10.1371/journal.pone.0330841 (PMC12431326; doi:10.1371/journal.pone.0330841)
Supplement: S10 Table — (DOCX) [file pone.0330841.s010.docx]

**Table S11**

The top 10 ranked hub genes by the Betweenness, BottleNeck, Closeness, Degree, and Stress algorithms in the CytoHubba plugin of Cytoscape.

| **Method** | **Rank** | **Name** | **Score** |
| --- | --- | --- | --- |
| **Betweenness** | 1 | IL6 | 6235.581 |
|  | 2 | KDR | 6010.981 |
|  | 3 | TRPC1 | 5825.962 |
|  | 4 | ALB | 4448.552 |
|  | 5 | FLT3LG | 3921.202 |
|  | 6 | NOS1 | 3692.229 |
|  | 7 | CD44 | 3622.869 |
|  | 8 | PKD1 | 3120 |
|  | 9 | NPHP1 | 2904 |
|  | 10 | FYN | 2681.89 |
| **BottleNeck** | 1 | KDR | 104 |
|  | 2 | FLT3LG | 71 |
|  | 3 | IL6 | 70 |
|  | 4 | TRPC1 | 51 |
|  | 5 | FYN | 28 |
|  | 5 | ALB | 28 |
|  | 7 | ITPR1 | 20 |
|  | 8 | NOS1 | 19 |
|  | 9 | CD44 | 17 |
|  | 10 | PKD1 | 14 |
| **Closeness** | 1 | IL6 | 53.45004 |
|  | 2 | ALB | 49.38532 |
|  | 3 | CD44 | 49.25718 |
|  | 4 | FGF7 | 46.80675 |
|  | 5 | FLT3LG | 46.23452 |
|  | 6 | KDR | 45.78135 |
|  | 7 | PTGS2 | 43.63813 |
|  | 8 | FYN | 42.94167 |
|  | 9 | TRAF6 | 41.91829 |
|  | 10 | FOS | 41.00245 |
| **Degree** | 1 | IL6 | 32 |
|  | 2 | CD44 | 26 |
|  | 3 | ALB | 24 |
|  | 4 | KDR | 20 |
|  | 5 | FLT3LG | 18 |
|  | 5 | CEP290 | 18 |
|  | 7 | FGF7 | 16 |
|  | 8 | PTGS2 | 14 |
|  | 8 | FOS | 14 |
|  | 10 | XAF1 | 12 |
| **Stress** | 1 | NOS1 | 9406704 |
|  | 2 | TRPC1 | 8695152 |
|  | 3 | KDR | 8259744 |
|  | 4 | PLN | 7083960 |
|  | 5 | DMPK | 6902472 |
|  | 6 | MBNL3 | 6539256 |
|  | 7 | ITPR1 | 6112968 |
|  | 8 | QKI | 5812728 |
|  | 9 | IL6 | 4798784 |
|  | 10 | DMD | 4430200 |
