# Supplementary material for: Using systems biology and drug repositioning approaches to discover FDA-approved drugs candidates for endometriosis treatment
Source: PLoS One. 2025 Sep 12;20(9):e0330841. doi: 10.1371/journal.pone.0330841 (PMC12431326; doi:10.1371/journal.pone.0330841)
Supplement: S11 Table — (DOCX) [file pone.0330841.s011.docx]

**Table S12**

The list of nodes inside the miRNAs/TFs-IL-6 network.

| **Id** | **Label** | **Degree** | **Betweenness** |
| --- | --- | --- | --- |
| 3569 | IL6 | 40 | 820 |
| MIMAT0000062 | hsa-let-7a-5p | 1 | 0 |
| MIMAT0000064 | hsa-let-7c-5p | 1 | 0 |
| MIMAT0000067 | hsa-let-7f-5p | 1 | 0 |
| MIMAT0000073 | hsa-miR-19a-3p | 1 | 0 |
| MIMAT0000082 | hsa-miR-26a-5p | 1 | 0 |
| MIMAT0000083 | hsa-miR-26b-5p | 1 | 0 |
| MIMAT0000096 | hsa-miR-98-5p | 1 | 0 |
| MIMAT0000100 | hsa-miR-29b-3p | 1 | 0 |
| MIMAT0000103 | hsa-miR-106a-5p | 1 | 0 |
| MIMAT0000104 | hsa-miR-107 | 1 | 0 |
| MIMAT0000232 | hsa-miR-199a-3p | 1 | 0 |
| MIMAT0000264 | hsa-miR-203a-3p | 1 | 0 |
| MIMAT0000280 | hsa-miR-223-3p | 1 | 0 |
| MIMAT0000416 | hsa-miR-1-3p | 1 | 0 |
| MIMAT0000422 | hsa-miR-124-3p | 1 | 0 |
| MIMAT0000423 | hsa-miR-125b-5p | 1 | 0 |
| MIMAT0000434 | hsa-miR-142-3p | 1 | 0 |
| MIMAT0000441 | hsa-miR-9-5p | 1 | 0 |
| MIMAT0000448 | hsa-miR-136-5p | 1 | 0 |
| MIMAT0000449 | hsa-miR-146a-5p | 1 | 0 |
| MIMAT0000450 | hsa-miR-149-5p | 1 | 0 |
| MIMAT0000646 | hsa-miR-155-5p | 1 | 0 |
| MIMAT0000710 | hsa-miR-365a-3p | 1 | 0 |
| MIMAT0000728 | hsa-miR-375 | 1 | 0 |
| MIMAT0000765 | hsa-miR-335-5p | 1 | 0 |
| MIMAT0001631 | hsa-miR-451a | 1 | 0 |
| MIMAT0002809 | hsa-miR-146b-5p | 1 | 0 |
| MIMAT0003271 | hsa-miR-603 | 1 | 0 |
| MIMAT0004602 | hsa-miR-125a-3p | 1 | 0 |
| MIMAT0004608 | hsa-miR-146a-3p | 1 | 0 |
| MIMAT0004692 | hsa-miR-340-5p | 1 | 0 |
| MIMAT0004901 | hsa-miR-298 | 1 | 0 |
| MIMAT0004982 | hsa-miR-939-5p | 1 | 0 |
| 2353 | FOS | 1 | 0 |
| 3725 | JUN | 1 | 0 |
| 1385 | CREB1 | 1 | 0 |
| 2296 | FOXC1 | 1 | 0 |
| 6774 | STAT3 | 1 | 0 |
| 4790 | NFKB1 | 1 | 0 |
| 7003 | TEAD1 | 1 | 0 |
