# Supplementary material for: Using systems biology and drug repositioning approaches to discover FDA-approved drugs candidates for endometriosis treatment
Source: PLoS One. 2025 Sep 12;20(9):e0330841. doi: 10.1371/journal.pone.0330841 (PMC12431326; doi:10.1371/journal.pone.0330841)
Supplement: S12 Table — (DOCX) [file pone.0330841.s012.docx]

**Table S13**

The list of nodes inside the miRNAs/TFs-KDR network.

| **Id** | **Label** | **Degree** | **Betweenness** |
| --- | --- | --- | --- |
| 3791 | KDR | 31 | 465 |
| MIMAT0000069 | hsa-miR-16-5p | 1 | 0 |
| MIMAT0000071 | hsa-miR-17-3p | 1 | 0 |
| MIMAT0000232 | hsa-miR-199a-3p | 1 | 0 |
| MIMAT0000255 | hsa-miR-34a-5p | 1 | 0 |
| MIMAT0000318 | hsa-miR-200b-3p | 1 | 0 |
| MIMAT0000417 | hsa-miR-15b-5p | 1 | 0 |
| MIMAT0000461 | hsa-miR-195-5p | 1 | 0 |
| MIMAT0000617 | hsa-miR-200c-3p | 1 | 0 |
| MIMAT0000646 | hsa-miR-155-5p | 1 | 0 |
| MIMAT0000680 | hsa-miR-106b-5p | 1 | 0 |
| MIMAT0000690 | hsa-miR-296-5p | 1 | 0 |
| MIMAT0000763 | hsa-miR-338-3p | 1 | 0 |
| MIMAT0000765 | hsa-miR-335-5p | 1 | 0 |
| MIMAT0004491 | hsa-miR-19b-1-5p | 1 | 0 |
| MIMAT0005591 | hsa-miR-1236-3p | 1 | 0 |
| MIMAT0018447 | hsa-miR-548aa | 1 | 0 |
| MIMAT0019976 | hsa-miR-4799-5p | 1 | 0 |
| MIMAT0021038 | hsa-miR-548ap-3p | 1 | 0 |
| MIMAT0022268 | hsa-miR-548as-3p | 1 | 0 |
| MIMAT0022278 | hsa-miR-548at-3p | 1 | 0 |
| MIMAT0022730 | hsa-miR-548t-3p | 1 | 0 |
| MIMAT0025453 | hsa-miR-548ay-3p | 1 | 0 |
| 6736 | SRY | 1 | 0 |
| 6689 | SPIB | 1 | 0 |
| 2296 | FOXC1 | 1 | 0 |
| 2624 | GATA2 | 1 | 0 |
| 2300 | FOXL1 | 1 | 0 |
| 6774 | STAT3 | 1 | 0 |
| 2625 | GATA3 | 1 | 0 |
| 3169 | FOXA1 | 1 | 0 |
| 6721 | SREBF2 | 1 | 0 |
